# A Thirty-Year Survey Reveals That Ecosystem Function of Fungi Predicts Phenology of Mushroom Fruiting

**DOI:** 10.1371/journal.pone.0049777

**Published:** 2012-11-27

**Authors:** Hirotoshi Sato, Shigeo Morimoto, Tsutomu Hattori

**Affiliations:** 1 Graduate School of Global Environmental Studies, Kyoto University, Kyoto, Japan; 2 Kansai Research Center, Forestry and Forest Products Research Institute, Kyoto, Japan; 3 Youkin Mycoflora Project, Kyoto, Japan; Centro de Investigación y de Estudios Avanzados, Mexico

## Abstract

Mushroom fruiting, the reproduction of fungi, has broad implications for forest health, terrestrial biomass turnover, and global carbon cycle. However, little is known about the difference in phenology and environmental drivers of mushroom fruiting between functional guilds, e.g., ectomycorrhizal (ECM) mutualists and saprotrophs (SAP). There is a remarkable difference between ECM and SAP fungi in their available carbon sources and lifecycles, and thus these fungal groups are likely to differ in fruiting phenology. We analyzed intra- and inter-annual phenological patterns of mushroom fruiting throughout the year using a long-term census dataset of mushroom-forming fungi in a Japanese oak forest in which a total of 11,923 mushroom counts (668 species) were recorded during monthly intervals from 1982 to 2011. ECM fungi showed a unimodal seasonal fruiting peak from mid-summer to early autumn; litter-decomposing fungi showed moderate fruiting peaks from early summer or early autumn, and the phenology of wood-decomposing fungi varied considerably among the genera. Each functional group was controlled by a different set of external factors; temperature and rainfall increased ECM fungal fruiting, but key factors substantially differed among the genera of litter- and wood-decomposing fungi in taxon-specific ways. Our results suggest that fungal fruiting phenology may be affected by the seasonality of carbohydrate availability. The highly scheduled reproduction of ECM fungi may reflect temperature-dependent increases and drought-induced decreases of photosynthetic activity in host plants rather than improved growth conditions for fungi during the summer. We argue that the way a fungus obtains carbohydrates may explain a substantial fraction of the fruiting phenology, which may make a differential contribution to the community structure of fungus-associated organisms and terrestrial biomass turnover based on fungal functional groups.

## Introduction

Phenology, the seasonal timing of life-history events of organisms, generally depends on climatic conditions and has broad implications for species interactions, ecosystem processes, and ecological communal structures [1]. A growing number of studies have indicated that phenology is a sensitive indicator of recent global climate change [1], [2], [3], [4], [5], but that phenological responses to climate change are not necessarily shared among community members [3], [4].

Fungi are one of the most diverse kingdom of living organisms that dominate terrestrial landscapes [6] and play key functional roles in ecosystems such as mutualistic symbionts [7], parasites [8], and saprotrophs [9], [10]. For fungi, phenological events include the formation of fruiting bodies and vegetative hyphal growth. Phenological changes in fungal reproduction may result in altered species interactions between fungal competitors, disruption of coevolved mycorrhizal symbiosis, and altered food and habitat availability for mammals [11], arthropods [12], mycoparasitic molds [13], and bacteria [14].

Phenological surveys of fruiting fungi have a very long history worldwide, and some long-term studies have demonstrated that mushroom fruiting was delayed in autumn [15], [16] or hastened in the spring [17]. A 14-year survey of epigenous fruiting bodies in Ireland found that fungal fruiting has a distinct peak around late summer to autumn, possibly triggered by high temperatures [18]. Straatsma et al. [19] found a fungal fruiting peak around the end of September in a Swiss forest based on the 21-year survey of fungal fruiting phenology. Büntgen et al. [20] indicated that intra-annual production of ectomycorrhizal (ECM) fungi increased continuously from late June until early October with a positive correlation with regional cumulative precipitation. Several field studies have argued that both edaphic and climatic factors are responsible for fungal fruiting phenology and enhanced biomass production for which soil water retention is an especially important factor [19], [21], [22], [23], [24]. However, our ability to predict phenological responses of fungi has been limited by a lack of coordinated efforts to combine time-series data with quantitative statistical methods. It is also unclear whether different fungal groups display consistent annual fruiting pattern changes.

ECM and saprotrophs (SAP) differ in methods to obtain carbon sources and other nutrients. While ECM fungi are root symbionts that rely on photosynthate carbon derived from host plants in return for increased access to water, nitrogen, and phosphorus [7], SAP fungi obtain their carbon directly through decomposing organic matter such as leaf litter and coarse woody debris [9], [10]. One may suspect that ECM and SAP fungi differ in fruiting phenology because carbohydrate availability would be more seasonal for ECM fungi than for SAP fungi. In fact, a controlled laboratory study demonstrated that fruiting body production of an ECM fungus, *Laccaria bicolor*, increased with the rate of net photosynthesis of the host plant [25]. This is also consistent with results from large-scale tree-girdling experiments in nurseries that experimentally terminated photosynthate supply to roots and associated ECM fungi, showing that ECM fruiting body biomass increased with host photosynthesis rates and seasonal photosynthate allocation to roots [26], [27]. Owing to the pulsed and seasonal photosynthate flux from host plants, seasonal changes in available carbohydrates are predictable for ECM fungi. However, for SAP fungi, the decomposing substrate supply (e.g., coarse woody debris and dead tree trunks) is often aseasonal, intermittent or long-lasting, and further complicated by time-lagged acquisition of carbohydrates from decomposing substrates due to a complicated community-level process involved with wood and leaf litter decomposition [9], [10].

Here, we report our results from a field survey of mushroom-forming fungi during monthly intervals from 1982 to 2011 in an evergreen oak (*Castanopsis*)-dominant forest in Japan. In particular, we compared the phenology and mushroom productivity between three different functional groups of fungi: ECM fungi, leaf decomposing fungi, and wood-decomposing fungi. We hypothesized that the fruiting phenology of ECM fungi is more seasonal than that of SAP fungi owing to the pulsed and intermittent photosynthate flux from host plants to ECM fungi. The aim of this study is to show what climatic factors are correlated with mushroom productivity of each functional group, thereby testing the above mentioned hypothesis.

## Materials and Methods

### Study site

The study site was located in the Higashiyama hills of the eastern part of Kyoto city in Japan (35.00°N, 135.78°E) at 100–240 m above sea level (Figure S1). The forested study site covers an area of ca. 116 ha, and is a mixed forest of *Castanopsis cuspidata*, *Quercus serrata*, *Quercus glauca*, *Ilex micrococca*, *Ilex pedunculosa*, *Lyonia ovalifolia* ssp. *neziki*, *Rhododendron macrosepalum*, *Eurya japonica*, *Pinus densiflora*, and *Chamaecyparis obtusa*. The forest was dominated by *C. cuspidata*, a shade-tolerant evergreen oak, which has been increasing in number and expanding its range since the 1960s (ca. 0.7 ha increase per year), whereas the incidence of Japanese red pine (*P. densiflora*) has remarkably decreased because of serious damage by pine wilt disease since the 1970s. The climate in this region is humid with a mean annual rainfall of 1480 mm and an average annual temperature of 15.96°C. Temperatures range from 4.6°C (minimum January mean) to 28.8°C (maximum August mean).

### Fruitbody sampling

Our data were sourced from a long-term fixed-point recording of mushroom-forming fungi conducted by amateur mycologists in Kyoto (members of Youkin Club: http://youkin.kinoko.ne.jp/). Monthly surveys were carried out at the study site from May 1982 to March 2011. The mycologists walked in the daytime along a linear census route (ca. 2 km) and searched for all epigeous fungal fruiting bodies in an area of 2 m on both sides of the line, including those on the ground, on coarse woody debris, on fallen trees, and on the trunks of living trees. Collected fruiting bodies were primarily identified in the field unless microscopic inspection (1000×) was required. We classified each fungal species as ECM or SAP fungi (i.e., litter-decomposing fungi or wood-decomposing fungi) following to Tedersoo et al. [28] and Imazeki and Hongo [29], [30].

The present survey used the number of fruiting species as a surrogate for fruiting body phenology and production. We believe that the survey results represent seasonal changes in fungal biomass because a greater encounter probability of productive species results in part from higher diversity [31]. Specimen identification was based on morphological characteristics using several key references; unfortunately, fungal materials in our surveys were not preserved, and thus we may have overlooked cryptic species that could have been distinguished by DNA sequencing. Nevertheless, sampling and identification methods were consistent across seasons.

We state that no specific permits were required for the described field studies, the location was not privately-owned or protected in any way and the field studies did not involve endangered or protected species

### Statistical analysis

We analyzed data from a total of 274 censuses with several missing data. Hypogenous species were excluded from analysis because the participants' sampling expertise could have caused sampling bias.

Nonparametric Steel-Dwass multiple comparisons were used to verify monthly changes in the number of ECM and SAP fruiting fungal species. Kendall's coefficient of concordance (W) was used to assess the monthly change of species richness of fruiting fungi among the following most frequently found genera of ECM fungi *Amanita* (Amanitaceae), *Boletus* (Boletaceae), *Tylopilus* (Boletaceae), *Cortinarius* (Cortinariaceae), *Inocybe* (Inocybaceae), *Lactarius* (Russulaceae), and *Russula* (Russulaceae); of litter-decomposing fungi *Agaricus* (Agaricaceae), *Lepiot*a (Agaricaceae), *Lycoperdon* (Agaricaceae), *Entoloma* (Entolomataceae), *Hygrocybe* (Hygrophoraceae), *Marasmius* (Marasmiaceae), and *Collybia* (Tricholomataceae); and of wood-decomposing fungi *Crepidotus* (Inocybaceae), *Mycena* (Mycenaceae), *Pluteus* (Pluteaceae), *Psathyrella* (Psathyrellaceae), *Pholiota* (Strophariaceae), *Tremella* (Dacrymycetaceae), and *Polyporus* (Polyporaceae). All statistical analyses except for Bayesian modeling were performed using R statistical software version 2.14.1 (http://www.r-project.org/).

We modeled the total number of fruiting species of each trophic group detected in each survey as a function of external factors such as climatic factors and time elapsed since the first observation. To further detect changes in phenological responses, we also fitted the model to the seven ECM genera, the seven litter-decomposing genera, and the seven wood-decomposing genera separately (genera selection was the same for concordance analysis). The observed data were fitted to a zero-inflated binomial (ZIB) model [32], [33] to account for a large proportion of zero values that usually violate the distributional assumptions (i.e., over-dispersion). The first part, *p*_i,j_, models the probability that a fruiting body of an arbitrary species in fungal group *j* (ECM fungi, litter-decomposing fungi, wood-decomposing fungi, and each fungal genus) is truly present in the *i* ^th^ survey. This probability is assumed to be equal for all species in the same fungal group, but the presence of fruiting bodies in individual species is independent of that of other species. The second part, *d*_i,j_, models the probability that a fruiting body of an arbitrary fungal species in fungal group *j* is detected in the *i* ^th^ survey, if present. Failure to detect a fruiting body in a given survey occurs either when the species is actually absent or when it is present but not found in the survey. Therefore, the ZIB model is described as follows:
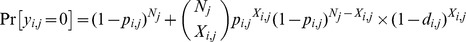


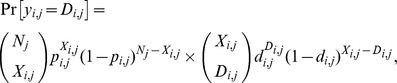
where *N*_j_ is the total number of fungal species in trophic group *j* that were observed throughout the survey years, *X*_i,j_ is the number of species that are actually present in *i* ^th^ survey and trophic group *j*, *D*_i,j_ is the number of species detected in *i* ^th^ survey and trophic group *j*, and *p*_i,j_ and *d*_i,j_ are defined as above.

The model can be extended to allow explanatory variables to influence *p*_i,j_. Therefore, we describe *p*_ij_ as follows:
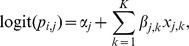
where “logit” is the logistic transformation, *x*_i,k_ is the value of the *k* ^th^ explanatory variable in the *i* ^th^ survey, *α*_j_ is an intercept, and *β*_j,k_ is the estimated regression coefficient for each explanatory variable, which included monthly average temperature, monthly accumulated amount of rainfall, weekly accumulated amount of rainfall, and number of years elapsed since the first observation. Climatic data were sourced from weather station data supplied by the Japan Meteorological Agency (www.jma.go.jp/jma/indexe.html; see Table S1). Daily climate data were obtained from an automated station at Kyoto city (35.00°N, 135.73°E), the closest station to the site. We used a Bayesian framework particularly useful for fitting hierarchical statistical models that include random effects and determining the uncertainty in parameter estimation [34]. We used conventional vague uninformative priors to *α*_j_ and *β*_j,k_ so that they were distributed normally (mean = 0, variance = 1,000,000). Choosing a very large value for the variance resulted in a vague or uninformative prior distribution. In addition, the detection rate of fruiting bodies on each survey date was predicted using an uninformative prior. We sampled posterior densities using MCMC (Markov chain Monte Carlo methods) and the Gibbs sampler in OpenBUGS software ver.3.12 (available from http://www.openbugs.info/w/). We did this with 400,000 interactions using the over relax option and “thinned” every 40th realization (resulting in 10,000 realizations) to reduce autocorrelations. The first 100,000 interactions were discarded as a burn-in period to guarantee convergence to the target posterior distribution. We used three chains with different initial values and thereafter evaluated the Markov-chain Monte Carlo convergence on the posterior distribution using Gelman-Rubin statistic diagnostics [35]. The code of OpenBUGS is shown in Text S1.

## Results

**Figure 1:**
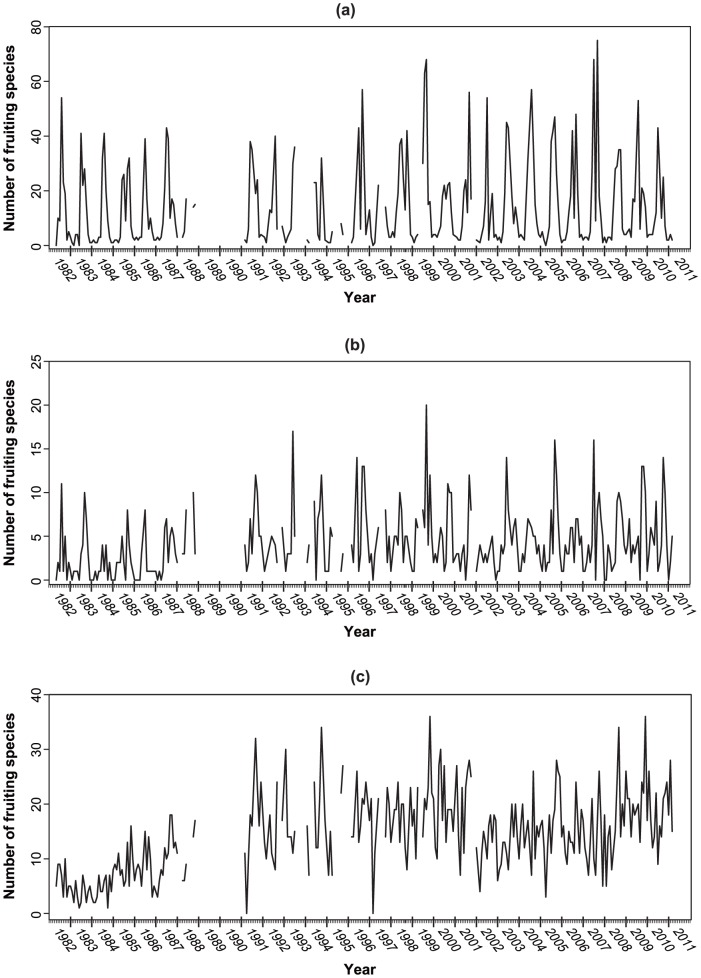
Counts of mushroom fruiting species throughout the survey years for ectomycorrhizal fungi (a), litter-decomposing fungi (b), and wood-decomposing fungi (c).

Within 11,923 total records, 668 epigeous fungal species (9,078 cumulative number of records) were identified to the species level in the study site (Table S2), including 271 ECM species (3,761 records), 137 litter-decomposing fungal species (1,138 records), 236 wood-decomposing fungal species (3,880 records) and 24 species (299 records) that were not assigned into these functional groups (e.g., parasites on arthropods and saprotrophs of pinecone or plant seed). ECM fungi continuously showed clear intra-annual fruiting changes throughout the survey years, but showed an unclear inter-annual fruiting change (Fig. 1a). Litter-decomposing fungi showed a moderate intra-annual change and showed an obscure inter-annual fruiting change (Fig. 1b). The number of fruiting species in wood-decomposing fungi showed indistinct intra-annual changes and a gradual time-dependent increase (Fig. 1c).

**Figure 2:**
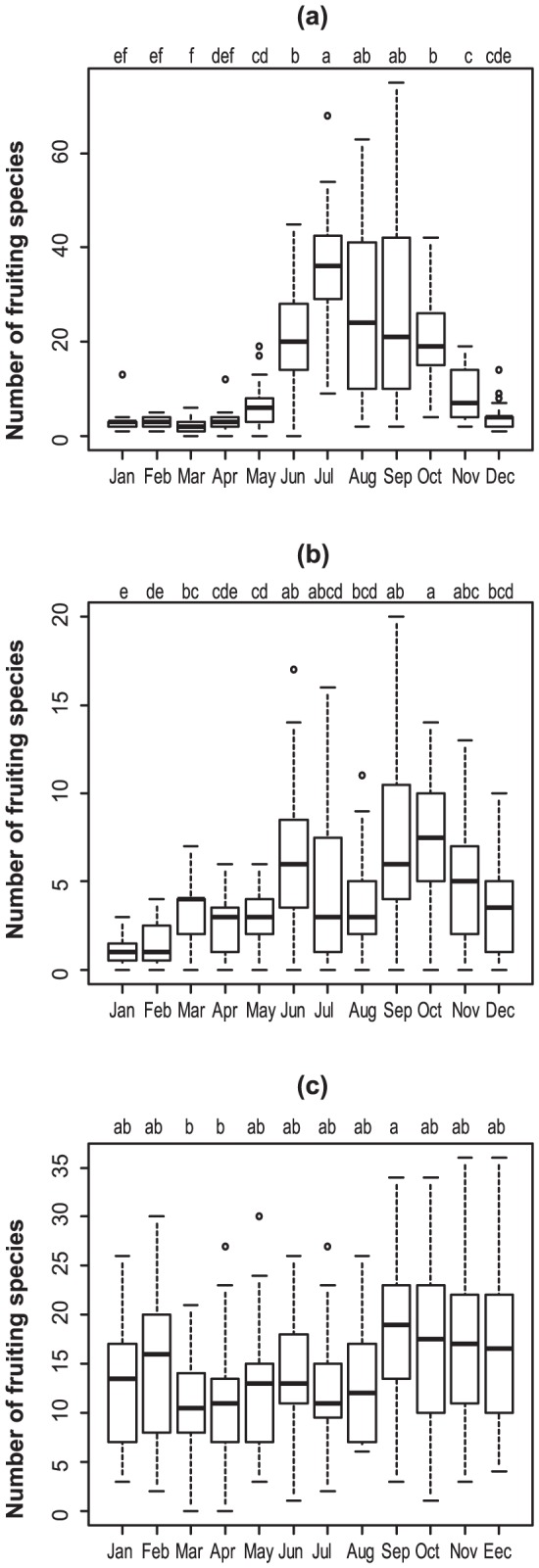
Monthly change in number of fruiting species for ectomycorrhizal fungi (a), litter-decomposing (b), and wood-decomposing fungi (c). Different letters indicate a significant difference at *P*<0.05 (Steel–Dwass test).

**Figure 3:**
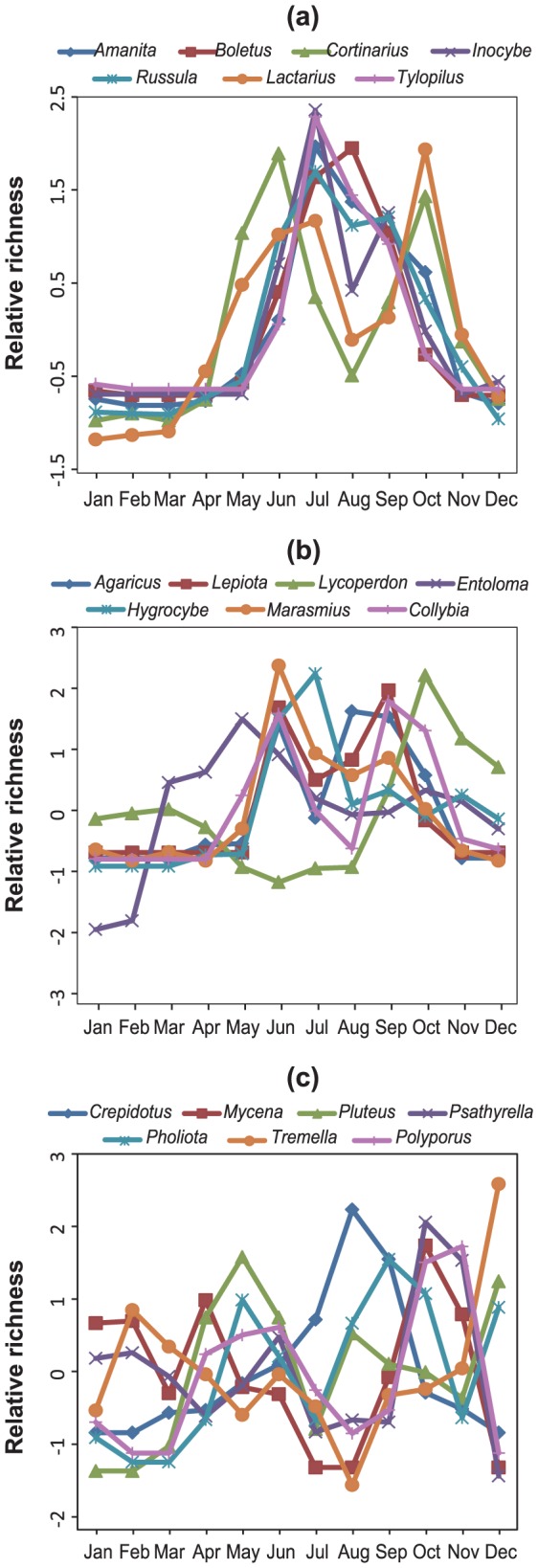
Intergeneric pattern of monthly change in number of fruiting species for ectomycorrhizal fungi (a), litter-decomposing fungi (b), and wood-decomposing fungi (c). Relative richness represents the standardized monthly mean number of fruiting species in each fungal genus.

We found a striking difference in the fruiting phenology among fungal functional groups. First, ECM fungi showed a clear phenology in the number of fruiting species throughout the survey with the highest number in July and the lowest in March (Fig. 2a). The likely unimodal fruiting phenology was consistent among the seven genera observed (Kendall's W = 0.773, P = 2.93E10; Fig. 3a). The number of litter-decomposing fungi showed a moderate seasonal change with the highest in early autumn and the lowest in winter (Fig. 2b); this trend was not clearly consistent among the seven genera of litter-decomposers, unlike ECM fungi (Kendall's W = 0.436, P = 4.22E–4; Fig. 3b). Wood-decomposing fungi showed a much less clear seasonal peak in the observed number of species than litter-decomposing fungi (Fig. 2c). The seasonal patterns of wood-decomposing fungi were substantially variable among the seven genera (Kendall's W = 0.219, P = 0.113; Fig. 3c), and were almost taxon-specific. This trend held when fungal species that form long-lived fruiting bodies (i.e., aphyllophoraceous fungi) were removed from the analysis (Figure S2).

**Figure 4:**
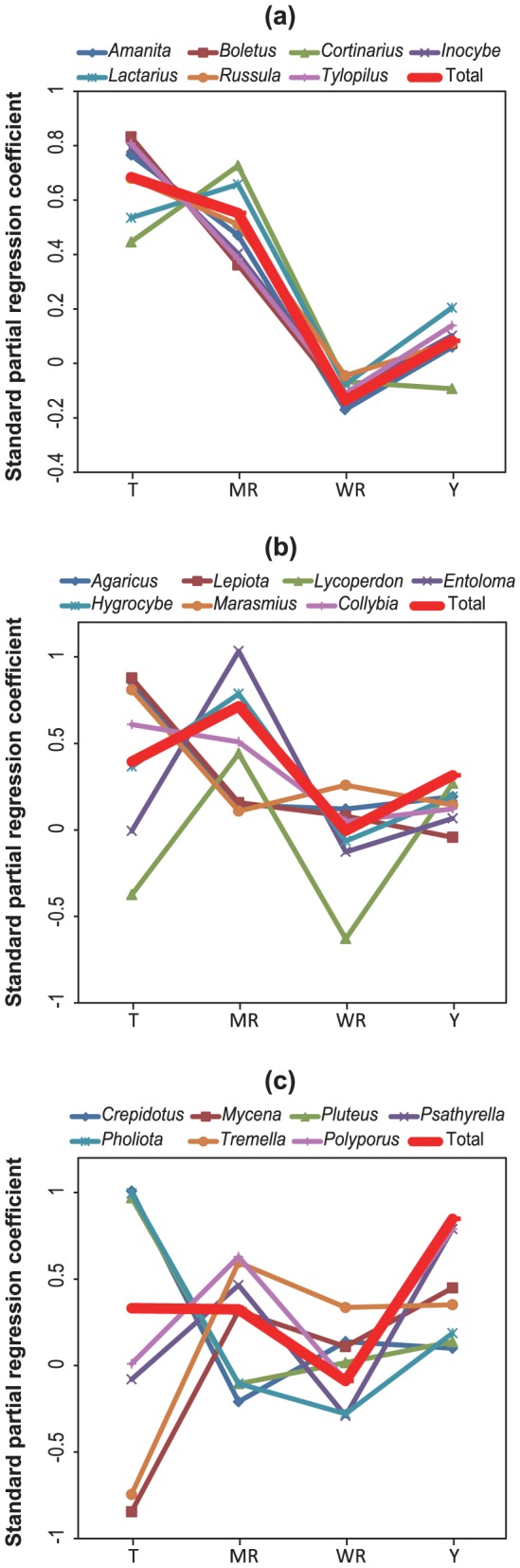
Posterior mean of standard partial regression coefficients when the number of fruiting species in ectomycorrhizal (ECM) (a), litter-decomposing (b), and wood-decomposing fungal genera was regressed against monthly temperature (T), monthly accumulated rainfall (MR), weekly accumulated rainfall (WR), and number of elapsed years (Y). A positive regression coefficient value indicates positive effects on the number of fruiting species, whereas a negative value indicates the reverse. Details concerning posterior distribution of regression coefficients are shown in Figure S3.

The ZIB modeling supported differential phenological fruiting responses between ECM and SAP fungi. Notably, key fruiting determinants were very similar among the examined ECM fungal genera (Fig. 4a). Fruiting ECM species increased more frequently with higher temperatures and increased monthly accumulated rainfall (Fig. 4a; see details in Figure S3). Weekly accumulated rainfall before the surveys slightly decreased the number of fruiting species, although the number of elapsed years somewhat increased the number of fruiting ECM fungi. Key factors increasing the number of fruiting species were somewhat similar within litter-decomposing fungal genera, but were much more divergent than that of ECM fungi (Fig. 4b; see details in Figure S3). The number of *Lepiota* and *Marasmius* fruiting species were affected by temperature, although those in *Entoloma* and *Hygrocybe* were primarily influenced by monthly rainfall. In wood-decomposing fungi, each wood-decomposing fungal genus was influenced by a different set of factors (Fig. 4c; see details in Figure S3). Monthly temperature had a positive influence on fruiting *Crepidotus*, *Pluteus*, and *Pholiota*, but had a negative influence on *Mycena* and *Tremella*. Monthly accumulated rainfall had a positive influence on the number of *Polyporus* fruiting bodies and the number of elapsed years had a positive effect on *Polyporus* and *Psathyrella*.

## Discussion

We found that seasonal fungal fruiting patterns were remarkably different depending on the fungal trophic group (Fig. 2, 3). ECM fungal taxa virtually had clear unimodal fruiting patterns from middle summer to early autumn, although the seasonal patterns among wood-decomposing fungal taxa varied greatly. Moreover, we showed that unclear seasonal peak fruiting of wood-decomposing fungi may not be attributed to the perennial nature of aphyllophoraceous fungi. Although there is, to a greater or lesser extent, an inter-annual fruiting phenology difference at the same calendar dates in each trophic fungal group (Fig. 2), these trends may be chiefly reflected by the inter-annual difference in rainfall to ensure high soil water retention [19], [21], [24]. Nevertheless, we found a time-dependent fruiting increase in wood-decomposing fungi, possibly due to the progressive effects of forest aging that can increase the abundance and variety of coarse woody debris [36].

These results provide strong support for our hypothesis that phenological ECM fungal patterns are more seasonally predictable than those of SAP fungi, owing to the predictable seasonal patterns in the preceding assimilate fluxes from host plants to ECM fungi. The unimodal patterns of fruiting phenology in ECM fungi are in accord with the recent work by Büntgen et al. [20], although they showed peak ECM fungal fruiting in autumn rather than summer. The different timing of the fruiting peak may be attributed to regional timing difference in which host plants of ECM fungi show the maximum rates of net photosynthesis. A clearer seasonality of litter-decomposing fungi compared to wood-decomposing fungi may reflect greater predictability of seasonal availability of decomposing substrates. The peak leaf fall of deciduous trees occurs in late autumn in a temperate forest, whereas that of evergreen trees is usually in late spring [37], [38]. However, in wood-decomposing fungi, the availability of decomposing substrates is almost aseasonal. Nevertheless, seasonal patterns in availability of litter-derived carbon seems less predictable than the photosynthate fluxes from host plants of ECM fungi because there is an unpredictable time lag between litter supply and carbohydrate acquisition from decomposing substrates [9]. This may account for less distinct seasonal fruiting patterns in litter-decomposing fungi than in ECM fungi.

While many animal species synchronize reproductive activities and life history events with the timing of food supply [39], [40], [41], [42], ECM fungi may also adjust their reproduction schedule to host photosynthetic activities [26], [27]. In fact, the unimodal pattern of ECM fruiting phenology is somewhat in accordance with the seasonal change of net photosynthetic rates in *Castanopsis* [43], the most dominant tree in the study site throughout the survey years. We therefore suspect that a large amount of assimilates from host plants can exceed the storage capacity of mycelia in ECM fungi, which can then route the excess to fruiting bodies during the host plant growing season. Moreover, our model showed that high temperature and sufficient accumulated rainfall are key determinants of fruiting of all ECM fungal taxa, but not necessarily for SAP fungi (Fig. 4), which implies that these climatic factors may be responsible for increased net photosynthesis rates in host plants of ECM fungi. Indeed, this trend is in agreement with a temperature-dependent increase in photosynthesis rates [42], [44]. Furthermore, rich rainfall may produce high soil water availability that can minimize reduction of photosynthesis in host plants during droughts [45].

Large photosynthate amounts are allocated through the tree-root interface to mycorrhizal fungal mycelia, thereby substantially contributing to the organic soil fraction in a forest and affecting CO_2_ exchange rates with the atmosphere [46]. Therefore, photosynthate fluxes to mycorrhizal symbionts are important drivers of biological processes in soil as well as the much slower carbon fluxes arising from trunk, shoot, and root-derived litter decomposition. Fungal fruiting bodies are an important food source for soil fauna and microbes [12], [47], and thus substantial carbon resources may have a far-reaching impact on soil organisms via fungus feeding. Indeed, highly seasonal ECM fungal fruiting ensures readily supplied food sources for mycophagous and mycoparasitic organisms of higher trophic levels, which may influence their survival and life history events. We argue that fruiting phenology of each functional fungal guild makes a differential contribution to the community structure of mycophagous organisms, terrestrial biomass turnover, and global carbon cycle.

## Supporting Information

Figure S1 **Locality and aerial photography of the study site.** The white arrow represents the trail followed during the surveys.
(EPS)

Figure S2 **Monthly change in the number of fruiting species for wood-decomposing fungi that form ephemeral fruiting bodies (a) and long-lived fruiting bodies (b).** Different letters indicate results that differ significantly at P<0.05 (SteelDwass test).
(EPS)

Figure S3 **Density plot for posterior distribution of standard partial regression coefficients when the number of fruiting species in ectomycorrhizal (ECM) fungi, litter-decomposing fungi, and wood-decomposing fungi was regressed against monthly temperature (T), monthly accumulated rainfall (MR), weekly accumulated rainfall (WR), and elapsed years (Y).** A positive regression coefficient value indicates that the corresponding variable is positively correlated with the number of fruiting species, whereas a negative value indicates the reverse. Single asterisks indicate that 95% credible intervals do not include zero, and double asterisks indicate 99% credible intervals.
(EPS)

Table S1 **Climatic information of each collection date.**
(DOC)

Table S2 **A list of observed species at each survey.** The nutritional guilds, including ectomycorrhizal fungi (ECM), litter-decomposing fungi (L), and wood-decomposing fungi (W) were identified for fungal species in which species-level identification could be performed. NA represents the nutritional guild of the fungus is not classified into these three guilds.
(DOC)

Text S1 **BUGS code used in this study.**
(DOC)
