# Supplementary material for: A Thirty-Year Survey Reveals That Ecosystem Function of Fungi Predicts Phenology of Mushroom Fruiting
Source: PLoS One. 2012 Nov 27;7(11):e49777. doi: 10.1371/journal.pone.0049777 (PMC3507881; doi:10.1371/journal.pone.0049777)
Supplement: Table S1 — Climatic information of each collection date. (DOC) [file pone.0049777.s004.doc]

**Table S1. Climatic information of each collection date.**

| **Collection date** | **Monthly accumulated rainfall (mm)** | **Weekly accumulated rainfall (mm)** | **Monthly average temperature (˚C)** |
| --- | --- | --- | --- |
| May 5, 1982 | 94.0 | 1.0 | 19.11 |
| Jun 6, 1982 | 133.0 | 11.5 | 22.02 |
| Jul. 7, 1982 | 64.0 | 30.5 | 22.49 |
| Aug. 8, 1982 | 402.0 | 153.5 | 25.68 |
| Sep. 9, 1982 | 188.5 | 1.5 | 26.54 |
| Oct. 10, 1982 | 89.5 | 3.0 | 21.57 |
| Nov. 11, 1982 | 22.5 | 5.5 | 16.86 |
| Dec. 12, 1982 | 112.0 | 51.5 | 12.82 |
| Jan. 1, 1983 | 50.0 | 0.0 | 5.73 |
| Feb. 2, 1983 | 29.0 | 7.0 | 4.47 |
| Mar. 3, 1983 | 125.5 | 55.0 | 5.47 |
| Apr. 4, 1983 | 152.0 | 93.0 | 12.20 |
| May 5, 1983 | 182.0 | 98.0 | 18.65 |
| Jun 6, 1983 | 100.5 | 0.0 | 20.76 |
| Jul. 7, 1983 | 330.5 | 55.0 | 23.12 |
| Aug. 8, 1983 | 135.5 | 55.5 | 28.59 |
| Sep. 9, 1983 | 163.5 | 60.5 | 27.55 |
| Oct. 10, 1983 | 137.5 | 19.5 | 16.96 |
| Nov. 11, 1983 | 89.0 | 1.5 | 13.97 |
| Dec. 12, 1983 | 30.5 | 1.0 | 8.92 |
| Jan. 1, 1984 | 26.0 | 3.5 | 2.67 |
| Feb. 2, 1984 | 77.5 | 22.5 | 1.99 |
| Mar. 3, 1984 | 78.0 | 29.5 | 3.96 |
| Apr. 4, 1984 | 74.5 | 42.0 | 10.86 |
| May 5, 1984 | 96.0 | 1.0 | 17.74 |
| Jun 6, 1984 | 117.5 | 75.5 | 20.44 |
| Jul. 7, 1984 | 292.5 | 0.0 | 24.50 |
| Aug. 8, 1984 | 194.0 | 69.5 | 27.06 |
| Sep. 9, 1984 | 109.0 | 38.0 | 27.85 |
| Oct. 10, 1984 | 120.5 | 16.0 | 21.65 |
| Nov. 11, 1984 | 44.5 | 32.0 | 14.09 |
| Dec. 12, 1984 | 41.0 | 4.5 | 10.63 |
| Jan. 1, 1985 | 25.0 | 0.0 | 4.23 |
| Feb. 2, 1985 | 104.5 | 30.5 | 4.89 |
| Mar. 3, 1985 | 156.0 | 67.0 | 6.84 |
| Apr. 4, 1985 | 187.5 | 24.0 | 12.54 |
| May 5, 1985 | 82.5 | 16.0 | 18.32 |
| Jun 6, 1985 | 338.5 | 238.0 | 21.76 |
| Jul. 7, 1985 | 390.0 | 53.0 | 26.05 |
| Aug. 8, 1985 | 76.0 | 10.5 | 29.02 |
| Sep. 9, 1985 | 190.0 | 92.5 | 24.90 |
| Oct. 10, 1985 | 160.0 | 10.5 | 21.19 |
| Nov. 11, 1985 | 46.0 | 1.0 | 14.29 |
| Dec. 12, 1985 | 56.5 | 6.0 | 6.32 |
| Jan. 1, 1986 | 38.5 | 0.0 | 3.59 |
| Feb. 2, 1986 | 0.0 | 0.0 | 2.92 |
| Mar. 3, 1986 | 31.0 | 0.0 | 3.19 |
| Apr. 4, 1986 | 148.0 | 23.5 | 9.54 |
| May 5, 1986 | 208.5 | 54.5 | 16.12 |
| Jul. 7, 1986 | 386.5 | 45.5 | 23.61 |
| Aug. 8, 1986 | 221.0 | 0.0 | 27.77 |
| Sep. 9, 1986 | 30.0 | 8.0 | 27.40 |
| Oct. 10, 1986 | 85.5 | 30.0 | 22.84 |
| Nov. 11, 1986 | 47.0 | 3.0 | 13.98 |
| Dec. 12, 1986 | 17.0 | 1.0 | 10.17 |
| Jan. 1, 1987 | 81.5 | 24.5 | 5.13 |
| Feb. 2, 1987 | 65.5 | 18.5 | 5.44 |
| Mar. 3, 1987 | 103.5 | 20.5 | 7.72 |
| Apr. 4, 1987 | 36.5 | 7.0 | 12.87 |
| May 5, 1987 | 163.0 | 1.5 | 18.70 |
| Jun 6, 1987 | 180.0 | 91.0 | 22.34 |
| Jul. 7, 1987 | 164.5 | 16.0 | 23.88 |
| Aug. 8, 1987 | 167.5 | 10.5 | 27.82 |
| Sep. 9, 1987 | 63.5 | 1.0 | 26.25 |
| Oct. 10, 1987 | 97.0 | 1.0 | 21.64 |
| Nov. 11, 1987 | 122.0 | 3.0 | 15.24 |
| Dec. 12, 1987 | 35.5 | 7.5 | 10.26 |
| Jan. 1, 1988 | 36.0 | 9.0 | 6.97 |
| Apr. 4, 1988 | 109.5 | 28.0 | 10.12 |
| May 5, 1988 | 136.5 | 17.5 | 17.22 |
| Jun 6, 1988 | 298.5 | 10.0 | 20.95 |
| Oct. 10, 1988 | 119.0 | 8.5 | 18.84 |
| Nov. 11, 1988 | 43.0 | 0.0 | 13.35 |
| Jul. 7, 1989 | 259.0 | 105.5 | 23.24 |
| Jul. 7, 1990 | 223.0 | 14.5 | 26.39 |
| Oct. 10, 1990 | 171.0 | 24.5 | 18.77 |
| Mar. 3, 1991 | 177.0 | 28.5 | 9.42 |
| Apr. 4, 1991 | 206.0 | 15.5 | 13.65 |
| May 5, 1991 | 81.0 | 0.0 | 18.05 |
| Jun 6, 1991 | 288.0 | 27.0 | 23.73 |
| Jul. 7, 1991 | 311.0 | 174.0 | 26.04 |
| Sep. 9, 1991 | 92.0 | 5.5 | 25.31 |
| Oct. 10, 1991 | 249.0 | 32.5 | 18.64 |
| Nov. 11, 1991 | 53.5 | 0.0 | 13.84 |
| Dec. 12, 1991 | 84.0 | 0.0 | 11.26 |
| Feb. 2, 1992 | 44.0 | 0.5 | 5.26 |
| Mar. 3, 1992 | 138.0 | 16.0 | 9.50 |
| May 5, 1992 | 168.0 | 22.5 | 17.05 |
| Jun 6, 1992 | 149.5 | 75.0 | 21.53 |
| Aug. 8, 1992 | 165.5 | 8.5 | 27.78 |
| Sep. 9, 1992 | 32.5 | 22.0 | 25.02 |
| Dec. 12, 1992 | 74.5 | 12.0 | 11.36 |
| Feb. 2, 1993 | 84.0 | 39.5 | 5.87 |
| Mar. 3, 1993 | 63.0 | 20.0 | 7.30 |
| May 5, 1993 | 86.0 | 0.5 | 18.03 |
| Jun 6, 1993 | 256.5 | 77.0 | 21.86 |
| Jul. 7, 1993 | 596.0 | 35.0 | 23.18 |
| Nov. 11, 1993 | 130.5 | 3.5 | 15.04 |
| Feb. 2, 1994 | 89.0 | 29.0 | 4.38 |
| Mar. 3, 1994 | 32.5 | 5.0 | 6.43 |
| Jun 6, 1994 | 101.0 | 18.0 | 22.54 |
| Jul. 7, 1994 | 82.0 | 6.0 | 29.33 |
| Aug. 8, 1994 | 39.5 | 19.5 | 29.81 |
| Sep. 9, 1994 | 99.0 | 10.5 | 26.53 |
| Oct. 10, 1994 | 141.0 | 14.5 | 23.05 |
| Dec. 12, 1994 | 23.0 | 0.0 | 12.74 |
| Feb. 2, 1995 | 19.0 | 0.0 | 4.32 |
| Mar. 3, 1995 | 44.5 | 1.0 | 7.96 |
| Apr. 4, 1995 | 89.5 | 11.5 | 11.96 |
| Sep. 9, 1995 | 82.0 | 30.0 | 26.84 |
| Oct. 10, 1995 | 50.5 | 0.0 | 20.37 |
| Mar. 3, 1996 | 136.5 | 45.5 | 6.15 |
| Apr. 4, 1996 | 111.0 | 35.0 | 9.74 |
| Jun 6, 1996 | 153.0 | 92.0 | 22.68 |
| Jul. 7, 1996 | 217.5 | 26.5 | 25.55 |
| Aug. 8, 1996 | 153.0 | 106.0 | 29.23 |
| Sep. 9, 1996 | 185.5 | 11.5 | 22.89 |
| Oct. 10, 1996 | 140.0 | 44.0 | 18.72 |
| Nov. 11, 1996 | 72.5 | 20.0 | 15.32 |
| Jul. 7, 1997 | 33.0 | 0.0 | 26.54 |
| Feb. 2, 1997 | 44.0 | 14.0 | 3.91 |
| Mar. 3, 1997 | 61.5 | 21.5 | 8.32 |
| Apr. 4, 1997 | 126.5 | 0.0 | 12.24 |
| Jun 6, 1997 | 56.5 | 18.0 | 21.21 |
| Oct. 10, 1997 | 168.5 | 3.0 | 20.67 |
| Nov. 11, 1997 | 58.0 | 39.0 | 13.10 |
| Dec. 12, 1997 | 160.5 | 19.5 | 9.97 |
| Jan. 1, 1998 | 149.0 | 20.5 | 5.76 |
| Feb. 2, 1998 | 39.0 | 32.0 | 5.45 |
| Mar. 3, 1998 | 126.0 | 34.5 | 8.81 |
| Apr. 4, 1998 | 178.0 | 9.5 | 16.42 |
| May 5, 1998 | 232.5 | 65.0 | 21.17 |
| Jun 6, 1998 | 232.0 | 87.0 | 21.97 |
| Jul. 7, 1998 | 262.0 | 37.5 | 25.76 |
| Aug. 8, 1998 | 90.0 | 47.5 | 29.01 |
| Sep. 9, 1998 | 57.5 | 9.0 | 26.49 |
| Oct. 10, 1998 | 255.5 | 34.5 | 23.96 |
| Dec. 12, 1998 | 32.5 | 1.0 | 9.15 |
| Jan. 1, 1999 | 34.5 | 14.0 | 5.13 |
| Feb. 2, 1999 | 57.5 | 23.5 | 4.58 |
| Mar. 3, 1999 | 153.0 | 23.5 | 9.84 |
| Apr. 4, 1999 | 78.5 | 26.0 | 13.08 |
| Jul. 7, 1999 | 506.5 | 0.0 | 23.56 |
| Aug. 8, 1999 | 99.5 | 7.5 | 28.33 |
| Sep. 9, 1999 | 154.0 | 45.5 | 27.19 |
| Oct. 10, 1999 | 100.5 | 31.0 | 19.03 |
| Nov. 11, 1999 | 94.0 | 12.5 | 14.50 |
| Dec. 12, 1999 | 19.0 | 0.0 | 9.28 |
| Jan. 1, 2000 | 66.5 | 18.0 | 6.16 |
| Feb. 2, 2000 | 39.5 | 1.5 | 3.74 |
| Mar. 3, 2000 | 62.5 | 18.5 | 5.05 |
| Apr. 4, 2000 | 87.0 | 30.0 | 12.14 |
| May 5, 2000 | 123.5 | 44.5 | 17.63 |
| Jun 6, 2000 | 135.5 | 37.0 | 21.68 |
| Jul. 7, 2000 | 49.0 | 43.5 | 28.19 |
| Aug. 8, 2000 | 76.5 | 0.0 | 28.95 |
| Sep. 9, 2000 | 283.0 | 247.5 | 27.71 |
| Oct. 10, 2000 | 158.5 | 59.5 | 19.04 |
| Nov. 11, 2000 | 194.5 | 17.0 | 13.97 |
| Dec. 12, 2000 | 40.0 | 2.0 | 9.27 |
| Feb. 2, 2001 | 85.5 | 24.0 | 5.24 |
| Mar. 3, 2001 | 108.5 | 3.5 | 7.88 |
| Apr. 4, 2001 | 27.5 | 11.0 | 13.91 |
| May 5, 2001 | 127.5 | 75.0 | 19.39 |
| Jun 6, 2001 | 186.0 | 57.5 | 21.77 |
| Jul. 7, 2001 | 110.0 | 18.0 | 28.57 |
| Aug. 8, 2001 | 87.5 | 83.5 | 28.96 |
| Sep. 9, 2001 | 217.5 | 53.5 | 25.82 |
| Oct. 10, 2001 | 172.5 | 19.0 | 18.59 |
| Jan. 1, 2002 | 60.0 | 34.5 | 5.87 |
| Mar. 3, 2002 | 73.5 | 1.0 | 9.79 |
| Apr. 4, 2002 | 145.5 | 70.5 | 13.63 |
| May 5, 2002 | 114.0 | 3.5 | 18.51 |
| Jun 6, 2002 | 57.0 | 13.5 | 23.03 |
| Jul. 7, 2002 | 172.5 | 0.0 | 27.33 |
| Aug. 8, 2002 | 66.5 | 39.0 | 28.20 |
| Sep. 9, 2002 | 72.0 | 1.5 | 26.71 |
| Oct. 10, 2002 | 91.5 | 5.5 | 20.51 |
| Nov. 11, 2002 | 31.0 | 0.0 | 10.38 |
| Dec. 12, 2002 | 13.5 | 1.5 | 8.47 |
| Jan. 1, 2003 | 56.5 | 25.0 | 3.99 |
| Feb. 2, 2003 | 105.0 | 44.0 | 4.86 |
| Mar. 3, 2003 | 119.0 | 12.5 | 5.87 |
| Apr. 4, 2003 | 168.5 | 81.0 | 14.13 |
| May 5, 2003 | 123.0 | 13.5 | 18.85 |
| Jun 6, 2003 | 296.5 | 149.0 | 22.55 |
| Jul. 7, 2003 | 377.5 | 72.0 | 23.72 |
| Sep. 9, 2003 | 141.5 | 110.5 | 25.83 |
| Oct. 10, 2003 | 52.5 | 28.0 | 17.66 |
| Nov. 11, 2003 | 86.5 | 16.5 | 15.15 |
| Dec. 12, 2003 | 140.5 | 18.5 | 11.37 |
| Jan. 1, 2004 | 27.0 | 10.0 | 4.69 |
| Feb. 2, 2004 | 42.0 | 36.0 | 6.27 |
| Mar. 3, 2004 | 73.0 | 20.5 | 8.31 |
| Apr. 4, 2004 | 106.0 | 51.5 | 14.87 |
| Jun 6, 2004 | 166.5 | 81.0 | 24.02 |
| Aug. 8, 2004 | 196.0 | 95.5 | 27.72 |
| Sep. 9, 2004 | 157.0 | 38.0 | 26.16 |
| Oct. 10, 2004 | 359.5 | 160.0 | 20.04 |
| Nov. 11, 2004 | 92.0 | 0.0 | 14.15 |
| Dec. 12, 2004 | 82.0 | 1.5 | 9.74 |
| Jan. 1, 2005 | 60.5 | 15.0 | 4.52 |
| Feb. 2, 2005 | 52.0 | 34.5 | 4.96 |
| Mar. 3, 2005 | 74.5 | 24.5 | 6.83 |
| Apr. 4, 2005 | 39.5 | 4.0 | 13.01 |
| May 5, 2005 | 94.5 | 7.0 | 18.53 |
| Jun 6, 2005 | 47.0 | 12.0 | 21.67 |
| Jul. 7, 2005 | 214.0 | 119.5 | 25.23 |
| Aug. 8, 2005 | 65.5 | 5.0 | 28.56 |
| Sep. 9, 2005 | 127.0 | 71.0 | 27.31 |
| Oct. 10, 2005 | 75.0 | 0.0 | 20.96 |
| Nov. 11, 2005 | 76.5 | 0.0 | 13.77 |
| Dec. 12, 2005 | 60.0 | 32.5 | 8.79 |
| Jan. 1, 2006 | 36.5 | 1.5 | 3.78 |
| Feb. 2, 2006 | 46.5 | 13.5 | 4.29 |
| Mar. 3, 2006 | 152.0 | 43.5 | 6.97 |
| Apr. 4, 2006 | 139.0 | 21.5 | 10.94 |
| May 5, 2006 | 158.5 | 9.0 | 18.42 |
| Jun 6, 2006 | 172.0 | 77.0 | 21.54 |
| Jul. 7, 2006 | 376.0 | 250.5 | 25.46 |
| Aug. 8, 2006 | 33.0 | 2.5 | 29.39 |
| Sep. 9, 2006 | 185.0 | 1.5 | 25.09 |
| Oct. 10, 2006 | 84.0 | 0.0 | 20.39 |
| Nov. 11, 2006 | 47.5 | 25.5 | 14.00 |
| Dec. 12, 2006 | 95.0 | 24.0 | 11.01 |
| Jan. 1, 2007 | 24.0 | 0.5 | 5.49 |
| Feb. 2, 2007 | 63.5 | 23.0 | 7.08 |
| Mar. 3, 2007 | 59.5 | 15.5 | 7.54 |
| Apr. 4, 2007 | 37.5 | 8.5 | 13.15 |
| May 5, 2007 | 89.0 | 21.0 | 17.50 |
| Jun 6, 2007 | 130.5 | 27.5 | 20.73 |
| Jul. 7, 2007 | 343.5 | 45.5 | 24.72 |
| Aug. 8, 2007 | 70.0 | 61.5 | 29.05 |
| Sep. 9, 2007 | 141.5 | 1.0 | 27.32 |
| Oct. 10, 2007 | 113.5 | 53.5 | 21.18 |
| Nov. 11, 2007 | 46.0 | 1.5 | 13.41 |
| Dec. 12, 2007 | 16.5 | 5.5 | 10.25 |
| Jan. 1, 2008 | 81.0 | 15.5 | 5.13 |
| Feb. 2, 2008 | 44.0 | 3.0 | 3.58 |
| Mar. 3, 2008 | 112.5 | 41.5 | 7.78 |
| Apr. 4, 2008 | 195.5 | 17.5 | 13.21 |
| May 5, 2008 | 92.0 | 40.0 | 17.35 |
| Jul. 7, 2008 | 75.0 | 0.0 | 27.89 |
| Aug. 8, 2008 | 87.5 | 34.5 | 28.04 |
| Sep. 9, 2008 | 182.5 | 149.0 | 24.96 |
| Oct. 10, 2008 | 273.0 | 18.5 | 20.14 |
| Nov. 11, 2008 | 66.5 | 37.0 | 12.09 |
| Dec. 12, 2008 | 71.0 | 2.5 | 9.68 |
| Jan. 1, 2009 | 45.5 | 24.0 | 4.79 |
| Feb. 2, 2009 | 119.5 | 26.5 | 6.41 |
| Mar. 3, 2009 | 174.5 | 4.5 | 8.63 |
| Apr. 4, 2009 | 38.0 | 7.0 | 13.45 |
| May 5, 2009 | 100.0 | 18.5 | 19.36 |
| Jun 6, 2009 | 68.5 | 6.0 | 21.64 |
| Aug. 8, 2009 | 386.0 | 34.0 | 27.40 |
| Sep. 9, 2009 | 33.0 | 0.0 | 23.96 |
| Oct. 10, 2009 | 136.5 | 0.0 | 20.65 |
| Nov. 11, 2009 | 139.0 | 99.5 | 16.15 |
| Dec. 12, 2009 | 92.5 | 23.5 | 10.47 |
| Jan. 1, 2010 | 17.5 | 10.0 | 4.98 |
| Feb. 2, 2010 | 118.5 | 36.5 | 6.63 |
| Mar. 3, 2010 | 222.0 | 70.0 | 9.10 |
| Apr. 4, 2010 | 169.0 | 67.5 | 11.11 |
| May 5, 2010 | 142.5 | 11.5 | 15.02 |
| Jun 6, 2010 | 295.5 | 131.5 | 21.05 |
| Jul. 7, 2010 | 465.0 | 24.5 | 26.95 |
| Aug. 8, 2010 | 175.0 | 0.0 | 29.98 |
| Sep. 9, 2010 | 113.5 | 58.5 | 29.03 |
| Oct. 10, 2010 | 112.0 | 23.5 | 19.22 |
| Nov. 11, 2010 | 64.5 | 10.5 | 12.44 |
| Dec. 12, 2010 | 50.5 | 0.5 | 10.65 |
| Jan. 1, 2011 | 20.0 | 0.0 | 2.88 |
| Feb. 2, 2011 | 45.5 | 0.0 | 5.38 |
| Mar. 3, 2011 | 95.0 | 30.5 | 7.06 |
