# Supplementary material for: A Thirty-Year Survey Reveals That Ecosystem Function of Fungi Predicts Phenology of Mushroom Fruiting
Source: PLoS One. 2012 Nov 27;7(11):e49777. doi: 10.1371/journal.pone.0049777 (PMC3507881; doi:10.1371/journal.pone.0049777)
Supplement: Table S2 — A list of observed species at each survey. The nutritional guilds, including ectomycorrhizal fungi (ECM), litter-decomposing fungi (L), and wood-decomposing fungi (W) were identified for fungal species in which species-level identification could be performed. NA represents the nutritional guild of the fungus is not classified into these three guilds. (DOC) [file pone.0049777.s005.doc]

**Table S2. A list of observed species at each survey.** The nutritional guilds, including ectomycorrhizal fungi (ECM), litter-decomposing fungi (L), and wood-decomposing fungi (W) were identified for fungal species in which species-level identification could be performed. NA represents the nutritional guild of the fungus is not assigned into these guilds.

| **Survey date** | **Observed species (Nutritional guild)** |
| --- | --- |
| 23-May-82 | *Cryptoporus volvatus* (W)*, Cyclomyces fuscus* (W)*, Microporus affinis* (W)*, Neolentinus lepideus* (W)*, Schizophyllum commune* (W) |
| 27-Jun-82 | *Armillaria mellea* (W)*, Astraeus hygrometricus* (ECM)*, Clavaria vermicularis* (L)*, Coltricia cinnamomea* (ECM)*, Coprinus atramentarius* (L)*, Cryptoporus volvatus* (W)*, Ganoderma applanatum* (W)*, Laccaria vinaceoavellanea* (ECM)*, Lactarius gracilis* (ECM)*, Pluteus atricapillus* (W)*, Psathyrella candolliana* (W)*, Psathyrella velutina* (W)*, Rhizopogon superiorensis* (ECM)*, Russula alboareolata* (ECM)*, Russula cyanoxantha* (ECM)*, Russula mariae* (ECM)*, Scleroderma areolatum* (ECM)*, Scutellinia scutellata* (W)*, Suillus bovinus* (ECM)*, Trametes versicolor* (W)*, Tyromyces chioneus* (W) |
| 11-Jul-82 | *Amanita griseofarinosa* (ECM)*, Armillaria mellea* (W)*, Artomyces pyxidatus* (W)*, Astraeus hygrometricus* (ECM)*, Calostoma japonicum* (ECM)*, Collybia dryophila* (L)*, Coprinus disseminatus* (W)*, Cordyceps japonensis (NA), Cryptoporus volvatus* (W)*, Daedaleopsis styracina* (W)*, Ganoderma applanatum* (W)*, Gomphidius roseus* (ECM)*, Hebeloma vinosophyllum* (ECM)*, Laccaria bicolor* (ECM)*, Phylloporus bellus* (ECM)*, Psathyrella candolliana* (W)*, Russula alboareolata* (ECM)*, Russula cyanoxantha* (ECM)*, Schizophyllum commune* (W)*, Xerula pudens* (W) |
| 22-Aug-82 | *Amanita farinosa* (ECM)*, Amanita fuliginea* (ECM)*, Amanita fulva* (ECM)*, Amanita griseofarinosa* (ECM)*, Amanita pseudoporphyria* (ECM)*, Amanita punctata* (ECM)*, Amanita rufoferruginea* (ECM)*, Amanita spissacea* (ECM)*, Amanita sychnopyramis f. subannulata* (ECM)*, Amanita vaginata var. punctata* (ECM)*, Amanita virgineoides* (ECM)*, Amanita virosa* (ECM)*, Amanita volvata* (ECM)*, Astraeus hygrometricus* (ECM)*, Austroboletus fusisporus* (ECM)*, Austroboletus subvirens* (ECM)*, Boletellus emodensis* (ECM)*, Boletellus obscurecoccineus* (ECM)*, Boletellus russellii* (ECM)*, Boletus fraternus* (ECM)*, Boletus violaceofuscus* (ECM)*, Calostoma japonicum* (ECM)*, Camarophyllus subviolaceus* (L)*, Cantharellus cibarius* (ECM)*, Clavaria aurantio-cinnabarina* (L)*, Clavaria purpurea* (L)*, Clavaria zollingeri* (L)*, Clavulina cristata* (ECM)*, Coprinus atramentarius* (L)*, Cortinarius rubicundulus* (ECM)*, Cryptoporus volvatus* (W)*, Entoloma album* (L)*, Entoloma kansaiensis* (L)*, Entoloma murraii* (L)*, Entoloma quadratus* (L)*, Fistulina hepatica* (W)*, Ganoderma applanatum* (W)*, Hygrocybe punicea* (L)*, Inocybe lutea* (ECM)*, Inonotus vallatus* (W)*, Kobayasia nipponica* (L)*, Laccaria vinaceoavellanea* (ECM)*, Lactarius gerardii* (ECM)*, Lactarius piperatus* (ECM)*, Lactarius volemus* (ECM)*, Leccinum hortonii* (ECM)*, Paxillus atrotomentosus* (W)*, Phylloporus bellus* (ECM)*, Pisolithus tinctorius* (ECM)*, Pulveroboletus ravenelii* (ECM)*, Ramariopsis fusiformis* (ECM)*, Rhizopogon superiorensis* (ECM)*, Russula alboareolata* (ECM)*, Russula amoena* (ECM)*, Russula castanopsidis* (ECM)*, Russula densifolia* (ECM)*, Russula eburneoareolata* (ECM)*, Russula japonica* (ECM)*, Russula lilacea* (ECM)*, Russula mariae* (ECM)*, Russula rosacea* (ECM)*, Russula virescens* (ECM)*, Scleroderma areolatum* (ECM)*, Scleroderma cepa* (ECM)*, Scleroderma verrucosum* (ECM)*, Strobilomyces confusus* (ECM)*, Trametes versicolor* (W)*, Tylopilus fumosipes* (ECM)*, Tylopilus nigropurpureus* (ECM)*, Tylopilus valens* (ECM)*, Xerocomus chrysenteron* (ECM)*, Xerula pudens* (W) |
| 5-Sep-82 | *Amanita farinosa* (ECM)*, Amanita rufoferruginea* (ECM)*, Amanita virosa* (ECM)*, Amanita volvata* (ECM)*, Astraeus hygrometricus* (ECM)*, Boletellus emodensis* (ECM)*, Boletus granulopunctatus* (ECM)*, Boletus ornatipes* (ECM)*, Boletus umbriniporus* (ECM)*, Calostoma japonicum* (ECM)*, Cantharellus cibarius* (ECM)*, Coltricia cinnamomea* (ECM)*, Cortinarius rubicundulus* (ECM)*, Fistulina hepatica* (W)*, Ganoderma applanatum* (W)*, Inocybe lutea* (ECM)*, Kobayasia nipponica* (L)*, Laccaria vinaceoavellanea* (ECM)*, Leccinum intusrubens* (ECM)*, Microporus affinis* (W)*, Phylloporus bellus* (ECM)*, Pisolithus tinctorius* (ECM)*, Pulveroboletus ravenelii* (ECM)*, Russula castanopsidis* (ECM)*, Russula mariae* (ECM)*, Strobilomyces confusus* (ECM)*, Xerocomus chrysenteron* (ECM) |
| 3-Oct-82 | *Amanita pantherina* (ECM)*, Amanita spreta* (ECM)*, Amanita vaginata var. vaginata* (ECM)*, Amanita virgineoides* (ECM)*, Amanita virosa* (ECM)*, Armillaria mellea* (W)*, Armillaria tabescens* (W)*, Astraeus hygrometricus* (ECM)*, Calostoma japonicum* (ECM)*, Collybia dryophila* (L)*, Coprinus atramentarius* (L)*, Coprinus disseminatus* (W)*, Cordyceps militaris (NA), Fistulina hepatica* (W)*, Hypholoma fasciculare* (W)*, Kobayasia nipponica* (L)*, Laccaria bicolor* (ECM)*, Lactarius chrysorrheus* (ECM)*, Lactarius gerardii* (ECM)*, Lactarius gracilis* (ECM)*, Leotia lubrica* (L)*, Lycoperdon perlatum* (L)*, Microporus affinis* (W)*, Pleurotus ostreatus* (W)*, Pluteus atricapillus* (W)*, Psathyrella candolliana* (W)*, Russula alboareolata* (ECM)*, Russula castanopsidis* (ECM)*, Russula cyanoxantha* (ECM)*, Russula mariae* (ECM)*, Russula sororia* (ECM)*, Russula subnigricans* (ECM)*, Stereopsis burtianum* (W)*, Suillus bovinus* (ECM)*, Suillus granulatus* (ECM) |
| 3-Nov-82 | *Astraeus hygrometricus* (ECM)*, Calostoma japonicum* (ECM)*, Ganoderma applanatum* (W)*, Hypholoma fasciculare* (W)*, Psathyrella piluliformis* (W) |
| 5-Dec-82 | *Astraeus hygrometricus* (ECM)*, Auricularia polytricha* (W)*, Calostoma japonicum* (ECM)*, Flammulina velutipes* (W)*, Hygrocybe ceracea* (L)*, Hypholoma fasciculare* (W)*, Hypholoma sublateritium* (W)*, Laccaria bicolor* (ECM)*, Lycoperdon perlatum* (L)*, Suillus bovinus* (ECM)*, Trichocoma paradoxa* (W)*, Tricholoma orirubens* (ECM) |
| 16-Jan-83 | *Astraeus hygrometricus* (ECM)*, Auricularia polytricha* (W)*, Calostoma japonicum* (ECM)*, Cyclomyces fuscus* (W)*, Flammulina velutipes* (W)*, Lycoperdon perlatum* (L)*, Mycena laevigata* (W)*, Pleurotus ostreatus* (W)*, Russula omiensis* (ECM) |
| 20-Feb-83 | *Calostoma japonicum* (ECM)*, Cryptoporus volvatus* (W)*, Flammulina velutipes* (W)*, Microporus affinis* (W)*, Pleurotus ostreatus* (W) |
| 20-Mar-83 | *Entoloma staurosporum* (L)*, Flammulina velutipes* (W)*, Microporus affinis* (W) |
| 17-Apr-83 | *Cordyceps heteropoda (NA), Entoloma staurosporum* (L)*, Exdia uvapassa* (W)*, Flammulina velutipes* (W)*, Hypholoma fasciculare* (W)*, Laccaria bicolor* (ECM)*, Lactarius camphoratus* (ECM)*, Microporus affinis* (W)*, Mycena laevigata* (W)*, Russula cyanoxantha* (ECM)*, Russula omiensis* (ECM)*, Trichocoma paradoxa* (W) |
| 22-May-83 | *Auricularia polytricha* (W)*, Coprinus disseminatus* (W)*, Cortinarius subalboviolaceus* (ECM)*, Craterellus cornucopioides* (ECM)*, Entoloma staurosporum* (L)*, Lactarius camphoratus* (ECM)*, Lactarius gracilis* (ECM)*, Mycena crocata* (W) |
| 12-Jun-83 | *Cryptoporus volvatus* (W) |
| 17-Jul-83 | *Agaricus abruptibulbus* (L)*, Amanita abrupta* (ECM)*, Amanita citrina var. citrina* (ECM)*, Amanita excelsa* (ECM)*, Amanita griseofarinosa* (ECM)*, Amanita longistriata* (ECM)*, Amanita pantherina* (ECM)*, Amanita pseudoporphyria* (ECM)*, Amanita rubescens* (ECM)*, Amanita sychnopyramis f. subannulata* (ECM)*, Amanita vaginata var. vaginata* (ECM)*, Austroboletus fusisporus* (ECM)*, Boletus auripes* (ECM)*, Boletus erythropus* (ECM)*, Boletus violaceofuscus* (ECM)*, Clavaria zollingeri* (L)*, Clavulina cristata* (ECM)*, Cortinarius salor* (ECM)*, Cortinarius subalboviolaceus* (ECM)*, Entoloma murraii* (L)*, Helvella ephippioides* (ECM)*, Laccaria bicolor* (ECM)*, Laccaria vinaceoavellanea* (ECM)*, Lactarius gracilis* (ECM)*, Phylloporus bellus* (ECM)*, Pisolithus tinctorius* (ECM)*, Psathyrella candolliana* (W)*, Pulveroboletus ravenelii* (ECM)*, Rhizopogon rubescens* (ECM)*, Russula alboareolata* (ECM)*, Russula amoena* (ECM)*, Russula castanopsidis* (ECM)*, Russula cyanoxantha* (ECM)*, Russula mariae* (ECM)*, Russula pectinata* (ECM)*, Russula violeipes* (ECM)*, Russula virescens* (ECM)*, Scleroderma areolatum* (ECM)*, Scleroderma verrucosum* (ECM)*, Strobilomyces confusus* (ECM)*, Tylopilus ballouii* (ECM)*, Tylopilus chromapes* (ECM)*, Tylopilus rugulosoreticulatus* (ECM)*, Tylopilus valens* (ECM)*, Xerocomus nigromaculatus* (ECM)*, Xerula pudens* (W) |
| 21-Aug-83 | *Amanita alboflavescens* (ECM)*, Amanita fuliginea* (ECM)*, Amanita rufoferruginea* (ECM)*, Amanita sychnopyramis f. subannulata* (ECM)*, Amanita vaginata var. vaginata* (ECM)*, Amanita virosa* (ECM)*, Amanita volvata* (ECM)*, Astraeus hygrometricus* (ECM)*, Bovista plumbea* (L)*, Calostoma japonicum* (ECM)*, Coprinus friesii* (L)*, Crepidotus mollis* (W)*, Hebeloma vinosophyllum* (ECM)*, Inocybe lutea* (ECM)*, Kobayasia nipponica* (L)*, Laccaria vinaceoavellanea* (ECM)*, Lactarius gerardii* (ECM)*, Lactarius piperatus* (ECM)*, Lactarius volemus* (ECM)*, Leotia lubrica* (L)*, Micromphale pacificum* (W)*, Paxillus atrotomentosus* (W)*, Pycnoporus coccineus* (W)*, Russula eburneoareolata* (ECM)*, Russula japonica* (ECM)*, Russula mariae* (ECM)*, Russula subnigricans* (ECM)*, Russula virescens* (ECM)*, Scleroderma verrucosum* (ECM)*, Trichocoma paradoxa* (W)*, Tylopilus nigropurpureus* (ECM)*, Xerula pudens* (W)*, Xylaria polymorpha* (W) |
| 11-Sep-83 | *Agaricus abruptibulbus* (L)*, Amanita citrina var. citrina* (ECM)*, Amanita citrina var. grisea* (ECM)*, Amanita farinosa* (ECM)*, Amanita fulva* (ECM)*, Amanita griseofarinosa* (ECM)*, Amanita spissacea* (ECM)*, Amanita sychnopyramis f. subannulata* (ECM)*, Amanita vaginata var. punctata* (ECM)*, Amanita vaginata var. vaginata* (ECM)*, Amanita virosa* (ECM)*, Amanita volvata* (ECM)*, Armillaria tabescens* (W)*, Astraeus hygrometricus* (ECM)*, Austroboletus fusisporus* (ECM)*, Austroboletus subvirens* (ECM)*, Boletellus emodensis* (ECM)*, Calostoma japonicum* (ECM)*, Calvatia craniiformis* (L)*, Collybia dryophila* (L)*, Collybia peronata* (L)*, Coprinus disseminatus* (W)*, Cortinarius rubicundulus* (ECM)*, Entoloma album* (L)*, Gyroporus castaneus* (ECM)*, Inocybe lutea* (ECM)*, Kobayasia nipponica* (L)*, Laccaria vinaceoavellanea* (ECM)*, Leotia lubrica* (L)*, Leucocoprinus fragilissimus* (L)*, Lycoperdon hiemale* (L)*, Marasmius purpureostriatus* (L)*, Phylloporus bellus* (ECM)*, Pleurotus pulmonarius* (W)*, Ramariopsis helvola* (ECM)*, Russula alboareolata* (ECM)*, Russula castanopsidis* (ECM)*, Russula foetens* (ECM)*, Russula japonica* (ECM)*, Russula mariae* (ECM)*, Tremella foliacea* (W)*, Tylopilus valens* (ECM)*, Xerula pudens* (W) |
| 30-Oct-83 | *Amanita pantherina* (ECM)*, Amanita virosa* (ECM)*, Astraeus hygrometricus* (ECM)*, Callistosporium luteoolivaceum* (L)*, Calostoma japonicum* (ECM)*, Coprinus atramentarius* (L)*, Cortinarius shigaensis* (ECM)*, Gomphidius roseus* (ECM)*, Grifola frondosa* (W)*, Hydnum repandum* (ECM)*, Hymenogaster arenarius* (ECM)*, Hypholoma fasciculare* (W)*, Ileodictyon gracile* (L)*, Laccaria bicolor* (ECM)*, Lactarius lividatus* (ECM)*, Leotia lubrica* (L)*, Morganella pyriformis* (L)*, Neolecta vittelina* (L)*, Phallus impudicus* (L)*, Rozites caperata* (ECM)*, Russula castanopsidis* (ECM)*, Suillus bovinus* (ECM)*, Suillus granulatus* (ECM)*, Suillus luteus* (ECM) |
| 13-Nov-83 | *Amanita virosa* (ECM)*, Calostoma japonicum* (ECM)*, Flammulina velutipes* (W)*, Hypholoma fasciculare* (W)*, Hypholoma sublateritium* (W)*, Ileodictyon gracile* (L)*, Laccaria bicolor* (ECM)*, Lactarius gracilis* (ECM)*, Lentinula edodes* (W)*, Morganella pyriformis* (L)*, Phallus impudicus* (L) |
| 11-Dec-83 | *Calostoma japonicum* (ECM)*, Flammulina velutipes* (W)*, Gloeophyllum sepiarium* (W)*, Hypholoma sublateritium* (W)*, Trametes versicolor* (W)*, Xylaria polymorpha* (W) |
| 21-Jan-84 | *Calostoma japonicum* (ECM)*, Flammulina velutipes* (W)*, Trametes versicolor* (W)*, Trichaptum biforme* (W) |
| 26-Feb-84 | *Astraeus hygrometricus* (ECM)*, Calostoma japonicum* (ECM)*, Mycena laevigata* (W)*, Trichocoma paradoxa* (W) |
| 20-Mar-84 | *Caloscypha fulgens* (L)*, Calostoma japonicum* (ECM)*, Trametes versicolor* (W)*, Trichocoma paradoxa* (W) |
| 22-Apr-84 | *Cordyceps japonensis (NA), Daldinia concentrica* (W)*, Hypholoma fasciculare* (W)*, Lentinula edodes* (W)*, Russula omiensis* (ECM) |
| 27-May-84 | *Agrocybe praecox* (W)*, Coprinus atramentarius* (L)*, Cordyceps heteropoda (NA), Cordyceps japonensis (NA), Cryptoporus volvatus* (W)*, Laccaria bicolor* (ECM)*, Lactarius gracilis* (ECM)*, Mycena galericulata* (W)*, Mycena haematopoda* (W)*, Pluteus atricapillus* (W)*, Pluteus aurantiorugosus* (W)*, Russula omiensis* (ECM)*, Scutellinia scutellata* (W) |
| 10-Jun-84 | *Calostoma japonicum* (ECM)*, Collybia dryophila* (L)*, Cryptoporus volvatus* (W)*, Lactarius gracilis* (ECM)*, Pluteus atricapillus* (W)*, Polyporus arcularius* (W)*, Russula omiensis* (ECM)*, Xylaria polymorpha* (W) |
| 8-Jul-84 | *Agrocybe cylindracea* (W)*, Amanita excelsa* (ECM)*, Amanita farinosa* (ECM)*, Amanita pantherina* (ECM)*, Amanita pseudoporphyria* (ECM)*, Amanita rubescens* (ECM)*, Amanita sychnopyramis f. subannulata* (ECM)*, Amanita vaginata var. vaginata* (ECM)*, Astraeus hygrometricus* (ECM)*, Boletellus emodensis* (ECM)*, Boletus edulis* (ECM)*, Boletus erythropus* (ECM)*, Boletus violaceofuscus* (ECM)*, Cantharellus minor* (ECM)*, Coprinus disseminatus* (W)*, Cortinarius subalboviolaceus* (ECM)*, Entoloma album* (L)*, Entoloma murraii* (L)*, Gyroporus castaneus* (ECM)*, Hebeloma vinosophyllum* (ECM)*, Inocybe asterospora* (ECM)*, Inocybe fastigiata* (ECM)*, Laccaria bicolor* (ECM)*, Laccaria vinaceoavellanea* (ECM)*, Lactarius gracilis* (ECM)*, Lactarius piperatus* (ECM)*, Lactarius volemus* (ECM)*, Lepiota atrosquamulosa* (L)*, Marasmius maximus* (L)*, Nomuraea atypicola (NA), Phylloporus bellus* (ECM)*, Ramariopsis fusiformis* (ECM)*, Russula castanopsidis* (ECM)*, Russula cyanoxantha* (ECM)*, Russula mariae* (ECM)*, Russula virescens* (ECM)*, Sparassis crispa* (W)*, Strobilomyces confusus* (ECM)*, Tylopilus fumosipes* (ECM)*, Tylopilus rugulosoreticulatus* (ECM)*, Xylaria polymorpha* (W) |
| 1-Aug-84 | *Agrocybe cylindracea* (W)*, Amanita citrina var. grisea* (ECM)*, Amanita flavipes* (ECM)*, Amanita fuliginea* (ECM)*, Amanita griseofarinosa* (ECM)*, Amanita pantherina* (ECM)*, Amanita pseudoporphyria* (ECM)*, Amanita sychnopyramis f. subannulata* (ECM)*, Amanita vaginata var. vaginata* (ECM)*, Amanita virgineoides* (ECM)*, Amanita volvata* (ECM)*, Armillaria tabescens* (W)*, Astraeus hygrometricus* (ECM)*, Austroboletus subvirens* (ECM)*, Boletellus emodensis* (ECM)*, Boletellus russellii* (ECM)*, Boletus auripes* (ECM)*, Boletus griseus* (ECM)*, Boletus obscureumbrinus* (ECM)*, Boletus umbriniporus* (ECM)*, Boletus violaceofuscus* (ECM)*, Coprinus disseminatus* (W)*, Cryptoporus volvatus* (W)*, Entoloma quadratus* (L)*, Laccaria vinaceoavellanea* (ECM)*, Lactarius gerardii* (ECM)*, Lactarius volemus* (ECM)*, Leccinum extremiorientale* (ECM)*, Leccinum hortonii* (ECM)*, Phylloporus bellus* (ECM)*, Pisolithus tinctorius* (ECM)*, Psathyrella candolliana* (W)*, Pulveroboletus ravenelii* (ECM)*, Rhizopogon rubescens* (ECM)*, Russula alboareolata* (ECM)*, Russula castanopsidis* (ECM)*, Russula cyanoxantha* (ECM)*, Russula densifolia* (ECM)*, Russula japonica* (ECM)*, Russula lilacea* (ECM)*, Russula rosacea* (ECM)*, Russula virescens* (ECM)*, Strobilomyces confusus* (ECM)*, Strobilomyces seminudus* (ECM)*, Tylopilus ballouii* (ECM)*, Tylopilus valens* (ECM)*, Xerocomus nigromaculatus* (ECM)*, Xerula pudens* (W) |
| 9-Sep-84 | *Amanita fuliginea* (ECM)*, Amanita pseudoporphyria* (ECM)*, Amanita rufoferruginea* (ECM)*, Amanita sychnopyramis f. subannulata* (ECM)*, Amanita virgineoides* (ECM)*, Armillaria tabescens* (W)*, Astraeus hygrometricus* (ECM)*, Boletellus emodensis* (ECM)*, Boletus erythropus* (ECM)*, Boletus umbriniporus* (ECM)*, Calostoma japonicum* (ECM)*, Calvatia craniiformis* (L)*, Clavulina cristata* (ECM)*, Collybia peronata* (L)*, Coprinus disseminatus* (W)*, Cortinarius rubicundulus* (ECM)*, Cryptoporus volvatus* (W)*, Ganoderma lucidum* (W)*, Laccaria laccata* (ECM)*, Lycoperdon hiemale* (L)*, Marasmius maximus* (L)*, Pisolithus tinctorius* (ECM)*, Rhizopogon nigrescens* (ECM)*, Russula alboareolata* (ECM)*, Russula castanopsidis* (ECM)*, Russula japonica* (ECM)*, Russula subnigricans* (ECM)*, Russula virescens* (ECM)*, Sparassis crispa* (W)*, Vibrissea truncotum* (W)*, Xeromphalina campanella* (W) |
| 7-Oct-84 | *Agrocybe cylindracea* (W)*, Amanita farinosa* (ECM)*, Amanita vaginata var. vaginata* (ECM)*, Astraeus hygrometricus* (ECM)*, Boletellus emodensis* (ECM)*, Gyroporus castaneus* (ECM)*, Lactarius piperatus* (ECM)*, Nomuraea atypicola (NA), Ramariopsis fusiformis* (ECM)*, Russula mariae* (ECM)*, Russula virescens* (ECM) |
| 18-Nov-84 | *Antrodiella zonata* (W)*, Astraeus hygrometricus* (ECM)*, Auricularia polytricha* (W)*, Calostoma japonicum* (ECM)*, Coprinus atramentarius* (L)*, Fistulina hepatica* (W)*, Hymenogaster arenarius* (ECM)*, Hypholoma fasciculare* (W)*, Lentinula edodes* (W)*, Leotia lubrica* (L)*, Neolentinus lepideus* (W)*, Trichaptum biforme* (W) |
| 9-Dec-84 | *Calostoma japonicum* (ECM)*, Hypholoma fasciculare* (W)*, Lentinellus ursinus* (W)*, Microporus affinis* (W)*, Psathyrella candolliana* (W) |
| 13-Jan-85 | *Antrodiella zonata* (W)*, Astraeus hygrometricus* (ECM)*, Cyclomyces fuscus* (W)*, Daedaleopsis styracina* (W)*, Flammulina velutipes* (W)*, Hypholoma fasciculare* (W)*, Phellinus substygius* (W)*, Trametes versicolor* (W)*, Xylaria polymorpha* (W) |
| 24-Feb-85 | *Antrodiella zonata* (W)*, Calostoma japonicum* (ECM)*, Exdia uvapassa* (W)*, Flammulina velutipes* (W)*, Lentinula edodes* (W)*, Microporus affinis* (W)*, Mycena laevigata* (W)*, Porodisculus pendulus* (W)*, Russula omiensis* (ECM)*, Trametes versicolor* (W)*, Tremella foliacea* (W) |
| 24-Mar-85 | *Coltricia pusilla* (ECM)*, Cryptoporus volvatus* (W)*, Cyclomyces fuscus* (W)*, Entoloma staurosporum* (L)*, Exdia glandulosa* (W)*, Hypholoma fasciculare* (W)*, Lentinula edodes* (W)*, Lycoperdon perlatum* (L)*, Microporus affinis* (W)*, Mycena laevigata* (W)*, Radulodon capelandii* (W)*, Russula omiensis* (ECM) |
| 21-Apr-85 | *Artomyces pyxidatus* (W)*, Coprinus atramentarius* (L)*, Cordyceps heteropoda (NA), Cryptoporus volvatus* (W)*, Cyclomyces fuscus* (W)*, Entoloma staurosporum* (L)*, Flammulina velutipes* (W)*, Hypholoma fasciculare* (W)*, Lactarius camphoratus* (ECM)*, Lentinula edodes* (W)*, Mycena alcalina* (W)*, Mycena crocata* (W)*, Pholiota highlandensis* (W)*, Psathyrella candolliana* (W)*, Tremella fuciformis* (W) |
| 19-May-85 | *Agrocybe praecox* (W)*, Coprinus atramentarius* (L)*, Cryptoporus volvatus* (W)*, Entoloma staurosporum* (L)*, Laccaria bicolor* (ECM)*, Lactarius camphoratus* (ECM)*, Lactarius gracilis* (ECM)*, Microporus affinis* (W)*, Pholiota highlandensis* (W)*, Pholiota terrestris* (W)*, Scutellinia scutellata* (W)*, Tyromyces chioneus* (W) |
| 30-Jun-85 | *Agrocybe cylindracea* (W)*, Amanita farinosa* (ECM)*, Amanita punctata* (ECM)*, Amanita rubescens* (ECM)*, Amanita vaginata var. vaginata* (ECM)*, Astraeus hygrometricus* (ECM)*, Auricularia polytricha* (W)*, Boletus fraternus* (ECM)*, Calocera cornea* (W)*, Cantharellus minor* (ECM)*, Clavulina cristata* (ECM)*, Coprinus atramentarius* (L)*, Cordyceps annullata (NA), Cortinarius salor* (ECM)*, Cortinarius subalboviolaceus* (ECM)*, Entoloma murraii* (L)*, Gyroporus castaneus* (ECM)*, Hygrocybe cuspidata* (L)*, Laccaria bicolor* (ECM)*, Laccaria vinaceoavellanea* (ECM)*, Lactarius akahatsu* (ECM)*, Lactarius camphoratus* (ECM)*, Lactarius gracilis* (ECM)*, Lactarius volemus* (ECM)*, Lentinula edodes* (W)*, Marasmius maximus* (L)*, Marasmius siccus* (L)*, Microporus affinis* (W)*, Microporus vernicipes* (W)*, Pholiota highlandensis* (W)*, Phylloporus bellus* (ECM)*, Rhizopogon superiorensis* (ECM)*, Russula cyanoxantha* (ECM)*, Russula pectinata* (ECM)*, Russula virescens* (ECM)*, Suillus granulatus* (ECM)*, Tremella fuciformis* (W)*, Tylopilus chromapes* (ECM) |
| 28-Jul-85 | *Amanita abrupta* (ECM)*, Amanita citrina var. citrina* (ECM)*, Amanita fuliginea* (ECM)*, Amanita hongoi* (ECM)*, Amanita pseudoporphyria* (ECM)*, Amanita volvata* (ECM)*, Astraeus hygrometricus* (ECM)*, Austroboletus fusisporus* (ECM)*, Boletellus emodensis* (ECM)*, Boletellus obscurecoccineus* (ECM)*, Boletus pulverulentus* (ECM)*, Cantharellus minor* (ECM)*, Clavaria purpurea* (L)*, Cryptoporus volvatus* (W)*, Heimiella japonica* (ECM)*, Helvella ephippioides* (ECM)*, Hygrocybe cuspidata* (L)*, Inocybe lutea* (ECM)*, Laccaria vinaceoavellanea* (ECM)*, Lactarius piperatus* (ECM)*, Lactarius subvellereus* (ECM)*, Megacollybia platyphylla* (W)*, Nomuraea atypicola (NA), Paxillus atrotomentosus* (W)*, Pisolithus tinctorius* (ECM)*, Psathyrella candolliana* (W)*, Pulveroboletus ravenelii* (ECM)*, Russula castanopsidis* (ECM)*, Russula cyanoxantha* (ECM)*, Russula japonica* (ECM)*, Scleroderma areolatum* (ECM)*, Tylopilus ballouii* (ECM)*, Tylopilus rugulosoreticulatus* (ECM)*, Xylaria polymorpha* (W) |
| 18-Aug-85 | *Amanita farinosa* (ECM)*, Astraeus hygrometricus* (ECM)*, Austroboletus fusisporus* (ECM)*, Boletellus emodensis* (ECM)*, Ganoderma applanatum* (W)*, Laccaria vinaceoavellanea* (ECM)*, Laetiporus versisporus* (W)*, Leccinum intusrubens* (ECM)*, Microporus affinis* (W)*, Neolentinus lepideus* (W)*, Paxillus atrotomentosus* (W)*, Russula castanopsidis* (ECM)*, Russula subnigricans* (ECM)*, Thelephora terrestris* (ECM)*, Trametes versicolor* (W) |
| 29-Sep-85 | *Agrocybe cylindracea* (W)*, Agrocybe erebia* (W)*, Amanita pantherina* (ECM)*, Amanita rubescens* (ECM)*, Amanita spissacea* (ECM)*, Amanita vaginata var. vaginata* (ECM)*, Amanita virosa* (ECM)*, Armillaria tabescens* (W)*, Astraeus hygrometricus* (ECM)*, Aureoboletus thibetanus* (ECM)*, Austroboletus fusisporus* (ECM)*, Austroboletus subvirens* (ECM)*, Boletellus russellii* (ECM)*, Boletus erythropus* (ECM)*, Boletus griseus* (ECM)*, Boletus violaceofuscus* (ECM)*, Calvatia craniiformis* (L)*, Collybia dryophila* (L)*, Collybia peronata* (L)*, Coprinus disseminatus* (W)*, Cortinarius rubicundulus* (ECM)*, Crucibulum leave* (W)*, Ganoderma applanatum* (W)*, Hebeloma vinosophyllum* (ECM)*, Heimiella japonica* (ECM)*, Hypholoma fasciculare* (W)*, Leccinum extremiorientale* (ECM)*, Leccinum hortonii* (ECM)*, Lenzites betulinus* (W)*, Leotia lubrica* (L)*, Lepiota cristata* (L)*, Lepista sordida* (L)*, Leucocoprinus fragilissimus* (L)*, Lycoperdon perlatum* (L)*, Paxillus atrotomentosus* (W)*, Phylloporus bellus* (ECM)*, Pisolithus tinctorius* (ECM)*, Rhizopogon nigrescens* (ECM)*, Rhizopogon rubescens* (ECM)*, Russula amoena* (ECM)*, Russula pectinata* (ECM)*, Russula subnigricans* (ECM)*, Stereum ostrea* (W)*, Suillus granulatus* (ECM)*, Trametes versicolor* (W)*, Trichocoma paradoxa* (W)*, Tylopilus chromapes* (ECM)*, Tyromyces chioneus* (W)*, Xerocomus chrysenteron* (ECM) |
| 13-Oct-85 | *Aleuria aurantia* (L)*, Amanita abrupta* (ECM)*, Amanita farinosa* (ECM)*, Amanita fulva* (ECM)*, Amanita neoovoidea* (ECM)*, Amanita pseudoporphyria* (ECM)*, Amanita rubescens* (ECM)*, Amanita vaginata var. punctata* (ECM)*, Amanita vaginata var. vaginata* (ECM)*, Amanita volvata* (ECM)*, Armillaria mellea* (W)*, Austroboletus fusisporus* (ECM)*, Austroboletus subvirens* (ECM)*, Boletus fraternus* (ECM)*, Boletus pseudocalopus* (ECM)*, Calostoma japonicum* (ECM)*, Clavulina cristata* (ECM)*, Cortinarius rubicundulus* (ECM)*, Cortinarius subalboviolaceus* (ECM)*, Entoloma murraii* (L)*, Flammulina velutipes* (W)*, Gomphidius roseus* (ECM)*, Hypholoma fasciculare* (W)*, Laccaria bicolor* (ECM)*, Laccaria vinaceoavellanea* (ECM)*, Lactarius chrysorrheus* (ECM)*, Lactarius gracilis* (ECM)*, Laetiporus versisporus* (W)*, Lyophyllum decastes* (L)*, Phylloporus bellus* (ECM)*, Pisolithus tinctorius* (ECM)*, Psathyrella velutina* (W)*, Psilocybe argentipes* (L)*, Russula castanopsidis* (ECM)*, Russula cyanoxantha* (ECM)*, Russula densifolia* (ECM)*, Russula mariae* (ECM)*, Strobilomyces confusus* (ECM)*, Suillus bovinus* (ECM)*, Suillus granulatus* (ECM)*, Suillus luteus* (ECM) |
| 17-Nov-85 | *Antrodiella zonata* (W)*, Astraeus hygrometricus* (ECM)*, Bjerkandera adusta* (W)*, Calostoma japonicum* (ECM)*, Coltricia pusilla* (ECM)*, Coprinus atramentarius* (L)*, Cortinarius bovinus* (ECM)*, Craterellus cornucopioides* (ECM)*, Crepidotus badiofloccosus* (W)*, Cryptoporus volvatus* (W)*, Cyclomyces fuscus* (W)*, Hypholoma fasciculare* (W)*, Hypholoma sublateritium* (W)*, Lactarius gracilis* (ECM)*, Lentinellus ursinus* (W)*, Lentinula edodes* (W)*, Leotia lubrica* (L)*, Merulius tremellosus* (W)*, Microporus affinis* (W)*, Oligoporus caesius* (W)*, Russula omiensis* (ECM)*, Stereum hirsutum* (W)*, Trametes versicolor* (W)*, Tremella fuciformis* (W)*, Trichaptum abietinum* (W) |
| 22-Dec-85 | *Antrodiella zonata* (W)*, Calostoma japonicum* (ECM)*, Crucibulum leave* (W)*, Cryptoporus volvatus* (W)*, Cyathus striatus* (W)*, Flammulina velutipes* (W)*, Gloeophyllum abietinum* (W)*, Hypholoma fasciculare* (W)*, Ileodictyon gracile* (L)*, Lentinula edodes* (W)*, Lenzites betulinus* (W)*, Russula omiensis* (ECM)*, Scleroderma areolatum* (ECM)*, Trametes versicolor* (W) |
| 12-Jan-86 | *Cyclomyces fuscus* (W)*, Ganoderma applanatum* (W)*, Gloeophyllum subferrugineum* (W)*, Laetiporus versisporus* (W)*, Microporus affinis* (W)*, Russula rosea* (ECM)*, Scleroderma verrucosum* (ECM)*, Trametes versicolor* (W) |
| 9-Feb-86 | *Coltricia cinnamomea* (ECM)*, Coltricia pusilla* (ECM)*, Crucibulum leave* (W)*, Cyclomyces fuscus* (W)*, Gloeophyllum subferrugineum* (W)*, Hypholoma fasciculare* (W)*, Hypoxylon fragiforme* (W)*, Microporus affinis* (W)*, Russula omiensis* (ECM)*, Stereum ochraceo-flavum* (W)*, Tyromyces chioneus* (W) |
| 9-Mar-86 | *Coltricia pusilla* (ECM)*, Daedaleopsis purpurea* (W)*, Gloeoporus dichrous* (W)*, Hypholoma sublateritium* (W)*, Inonotus xeranticus* (W)*, Laetiporus versisporus* (W)*, Lentinula edodes* (W)*, Phellinus umbrinellus* (W)*, Polyporus alveolarius* (W)*, Russula omiensis* (ECM)*, Trametes versicolor* (W) |
| 13-Apr-86 | *Astraeus hygrometricus* (ECM)*, Coltricia pusilla* (ECM)*, Cordyceps japonensis (NA), Cordyceps ormicarum (NA), Daedalea dickinsii* (W)*, Flammulina velutipes* (W)*, Ganoderma applanatum* (W)*, Hypholoma fasciculare* (W)*, Lentinula edodes* (W)*, Lenzites betulinus* (W)*, Panellus stypticus* (W)*, Pleurotus ostreatus* (W)*, Russula omiensis* (ECM) |
| 11-May-86 | *Agrocybe cylindracea* (W)*, Collybia confluens* (L)*, Coprinus atramentarius* (L)*, Cordyceps annullata (NA), Cordyceps heteropoda (NA), Cordyceps ophioglossoides (NA), Hygrocybe psittacina* (L)*, Lactarius camphoratus* (ECM)*, Lentinula edodes* (W)*, Neolentinus lepideus* (W)*, Nidula niveo-tomentosa* (W)*, Pluteus atricapillus* (W)*, Russula fragilis* (ECM)*, Russula rosea* (ECM) |
| 18-Jul-86 | *Agrocybe cylindracea* (W)*, Amanita pantherina* (ECM)*, Amanita pseudoporphyria* (ECM)*, Amanita spissacea* (ECM)*, Amanita vaginata var. vaginata* (ECM)*, Armillaria tabescens* (W)*, Artomyces pyxidatus* (W)*, Astraeus hygrometricus* (ECM)*, Aureoboletus thibetanus* (ECM)*, Boletus fraternus* (ECM)*, Cantharellus cibarius* (ECM)*, Cantharellus minor* (ECM)*, Clavaria purpurea* (L)*, Clavulina cristata* (ECM)*, Coltricia pusilla* (ECM)*, Coprinus radians* (W)*, Cortinarius anomalus* (ECM)*, Cortinarius galeroides* (ECM)*, Cortinarius subalboviolaceus* (ECM)*, Craterellus cornucopioides* (ECM)*, Cryptoporus volvatus* (W)*, Cyclomyces fuscus* (W)*, Entoloma murraii* (L)*, Entoloma quadratus* (L)*, Ganoderma applanatum* (W)*, Gerronema fibula* (L)*, Gyroporus castaneus* (ECM)*, Helvella ephippioides* (ECM)*, Hygrocybe cantharellus* (L)*, Hygrocybe psittacina* (L)*, Inocybe lutea* (ECM)*, Laccaria vinaceoavellanea* (ECM)*, Lactarius piperatus* (ECM)*, Leccinum extremiorientale* (ECM)*, Marasmius maximus* (L)*, Microporus affinis* (W)*, Nidula niveo-tomentosa* (W)*, Nomuraea atypicola (NA), Paxillus atrotomentosus* (W)*, Phylloporus bellus* (ECM)*, Pisolithus tinctorius* (ECM)*, Pulveroboletus ravenelii* (ECM)*, Pycnoporus coccineus* (W)*, Ramariopsis fusiformis* (ECM)*, Russula alboareolata* (ECM)*, Russula amoena* (ECM)*, Russula castanopsidis* (ECM)*, Russula compacta* (ECM)*, Russula densifolia* (ECM)*, Russula foetens* (ECM)*, Russula mariae* (ECM)*, Russula sororia* (ECM)*, Russula violeipes* (ECM)*, Russula virescens* (ECM)*, Scleroderma areolatum* (ECM)*, Sparassis crispa* (W)*, Strobilomyces confusus* (ECM)*, Trametes versicolor* (W)*, Trichoglossum hirsutum* (L)*, Tylopilus nigropurpureus* (ECM)*, Xerocomus nigromaculatus* (ECM)*, Xerula pudens* (W)*, Xerula radicata* (W) |
| 17-Aug-86 | *Amanita spissacea* (ECM)*, Amanita volvata* (ECM)*, Armillaria tabescens* (W)*, Astraeus hygrometricus* (ECM)*, Boletellus emodensis* (ECM)*, Boletellus obscurecoccineus* (ECM)*, Boletus ornatipes* (ECM)*, Boletus umbriniporus* (ECM)*, Clavulina cristata* (ECM)*, Coltricia pusilla* (ECM)*, Coprinus atramentarius* (L)*, Cordyceps sobolifera (NA), Cryptoporus volvatus* (W)*, Cyclomyces fuscus* (W)*, Daldinia concentrica* (W)*, Leccinum intusrubens* (ECM)*, Neolentinus lepideus* (W)*, Nidula niveo-tomentosa* (W)*, Nomuraea atypicola (NA), Paxillus atrotomentosus* (W)*, Phylloporus bellus* (ECM)*, Russula alboareolata* (ECM)*, Russula castanopsidis* (ECM)*, Russula mariae* (ECM)*, Russula violeipes* (ECM)*, Scleroderma areolatum* (ECM)*, Tylopilus ballouii* (ECM)*, Xylaria polymorpha* (W) |
| 14-Sep-86 | *Antrodiella zonata* (W)*, Astraeus hygrometricus* (ECM)*, Auricularia polytricha* (W)*, Boletus fraternus* (ECM)*, Calostoma japonicum* (ECM)*, Cryptoporus volvatus* (W)*, Cyclomyces fuscus* (W)*, Daedalea dickinsii* (W)*, Inocybe lutea* (ECM)*, Kobayasia nipponica* (L)*, Lentinula edodes* (W)*, Lenzites betulinus* (W)*, Microporus affinis* (W)*, Neolentinus lepideus* (W)*, Nidula niveo-tomentosa* (W)*, Oligoporus caesius* (W)*, Paxillus atrotomentosus* (W)*, Phylloporus bellus* (ECM)*, Russula subnigricans* (ECM)*, Sparassis crispa* (W)*, Xylobolus spectabilis* (W) |
| 5-Oct-86 | *Agrocybe cylindracea* (W)*, Amanita pseudoporphyria* (ECM)*, Amanita punctata* (ECM)*, Armillaria mellea* (W)*, Astraeus hygrometricus* (ECM)*, Cortinarius salor* (ECM)*, Gomphidius roseus* (ECM)*, Grifola frondosa* (W)*, Gymnopilus liquiritiae* (W)*, Hypholoma fasciculare* (W)*, Laccaria vinaceoavellanea* (ECM)*, Lactarius gracilis* (ECM)*, Lenzites betulinus* (W)*, Lepiota atrosquamulosa* (L)*, Microporus affinis* (W)*, Nidula niveo-tomentosa* (W)*, Russula cyanoxantha* (ECM)*, Russula eburneoareolata* (ECM)*, Thelephora terrestris* (ECM)*, Tyromyces chioneus* (W)*, Xeromphalina campanella* (W) |
| 9-Nov-86 | *Agrocybe farinacea* (W)*, Amanita virosa* (ECM)*, Armillaria mellea* (W)*, Astraeus hygrometricus* (ECM)*, Coprinus atramentarius* (L)*, Hypholoma fasciculare* (W)*, Russula compacta* (ECM)*, Russula omiensis* (ECM)*, Russula vesca* (ECM) |
| 7-Dec-86 | *Astraeus hygrometricus* (ECM)*, Calostoma japonicum* (ECM)*, Hypholoma fasciculare* (W)*, Ileodictyon gracile* (L)*, Lentinula edodes* (W)*, Microporus affinis* (W)*, Nidula niveo-tomentosa* (W)*, Panellus stypticus* (W) |
| 25-Jan-87 | *Astraeus hygrometricus* (ECM)*, Flammulina velutipes* (W)*, Galerina pseudocamerina* (L)*, Lentinula edodes* (W)*, Nidula niveo-tomentosa* (W)*, Russula omiensis* (ECM)*, Xeromphalina campanella* (W) |
| 22-Feb-87 | *Astraeus hygrometricus* (ECM)*, Calostoma japonicum* (ECM)*, Lentinula edodes* (W)*, Polyporus arcularius* (W)*, Russula omiensis* (ECM)*, Trametes versicolor* (W) |
| 29-Mar-87 | *Calostoma japonicum* (ECM)*, Cordyceps sphecocephala (NA), Cryptoporus volvatus* (W)*, Elaphocordyceps longisegmentis (NA), Hypholoma fasciculare* (W)*, Lentinula edodes* (W)*, Lycoperdon perlatum* (L)*, Mycena alcalina* (W)*, Psathyrella candolliana* (W)*, Russula omiensis* (ECM)*, Trichaptum biforme* (W) |
| 26-Apr-87 | *Astraeus hygrometricus* (ECM)*, Crepidotus mollis* (W)*, Daedaleopsis tricolor* (W)*, Hypholoma fasciculare* (W)*, Lentinula edodes* (W)*, Lenzites betulinus* (W)*, Microporus affinis* (W)*, Russula omiensis* (ECM)*, Russula rosea* (ECM)*, Schizophyllum commune* (W)*, Trichaptum abietinum* (W) |
| 31-May-87 | *Agrocybe cylindracea* (W)*, Amanita citrina var. citrina* (ECM)*, Amanita pseudoporphyria* (ECM)*, Clavulina rugosa* (ECM)*, Collybia confluens* (L)*, Cordyceps heteropoda (NA), Cordyceps japonensis (NA), Cortinarius violaceus* (ECM)*, Craterellus cornucopioides* (ECM)*, Cyclomyces fuscus* (W)*, Gymnopilus liquiritiae* (W)*, Hypholoma fasciculare* (W)*, Lactarius gracilis* (ECM)*, Megacollybia platyphylla* (W)*, Polyporus arcularius* (W)*, Psathyrella velutina* (W)*, Russula omiensis* (ECM)*, Russula rosea* (ECM) |
| 21-Jun-87 | *Agaricus abruptibulbus* (L)*, Amanita pantherina* (ECM)*, Amanita rubescens* (ECM)*, Amanita spreta* (ECM)*, Amanita sychnopyramis f. subannulata* (ECM)*, Astraeus hygrometricus* (ECM)*, Boletus erythropus* (ECM)*, Boletus fraternus* (ECM)*, Cordyceps annullata (NA), Cordyceps heteropoda (NA), Cortinarius galeroides* (ECM)*, Cortinarius subalboviolaceus* (ECM)*, Crepidotus subsphaerosporus* (W)*, Cryptoporus volvatus* (W)*, Entoloma murraii* (L)*, Fistulina hepatica* (W)*, Galiella celebica* (W)*, Inocybe lacera* (ECM)*, Laccaria vinaceoavellanea* (ECM)*, Lactarius camphoratus* (ECM)*, Lactarius gracilis* (ECM)*, Lepiota atrosquamulosa* (L)*, Leucocoprinus fragilissimus* (L)*, Marasmius maximus* (L)*, Microporus affinis* (W)*, Nidula niveo-tomentosa* (W)*, Nidularia farcta* (W)*, Phylloporus bellus* (ECM)*, Pluteus nanus* (W)*, Polyporus alveolarius* (W)*, Psilocybe argentipes* (L)*, Pycnoporus coccineus* (W)*, Rhizopogon nigrescens* (ECM)*, Rhizopogon superiorensis* (ECM)*, Russula alboareolata* (ECM)*, Russula compacta* (ECM)*, Russula mariae* (ECM)*, Russula rosea* (ECM)*, Schizophyllum commune* (W)*, Tylopilus chromapes* (ECM)*, Xerula radicata* (W) |
| 12-Jul-87 | *Agaricus abruptibulbus* (L)*, Agaricus subrutilescens* (L)*, Agrocybe cylindracea* (W)*, Amanita citrina var. citrina* (ECM)*, Amanita farinosa* (ECM)*, Amanita griseofarinosa* (ECM)*, Amanita pantherina* (ECM)*, Amanita pseudoporphyria* (ECM)*, Amanita spissacea* (ECM)*, Amanita spreta* (ECM)*, Amanita sychnopyramis f. subannulata* (ECM)*, Amanita vaginata var. vaginata* (ECM)*, Armillaria tabescens* (W)*, Astraeus hygrometricus* (ECM)*, Austroboletus fusisporus* (ECM)*, Austroboletus subvirens* (ECM)*, Boletus aokii* (ECM)*, Boletus edulis* (ECM)*, Boletus fraternus* (ECM)*, Boletus subvelutipes* (ECM)*, Boletus violaceofuscus* (ECM)*, Cantharellus minor* (ECM)*, Cordyceps annullata (NA), Cordyceps japonensis (NA), Cordyceps sobolifera (NA), Cortinarius galeroides* (ECM)*, Cortinarius subalboviolaceus* (ECM)*, Cryptoporus volvatus* (W)*, Entoloma murraii* (L)*, Gyroporus castaneus* (ECM)*, Hebeloma vinosophyllum* (ECM)*, Helvella ephippioides* (ECM)*, Inocybe asterospora* (ECM)*, Inocybe fastigiata* (ECM)*, Inocybe lutea* (ECM)*, Laccaria vinaceoavellanea* (ECM)*, Lactarius akahatsu* (ECM)*, Lactarius chrysorrheus* (ECM)*, Lepiota atrosquamulosa* (L)*, Lepiota cygnea* (L)*, Lepiota pseudogranulosa* (L)*, Microporus affinis* (W)*, Nidularia farcta* (W)*, Nomuraea atypicola (NA), Paxillus atrotomentosus* (W)*, Pholiota terrestris* (W)*, Phylloporus bellus* (ECM)*, Psilocybe argentipes* (L)*, Ramariopsis fusiformis* (ECM)*, Russula alboareolata* (ECM)*, Russula castanopsidis* (ECM)*, Russula cyanoxantha* (ECM)*, Russula mariae* (ECM)*, Russula rosea* (ECM)*, Russula senecis* (ECM)*, Russula sororia* (ECM)*, Sparassis crispa* (W)*, Strobilomyces confusus* (ECM)*, Tylopilus fumosipes* (ECM)*, Tylopilus valens* (ECM)*, Xerocomus chrysenteron* (ECM)*, Xerocomus nigromaculatus* (ECM)*, Xerula radicata* (W)*, Xylaria polymorpha* (W) |
| 16-Aug-87 | *Agrocybe cylindracea* (W)*, Amanita ceciliae* (ECM)*, Amanita fuliginea* (ECM)*, Amanita griseofarinosa* (ECM)*, Amanita pseudoporphyria* (ECM)*, Amanita spissacea* (ECM)*, Amanita vaginata var. vaginata* (ECM)*, Amanita virosa* (ECM)*, Amanita volvata* (ECM)*, Artomyces pyxidatus* (W)*, Austroboletus fusisporus* (ECM)*, Austroboletus subvirens* (ECM)*, Boletellus emodensis* (ECM)*, Boletus fraternus* (ECM)*, Boletus obscureumbrinus* (ECM)*, Boletus ornatipes* (ECM)*, Boletus subvelutipes* (ECM)*, Boletus violaceofuscus* (ECM)*, Cantharellus minor* (ECM)*, Clavulina cristata* (ECM)*, Cordyceps sobolifera (NA), Cyclomyces fuscus* (W)*, Entoloma murraii* (L)*, Gloeophyllum abietinum* (W)*, Gyroporus castaneus* (ECM)*, Inonotus mikadoi* (W)*, Kobayasia nipponica* (L)*, Laccaria vinaceoavellanea* (ECM)*, Lactarius gerardii* (ECM)*, Lactarius volemus* (ECM)*, Leccinum hortonii* (ECM)*, Leccinum intusrubens* (ECM)*, Megacollybia platyphylla* (W)*, Nidula niveo-tomentosa* (W)*, Paxillus atrotomentosus* (W)*, Paxillus cutisii* (W)*, Phylloporus bellus* (ECM)*, Pluteus atricapillus* (W)*, Pulveroboletus ravenelii* (ECM)*, Ramariopsis fusiformis* (ECM)*, Russula alboareolata* (ECM)*, Russula castanopsidis* (ECM)*, Russula densifolia* (ECM)*, Russula lilacea* (ECM)*, Russula mariae* (ECM)*, Russula neoemetica* (ECM)*, Russula rosacea* (ECM)*, Russula subnigricans* (ECM)*, Russula virescens* (ECM)*, Strobilomyces confusus* (ECM)*, Trametes versicolor* (W)*, Tylopilus ferrugineus* (ECM)*, Xerocomus nigromaculatus* (ECM) |
| 20-Sep-87 | *Agrocybe cylindracea* (W)*, Amanita volvata* (ECM)*, Armillaria tabescens* (W)*, Boletellus russellii* (ECM)*, Callistosporium luteoolivaceum* (L)*, Calostoma japonicum* (ECM)*, Calvatia craniiformis* (L)*, Coltricia pusilla* (ECM)*, Coprinus disseminatus* (W)*, Cryptoporus volvatus* (W)*, Cyclomyces fuscus* (W)*, Daedaleopsis styracina* (W)*, Galiella celebica* (W)*, Gloeophyllum sepiarium* (W)*, Grifola frondosa* (W)*, Gymnopilus spectabilis* (W)*, Hypholoma fasciculare* (W)*, Kobayasia nipponica* (L)*, Laccaria vinaceoavellanea* (ECM)*, Lentinellus ursinus* (W)*, Lenzites betulinus* (W)*, Leotia lubrica* (L)*, Lycoperdon perlatum* (L)*, Microporus affinis* (W)*, Pleurotus pulmonarius* (W)*, Russula castanopsidis* (ECM)*, Russula mariae* (ECM)*, Russula neoemetica* (ECM)*, Russula subnigricans* (ECM)*, Stereopsis burtianum* (W)*, Trichaptum abietinum* (W)*, Tylopilus castaneiceps* (ECM)*, Xylobolus spectabilis* (W) |
| 11-Oct-87 | *Agaricus abruptibulbus* (L)*, Agrocybe cylindracea* (W)*, Amanita sychnopyramis f. subannulata* (ECM)*, Amanita virosa* (ECM)*, Astraeus hygrometricus* (ECM)*, Calvatia craniiformis* (L)*, Clavulina rugosa* (ECM)*, Cortinarius anomalus* (ECM)*, Cortinarius galeroides* (ECM)*, Cryptoporus volvatus* (W)*, Daedaleopsis purpurea* (W)*, Galiella celebica* (W)*, Ganoderma lucidum* (W)*, Gomphidius roseus* (ECM)*, Gymnopilus liquiritiae* (W)*, Hypholoma fasciculare* (W)*, Inonotus vallatus* (W)*, Kobayasia nipponica* (L)*, Laccaria vinaceoavellanea* (ECM)*, Lactarius lividatus* (ECM)*, Lactarius subzonarius* (ECM)*, Lycoperdon perlatum* (L)*, Lyophyllum decastes* (L)*, Microporus affinis* (W)*, Morganella pyriformis* (L)*, Mycena haematopoda* (W)*, Mycena laevigata* (W)*, Neolentinus lepideus* (W)*, Nidula niveo-tomentosa* (W)*, Pholiota terrestris* (W)*, Pisolithus tinctorius* (ECM)*, Pluteus atricapillus* (W)*, Pluteus aurantiorugosus* (W)*, Pycnoporus coccineus* (W)*, Russula alboareolata* (ECM)*, Russula castanopsidis* (ECM)*, Russula cyanoxantha* (ECM)*, Suillus bovinus* (ECM)*, Suillus granulatus* (ECM)*, Suillus luteus* (ECM)*, Tyromyces chioneus* (W) |
| 15-Nov-87 | *Agaricus abruptibulbus* (L)*, Amanita citrina var. citrina* (ECM)*, Astraeus hygrometricus* (ECM)*, Coprinus atramentarius* (L)*, Cortinarius anomalus* (ECM)*, Cortinarius galeroides* (ECM)*, Cortinarius purpurascens* (ECM)*, Cortinarius vibratilis* (ECM)*, Entoloma staurosporum* (L)*, Gloeophyllum subferrugineum* (W)*, Gomphidius roseus* (ECM)*, Hymenochaete yasudai* (W)*, Hypholoma fasciculare* (W)*, Hypholoma sublateritium* (W)*, Laccaria bicolor* (ECM)*, Lactarius chrysorrheus* (ECM)*, Lentinula edodes* (W)*, Lenzites betulinus* (W)*, Leotia lubrica* (L)*, Lepista nuda* (L)*, Mycena pura* (W)*, Nidularia farcta* (W)*, Oligoporus caesius* (W)*, Phaeolepiota aurea* (W)*, Rhizopogon nigrescens* (ECM)*, Russula rosea* (ECM)*, Stereopsis burtianum* (W)*, Suillus bovinus* (ECM)*, Suillus granulatus* (ECM)*, Suillus luteus* (ECM)*, Tricholoma ustale* (ECM)*, Tyromyces chioneus* (W) |
| 6-Dec-87 | *Cortinarius purpurascens* (ECM)*, Cyclomyces fuscus* (W)*, Entoloma staurosporum* (L)*, Exdia uvapassa* (W)*, Hydnum repandum* (ECM)*, Hygrocybe aurantia* (L)*, Hymenogaster tener* (ECM)*, Hypholoma fasciculare* (W)*, Lactarius chrysorrheus* (ECM)*, Lactarius gracilis* (ECM)*, Laeticorticium roseocarneum* (W)*, Lenzites betulinus* (W)*, Lycoperdon spadiceum* (L)*, Merulius tremellosus* (W)*, Mycena galericulata* (W)*, Nidula niveo-tomentosa* (W)*, Nidularia farcta* (W)*, Pholiota terrestris* (W)*, Polyporus alveolarius* (W)*, Russula omiensis* (ECM)*, Russula rosea* (ECM)*, Suillus granulatus* (ECM)*, Trichaptum fuscoviolaceum* (W)*, Xylobolus annosus* (W) |
| 24-Jan-88 | *Coltricia cinnamomea* (ECM)*, Cryptoporus volvatus* (W)*, Cyclomyces fuscus* (W)*, Gloeophyllum sepiarium* (W)*, Hypholoma fasciculare* (W)*, Lentinula edodes* (W)*, Lenzites betulinus* (W)*, Lycoperdon perlatum* (L)*, Morganella pyriformis* (L)*, Mycena laevigata* (W)*, Nidula niveo-tomentosa* (W)*, Pleurotus ostreatus* (W)*, Polyporus alveolarius* (W)*, Psathyrella piluliformis* (W)*, Russula adusta* (ECM)*, Russula omiensis* (ECM) |
| 17-Apr-88 | *Caloscypha fulgens* (L)*, Ciborinia camelliae* (L)*, Cordyceps ophioglossoides (NA), Cortinarius subalboviolaceus* (ECM)*, Daedaleopsis styracina* (W)*, Entoloma staurosporum* (L)*, Ganoderma applanatum* (W)*, Hypholoma fasciculare* (W)*, Lentinula edodes* (W)*, Mycena alcalina* (W)*, Russula omiensis* (ECM)*, Russula rosea* (ECM)*, Trametes versicolor* (W) |
| 22-May-88 | *Amanita citrina var. grisea* (ECM)*, Coprinus atramentarius* (L)*, Cordyceps japonensis (NA), Cordyceps sobolifera (NA), Cortinarius subalboviolaceus* (ECM)*, Cryptoporus volvatus* (W)*, Entoloma papillatus* (L)*, Fistulina hepatica* (W)*, Hydnotrya tulasnei* (ECM)*, Hygrocybe aurantia* (L)*, Hypholoma fasciculare* (W)*, Lactarius gracilis* (ECM)*, Neolentinus lepideus* (W)*, Nidularia farcta* (W)*, Pluteus atricapillus* (W)*, Russula rosea* (ECM) |
| 19-Jun-88 | *Agaricus abruptibulbus* (L)*, Amanita ceciliae* (ECM)*, Amanita citrina var. grisea* (ECM)*, Amanita pantherina* (ECM)*, Amanita pseudoporphyria* (ECM)*, Astraeus hygrometricus* (ECM)*, Collybia butyracea* (L)*, Collybia neofusipes* (L)*, Coprinus atramentarius* (L)*, Cordyceps japonensis (NA), Cortinarius galeroides* (ECM)*, Cortinarius rubicundulus* (ECM)*, Cortinarius subalboviolaceus* (ECM)*, Cortinarius violaceus* (ECM)*, Cryptoporus volvatus* (W)*, Entoloma murraii* (L)*, Gymnopilus liquiritiae* (W)*, Hydnotrya tulasnei* (ECM)*, Hygrocybe cuspidata* (L)*, Inonotus vallatus* (W)*, Lactarius gracilis* (ECM)*, Lepiota pseudogranulosa* (L)*, Microporus vernicipes* (W)*, Neolentinus lepideus* (W)*, Nidula niveo-tomentosa* (W)*, Paxillus cutisii* (W)*, Polyporus alveolarius* (W)*, Psilocybe argentipes* (L)*, Russula alboareolata* (ECM)*, Russula amoena* (ECM)*, Russula mariae* (ECM)*, Russula pectinatoides* (ECM)*, Thelephora multipartita* (ECM)*, Tylopilus ferrugineus* (ECM)*, Xerula pudens* (W) |
| 23-Oct-88 | *Agrocybe erebia* (W)*, Amanita virosa* (ECM)*, Chroogomphus rutilus* (ECM)*, Collybia maculata* (L)*, Collybia neofusipes* (L)*, Coprinus atramentarius* (L)*, Cortinarius aureobrunneus* (ECM)*, Cortinarius purpurascens* (ECM)*, Cyclomyces fuscus* (W)*, Daedaleopsis purpurea* (W)*, Entoloma murraii* (L)*, Entoloma staurosporum* (L)*, Gerronema fibula* (L)*, Gomphidius roseus* (ECM)*, Gymnopilus liquiritiae* (W)*, Hygrocybe aurantia* (L)*, Inonotus vallatus* (W)*, Laccaria bicolor* (ECM)*, Lactarius chrysorrheus* (ECM)*, Laetiporus versisporus* (W)*, Lentinellus ursinus* (W)*, Lepista sordida* (L)*, Lyophyllum decastes* (L)*, Microporus affinis* (W)*, Morganella pyriformis* (L)*, Mycena galericulata* (W)*, Neolentinus lepideus* (W)*, Phaeolepiota aurea* (W)*, Polyporus alveolarius* (W)*, Psathyrella piluliformis* (W)*, Russula alboareolata* (ECM)*, Russula compacta* (ECM)*, Russula cyanoxantha* (ECM)*, Russula emetica* (ECM)*, Russula metachroa* (ECM)*, Scutellinia scutellata* (W)*, Suillus bovinus* (ECM)*, Suillus granulatus* (ECM) |
| 13-Nov-88 | *Amanita virosa* (ECM)*, Antrodiella zonata* (W)*, Astraeus hygrometricus* (ECM)*, Calostoma japonicum* (ECM)*, Cortinarius pseudopurpurascens* (ECM)*, Cortinarius purpurascens* (ECM)*, Daedaleopsis purpurea* (W)*, Entoloma staurosporum* (L)*, Gloeophyllum subferrugineum* (W)*, Grifola frondosa* (W)*, Hydnum repandum* (ECM)*, Hygrocybe aurantia* (L)*, Hymenochaete rubiginosa* (W)*, Hypholoma fasciculare* (W)*, Hypholoma sublateritium* (W)*, Laccaria amethystea* (ECM)*, Lactarius chrysorrheus* (ECM)*, Laetiporus versisporus* (W)*, Lentinula edodes* (W)*, Lenzites betulinus* (W)*, Leotia lubrica* (L)*, Loweporus pubertatis* (W)*, Microporus affinis* (W)*, Pluteus leoninus* (W)*, Psathyrella velutina* (W)*, Russula omiensis* (ECM)*, Russula vesca* (ECM)*, Scleroderma flavidum* (ECM)*, Scleroderma verrucosum* (ECM)*, Stereum gausapatum* (W)*, Suillus bovinus* (ECM)*, Suillus luteus* (ECM)*, Trametes versicolor* (W)*, Tricholoma ustale* (ECM)*, Tyromyces chioneus* (W) |
| 16-Jul-89 | *Agrocybe erebia* (W)*, Amanita longistriata* (ECM)*, Amanita pseudoporphyria* (ECM)*, Amanita spissacea* (ECM)*, Amanita spreta* (ECM)*, Amanita vaginata var. vaginata* (ECM)*, Auricularia polytricha* (W)*, Austroboletus subvirens* (ECM)*, Boletellus emodensis* (ECM)*, Boletus fraternus* (ECM)*, Calvatia craniiformis* (L)*, Cantharellus minor* (ECM)*, Clavulina cristata* (ECM)*, Clavulina rugosa* (ECM)*, Coltricia pusilla* (ECM)*, Cortinarius galeroides* (ECM)*, Cortinarius subalboviolaceus* (ECM)*, Daldinia concentrica* (W)*, Ganoderma applanatum* (W)*, Gerronema fibula* (L)*, Gyroporus castaneus* (ECM)*, Helvella elastica* (ECM)*, Helvella ephippioides* (ECM)*, Helvella macropus* (ECM)*, Hypholoma fasciculare* (W)*, Laccaria vinaceoavellanea* (ECM)*, Lactarius gerardii* (ECM)*, Lactarius gracilis* (ECM)*, Lactarius subvellereus* (ECM)*, Marasmius maximus* (L)*, Marasmius pulcherripes* (L)*, Marasmius purpureostriatus* (L)*, Microporus affinis* (W)*, Nomuraea atypicola (NA), Phylloporus bellus* (ECM)*, Polyporus alveolarius* (W)*, Polyporus brumalis* (W)*, Russula alboareolata* (ECM)*, Russula castanopsidis* (ECM)*, Russula mariae* (ECM)*, Russula nigricans* (ECM)*, Russula pectinatoides* (ECM)*, Russula senecis* (ECM)*, Russula virescens* (ECM)*, Trametes versicolor* (W)*, Trichaptum abietinum* (W)*, Xerocomus nigromaculatus* (ECM)*, Xerula pudens* (W) |
| 23-Jul-90 | *Amanita citrina var. grisea* (ECM)*, Amanita farinosa* (ECM)*, Amanita griseofarinosa* (ECM)*, Amanita pseudoporphyria* (ECM)*, Amanita rufoferruginea* (ECM)*, Amanita spissacea* (ECM)*, Amanita vaginata var. vaginata* (ECM)*, Amanita volvata* (ECM)*, Armillaria tabescens* (W)*, Austroboletus fusisporus* (ECM)*, Boletellus emodensis* (ECM)*, Boletus fraternus* (ECM)*, Boletus ornatipes* (ECM)*, Boletus violaceofuscus* (ECM)*, Cantharellus minor* (ECM)*, Clavulina cristata* (ECM)*, Cyclomyces fuscus* (W)*, Ganoderma applanatum* (W)*, Gymnopilus liquiritiae* (W)*, Gyroporus logicystidiatus* (ECM)*, Inocybe nodulosospora* (ECM)*, Laccaria vinaceoavellanea* (ECM)*, Lactarius gerardii* (ECM)*, Microporus affinis* (W)*, Nomuraea atypicola (NA), Perenniporia ochroleuca* (W)*, Phylloporus bellus* (ECM)*, Polyporus varius* (W)*, Psilocybe argentipes* (L)*, Pulveroboletus ravenelii* (ECM)*, Russula alboareolata* (ECM)*, Russula castanopsidis* (ECM)*, Russula cyanoxantha* (ECM)*, Russula eburneoareolata* (ECM)*, Russula japonica* (ECM)*, Russula lilacea* (ECM)*, Russula pectinatoides* (ECM)*, Russula senecis* (ECM)*, Russula vesca* (ECM)*, Sparassis crispa* (W)*, Trametes versicolor* (W)*, Tylopilus ballouii* (ECM)*, Tylopilus fumosipes* (ECM)*, Tylopilus nigropurpureus* (ECM)*, Xerocomus nigromaculatus* (ECM)*, Xerula radicata* (W)*, Xylobolus spectabilis* (W) |
| 28-Oct-90 | *Agrocybe erebia* (W)*, Amanita abrupta* (ECM)*, Amanita ceciliae* (ECM)*, Amanita citrina var. citrina* (ECM)*, Amanita fulva* (ECM)*, Amanita pseudoporphyria* (ECM)*, Amanita rufoferruginea* (ECM)*, Amanita vaginata var. punctata* (ECM)*, Amanita vaginata var. vaginata* (ECM)*, Amanita virgineoides* (ECM)*, Amanita virosa* (ECM)*, Amanita volvata* (ECM)*, Armillaria mellea* (W)*, Callistosporium luteoolivaceum* (L)*, Calostoma japonicum* (ECM)*, Calvatia craniiformis* (L)*, Clavulina rugosa* (ECM)*, Clitocybe fragrans* (L)*, Coltricia cinnamomea* (ECM)*, Coltricia pusilla* (ECM)*, Coprinus atramentarius* (L)*, Cortinarius galeroides* (ECM)*, Craterellus cornucopioides* (ECM)*, Cyclomyces fuscus* (W)*, Daedalea dickinsii* (W)*, Daedaleopsis purpurea* (W)*, Daedaleopsis tricolor* (W)*, Galerina sphagnorum* (L)*, Galiella celebica* (W)*, Ganoderma applanatum* (W)*, Gerronema fibula* (L)*, Gloeophyllum abietinum* (W)*, Gomphidius roseus* (ECM)*, Hebeloma crustuliniforme* (ECM)*, Helvella macropus* (ECM)*, Hygrocybe aurantia* (L)*, Hypholoma fasciculare* (W)*, Laccaria amethystea* (ECM)*, Laccaria bicolor* (ECM)*, Laccaria ohiensis* (ECM)*, Lactarius camphoratus* (ECM)*, Lactarius chrysorrheus* (ECM)*, Lactarius gracilis* (ECM)*, Lactarius lividatus* (ECM)*, Lactarius quietus* (ECM)*, Lentinellus ursinus* (W)*, Lycoperdon perlatum* (L)*, Lyophyllum decastes* (L)*, Macrolepiota procena* (L)*, Marasmius pulcherripes* (L)*, Mycena amygdalina* (W)*, Oligoporus caesius* (W)*, Pholiota highlandensis* (W)*, Pisolithus tinctorius* (ECM)*, Pleurotus ostreatus* (W)*, Psathyrella candolliana* (W)*, Psathyrella velutina* (W)*, Pseudocolus schellenbergiae* (L)*, Pycnoporus coccineus* (W)*, Russula alboareolata* (ECM)*, Russula castanopsidis* (ECM)*, Russula emetica* (ECM)*, Russula metachroa* (ECM)*, Russula pectinatoides* (ECM)*, Sarcodon scabrosus* (ECM)*, Scleroderma flavidum* (ECM)*, Scleroderma verrucosum* (ECM)*, Scutellinia scutellata* (W)*, Stereum gausapatum* (W)*, Suillus bovinus* (ECM)*, Suillus luteus* (ECM)*, Tremella foliacea* (W)*, Trichaptum biforme* (W)*, Tylopilus ballouii* (ECM) |
| 31-Mar-91 | *Auricularia auricula* (W)*, Caloscypha fulgens* (L)*, Coprinus micaceus* (W)*, Cryptoporus volvatus* (W)*, Entoloma staurosporum* (L)*, Flammulina velutipes* (W)*, Hypholoma fasciculare* (W)*, Lentinula edodes* (W)*, Lycoperdon perlatum* (L)*, Marasmiellus nigripes* (W)*, Morchella conica* (L)*, Perenniporia ochroleuca* (W)*, Pluteus atricapillus* (W)*, Psathyrella piluliformis* (W)*, Russula omiensis* (ECM)*, Tremella foliacea* (W)*, Tricholoma ustale* (ECM) |
| 21-Apr-91 | *Coprinus atramentarius* (L)*, Russula omiensis* (ECM) |
| 26-May-91 | *Amanita volvata* (ECM)*, Coprinus atramentarius* (L)*, Cortinarius subalboviolaceus* (ECM)*, Cortinarius violaceus* (ECM)*, Cryptoporus volvatus* (W)*, Cyclomyces fuscus* (W)*, Fistulina hepatica* (W)*, Galiella celebica* (W)*, Hygrocybe aurantia* (L)*, Lactarius gracilis* (ECM)*, Microporus affinis* (W)*, Neolentinus lepideus* (W)*, Oligoporus caesius* (W)*, Russula vesca* (ECM)*, Suillus granulatus* (ECM)*, Trichaptum abietinum* (W)*, Trichaptum biforme* (W)*, Trichaptum fuscoviolaceum* (W) |
| 30-Jun-91 | *Agaricus abruptibulbus* (L)*, Amanita citrina var. citrina* (ECM)*, Amanita excelsa* (ECM)*, Amanita pantherina* (ECM)*, Amanita pseudoporphyria* (ECM)*, Amanita rubescens* (ECM)*, Amanita spissacea* (ECM)*, Amanita sychnopyramis f. subannulata* (ECM)*, Amanita vaginata var. vaginata* (ECM)*, Bjerkandera adusta* (W)*, Boletus aokii* (ECM)*, Boletus reticulatus* (ECM)*, Callistosporium luteoolivaceum* (L)*, Clavulina rugosa* (ECM)*, Collybia neofusipes* (L)*, Coltricia cinnamomea* (ECM)*, Coltricia pusilla* (ECM)*, Cordyceps annullata (NA), Cortinarius galeroides* (ECM)*, Cortinarius subalboviolaceus* (ECM)*, Cryptoporus volvatus* (W)*, Cyclomyces fuscus* (W)*, Dicephalospora rufocornea* (W)*, Entoloma murraii* (L)*, Gyroporus castaneus* (ECM)*, Helvella elastica* (ECM)*, Hygrocybe cuspidata* (L)*, Hypholoma fasciculare* (W)*, Inocybe lutea* (ECM)*, Laccaria vinaceoavellanea* (ECM)*, Lactarius gerardii* (ECM)*, Lactarius gracilis* (ECM)*, Leccinum extremiorientale* (ECM)*, Megacollybia platyphylla* (W)*, Microporus affinis* (W)*, Nomuraea atypicola (NA), Omphalina epichysium* (L)*, Perenniporia ochroleuca* (W)*, Phylloporus bellus* (ECM)*, Psathyrella candolliana* (W)*, Pseudmerulius aureus* (W)*, Ramariopsis fusiformis* (ECM)*, Russula alboareolata* (ECM)*, Russula castanopsidis* (ECM)*, Russula lilacea* (ECM)*, Russula mariae* (ECM)*, Russula senecis* (ECM)*, Russula vesca* (ECM)*, Russula violeipes* (ECM)*, Russula viridirubrolimbata* (ECM)*, Stereum gausapatum* (W)*, Strobilomyces confusus* (ECM)*, Tremella mesenterica* (W)*, Trichaptum abietinum* (W)*, Trichocoma paradoxa* (W)*, Tylopilus ballouii* (ECM)*, Tylopilus fumosipes* (ECM)*, Veluticeps berkeleyi* (W)*, Volvariella subtaylori* (L)*, Xerocomus nigromaculatus* (ECM)*, Xerocomus parvulus* (ECM)*, Xerocomus subtomentosus* (ECM)*, Xeromphalina campanella* (W)*, Xerula pudens* (W)*, Xerula radicata* (W) |
| 21-Jul-91 | *Amanita fuliginea* (ECM)*, Amanita griseofarinosa* (ECM)*, Amanita pseudoporphyria* (ECM)*, Amanita spissacea* (ECM)*, Amanita sychnopyramis f. subannulata* (ECM)*, Amanita vaginata var. vaginata* (ECM)*, Amanita virosa* (ECM)*, Astraeus hygrometricus* (ECM)*, Boletellus emodensis* (ECM)*, Boletus fraternus* (ECM)*, Boletus granulopunctatus* (ECM)*, Boletus ornatipes* (ECM)*, Cortinarius galeroides* (ECM)*, Crepidotus mollis* (W)*, Cryptoporus volvatus* (W)*, Cyclomyces fuscus* (W)*, Ganoderma applanatum* (W)*, Gerronema fibula* (L)*, Hygrocybe conica* (L)*, Hygrocybe cuspidata* (L)*, Laccaria bicolor* (ECM)*, Lactarius corrugis* (ECM)*, Lactarius piperatus* (ECM)*, Lactarius vellereus* (ECM)*, Leccinum extremiorientale* (ECM)*, Microporus affinis* (W)*, Nomuraea atypicola (NA), Onnia scaura* (W)*, Paxillus atrotomentosus* (W)*, Perenniporia fraxinea* (W)*, Phylloporus bellus* (ECM)*, Pluteus leoninus* (W)*, Psathyrella piluliformis* (W)*, Pulveroboletus ravenelii* (ECM)*, Radulodon capelandii* (W)*, Ramariopsis fusiformis* (ECM)*, Russula alboareolata* (ECM)*, Russula castanopsidis* (ECM)*, Russula densifolia* (ECM)*, Russula japonica* (ECM)*, Russula kansaiensis* (ECM)*, Russula mariae* (ECM)*, Russula rosacea* (ECM)*, Russula rubescens* (ECM)*, Russula senecis* (ECM)*, Stereum hirsutum* (W)*, Trametes versicolor* (W)*, Tremella fuciformis* (W)*, Tremella iduensis* (W)*, Tylopilus ballouii* (ECM)*, Tylopilus fumosipes* (ECM)*, Tylopilus otsuensis* (ECM)*, Xerocomus chrysenteron* (ECM)*, Xerocomus nigromaculatus* (ECM)*, Xerula pudens* (W) |
| 29-Sep-91 | *Agaricus abruptibulbus* (L)*, Agaricus subrutilescens* (L)*, Agrocybe cylindracea* (W)*, Amanita ceciliae* (ECM)*, Amanita farinosa* (ECM)*, Amanita fuliginea* (ECM)*, Amanita fulva* (ECM)*, Amanita pantherina* (ECM)*, Amanita vaginata var. punctata* (ECM)*, Antrodiella zonata* (W)*, Auricularia polytricha* (W)*, Austroboletus fusisporus* (ECM)*, Austroboletus subvirens* (ECM)*, Boletellus emodensis* (ECM)*, Callistosporium luteoolivaceum* (L)*, Calostoma japonicum* (ECM)*, Collybia dryophila* (L)*, Coprinus atramentarius* (L)*, Coprinus disseminatus* (W)*, Cyclomyces fuscus* (W)*, Cyptotrama asprata* (W)*, Daedaleopsis tricolor* (W)*, Dicephalospora rufocornea* (W)*, Entoloma bisporus* (L)*, Entoloma sericellum* (L)*, Galiella celebica* (W)*, Gymnopilus liquiritiae* (W)*, Helvella crispa* (ECM)*, Hypholoma fasciculare* (W)*, Laccaria vinaceoavellanea* (ECM)*, Lenzites betulinus* (W)*, Lepiota atrosquamulosa* (L)*, Lepiota pseudogranulosa* (L)*, Loweporus pubertatis* (W)*, Lycoperdon perlatum* (L)*, Lyophyllum decastes* (L)*, Megacollybia platyphylla* (W)*, Melanogaster intermedius* (ECM)*, Mycena haematopoda* (W)*, Neolentinus lepideus* (W)*, Nidularia farcta* (W)*, Oligoporus caesius* (W)*, Paxillus atrotomentosus* (W)*, Perenniporia ochroleuca* (W)*, Phallus impudicus* (L)*, Phanerochaete crossa* (W)*, Pholiota terrestris* (W)*, Pleurotus pulmonarius* (W)*, Pluteus atricapillus* (W)*, Pseudmerulius aureus* (W)*, Russula cyanoxantha* (ECM)*, Russula laurocerasi* (ECM)*, Russula mariae* (ECM)*, Russula rosacea* (ECM)*, Scleroderma areolatum* (ECM)*, Sparassis crispa* (W)*, Stereum gausapatum* (W)*, Stereum hirsutum* (W)*, Trametes versicolor* (W)*, Tyromyces chioneus* (W)*, Xerocomus nigromaculatus* (ECM)*, Xeromphalina campanella* (W)*, Xylobolus spectabilis* (W) |
| 27-Oct-91 | *Amanita citrina var. citrina* (ECM)*, Amanita ibotengutake* (ECM)*, Amanita pseudoporphyria* (ECM)*, Amanita vaginata var. vaginata* (ECM)*, Amanita virosa* (ECM)*, Antrodiella zonata* (W)*, Bjerkandera adusta* (W)*, Calostoma japonicum* (ECM)*, Clitocybe fragrans* (L)*, Collybia dryophila* (L)*, Collybia neofusipes* (L)*, Coprinus atramentarius* (L)*, Cortinarius azureus* (ECM)*, Cortinarius salor* (ECM)*, Crepidotus mollis* (W)*, Cyptotrama asprata* (W)*, Daedaleopsis purpurea* (W)*, Daedaleopsis styracina* (W)*, Entoloma papillatus* (L)*, Exdia uvapassa* (W)*, Fistulina hepatica* (W)*, Fomitopsis pinicola* (W)*, Gloeophyllum subferrugineum* (W)*, Gymnopilus liquiritiae* (W)*, Helvella ephippioides* (ECM)*, Hydnum repandum* (ECM)*, Hypholoma fasciculare* (W)*, Inonotus mikadoi* (W)*, Laccaria bicolor* (ECM)*, Lactarius akahatsu* (ECM)*, Lactarius chrysorrheus* (ECM)*, Lactarius gracilis* (ECM)*, Lactarius subzonarius* (ECM)*, Lentinula edodes* (W)*, Lenzites betulinus* (W)*, Leotia lubrica* (L)*, Leucogyrophana mollusca* (W)*, Lycoperdon perlatum* (L)*, Lycoperdon spadiceum* (L)*, Lyophyllum decastes* (L)*, Mycena pura* (W)*, Oligoporus caesius* (W)*, Phaeolepiota aurea* (W)*, Phallus impudicus* (L)*, Porodisculus pendulus* (W)*, Russula alboareolata* (ECM)*, Russula castanopsidis* (ECM)*, Russula cyanoxantha* (ECM)*, Russula japonica* (ECM)*, Russula kansaiensis* (ECM)*, Russula mariae* (ECM)*, Russula vesca* (ECM)*, Stereum gausapatum* (W)*, Suillus granulatus* (ECM)*, Suillus luteus* (ECM)*, Tremella mesenterica* (W)*, Trichaptum abietinum* (W) |
| 17-Nov-91 | *Calostoma japonicum* (ECM)*, Coprinus atramentarius* (L)*, Cyclomyces fuscus* (W)*, Fistulina hepatica* (W)*, Gymnopilus liquiritiae* (W)*, Hygrocybe aurantia* (L)*, Hymenochaete intricatae* (W)*, Hypholoma fasciculare* (W)*, Hypholoma sublateritium* (W)*, Lentinula edodes* (W)*, Lenzites betulinus* (W)*, Leotia lubrica* (L)*, Lycoperdon perlatum* (L)*, Lyophyllum decastes* (L)*, Merulius tremellosus* (W)*, Microporus affinis* (W)*, Oligoporus caesius* (W)*, Russula vesca* (ECM)*, Scleroderma verrucosum* (ECM)*, Stereopsis burtianum* (W)*, Stereum gausapatum* (W)*, Trametes hirsuta* (W)*, Trametes versicolor* (W)*, Xylaria polymorpha* (W) |
| 8-Dec-91 | *Auricularia polytricha* (W)*, Calostoma japonicum* (ECM)*, Craterellus cornucopioides* (ECM)*, Cyclomyces fuscus* (W)*, Daedaleopsis purpurea* (W)*, Entoloma staurosporum* (L)*, Flammulina velutipes* (W)*, Galerina pseudocamerina* (L)*, Gloeophyllum sepiarium* (W)*, Hypholoma fasciculare* (W)*, Hypholoma sublateritium* (W)*, Hypoxylon howeianum* (W)*, Inonotus mikadoi* (W)*, Lentinula edodes* (W)*, Lenzites betulinus* (W)*, Leotia lubrica* (L)*, Lycoperdon spadiceum* (L)*, Lyophyllum decastes* (L)*, Microporus affinis* (W)*, Microporus vernicipes* (W)*, Mycena haematopoda* (W)*, Mycena polygramma* (W)*, Neolentinus lepideus* (W)*, Nidula niveo-tomentosa* (W)*, Nidularia farcta* (W)*, Phanerochaete crossa* (W)*, Polyporus badius* (W)*, Russula omiensis* (ECM)*, Russula rosea* (ECM)*, Stereopsis burtianum* (W)*, Stereum gausapatum* (W)*, Strobilurus ohshimae* (W)*, Strobilurus stephanocystis (NA), Trametes versicolor* (W) |
| 23-Feb-92 | *Calostoma japonicum* (ECM)*, Craterellus cornucopioides* (ECM)*, Cyclomyces fuscus* (W)*, Entoloma staurosporum* (L)*, Flammulina velutipes* (W)*, Fomitopsis pinicola* (W)*, Hypholoma fasciculare* (W)*, Inonotus xeranticus* (W)*, Lentinula edodes* (W)*, Lenzites betulinus* (W)*, Psathyrella piluliformis* (W)*, Punctularia strigosozonata* (W)*, Pycnoporus coccineus* (W)*, Russula omiensis* (ECM)*, Strobilurus stephanocystis (NA), Trametes hirsuta* (W)*, Trametes orientalis* (W)*, Tremella fimbriata* (W) |
| 29-Mar-92 | *Cryptoporus volvatus* (W)*, Entoloma staurosporum* (L)*, Exdia glandulosa* (W)*, Hypholoma fasciculare* (W)*, Hypoxylon howeianum* (W)*, Lambertella brunneola* (L)*, Lentinula edodes* (W)*, Lenzites betulinus* (W)*, Panellus stypticus* (W)*, Russula omiensis* (ECM)*, Stereum gausapatum* (W)*, Tremella mesenterica* (W)*, Trichocoma paradoxa* (W) |
| 31-May-92 | *Agrocybe erebia* (W)*, Amanita citrina var. grisea* (ECM)*, Amanita spissacea* (ECM)*, Auricularia auricula* (W)*, Collybia dryophila* (L)*, Coprinus atramentarius* (L)*, Coprinus disseminatus* (W)*, Cordyceps annullata (NA), Cordyceps ormicarum (NA), Cortinarius galeroides* (ECM)*, Cortinarius subalboviolaceus* (ECM)*, Cortinarius violaceus* (ECM)*, Cryptoporus volvatus* (W)*, Entoloma staurosporum* (L)*, Fistulina hepatica* (W)*, Gymnopilus liquiritiae* (W)*, Hydnotrya tulasnei* (ECM)*, Hygrocybe aurantia* (L)*, Hypholoma fasciculare* (W)*, Inonotus mikadoi* (W)*, Laccaria bicolor* (ECM)*, Lactarius gracilis* (ECM)*, Lentinula edodes* (W)*, Lenzites betulinus* (W)*, Microporus affinis* (W)*, Neolentinus lepideus* (W)*, Panellus stypticus* (W)*, Pholiota terrestris* (W)*, Pluteus atricapillus* (W)*, Russula cyanoxantha* (ECM)*, Russula emetica* (ECM)*, Russula kansaiensis* (ECM)*, Russula omiensis* (ECM)*, Russula rosea* (ECM)*, Scutellinia scutellata* (W)*, Tremella mesenterica* (W)*, Xerula pudens* (W) |
| 28-Jun-92 | *Astraeus hygrometricus* (ECM)*, Calocera cornea* (W)*, Clavulina cristata* (ECM)*, Coprinus atramentarius* (L)*, Coprinus disseminatus* (W)*, Cordyceps annullata (NA), Cortinarius galeroides* (ECM)*, Cortinarius subalboviolaceus* (ECM)*, Cortinarius violaceus* (ECM)*, Craterellus cornucopioides* (ECM)*, Dicephalospora rufocornea* (W)*, Entoloma staurosporum* (L)*, Fistulina hepatica* (W)*, Ganoderma applanatum* (W)*, Gymnopilus liquiritiae* (W)*, Helvella ephippioides* (ECM)*, Hygrocybe cuspidata* (L)*, Lepiota pseudogranulosa* (L)*, Marasmius maximus* (L)*, Megacollybia platyphylla* (W)*, Microporus affinis* (W)*, Perenniporia ochroleuca* (W)*, Polyporus arcularius* (W)*, Psathyrella spadiceogrisea* (W)*, Russula alboareolata* (ECM)*, Russula cyanoxantha* (ECM)*, Russula mariae* (ECM)*, Tylopilus chromapes* (ECM)*, Tylopilus rigens* (ECM) |
| 30-Aug-92 | *Agaricus praeclaresquamosus* (L)*, Agaricus subrutilescens* (L)*, Agrocybe cylindracea* (W)*, Amanita fuliginea* (ECM)*, Amanita perpasta* (ECM)*, Amanita porphyria* (ECM)*, Amanita pseudoporphyria* (ECM)*, Amanita spissacea* (ECM)*, Amanita sychnopyramis f. subannulata* (ECM)*, Amanita vaginata var. vaginata* (ECM)*, Amanita virgineoides* (ECM)*, Amanita virosa* (ECM)*, Amanita volvata* (ECM)*, Armillaria tabescens* (W)*, Aureoboletus thibetanus* (ECM)*, Austroboletus fusisporus* (ECM)*, Austroboletus subvirens* (ECM)*, Boletellus emodensis* (ECM)*, Boletus erythropus* (ECM)*, Boletus fraternus* (ECM)*, Boletus griseus* (ECM)*, Boletus obscureumbrinus* (ECM)*, Boletus ornatipes* (ECM)*, Boletus umbriniporus* (ECM)*, Boletus violaceofuscus* (ECM)*, Coltricia pusilla* (ECM)*, Cortinarius rubicundulus* (ECM)*, Crepidotus mollis* (W)*, Cyclomyces fuscus* (W)*, Entoloma murraii* (L)*, Gyroporus castaneus* (ECM)*, Heimiella japonica* (ECM)*, Inocybe lutea* (ECM)*, Leccinum extremiorientale* (ECM)*, Leccinum hortonii* (ECM)*, Lenzites betulinus* (W)*, Lepiota cygnea* (L)*, Microporus affinis* (W)*, Nomuraea atypicola (NA), Paxillus atrotomentosus* (W)*, Phylloporus bellus* (ECM)*, Pisolithus tinctorius* (ECM)*, Pulveroboletus ravenelii* (ECM)*, Russula alboareolata* (ECM)*, Russula amoena* (ECM)*, Russula peltereaui* (ECM)*, Russula subnigricans* (ECM)*, Strobilomyces confusus* (ECM)*, Strobilomyces seminudus* (ECM)*, Tylopilus ferrugineus* (ECM)*, Tylopilus fumosipes* (ECM)*, Xerocomus nigromaculatus* (ECM)*, Xerula radicata* (W) |
| 27-Sep-92 | *Agrocybe cylindracea* (W)*, Antrodiella zonata* (W)*, Astraeus hygrometricus* (ECM)*, Calostoma japonicum* (ECM)*, Cortinarius rubicundulus* (ECM)*, Cryptoporus volvatus* (W)*, Cyclomyces fuscus* (W)*, Daedaleopsis styracina* (W)*, Galerina fasciculata* (W)*, Ganoderma applanatum* (W)*, Gloeophyllum subferrugineum* (W)*, Gymnopilus liquiritiae* (W)*, Inonotus mikadoi* (W)*, Inonotus vallatus* (W)*, Lenzites betulinus* (W)*, Loweporus pubertatis* (W)*, Lyophyllum decastes* (L)*, Mycena haematopoda* (W)*, Nidula niveo-tomentosa* (W)*, Perenniporia ochroleuca* (W)*, Phallus impudicus* (L)*, Pholiota highlandensis* (W)*, Psathyrella piluliformis* (W)*, Russula castanopsidis* (ECM)*, Russula rosacea* (ECM)*, Russula vesca* (ECM)*, Stereum hirsutum* (W)*, Trametes versicolor* (W)*, Trichaptum abietinum* (W)*, Tyromyces chioneus* (W)*, Xeromphalina campanella* (W)*, Xylobolus spectabilis* (W) |
| 6-Dec-92 | *Auricularia polytricha* (W)*, Baeospora myosura (NA), Calostoma japonicum* (ECM)*, Coltricia pusilla* (ECM)*, Coprinus atramentarius* (L)*, Craterellus cornucopioides* (ECM)*, Cyclomyces fuscus* (W)*, Entoloma staurosporum* (L)*, Flammulina velutipes* (W)*, Hygrocybe aurantia* (L)*, Hypholoma fasciculare* (W)*, Lactarius chrysorrheus* (ECM)*, Lentinula edodes* (W)*, Lenzites betulinus* (W)*, Lepista nuda* (L)*, Lycoperdon bispinosum* (L)*, Lyophyllum decastes* (L)*, Microporus affinis* (W)*, Mycena polygramma* (W)*, Pholiota highlandensis* (W)*, Pholiota terrestris* (W)*, Polyporus alveolarius* (W)*, Psathyrella piluliformis* (W)*, Resupinatus trichotis* (W)*, Russula omiensis* (ECM)*, Schizophyllum commune* (W)*, Scleroderma areolatum* (ECM)*, Strobilurus stephanocystis (NA), Suillus granulatus* (ECM)*, Trametes versicolor* (W)*, Trichaptum abietinum* (W)*, Trichaptum biforme* (W) |
| 28-Feb-93 | *Antrodiella zonata* (W)*, Auricularia auricula* (W)*, Cordyceps ormicarum (NA), Cyclomyces fuscus* (W)*, Daedaleopsis styracina* (W)*, Exdia uvapassa* (W)*, Flammulina velutipes* (W)*, Fomitopsis pinicola* (W)*, Hydnochaete tabacinoides* (W)*, Hypholoma fasciculare* (W)*, Hypoxylon howeianum* (W)*, Irpex lacteus* (W)*, Lentinula edodes* (W)*, Lenzites betulinus* (W)*, Lycoperdon spadiceum* (L)*, Microporus affinis* (W)*, Mycena laevigata* (W)*, Nidula niveo-tomentosa* (W)*, Panellus stypticus* (W)*, Perenniporia ochroleuca* (W)*, Pleurotus ostreatus* (W)*, Polyporus alveolarius* (W)*, Psathyrella piluliformis* (W)*, Pycnoporus coccineus* (W)*, Resupinatus trichotis* (W)*, Russula omiensis* (ECM)*, Skeletocutis nivea* (W)*, Stereum gausapatum* (W)*, Stereum hirsutum* (W)*, Trametes versicolor* (W)*, Tremella mesenterica* (W)*, Trichaptum biforme* (W)*, Xylobolus annosus* (W) |
| 28-Mar-93 | *Ciborinia camelliae* (L)*, Coltricia pusilla* (ECM)*, Cryptoporus volvatus* (W)*, Daedaleopsis styracina* (W)*, Entoloma staurosporum* (L)*, Exdia glandulosa* (W)*, Flammulina velutipes* (W)*, Hypholoma fasciculare* (W)*, Lentinula edodes* (W)*, Microporus affinis* (W)*, Morchella conica* (L)*, Mycena alcalina* (W)*, Mycena amygdalina* (W)*, Panellus stypticus* (W)*, Russula omiensis* (ECM)*, Schizophyllum commune* (W)*, Stereum hirsutum* (W)*, Trametes versicolor* (W)*, Trichaptum abietinum* (W)*, Tricholoma ustale* (ECM) |
| 30-May-93 | *Amanita citrina var. grisea* (ECM)*, Antrodiella gypsea* (W)*, Auricularia auricula* (W)*, Auricularia polytricha* (W)*, Coprinus disseminatus* (W)*, Cordyceps annullata (NA), Cordyceps heteropoda (NA), Cordyceps ormicarum (NA), Cordyceps prolifica (NA), Cordyceps sobolifera (NA), Cortinarius violaceus* (ECM)*, Craterellus cornucopioides* (ECM)*, Cryptoporus volvatus* (W)*, Entoloma staurosporum* (L)*, Galiella celebica* (W)*, Hygrocybe aurantia* (L)*, Hypholoma fasciculare* (W)*, Lactarius gracilis* (ECM)*, Lentinula edodes* (W)*, Marasmius purpureostriatus* (L)*, Neolentinus lepideus* (W)*, Pholiota terrestris* (W)*, Polyporus arcularius* (W)*, Russula omiensis* (ECM)*, Russula vesca* (ECM)*, Stereum hirsutum* (W)*, Tremella mesenterica* (W)*, Trichaptum biforme* (W) |
| 27-Jun-93 | *Agaricus subrutilescens* (L)*, Agrocybe cylindracea* (W)*, Amanita excelsa* (ECM)*, Amanita farinosa* (ECM)*, Amanita pantherina* (ECM)*, Amanita rubescens* (ECM)*, Artomyces pyxidatus* (W)*, Astraeus hygrometricus* (ECM)*, Auricularia polytricha* (W)*, Austroboletus gracilis* (ECM)*, Boletus fraternus* (ECM)*, Boletus subvelutipes* (ECM)*, Bovista plumbea* (L)*, Callistosporium luteoolivaceum* (L)*, Calvatia craniiformis* (L)*, Chalciporus piperatus* (ECM)*, Clavulina cristata* (ECM)*, Collybia butyracea* (L)*, Collybia confluens* (L)*, Coltricia cinnamomea* (ECM)*, Coprinus plicatilis* (L)*, Cordyceps annullata (NA), Cordyceps sphecocephala (NA), Cortinarius galeroides* (ECM)*, Cortinarius subalboviolaceus* (ECM)*, Cryptoporus volvatus* (W)*, Entoloma murraii* (L)*, Gymnopilus aeruginosus* (W)*, Gyroporus castaneus* (ECM)*, Hebeloma vinosophyllum* (ECM)*, Helvella lacunosa* (ECM)*, Hygrocybe aurantia* (L)*, Hygrocybe conica* (L)*, Hypholoma fasciculare* (W)*, Inocybe asterospora* (ECM)*, Inocybe kobayasii* (ECM)*, Inocybe lacera* (ECM)*, Lactarius gerardii* (ECM)*, Lactarius gracilis* (ECM)*, Lepiota praetervisa* (L)*, Lepista sordida* (L)*, Lyophyllum decastes* (L)*, Marasmius maximus* (L)*, Marasmius siccus* (L)*, Megacollybia platyphylla* (W)*, Microporus affinis* (W)*, Nomuraea atypicola (NA), Omphalina epichysium* (L)*, Phylloporus bellus* (ECM)*, Pluteus atricapillus* (W)*, Psathyrella obutusata* (W)*, Psilocybe argentipes* (L)*, Russula alboareolata* (ECM)*, Russula compacta* (ECM)*, Russula mariae* (ECM)*, Russula sororia* (ECM)*, Russula violeipes* (ECM)*, Scleroderma areolatum* (ECM)*, Suillus granulatus* (ECM)*, Xerocomus parvulus* (ECM)*, Xerula pudens* (W) |
| 18-Jul-93 | *Amanita citrina var. grisea* (ECM)*, Amanita farinosa* (ECM)*, Amanita fuliginea* (ECM)*, Amanita fulva* (ECM)*, Amanita longistriata* (ECM)*, Amanita pantherina* (ECM)*, Amanita pseudoporphyria* (ECM)*, Amanita rubescens* (ECM)*, Amanita spissacea* (ECM)*, Amanita sychnopyramis f. subannulata* (ECM)*, Armillaria tabescens* (W)*, Artomyces pyxidatus* (W)*, Astraeus hygrometricus* (ECM)*, Auricularia polytricha* (W)*, Boletus fraternus* (ECM)*, Cantharellus minor* (ECM)*, Clavulina amethystinoides* (ECM)*, Clavulina cristata* (ECM)*, Clavulina rugosa* (ECM)*, Collybia dryophila* (L)*, Coprinus atramentarius* (L)*, Cordyceps annullata (NA), Cortinarius galeroides* (ECM)*, Cortinarius subalboviolaceus* (ECM)*, Cryptoporus volvatus* (W)*, Cyclomyces fuscus* (W)*, Dicephalospora rufocornea* (W)*, Entoloma murraii* (L)*, Gymnopilus liquiritiae* (W)*, Gyroporus castaneus* (ECM)*, Helvella elastica* (ECM)*, Inocybe lutea* (ECM)*, Inonotus mikadoi* (W)*, Laccaria amethystea* (ECM)*, Laccaria laccata* (ECM)*, Laccaria vinaceoavellanea* (ECM)*, Lactarius quietus* (ECM)*, Lactarius subvellereus* (ECM)*, Lentinula edodes* (W)*, Lepiota pseudogranulosa* (L)*, Microporus affinis* (W)*, Neolentinus lepideus* (W)*, Nomuraea atypicola (NA), Phylloporus bellus* (ECM)*, Resupinatus applicatus* (W)*, Russula castanopsidis* (ECM)*, Russula cyanoxantha* (ECM)*, Russula japonica* (ECM)*, Russula nigricans* (ECM)*, Russula pectinatoides* (ECM)*, Russula violeipes* (ECM)*, Stereum hirsutum* (W)*, Strobilomyces confusus* (ECM)*, Suillus granulatus* (ECM)*, Tremella mesenterica* (W)*, Trichoglossum walteri* (L)*, Tylopilus valens* (ECM)*, Xerula radicata* (W) |
| 21-Nov-93 | *Coltricia pusilla* (ECM)*, Coprinus atramentarius* (L)*, Cyclomyces fuscus* (W)*, Entoloma staurosporum* (L)*, Geastrum triplex* (L)*, Hygrocybe aurantia* (L)*, Hypholoma fasciculare* (W)*, Hypholoma sublateritium* (W)*, Lyophyllum decastes* (L)*, Merulius tremellosus* (W)*, Mycena haematopoda* (W)*, Mycena polygramma* (W)*, Pluteus leoninus* (W)*, Russula cyanoxantha* (ECM)*, Russula emetica* (ECM)*, Russula omiensis* (ECM)*, Suillus luteus* (ECM)*, Trametes versicolor* (W)*, Trichocoma paradoxa* (W) |
| 27-Feb-94 | *Auricularia polytricha* (W)*, Calostoma japonicum* (ECM)*, Ciborinia camelliae* (L)*, Cordyceps annullata (NA), Cyclomyces fuscus* (W)*, Daedalea dickinsii* (W)*, Daedaleopsis styracina* (W)*, Daldinia concentrica* (W)*, Flammulina velutipes* (W)*, Gloeophyllum subferrugineum* (W)*, Inonotus mikadoi* (W)*, Lenzites betulinus* (W)*, Morchella conica* (L)*, Mycena laevigata* (W)*, Panellus stypticus* (W)*, Pleurotus ostreatus* (W)*, Russula omiensis* (ECM)*, Trametes versicolor* (W)*, Tremella mesenterica* (W)*, Trichaptum abietinum* (W)*, Trichaptum biforme* (W) |
| 27-Mar-94 | *Calvatia craniiformis* (L)*, Ciborinia camelliae* (L)*, Cordyceps ophioglossoides (NA), Daedaleopsis styracina* (W)*, Elaphocordyceps longisegmentis (NA), Entoloma staurosporum* (L)*, Exdia glandulosa* (W)*, Fomitopsis pinicola* (W)*, Hypholoma fasciculare* (W)*, Lentinula edodes* (W)*, Morchella conica* (L)*, Pleurotus ostreatus* (W)*, Russula omiensis* (ECM)*, Trichaptum biforme* (W) |
| 26-Jun-94 | *Agaricus abruptibulbus* (L)*, Agrocybe cylindracea* (W)*, Agrocybe erebia* (W)*, Amanita farinosa* (ECM)*, Astraeus hygrometricus* (ECM)*, Auricularia polytricha* (W)*, Boletus fraternus* (ECM)*, Clavulina rugosa* (ECM)*, Collybia peronata* (L)*, Coprinus atramentarius* (L)*, Coprinus plicatilis* (L)*, Cordyceps annullata (NA), Cortinarius alboviolaceus* (ECM)*, Cortinarius galeroides* (ECM)*, Craterellus cornucopioides* (ECM)*, Crepidotus badiofloccosus* (W)*, Cyclomyces fuscus* (W)*, Entoloma murraii* (L)*, Fistulina hepatica* (W)*, Gyroporus castaneus* (ECM)*, Hygrocybe aurantia* (L)*, Hygrocybe cuspidata* (L)*, Hygrocybe nitida* (L)*, Inocybe asterospora* (ECM)*, Laccaria vinaceoavellanea* (ECM)*, Lactarius camphoratus* (ECM)*, Lactarius gracilis* (ECM)*, Laetiporus versisporus* (W)*, Lenzites betulinus* (W)*, Microporus affinis* (W)*, Mycena haematopoda* (W)*, Mycena stylobates* (W)*, Neolentinus lepideus* (W)*, Nidula niveo-tomentosa* (W)*, Phylloporus bellus* (ECM)*, Pleurotus ostreatus* (W)*, Pluteus aurantiorugosus* (W)*, Polyporus alveolarius* (W)*, Psathyrella candolliana* (W)*, Psathyrella velutina* (W)*, Psilocybe argentipes* (L)*, Ramariopsis fusiformis* (ECM)*, Russula cyanoxantha* (ECM)*, Russula kansaiensis* (ECM)*, Russula mariae* (ECM)*, Russula pectinatoides* (ECM)*, Russula sororia* (ECM)*, Russula violeipes* (ECM)*, Stereum hirsutum* (W)*, Thelephora aurantiotincta* (ECM)*, Tremella foliacea* (W)*, Trichaptum abietinum* (W)*, Trichocoma paradoxa* (W)*, Xerocomus chrysenteron* (ECM)*, Xerocomus subtomentosus* (ECM)*, Xeromphalina campanella* (W)*, Xerula radicata* (W) |
| 31-Jul-94 | *Agrocybe cylindracea* (W)*, Amanita citrina var. grisea* (ECM)*, Amanita cokeri* (ECM)*, Amanita fuliginea* (ECM)*, Amanita virosa* (ECM)*, Amanita volvata* (ECM)*, Astraeus hygrometricus* (ECM)*, Boletellus emodensis* (ECM)*, Boletellus obscurecoccineus* (ECM)*, Boletus fraternus* (ECM)*, Boletus griseus* (ECM)*, Cordyceps annullata (NA), Crepidotus mollis* (W)*, Ganoderma applanatum* (W)*, Gloeophyllum subferrugineum* (W)*, Inocybe lutea* (ECM)*, Laccaria vinaceoavellanea* (ECM)*, Lactarius gerardii* (ECM)*, Lactarius piperatus* (ECM)*, Laetiporus versisporus* (W)*, Leccinum intusrubens* (ECM)*, Loweporus tepbroporus* (W)*, Neolentinus lepideus* (W)*, Nomuraea atypicola (NA), Oligoporus caesius* (W)*, Perenniporia ochroleuca* (W)*, Psathyrella candolliana* (W)*, Russula densifolia* (ECM)*, Russula japonica* (ECM)*, Russula mariae* (ECM)*, Russula ochroleuca* (ECM)*, Russula subnigricans* (ECM)*, Russula vesca* (ECM)*, Tylopilus ferrugineus* (ECM)*, Tylopilus vinosobrunneus* (ECM)*, Tyromyces chioneus* (W)*, Xeromphalina campanella* (W) |
| 28-Aug-94 | *Agaricus praeclaresquamosus* (L)*, Agrocybe cylindracea* (W)*, Auricularia auricula* (W)*, Auricularia polytricha* (W)*, Boletus fraternus* (ECM)*, Calostoma japonicum* (ECM)*, Calvatia craniiformis* (L)*, Coprinus disseminatus* (W)*, Cryptoporus volvatus* (W)*, Lepiota clypeolaria* (L)*, Lepiota cygnea* (L)*, Leucoagaricus rubrotinctus* (L)*, Leucocoprinus fragilissimus* (L)*, Loweporus tephroporus* (W)*, Marasmius crinisequi* (L)*, Perenniporia ochroleuca* (W)*, Pleurotus pulmonarius* (W)*, Pluteus leoninus* (W)*, Psathyrella candolliana* (W)*, Pulveroboletus auriflammeus* (ECM)*, Russula vesca* (ECM)*, Xerula radicata* (W)*, Xylaria polymorpha* (W) |
| 25-Sep-94 | *Agaricus praeclaresquamosus* (L)*, Agrocybe cylindracea* (W)*, Aleuria aurantia* (L)*, Auricularia polytricha* (W)*, Calvatia craniiformis* (L)*, Cryptoporus volvatus* (W)*, Cyclomyces fuscus* (W)*, Daedaleopsis styracina* (W)*, Entoloma staurosporum* (L)*, Hebeloma vinosophyllum* (ECM)*, Isaria takamizusanensis (NA), Kuehneromyces mutabilis* (W)*, Lentinula edodes* (W)*, Lenzites betulinus* (W)*, Lepiota cygnea* (L)*, Leucoagaricus rubrotinctus* (L)*, Leucocoprinus fragilissimus* (L)*, Marasmius crinisequi* (L)*, Microporus affinis* (W)*, Microporus vernicipes* (W)*, Mycena haematopoda* (W)*, Perenniporia ochroleuca* (W)*, Pholiota highlandensis* (W)*, Piptoporus soloniensis* (W)*, Pleurotus ostreatus* (W)*, Pleurotus pulmonarius* (W)*, Psathyrella candolliana* (W)*, Psathyrella piluliformis* (W)*, Russula eburneoareolata* (ECM)*, Trametes versicolor* (W)*, Tyromyces chioneus* (W)*, Xeromphalina campanella* (W) |
| 30-Oct-94 | *Agaricus abruptibulbus* (L)*, Agaricus subrutilescens* (L)*, Amanita abrupta* (ECM)*, Amanita fulva* (ECM)*, Amanita pantherina* (ECM)*, Amanita pseudoporphyria* (ECM)*, Amanita vaginata var. punctata* (ECM)*, Amanita virosa* (ECM)*, Armillaria mellea* (W)*, Auricularia polytricha* (W)*, Austroboletus fusisporus* (ECM)*, Austroboletus subvirens* (ECM)*, Boletellus emodensis* (ECM)*, Boletus fraternus* (ECM)*, Calocera cornea* (W)*, Collybia butyracea* (L)*, Coltricia pusilla* (ECM)*, Coprinus atramentarius* (L)*, Coprinus comatus* (L)*, Cortinarius aureobrunneus* (ECM)*, Cortinarius galeroides* (ECM)*, Cortinarius purpurascens* (ECM)*, Cortinarius rubicundulus* (ECM)*, Cortinarius salor* (ECM)*, Cyclomyces fuscus* (W)*, Daedaleopsis purpurea* (W)*, Daedaleopsis styracina* (W)*, Dicephalospora rufocornea* (W)*, Ganoderma applanatum* (W)*, Gymnopilus liquiritiae* (W)*, Hygrocybe aurantia* (L)*, Hypholoma fasciculare* (W)*, Inonotus mikadoi* (W)*, Lactarius camphoratus* (ECM)*, Lactarius chrysorrheus* (ECM)*, Lactarius gracilis* (ECM)*, Lactarius hysginus* (ECM)*, Lactarius quietus* (ECM)*, Lentinellus ursinus* (W)*, Lenzites betulinus* (W)*, Leucoagaricus rubrotinctus* (L)*, Loweporus tephroporus* (W)*, Lyophyllum decastes* (L)*, Marasmius cohaerens* (L)*, Marasmius crinisequi* (L)*, Microporus affinis* (W)*, Microporus vernicipes* (W)*, Morganella pyriformis* (L)*, Mycena haematopoda* (W)*, Oligoporus caesius* (W)*, Paxillus atrotomentosus* (W)*, Perenniporia ochroleuca* (W)*, Phallus impudicus* (L)*, Pholiota highlandensis* (W)*, Phylloporus bellus* (ECM)*, Pleurocybella porrigens* (W)*, Pleurotus pulmonarius* (W)*, Pluteus leoninus* (W)*, Polyporus alveolarius* (W)*, Psathyrella candolliana* (W)*, Psathyrella piluliformis* (W)*, Pulveroboletus ravenelii* (ECM)*, Russula alboareolata* (ECM)*, Russula castanopsidis* (ECM)*, Russula emetica* (ECM)*, Russula omiensis* (ECM)*, Russula rosea* (ECM)*, Russula sororia* (ECM)*, Scleroderma areolatum* (ECM)*, Scutellinia scutellata* (W)*, Stereopsis burtianum* (W)*, Stereum hirsutum* (W)*, Strobilomyces confusus* (ECM)*, Trametes versicolor* (W)*, Tremella mesenterica* (W)*, Trichaptum abietinum* (W)*, Tylopilus valens* (ECM)*, Tyromyces chioneus* (W) |
| 4-Dec-94 | *Amanita virosa* (ECM)*, Auricularia polytricha* (W)*, Cyathus stercoreus* (W)*, Cyclomyces fuscus* (W)*, Flammulina velutipes* (W)*, Fomitopsis pinicola* (W)*, Hypholoma fasciculare* (W)*, Hypholoma sublateritium* (W)*, Inonotus mikadoi* (W)*, Lentinula edodes* (W)*, Lenzites betulinus* (W)*, Loweporus tephroporus* (W)*, Lycoperdon spadiceum* (L)*, Perenniporia ochroleuca* (W)*, Pholiota highlandensis* (W)*, Psathyrella piluliformis* (W)*, Scleroderma cepa* (ECM)*, Trametes versicolor* (W)*, Tremella foliacea* (W)*, Trichaptum abietinum* (W) |
| 26-Feb-95 | *Bjerkandera fumosa* (W)*, Ciborinia camelliae* (L)*, Cyclomyces fuscus* (W)*, Elaphocordyceps longisegmentis (NA), Flammulina velutipes* (W)*, Loweporus tephroporus* (W)*, Psathyrella piluliformis* (W)*, Russula omiensis* (ECM)*, Trametes versicolor* (W)*, Trichaptum abietinum* (W) |
| 26-Mar-95 | *Auricularia polytricha* (W)*, Bjerkandera fumosa* (W)*, Caloscypha fulgens* (L)*, Calvatia craniiformis* (L)*, Ciborinia camelliae* (L)*, Cryptoporus volvatus* (W)*, Cyathus stercoreus* (W)*, Cystidiophorus castaneus* (W)*, Elaphocordyceps longisegmentis (NA), Entoloma staurosporum* (L)*, Flammulina velutipes* (W)*, Hypholoma fasciculare* (W)*, Lentinula edodes* (W)*, Lenzites betulinus* (W)*, Melanoleuca melaleuca* (L)*, Morchella conica* (L)*, Pleurotus ostreatus* (W)*, Polyporus alveolarius* (W)*, Porodisculus pendulus* (W)*, Psathyrella piluliformis* (W)*, Russula omiensis* (ECM)*, Trametes versicolor* (W)*, Tremella mesenterica* (W) |
| 23-Apr-95 | *Amanita fuliginea* (ECM)*, Auricularia auricula* (W)*, Ciborinia camelliae* (L)*, Cordyceps annullata (NA), Elaphocordyceps longisegmentis (NA), Exdia glandulosa* (W)*, Hygrocybe aurantia* (L)*, Hypholoma fasciculare* (W)*, Lactarius camphoratus* (ECM)*, Lentinula edodes* (W)*, Lepista nuda* (L)*, Lycoperdon perlatum* (L)*, Morchella conica* (L)*, Mycena haematopoda* (W)*, Pluteus atricapillus* (W)*, Russula ochroleuca* (ECM)*, Russula omiensis* (ECM)*, Tricholoma ustale* (ECM)*, Tyromyces chioneus* (W) |
| 16-Sep-95 | *Agaricus praeclaresquamosus* (L)*, Agrocybe cylindracea* (W)*, Amanita griseofarinosa* (ECM)*, Antrodiella zonata* (W)*, Armillaria tabescens* (W)*, Astraeus hygrometricus* (ECM)*, Auricularia polytricha* (W)*, Boletus umbriniporus* (ECM)*, Calostoma japonicum* (ECM)*, Clavulina cristata* (ECM)*, Coltricia pusilla* (ECM)*, Cryptoporus volvatus* (W)*, Cyclomyces fuscus* (W)*, Daedaleopsis styracina* (W)*, Ganoderma applanatum* (W)*, Ganoderma lucidum* (W)*, Lenzites betulinus* (W)*, Microporus affinis* (W)*, Paxillus atrotomentosus* (W)*, Perenniporia ochroleuca* (W)*, Pluteus atricapillus* (W)*, Pluteus leoninus* (W)*, Psathyrella piluliformis* (W)*, Pycnoporus coccineus* (W)*, Sparassis crispa* (W)*, Trametes versicolor* (W)*, Trichaptum abietinum* (W)*, Trichaptum biforme* (W)*, Tylopilus castaneiceps* (ECM)*, Tylopilus ferrugineus* (ECM)*, Xeromphalina campanella* (W) |
| 22-Oct-95 | *Agrocybe cylindracea* (W)*, Amanita virosa* (ECM)*, Armillaria mellea* (W)*, Bjerkandera fumosa* (W)*, Coltricia pusilla* (ECM)*, Coprinus disseminatus* (W)*, Cyclomyces fuscus* (W)*, Datronia mollis* (W)*, Dicephalospora rufocornea* (W)*, Fomitopsis pinicola* (W)*, Ganoderma applanatum* (W)*, Geastrum triplex* (L)*, Grifola frondosa* (W)*, Gymnopilus liquiritiae* (W)*, Hygrocybe aurantia* (L)*, Hypholoma fasciculare* (W)*, Kobayasia nipponica* (L)*, Lactarius gracilis* (ECM)*, Lentinula edodes* (W)*, Lenzites betulinus* (W)*, Microporus affinis* (W)*, Mycena haematopoda* (W)*, Panellus stypticus* (W)*, Perenniporia ochroleuca* (W)*, Phaeolepiota aurea* (W)*, Pholiota malicola* (W)*, Pleurotus pulmonarius* (W)*, Pluteus atricapillus* (W)*, Polyporus alveolarius* (W)*, Psathyrella piluliformis* (W)*, Russula pectinatoides* (ECM)*, Trametes versicolor* (W)*, Tremella mesenterica* (W)*, Xylaria polymorpha* (W) |
| 24-Mar-96 | *Antrodiella zonata* (W)*, Auricularia polytricha* (W)*, Ciborinia camelliae* (L)*, Cryptoporus volvatus* (W)*, Cyclomyces fuscus* (W)*, Daedaleopsis purpurea* (W)*, Diatrype disciformis* (W)*, Elaphocordyceps longisegmentis (NA), Entoloma papillatus* (L)*, Entoloma staurosporum* (L)*, Exdia glandulosa* (W)*, Lentinula edodes* (W)*, Microporus affinis* (W)*, Morchella conica* (L)*, Pleurotus ostreatus* (W)*, Polyporus alveolarius* (W)*, Pycnoporus coccineus* (W)*, Russula omiensis* (ECM)*, Trametes versicolor* (W)*, Tremella mesenterica* (W) |
| 21-Apr-96 | *Coltricia pusilla* (ECM)*, Cordyceps ophioglossoides (NA), Cortinarius subalboviolaceus* (ECM)*, Cryptoporus volvatus* (W)*, Cyclomyces fuscus* (W)*, Elaphocordyceps longisegmentis (NA), Exdia glandulosa* (W)*, Exdia uvapassa* (W)*, Hypholoma fasciculare* (W)*, Lentinellus ursinus* (W)*, Lentinula edodes* (W)*, Lycoperdon perlatum* (L)*, Microporus affinis* (W)*, Morchella conica* (L)*, Pleurotus ostreatus* (W)*, Psathyrella candolliana* (W)*, Psathyrella piluliformis* (W)*, Pycnoporus coccineus* (W)*, Russula omiensis* (ECM)*, Trametes versicolor* (W)*, Tremella mesenterica* (W) |
| 23-Jun-96 | *Agaricus abruptibulbus* (L)*, Agaricus subrutilescens* (L)*, Agrocybe cylindracea* (W)*, Agrocybe erebia* (W)*, Amanita fuliginea* (ECM)*, Amanita pantherina* (ECM)*, Amanita vaginata var. vaginata* (ECM)*, Antrodiella zonata* (W)*, Artomyces pyxidatus* (W)*, Astraeus hygrometricus* (ECM)*, Auricularia auricula* (W)*, Auricularia polytricha* (W)*, Boletellus shichianus* (ECM)*, Boletus aokii* (ECM)*, Boletus fraternus* (ECM)*, Boletus subvelutipes* (ECM)*, Calvatia craniiformis* (L)*, Campanella junghuhnii* (W)*, Chalciporus piperatus* (ECM)*, Clavulina amethystinoides* (ECM)*, Clavulina cristata* (ECM)*, Clavulina rugosa* (ECM)*, Collybia neofusipes* (L)*, Coprinus micaceus* (W)*, Coprinus radians* (W)*, Cordyceps annullata (NA), Cordyceps heteropoda (NA), Cordyceps japonensis (NA), Cordyceps oxycephala (NA), Cordyceps sphecocephala (NA), Cortinarius galeroides* (ECM)*, Cortinarius subalboviolaceus* (ECM)*, Cortinarius violaceus* (ECM)*, Cryptoporus volvatus* (W)*, Exdia glandulosa* (W)*, Exdia uvapassa* (W)*, Gerronema fibula* (L)*, Gyroporus castaneus* (ECM)*, Hygrocybe aurantia* (L)*, Inonotus mikadoi* (W)*, Lactarius camphoratus* (ECM)*, Lactarius gerardii* (ECM)*, Lactarius gracilis* (ECM)*, Lepiota sistrata* (L)*, Leucocoprinus birnbaumii* (L)*, Leucocoprinus fragilissimus* (L)*, Leucocoprinus subglobisporus* (L)*, Marasmius maximus* (L)*, Marasmius pulcherripes* (L)*, Marasmius purpureostriatus* (L)*, Microporus affinis* (W)*, Mycena haematopoda* (W)*, Neolentinus lepideus* (W)*, Nomuraea atypicola (NA), Omphalina epichysium* (L)*, Phylloporus bellus* (ECM)*, Pleurotus pulmonarius* (W)*, Polyporus alveolarius* (W)*, Psathyrella candolliana* (W)*, Pycnoporus coccineus* (W)*, Russula alboareolata* (ECM)*, Russula compacta* (ECM)*, Russula cyanoxantha* (ECM)*, Russula emetica* (ECM)*, Russula laurocerasi* (ECM)*, Russula mariae* (ECM)*, Russula pectinatoides* (ECM)*, Russula violeipes* (ECM)*, Schizophyllum commune* (W)*, Suillus granulatus* (ECM)*, Tremella mesenterica* (W)*, Trichaptum biforme* (W)*, Xeromphalina campanella* (W)*, Xerula pudens* (W)*, Xerula radicata* (W) |
| 21-Jul-96 | *Agrocybe cylindracea* (W)*, Amanita castanopsidis* (ECM)*, Amanita citrina var. citrina* (ECM)*, Amanita farinosa* (ECM)*, Amanita griseofarinosa* (ECM)*, Amanita pantherina* (ECM)*, Amanita pseudoporphyria* (ECM)*, Amanita punctata* (ECM)*, Amanita rubescens* (ECM)*, Amanita sychnopyramis f. subannulata* (ECM)*, Amanita vaginata var. vaginata* (ECM)*, Amanita virosa* (ECM)*, Amanita volvata* (ECM)*, Armillaria tabescens* (W)*, Astraeus hygrometricus* (ECM)*, Aureoboletus thibetanus* (ECM)*, Austroboletus subvirens* (ECM)*, Boletellus emodensis* (ECM)*, Boletellus obscurecoccineus* (ECM)*, Boletus fraternus* (ECM)*, Boletus ornatipes* (ECM)*, Cantharellus cibarius* (ECM)*, Coltricia cinnamomea* (ECM)*, Cordyceps annullata (NA), Cordyceps japonensis (NA), Cryptoporus volvatus* (W)*, Helvella ephippioides* (ECM)*, Hydnangium carneum* (ECM)*, Hymenogaster tener* (ECM)*, Inocybe lutea* (ECM)*, Laccaria vinaceoavellanea* (ECM)*, Lactarius glaucescens* (ECM)*, Lactarius piperatus* (ECM)*, Lactarius subvellereus* (ECM)*, Marasmius purpureostriatus* (L)*, Microporus affinis* (W)*, Neolentinus lepideus* (W)*, Nomuraea atypicola (NA), Paxillus atrotomentosus* (W)*, Pisolithus tinctorius* (ECM)*, Pulveroboletus ravenelii* (ECM)*, Pycnoporus coccineus* (W)*, Russula amoena* (ECM)*, Russula castanopsidis* (ECM)*, Russula densifolia* (ECM)*, Russula japonica* (ECM)*, Russula kansaiensis* (ECM)*, Russula lilacea* (ECM)*, Russula vesca* (ECM)*, Scleroderma areolatum* (ECM)*, Stereum gausapatum* (W)*, Thelephora aurantiotincta* (ECM)*, Trametes versicolor* (W)*, Tremella foliacea* (W)*, Tremella mesenterica* (W)*, Trichaptum biforme* (W)*, Tylopilus ballouii* (ECM)*, Tylopilus ferrugineus* (ECM)*, Tylopilus valens* (ECM)*, Xeromphalina campanella* (W) |
| 18-Aug-96 | *Agaricus praeclaresquamosus* (L)*, Agrocybe cylindracea* (W)*, Amanita alboflavescens* (ECM)*, Amanita fuliginea* (ECM)*, Amanita verna* (ECM)*, Anamika lactariolens* (ECM)*, Auricularia auricula* (W)*, Auriscalpium vulgare (NA), Austroboletus fusisporus* (ECM)*, Cordyceps japonensis (NA), Crepidotus badiofloccosus* (W)*, Cryptoporus volvatus* (W)*, Cyclomyces fuscus* (W)*, Daedaleopsis styracina* (W)*, Entoloma depluens* (L)*, Fistulina hepatica* (W)*, Geastrum mirabile* (L)*, Isaria takamizusanensis (NA), Laetiporus versisporus* (W)*, Lenzites betulinus* (W)*, Marasmiellus nigripes* (W)*, Microporus affinis* (W)*, Neolentinus lepideus* (W)*, Paxillus atrotomentosus* (W)*, Perenniporia ochroleuca* (W)*, Russula subnigricans* (ECM)*, Xeromphalina campanella* (W)*, Xylobolus spectabilis* (W) |
| 29-Sep-96 | *Agaricus abruptibulbus* (L)*, Agrocybe cylindracea* (W)*, Amanita abrupta* (ECM)*, Amanita alboflavescens* (ECM)*, Amanita flavipes* (ECM)*, Amanita hongoi* (ECM)*, Amanita neoovoidea* (ECM)*, Amanita pantherina* (ECM)*, Amanita pseudoporphyria* (ECM)*, Amanita rubescens* (ECM)*, Amanita spissacea* (ECM)*, Amanita sychnopyramis f. subannulata* (ECM)*, Amanita vaginata var. vaginata* (ECM)*, Amanita virosa* (ECM)*, Amanita volvata* (ECM)*, Antrodiella zonata* (W)*, Armillaria mellea* (W)*, Aureoboletus thibetanus* (ECM)*, Auricularia polytricha* (W)*, Austroboletus subvirens* (ECM)*, Boletus granulopunctatus* (ECM)*, Boletus reticulatus* (ECM)*, Calostoma japonicum* (ECM)*, Calvatia craniiformis* (L)*, Cantharellus infundibuliformis* (ECM)*, Clavaria aurantio-cinnabarina* (L)*, Clavulina amethystinoides* (ECM)*, Clavulina rugosa* (ECM)*, Coltricia cinnamomea* (ECM)*, Coprinus radians* (W)*, Cortinarius anomalus* (ECM)*, Cortinarius galeroides* (ECM)*, Cortinarius rubicundulus* (ECM)*, Craterellus cornucopioides* (ECM)*, Entoloma album* (L)*, Entoloma murraii* (L)*, Fistulina hepatica* (W)*, Ganoderma lucidum* (W)*, Grifola frondosa* (W)*, Gymnopilus liquiritiae* (W)*, Helvella atra* (ECM)*, Hygrocybe aurantia* (L)*, Hygrocybe turunda* (L)*, Hypholoma fasciculare* (W)*, Inocybe lutea* (ECM)*, Inonotus mikadoi* (W)*, Isaria takamizusanensis (NA), Kobayasia nipponica* (L)*, Laccaria bicolor* (ECM)*, Laccaria nigra* (ECM)*, Laccaria vinaceoavellanea* (ECM)*, Lactarius akahatsu* (ECM)*, Lactarius gracilis* (ECM)*, Lactarius piperatus* (ECM)*, Lactarius quietus* (ECM)*, Lactarius vellereus* (ECM)*, Laetiporus versisporus* (W)*, Leotia lubrica* (L)*, Lepiota praetervisa* (L)*, Lycoperdon perlatum* (L)*, Lyophyllum decastes* (L)*, Morganella pyriformis* (L)*, Mycena haematopoda* (W)*, Paxillus atrotomentosus* (W)*, Phylloporus bellus* (ECM)*, Pisolithus tinctorius* (ECM)*, Pleurotus pulmonarius* (W)*, Polyporus alveolarius* (W)*, Russula alboareolata* (ECM)*, Russula castanopsidis* (ECM)*, Russula crustosa* (ECM)*, Russula cyanoxantha* (ECM)*, Russula densifolia* (ECM)*, Russula eburneoareolata* (ECM)*, Russula japonica* (ECM)*, Russula laurocerasi* (ECM)*, Russula lilacea* (ECM)*, Russula mariae* (ECM)*, Russula pectinatoides* (ECM)*, Russula vesca* (ECM)*, Russula violeipes* (ECM)*, Sparassis crispa* (W)*, Stereum gausapatum* (W)*, Strobilomyces confusus* (ECM)*, Suillus bovinus* (ECM)*, Suillus granulatus* (ECM)*, Suillus luteus* (ECM)*, Trametes versicolor* (W)*, Tremella foliacea* (W)*, Tylopilus rugulosoreticulatus* (ECM)*, Tylopilus valens* (ECM)*, Xerula pudens* (W) |
| 20-Oct-96 | *Agaricus subrutilescens* (L)*, Agrocybe cylindracea* (W)*, Agrocybe erebia* (W)*, Amanita abrupta* (ECM)*, Amanita rufoferruginea* (ECM)*, Amanita virosa* (ECM)*, Armillaria mellea* (W)*, Astraeus hygrometricus* (ECM)*, Callistosporium luteoolivaceum* (L)*, Calostoma japonicum* (ECM)*, Camarophyllus virgineus* (L)*, Cantharellus cinereus* (ECM)*, Clavaria aurantio-cinnabarina* (L)*, Collybia acervata* (L)*, Coltricia cinnamomea* (ECM)*, Coltricia pusilla* (ECM)*, Cortinarius bolaris* (ECM)*, Cortinarius galeroides* (ECM)*, Cortinarius purpurascens* (ECM)*, Cortinarius rubicundulus* (ECM)*, Craterellus cornucopioides* (ECM)*, Cyclomyces fuscus* (W)*, Daedaleopsis styracina* (W)*, Dicephalospora rufocornea* (W)*, Entoloma murraii* (L)*, Gloeophyllum sepiarium* (W)*, Gomphidius roseus* (ECM)*, Helvella macropus* (ECM)*, Hydnangium carneum* (ECM)*, Hygrocybe aurantia* (L)*, Hygrocybe conica* (L)*, Hypholoma fasciculare* (W)*, Inocybe lutea* (ECM)*, Inonotus vallatus* (W)*, Kobayasia nipponica* (L)*, Laccaria bicolor* (ECM)*, Laccaria laccata* (ECM)*, Lactarius chrysorrheus* (ECM)*, Lactarius gracilis* (ECM)*, Lactarius quietus* (ECM)*, Lentinellus ursinus* (W)*, Lenzites betulinus* (W)*, Leotia lubrica* (L)*, Lycoperdon perlatum* (L)*, Lyophyllum decastes* (L)*, Morganella pyriformis* (L)*, Mycena galericulata* (W)*, Mycena haematopoda* (W)*, Phaeolepiota aurea* (W)*, Pleurotus pulmonarius* (W)*, Polyporus alveolarius* (W)*, Psathyrella piluliformis* (W)*, Pycnoporus coccineus* (W)*, Russula castanopsidis* (ECM)*, Russula compacta* (ECM)*, Russula emetica* (ECM)*, Russula kansaiensis* (ECM)*, Russula lilacea* (ECM)*, Russula mariae* (ECM)*, Scleroderma areolatum* (ECM)*, Stereum gausapatum* (W)*, Suillus luteus* (ECM)*, Xerula radicata* (W) |
| 17-Nov-96 | *Antrodiella zonata* (W)*, Auricularia polytricha* (W)*, Bjerkandera fumosa* (W)*, Calvatia craniiformis* (L)*, Coprinus atramentarius* (L)*, Cordyceps ophioglossoides (NA), Craterellus cornucopioides* (ECM)*, Cyclomyces fuscus* (W)*, Daedaleopsis purpurea* (W)*, Daedaleopsis styracina* (W)*, Entoloma staurosporum* (L)*, Ganoderma applanatum* (W)*, Gloeophyllum sepiarium* (W)*, Hygrocybe aurantia* (L)*, Hypholoma fasciculare* (W)*, Hypholoma sublateritium* (W)*, Lenzites betulinus* (W)*, Leotia lubrica* (L)*, Lepista nuda* (L)*, Lyophyllum decastes* (L)*, Microporus affinis* (W)*, Morganella pyriformis* (L)*, Oligoporus caesius* (W)*, Panellus stypticus* (W)*, Phaeolepiota aurea* (W)*, Phellinus gilvus* (W)*, Pluteus atricapillus* (W)*, Psathyrella gracilis* (W)*, Psathyrella piluliformis* (W)*, Russula omiensis* (ECM)*, Scleroderma areolatum* (ECM)*, Suillus luteus* (ECM)*, Trametes versicolor* (W)*, Tremella fimbriata* (W)*, Tremella foliacea* (W)*, Trichocoma paradoxa* (W)*, Xylobolus spectabilis* (W) |
| 26-Jan-97 | *Amanita cokeri* (ECM)*, Amanita excelsa* (ECM)*, Amanita fuliginea* (ECM)*, Amanita virosa* (ECM)*, Astraeus hygrometricus* (ECM)*, Auricularia polytricha* (W)*, Boletus ornatipes* (ECM)*, Cryptoporus volvatus* (W)*, Cyclomyces fuscus* (W)*, Daedaleopsis styracina* (W)*, Galiella celebica* (W)*, Hebeloma vinosophyllum* (ECM)*, Hypholoma fasciculare* (W)*, Hypoxylon howeianum* (W)*, Leccinum intusrubens* (ECM)*, Lentinellus ursinus* (W)*, Lenzites betulinus* (W)*, Marasmius leveilleanus* (L)*, Microporus affinis* (W)*, Morganella pyriformis* (L)*, Perenniporia fraxinea* (W)*, Perenniporia ochroleuca* (W)*, Phanerochaete crossa* (W)*, Pholiota malicola* (W)*, Psathyrella piluliformis* (W)*, Russula castanopsidis* (ECM)*, Russula densifolia* (ECM)*, Russula japonica* (ECM)*, Russula omiensis* (ECM)*, Stereum gausapatum* (W)*, Trametes versicolor* (W)*, Tylopilus vinosobrunneus* (ECM) |
| 23-Feb-97 | *Auricularia polytricha* (W)*, Calostoma japonicum* (ECM)*, Ciborinia camelliae* (L)*, Coltricia pusilla* (ECM)*, Cyclomyces fuscus* (W)*, Daedaleopsis styracina* (W)*, Exdia glandulosa* (W)*, Galerina heterocystis* (L)*, Gloeophyllum subferrugineum* (W)*, Gloeophyllum trabeum* (W)*, Hypholoma fasciculare* (W)*, Lentinellus ursinus* (W)*, Lentinula edodes* (W)*, Lenzites betulinus* (W)*, Microporus affinis* (W)*, Morchella conica* (L)*, Pleurotus ostreatus* (W)*, Psathyrella piluliformis* (W)*, Pycnoporus coccineus* (W)*, Russula omiensis* (ECM)*, Schizophyllum commune* (W)*, Stereum hirsutum* (W)*, Trametes hirsuta* (W)*, Trametes orientalis* (W)*, Trametes versicolor* (W)*, Tremella mesenterica* (W)*, Trichaptum abietinum* (W) |
| 23-Mar-97 | *Cordyceps annullata (NA), Cordyceps ophioglossoides (NA), Elaphocordyceps longisegmentis (NA)* |
| 20-Apr-97 | *Auricularia polytricha* (W)*, Ciborinia camelliae* (L)*, Coprinus atramentarius* (L)*, Cryptoporus volvatus* (W)*, Cyclomyces fuscus* (W)*, Elaphocordyceps longisegmentis (NA), Entoloma staurosporum* (L)*, Ganoderma applanatum* (W)*, Hypholoma fasciculare* (W)*, Inonotus mikadoi* (W)*, Lentinellus ursinus* (W)*, Lentinula edodes* (W)*, Pluteus atricapillus* (W)*, Russula omiensis* (ECM)*, Trametes versicolor* (W)*, Trametes villosa* (W) |
| 22-Jun-97 | *Agaricus abruptibulbus* (L)*, Agaricus subrutilescens* (L)*, Agrocybe cylindracea* (W)*, Agrocybe erebia* (W)*, Amanita citrina var. grisea* (ECM)*, Amanita farinosa* (ECM)*, Artomyces pyxidatus* (W)*, Auricularia auricula* (W)*, Auricularia polytricha* (W)*, Boletellus shichianus* (ECM)*, Boletus fraternus* (ECM)*, Boletus pulverulentus* (ECM)*, Clavulina amethystinoides* (ECM)*, Coltricia pusilla* (ECM)*, Coprinus radians* (W)*, Cordyceps annullata (NA), Cordyceps heteropoda (NA), Cortinarius subalboviolaceus* (ECM)*, Cyclomyces fuscus* (W)*, Cyptotrama asprata* (W)*, Fistulina hepatica* (W)*, Gymnopilus aeruginosus* (W)*, Gymnopilus liquiritiae* (W)*, Hygrocybe aurantia* (L)*, Hygrocybe cuspidata* (L)*, Hymenogaster tener* (ECM)*, Laccaria vinaceoavellanea* (ECM)*, Lactarius camphoratus* (ECM)*, Lactarius gracilis* (ECM)*, Lyophyllum decastes* (L)*, Marasmius maximus* (L)*, Microporus affinis* (W)*, Neolentinus lepideus* (W)*, Nomuraea atypicola (NA), Pluteus atricapillus* (W)*, Psathyrella velutina* (W)*, Pycnoporus coccineus* (W)*, Russula alboareolata* (ECM)*, Russula amoena* (ECM)*, Russula compacta* (ECM)*, Russula cyanoxantha* (ECM)*, Russula kansaiensis* (ECM)*, Russula laurocerasi* (ECM)*, Russula mariae* (ECM)*, Russula pectinatoides* (ECM)*, Russula sororia* (ECM)*, Schizophyllum commune* (W)*, Scleroderma areolatum* (ECM)*, Tremella mesenterica* (W)*, Xeromphalina campanella* (W)*, Xerula pudens* (W)*, Xerula radicata* (W) |
| 12-Oct-97 | *Agaricus abruptibulbus* (L)*, Agaricus subrutilescens* (L)*, Agrocybe cylindracea* (W)*, Agrocybe erebia* (W)*, Aleuria aurantia* (L)*, Amanita abrupta* (ECM)*, Amanita citrina var. citrina* (ECM)*, Amanita pantherina* (ECM)*, Amanita vaginata var. vaginata* (ECM)*, Amanita virosa* (ECM)*, Antrodiella zonata* (W)*, Callistosporium luteoolivaceum* (L)*, Coltricia pusilla* (ECM)*, Coprinus atramentarius* (L)*, Cortinarius bolaris* (ECM)*, Cyclomyces fuscus* (W)*, Entoloma mycenoides* (L)*, Fistulina hepatica* (W)*, Grifola frondosa* (W)*, Gymnopilus liquiritiae* (W)*, Laccaria vinaceoavellanea* (ECM)*, Lactarius akahatsu* (ECM)*, Lactarius quietus* (ECM)*, Lenzites betulinus* (W)*, Loweporus tephroporus* (W)*, Lyophyllum decastes* (L)*, Microporus vernicipes* (W)*, Morganella pyriformis* (L)*, Pluteus atricapillus* (W)*, Psathyrella piluliformis* (W)*, Russula eburneoareolata* (ECM)*, Scleroderma areolatum* (ECM)*, Suillus granulatus* (ECM)*, Suillus luteus* (ECM)*, Trametes versicolor* (W)*, Tyromyces chioneus* (W) |
| 23-Nov-97 | *Antrodiella zonata* (W)*, Auricularia auricula* (W)*, Auricularia polytricha* (W)*, Baeospora myosura (NA), Calocera cornea* (W)*, Clavulina rugosa* (ECM)*, Coltricia pusilla* (ECM)*, Cordyceps ophioglossoides (NA), Cyclomyces fuscus* (W)*, Daedaleopsis styracina* (W)*, Exdia glandulosa* (W)*, Flammulina velutipes* (W)*, Gloeophyllum sepiarium* (W)*, Hydnangium carneum* (ECM)*, Hygrocybe imazekii* (L)*, Hypholoma fasciculare* (W)*, Inonotus mikadoi* (W)*, Inonotus xeranticus* (W)*, Lactarius camphoratus* (ECM)*, Lactarius gracilis* (ECM)*, Lentinellus ursinus* (W)*, Lenzites betulinus* (W)*, Marasmius crinisequi* (L)*, Microporus affinis* (W)*, Mycena alcalina* (W)*, Panellus stypticus* (W)*, Pluteus petasatus* (W)*, Psathyrella piluliformis* (W)*, Russula omiensis* (ECM)*, Scleroderma areolatum* (ECM)*, Strobilurus stephanocystis (NA), Trametes versicolor* (W)*, Xeromphalina campanella* (W)*, Xeromphalina cauticinalis* (W)*, Xylobolus spectabilis* (W) |
| 21-Dec-97 | *Antrodiella zonata* (W)*, Astraeus hygrometricus* (ECM)*, Calostoma japonicum* (ECM)*, Conocybe antipus* (L)*, Coprinus atramentarius* (L)*, Daedaleopsis styracina* (W)*, Entoloma staurosporum* (L)*, Exdia glandulosa* (W)*, Exdia uvapassa* (W)*, Gloeophyllum sepiarium* (W)*, Hypholoma fasciculare* (W)*, Hypholoma sublateritium* (W)*, Hypoxylon howeianum* (W)*, Lenzites betulinus* (W)*, Lepista nuda* (L)*, Loweporus tephroporus* (W)*, Microporus affinis* (W)*, Morganella pyriformis* (L)*, Mycena polygramma* (W)*, Panellus stypticus* (W)*, Pholiota terrestris* (W)*, Polyporus alveolarius* (W)*, Psathyrella piluliformis* (W)*, Russula omiensis* (ECM)*, Schizophyllum commune* (W)*, Stereum gausapatum* (W)*, Strobilurus stephanocystis (NA), Trametes versicolor* (W)*, Tremella mesenterica* (W) |
| 25-Jan-98 | *Antrodiella zonata* (W)*, Auricularia auricula* (W)*, Coltricia cinnamomea* (ECM)*, Coltricia pusilla* (ECM)*, Cordyceps annullata (NA), Cordyceps ophioglossoides (NA), Cyclomyces fuscus* (W)*, Daedaleopsis styracina* (W)*, Elaphocordyceps longisegmentis (NA), Flammulina velutipes* (W)*, Gerronema fibula* (L)*, Hypholoma fasciculare* (W)*, Lentinula edodes* (W)*, Mycena laevigata* (W)*, Perenniporia ochroleuca* (W)*, Psathyrella piluliformis* (W)*, Russula omiensis* (ECM)*, Schizophyllum commune* (W)*, Stereum gausapatum* (W)*, Trametes orientalis* (W) |
| 22-Feb-98 | *Antrodiella zonata* (W)*, Auricularia polytricha* (W)*, Calostoma japonicum* (ECM)*, Ciborinia camelliae* (L)*, Coltricia dependens* (ECM)*, Coltricia pusilla* (ECM)*, Cordyceps ophioglossoides (NA), Cyclomyces fuscus* (W)*, Elaphocordyceps longisegmentis (NA), Exdia glandulosa* (W)*, Exdia uvapassa* (W)*, Fomitopsis pinicola* (W)*, Hypholoma fasciculare* (W)*, Lentinellus ursinus* (W)*, Lentinula edodes* (W)*, Lycoperdon perlatum* (L)*, Morchella conica* (L)*, Mycena laevigata* (W)*, Panellus stypticus* (W)*, Polyporus alveolarius* (W)*, Psathyrella piluliformis* (W)*, Russula omiensis* (ECM)*, Scleroderma reae* (ECM)*, Stereum gausapatum* (W)*, Strobilurus stephanocystis (NA), Tremella foliacea* (W)*, Trichaptum biforme* (W) |
| 22-Mar-98 | *Antrodiella zonata* (W)*, Auricularia auricula* (W)*, Ciborinia camelliae* (L)*, Coltricia pusilla* (ECM)*, Cordyceps heteropoda (NA), Cordyceps ophioglossoides (NA), Cyclomyces fuscus* (W)*, Daedaleopsis styracina* (W)*, Entoloma staurosporum* (L)*, Exdia glandulosa* (W)*, Exdia uvapassa* (W)*, Galerina heterocystis* (L)*, Gloeophyllum sepiarium* (W)*, Hypholoma fasciculare* (W)*, Lentinula edodes* (W)*, Lenzites betulinus* (W)*, Melanoleuca melaleuca* (L)*, Microporus affinis* (W)*, Morchella conica* (L)*, Polyporus alveolarius* (W)*, Psathyrella piluliformis* (W)*, Russula omiensis* (ECM)*, Trametes versicolor* (W)*, Tremella fimbriata* (W)*, Tremella foliacea* (W)*, Tremella mesenterica* (W)*, Trichaptum abietinum* (W)*, Tricholoma ustale* (ECM)*, Xylobolus spectabilis* (W) |
| 26-Apr-98 | *Agrocybe cylindracea* (W)*, Amanita citrina var. grisea* (ECM)*, Artomyces pyxidatus* (W)*, Calostoma japonicum* (ECM)*, Coprinus atramentarius* (L)*, Cordyceps annullata (NA), Cordyceps heteropoda (NA), Cordyceps japonensis (NA), Cordyceps ophioglossoides (NA), Cortinarius violaceus* (ECM)*, Cryptoporus volvatus* (W)*, Cyclomyces fuscus* (W)*, Elaphocordyceps longisegmentis (NA), Entoloma staurosporum* (L)*, Exdia glandulosa* (W)*, Fistulina hepatica* (W)*, Hygrocybe aurantia* (L)*, Hypholoma fasciculare* (W)*, Isaria takamizusanensis (NA), Laccaria bicolor* (ECM)*, Laccaria nigra* (ECM)*, Lactarius camphoratus* (ECM)*, Lactarius gracilis* (ECM)*, Lactarius subplinthogalus* (ECM)*, Lentinellus ursinus* (W)*, Lyophyllum decastes* (L)*, Mycena sanguinolenta* (W)*, Neolentinus lepideus* (W)*, Oligoporus caesius* (W)*, Pholiota terrestris* (W)*, Pluteus atricapillus* (W)*, Psathyrella candolliana* (W)*, Psathyrella velutina* (W)*, Pycnoporus coccineus* (W)*, Russula castanopsidis* (ECM)*, Russula emetica* (ECM)*, Russula omiensis* (ECM)*, Russula vesca* (ECM)*, Stereum gausapatum* (W)*, Tremella mesenterica* (W)*, Trichoglossum farlowi* (L)*, Xeromphalina campanella* (W) |
| 31-May-98 | *Agaricus abruptibulbus* (L)*, Agrocybe cylindracea* (W)*, Amanita farinosa* (ECM)*, Amanita griseofarinosa* (ECM)*, Armillaria tabescens* (W)*, Astraeus hygrometricus* (ECM)*, Clavulina amethystinoides* (ECM)*, Collybia neofusipes* (L)*, Coltricia cinnamomea* (ECM)*, Coprinus radians* (W)*, Cordyceps annullata (NA), Cortinarius alboviolaceus* (ECM)*, Cortinarius violaceus* (ECM)*, Entoloma murraii* (L)*, Fistulina hepatica* (W)*, Ganoderma applanatum* (W)*, Gymnopilus liquiritiae* (W)*, Hygrocybe aurantia* (L)*, Hypholoma fasciculare* (W)*, Inonotus vallatus* (W)*, Lactarius gracilis* (ECM)*, Lactarius quietus* (ECM)*, Laetiporus versisporus* (W)*, Microporus affinis* (W)*, Neolentinus lepideus* (W)*, Oligoporus caesius* (W)*, Panus rudis* (W)*, Pholiota terrestris* (W)*, Pluteus atricapillus* (W)*, Pluteus leoninus* (W)*, Polyporus badius* (W)*, Psathyrella velutina* (W)*, Pycnoporus coccineus* (W)*, Russula alboareolata* (ECM)*, Russula laurocerasi* (ECM)*, Russula mariae* (ECM)*, Russula pectinata* (ECM)*, Russula pectinatoides* (ECM)*, Russula rosea* (ECM)*, Russula sororia* (ECM)*, Russula violeipes* (ECM)*, Scleroderma areolatum* (ECM)*, Stereum gausapatum* (W)*, Tremella mesenterica* (W)*, Tyromyces chioneus* (W)*, Xerocomus subtomentosus* (ECM)*, Xeromphalina campanella* (W)*, Xerula radicata* (W) |
| 21-Jun-98 | *Agrocybe cylindracea* (W)*, Amanita ceciliae* (ECM)*, Amanita farinosa* (ECM)*, Amanita pseudoporphyria* (ECM)*, Amanita rubescens* (ECM)*, Amanita sychnopyramis f. subannulata* (ECM)*, Amanita vaginata var. vaginata* (ECM)*, Astraeus hygrometricus* (ECM)*, Boletellus shichianus* (ECM)*, Boletus reticulatus* (ECM)*, Callistosporium luteoolivaceum* (L)*, Calocera cornea* (W)*, Clavulina rugosa* (ECM)*, Collybia butyracea* (L)*, Coprinus radians* (W)*, Cordyceps annullata (NA), Cortinarius anomalus* (ECM)*, Cortinarius galeroides* (ECM)*, Cortinarius salor* (ECM)*, Cortinarius subalboviolaceus* (ECM)*, Entoloma murraii* (L)*, Fistulina hepatica* (W)*, Ganoderma applanatum* (W)*, Gerronema fibula* (L)*, Gymnopilus liquiritiae* (W)*, Gyroporus castaneus* (ECM)*, Helvella atra* (ECM)*, Helvella ephippioides* (ECM)*, Hygrocybe aurantia* (L)*, Hygrocybe conica* (L)*, Hygrocybe cuspidata* (L)*, Ileodictyon gracile* (L)*, Laccaria bicolor* (ECM)*, Laccaria vinaceoavellanea* (ECM)*, Lactarius chrysorrheus* (ECM)*, Lactarius quietus* (ECM)*, Lepiota clypeolaria* (L)*, Nomuraea atypicola (NA), Oligoporus caesius* (W)*, Omphalina epichysium* (L)*, Panus rudis* (W)*, Phylloporus bellus* (ECM)*, Pisolithus tinctorius* (ECM)*, Psathyrella velutina* (W)*, Ramariopsis helvola* (ECM)*, Russula alboareolata* (ECM)*, Russula castanopsidis* (ECM)*, Russula compacta* (ECM)*, Russula emetica* (ECM)*, Russula kansaiensis* (ECM)*, Russula laurocerasi* (ECM)*, Russula mariae* (ECM)*, Russula pectinatoides* (ECM)*, Scleroderma areolatum* (ECM)*, Stereum gausapatum* (W)*, Strobilomyces confusus* (ECM)*, Tremella mesenterica* (W)*, Tylopilus ferrugineus* (ECM)*, Tylopilus fumosipes* (ECM)*, Tyromyces chioneus* (W)*, Xerocomus parvulus* (ECM)*, Xerula pudens* (W) |
| 12-Jul-98 | *Amanita ceciliae* (ECM)*, Amanita farinosa* (ECM)*, Amanita fuliginea* (ECM)*, Amanita griseofarinosa* (ECM)*, Amanita pseudoporphyria* (ECM)*, Amanita sychnopyramis f. subannulata* (ECM)*, Amanita vaginata var. vaginata* (ECM)*, Amanita virosa* (ECM)*, Armillaria tabescens* (W)*, Astraeus hygrometricus* (ECM)*, Boletellus emodensis* (ECM)*, Boletus fraternus* (ECM)*, Boletus granulopunctatus* (ECM)*, Boletus violaceofuscus* (ECM)*, Calostoma japonicum* (ECM)*, Clavaria zollingeri* (L)*, Clavulina rugosa* (ECM)*, Coltricia dependens* (ECM)*, Coltricia montagnei* (ECM)*, Coltricia pusilla* (ECM)*, Coprinus radians* (W)*, Cordyceps annullata (NA), Cryptoporus volvatus* (W)*, Cyclomyces fuscus* (W)*, Daedaleopsis styracina* (W)*, Fistulina hepatica* (W)*, Fomitopsis pinicola* (W)*, Geastrum mirabile* (L)*, Gyroporus castaneus* (ECM)*, Gyroporus logicystidiatus* (ECM)*, Hydnangium carneum* (ECM)*, Hygrocybe conica* (L)*, Hygrocybe cuspidata* (L)*, Hygrocybe turunda* (L)*, Inocybe lutea* (ECM)*, Kobayasia nipponica* (L)*, Laccaria vinaceoavellanea* (ECM)*, Lactarius gerardii* (ECM)*, Laetiporus versisporus* (W)*, Leccinum intusrubens* (ECM)*, Lycoperdon hiemale* (L)*, Microporus affinis* (W)*, Neolentinus lepideus* (W)*, Nomuraea atypicola (NA), Paxillus atrotomentosus* (W)*, Paxillus cutisii* (W)*, Perenniporia ochroleuca* (W)*, Phellinus gilvus* (W)*, Phylloporus bellus* (ECM)*, Pseudocolus schellenbergiae* (L)*, Pulveroboletus ravenelii* (ECM)*, Russula castanopsidis* (ECM)*, Russula japonica* (ECM)*, Russula kansaiensis* (ECM)*, Russula mariae* (ECM)*, Russula pectinatoides* (ECM)*, Scleroderma areolatum* (ECM)*, Sparassis crispa* (W)*, Stereum hirsutum* (W)*, Trametes versicolor* (W)*, Trichaptum abietinum* (W)*, Tylopilus ballouii* (ECM)*, Tylopilus ferrugineus* (ECM)*, Tylopilus fumosipes* (ECM)*, Tylopilus valens* (ECM)*, Xerocomus chrysenteron* (ECM)*, Xerocomus nigromaculatus* (ECM)*, Xerula pudens* (W)*, Xerula radicata* (W) |
| 30-Aug-98 | *Amanita cokeri* (ECM)*, Amanita excelsa* (ECM)*, Amanita fuliginea* (ECM)*, Amanita pseudoporphyria* (ECM)*, Amanita spissacea* (ECM)*, Amanita vaginata var. vaginata* (ECM)*, Amanita virosa* (ECM)*, Astraeus hygrometricus* (ECM)*, Boletellus emodensis* (ECM)*, Boletellus obscurecoccineus* (ECM)*, Boletus ornatipes* (ECM)*, Calostoma japonicum* (ECM)*, Clavulina cristata* (ECM)*, Coprinus radians* (W)*, Cryptoporus volvatus* (W)*, Cyclomyces fuscus* (W)*, Daedaleopsis tricolor* (W)*, Dicephalospora rufocornea* (W)*, Fomitopsis pinicola* (W)*, Galiella celebica* (W)*, Ganoderma applanatum* (W)*, Hebeloma vinosophyllum* (ECM)*, Inocybe geophylla* (ECM)*, Lactarius volemus* (ECM)*, Leccinum intusrubens* (ECM)*, Lenzites betulinus* (W)*, Marasmius crinisequi* (L)*, Marasmius leveilleanus* (L)*, Microporus affinis* (W)*, Neolentinus lepideus* (W)*, Panus rudis* (W)*, Paxillus atrotomentosus* (W)*, Perenniporia fraxinea* (W)*, Perenniporia ochroleuca* (W)*, Pluteus atricapillus* (W)*, Russula amoena* (ECM)*, Russula castanopsidis* (ECM)*, Russula densifolia* (ECM)*, Russula japonica* (ECM)*, Russula rubescens* (ECM)*, Russula subnigricans* (ECM)*, Stereum gausapatum* (W)*, Tylopilus vinosobrunneus* (ECM)*, Tyromyces chioneus* (W)*, Xeromphalina campanella* (W)*, Xerula raphanipes* (W) |
| 20-Sep-98 | *Agaricus praeclaresquamosus* (L)*, Amanita abrupta* (ECM)*, Amanita sychnopyramis f. subannulata* (ECM)*, Amanita volvata* (ECM)*, Astraeus hygrometricus* (ECM)*, Boletellus emodensis* (ECM)*, Calostoma japonicum* (ECM)*, Collybia neofusipes* (L)*, Crepidotus mollis* (W)*, Cyclomyces fuscus* (W)*, Entoloma coelestinus* (L)*, Fomitopsis pinicola* (W)*, Galerina fasciculata* (W)*, Gymnopilus liquiritiae* (W)*, Hebeloma vinosophyllum* (ECM)*, Inonotus vallatus* (W)*, Kobayasia nipponica* (L)*, Laccaria vinaceoavellanea* (ECM)*, Lactarius gracilis* (ECM)*, Leucocoprinus fragilissimus* (L)*, Megacollybia platyphylla* (W)*, Microporus affinis* (W)*, Neolentinus lepideus* (W)*, Piptoporus soloniensis* (W)*, Psathyrella candolliana* (W)*, Russula japonica* (ECM)*, Russula mariae* (ECM)*, Russula subnigricans* (ECM)*, Russula virescens* (ECM)*, Xeromphalina campanella* (W) |
| 11-Oct-98 | *Amanita ceciliae* (ECM)*, Amanita griseofarinosa* (ECM)*, Amanita hongoi* (ECM)*, Amanita pantherina* (ECM)*, Amanita pseudoporphyria* (ECM)*, Amanita rubescens* (ECM)*, Amanita spissacea* (ECM)*, Amanita sychnopyramis f. subannulata* (ECM)*, Amanita vaginata var. vaginata* (ECM)*, Amanita virosa* (ECM)*, Astraeus hygrometricus* (ECM)*, Aureoboletus thibetanus* (ECM)*, Austroboletus fusisporus* (ECM)*, Austroboletus subvirens* (ECM)*, Boletus aokii* (ECM)*, Boletus fraternus* (ECM)*, Boletus reticulatus* (ECM)*, Boletus subvelutipes* (ECM)*, Boletus violaceofuscus* (ECM)*, Calostoma japonicum* (ECM)*, Calvatia craniiformis* (L)*, Cantharellus minor* (ECM)*, Coprinus atramentarius* (L)*, Cortinarius galeroides* (ECM)*, Cortinarius subalboviolaceus* (ECM)*, Entoloma murraii* (L)*, Gloeophyllum abietinum* (W)*, Gymnopilus liquiritiae* (W)*, Hebeloma vinosophyllum* (ECM)*, Hygrocybe cuspidata* (L)*, Hypholoma fasciculare* (W)*, Laccaria ohiensis* (ECM)*, Laccaria vinaceoavellanea* (ECM)*, Lactarius gerardii* (ECM)*, Lactarius quietus* (ECM)*, Leccinum extremiorientale* (ECM)*, Lyophyllum decastes* (L)*, Mycena galericulata* (W)*, Paxillus atrotomentosus* (W)*, Phylloporus bellus* (ECM)*, Piptoporus soloniensis* (W)*, Pisolithus tinctorius* (ECM)*, Psathyrella candolliana* (W)*, Psathyrella piluliformis* (W)*, Russula alboareolata* (ECM)*, Russula castanopsidis* (ECM)*, Russula cyanoxantha* (ECM)*, Russula eburneoareolata* (ECM)*, Russula japonica* (ECM)*, Russula mariae* (ECM)*, Russula rosea* (ECM)*, Suillus granulatus* (ECM)*, Tylopilus ferrugineus* (ECM)*, Tylopilus fumosipes* (ECM)*, Xerocomus nigromaculatus* (ECM) |
| 20-Dec-98 | *Antrodiella semisupina* (W)*, Antrodiella zonata* (W)*, Astraeus hygrometricus* (ECM)*, Baeospora myosura (NA), Calostoma japonicum* (ECM)*, Cortinarius violaceus* (ECM)*, Cyclomyces fuscus* (W)*, Daedaleopsis purpurea* (W)*, Daedaleopsis styracina* (W)*, Daedaleopsis tricolor* (W)*, Galerina heterocystis* (L)*, Ganoderma applanatum* (W)*, Hygrocybe aurantia* (L)*, Hypholoma fasciculare* (W)*, Hypholoma sublateritium* (W)*, Inonotus xeranticus* (W)*, Lentinula edodes* (W)*, Lenzites betulinus* (W)*, Loweporus pubertatis* (W)*, Merulius tremellosus* (W)*, Microporus subaffinis* (W)*, Oligoporus caesius* (W)*, Phellinus gilvus* (W)*, Polyporus alveolarius* (W)*, Psathyrella piluliformis* (W)*, Russula omiensis* (ECM)*, Stereum gausapatum* (W)*, Strobilurus ohshimae* (W)*, Trametes hirsuta* (W)*, Trametes orientalis* (W) |
| 31-Jan-99 | *Antrodiella zonata* (W)*, Astraeus hygrometricus* (ECM)*, Calostoma japonicum* (ECM)*, Cyclomyces fuscus* (W)*, Daedaleopsis styracina* (W)*, Fomitopsis pinicola* (W)*, Galerina heterocystis* (L)*, Ganoderma applanatum* (W)*, Hypholoma fasciculare* (W)*, Lentinula edodes* (W)*, Lenzites betulinus* (W)*, Mycena laevigata* (W)*, Oligoporus caesius* (W)*, Panellus stypticus* (W)*, Polyporus alveolarius* (W)*, Russula omiensis* (ECM)*, Stereum sanguinolentum* (W)*, Strobilurus stephanocystis (NA), Trametes versicolor* (W)*, Tremella mesenterica* (W)*, Trichaptum biforme* (W) |
| 28-Feb-99 | *Auricularia polytricha* (W)*, Ciborinia camelliae* (L)*, Cordyceps annullata (NA), Cordyceps ophioglossoides (NA), Daedaleopsis styracina* (W)*, Daedaleopsis tricolor* (W)*, Elaphocordyceps longisegmentis (NA), Exdia glandulosa* (W)*, Exdia uvapassa* (W)*, Hydnochaete tabacinoides* (W)*, Hypholoma fasciculare* (W)*, Lentinula edodes* (W)*, Lenzites betulinus* (W)*, Loweporus tephroporus* (W)*, Mycena laevigata* (W)*, Oligoporus caesius* (W)*, Panellus stypticus* (W)*, Polyporus alveolarius* (W)*, Psathyrella piluliformis* (W)*, Pycnoporus coccineus* (W)*, Russula omiensis* (ECM)*, Stereum gausapatum* (W)*, Trametes versicolor* (W)*, Tremella mesenterica* (W) |
| 28-Mar-99 | *Antrodiella zonata* (W)*, Caloscypha fulgens* (L)*, Calostoma japonicum* (ECM)*, Ciborinia camelliae* (L)*, Cordyceps annullata (NA), Cordyceps heteropoda (NA), Cordyceps ophioglossoides (NA), Cyclomyces fuscus* (W)*, Daedaleopsis styracina* (W)*, Daedaleopsis tricolor* (W)*, Elaphocordyceps longisegmentis (NA), Entoloma staurosporum* (L)*, Exdia glandulosa* (W)*, Exdia uvapassa* (W)*, Geastrum triplex* (L)*, Hypholoma fasciculare* (W)*, Morchella conica* (L)*, Polyporus alveolarius* (W)*, Psathyrella piluliformis* (W)*, Russula omiensis* (ECM)*, Trametes orientalis* (W)*, Trichoglossum farlowi* (L)*, Trichoglossum walteri* (L)*, Tricholoma ustale* (ECM) |
| 25-Apr-99 | *Antrodiella gypsea* (W)*, Auricularia auricula* (W)*, Clavulina rugosa* (ECM)*, Coprinus atramentarius* (L)*, Cordyceps annullata (NA), Cordyceps heteropoda (NA), Cordyceps ormicarum (NA), Cyclomyces fuscus* (W)*, Daedaleopsis tricolor* (W)*, Entoloma staurosporum* (L)*, Exdia uvapassa* (W)*, Hygrocybe aurantia* (L)*, Hygrocybe conica* (L)*, Lactarius camphoratus* (ECM)*, Lentinula edodes* (W)*, Lenzites betulinus* (W)*, Lycoperdon umbrinum* (L)*, Microporus affinis* (W)*, Morchella conica* (L)*, Mycena haematopoda* (W)*, Mycena sanguinolenta* (W)*, Neolentinus lepideus* (W)*, Panellus stypticus* (W)*, Panus rudis* (W)*, Phellinus gilvus* (W)*, Phellinus robustus* (W)*, Pluteus atricapillus* (W)*, Psathyrella piluliformis* (W)*, Pycnoporus coccineus* (W)*, Russula omiensis* (ECM)*, Russula rosea* (ECM)*, Schizophyllum commune* (W)*, Schizopora flavipora* (W)*, Trametes hirsuta* (W)*, Trametes versicolor* (W)*, Tremella mesenterica* (W) |
| 11-Jul-99 | *Agaricus abruptibulbus* (L)*, Agaricus praeclaresquamosus* (L)*, Agaricus subrutilescens* (L)*, Agrocybe cylindracea* (W)*, Amanita ceciliae* (ECM)*, Amanita fulva* (ECM)*, Amanita griseofarinosa* (ECM)*, Amanita pantherina* (ECM)*, Amanita pseudoporphyria* (ECM)*, Amanita rubescens* (ECM)*, Amanita sychnopyramis f. subannulata* (ECM)*, Amanita vaginata var. punctata* (ECM)*, Amanita vaginata var. vaginata* (ECM)*, Astraeus hygrometricus* (ECM)*, Auricularia polytricha* (W)*, Austroboletus subvirens* (ECM)*, Boletus fraternus* (ECM)*, Boletus reticulatus* (ECM)*, Coprinus comatus* (L)*, Cortinarius subalboviolaceus* (ECM)*, Crepidotus mollis* (W)*, Cyclomyces fuscus* (W)*, Entoloma murraii* (L)*, Fistulina hepatica* (W)*, Hygrocybe cuspidata* (L)*, Hypholoma fasciculare* (W)*, Inocybe lutea* (ECM)*, Laccaria vinaceoavellanea* (ECM)*, Lactarius gracilis* (ECM)*, Lactarius vellereus* (ECM)*, Loweporus pubertatis* (W)*, Megacollybia platyphylla* (W)*, Melanogaster intermedius* (ECM)*, Microporus affinis* (W)*, Nomuraea atypicola (NA), Paxillus atrotomentosus* (W)*, Pseudocolus schellenbergiae* (L)*, Russula alboareolata* (ECM)*, Russula amoena* (ECM)*, Russula cyanoxantha* (ECM)*, Russula japonica* (ECM)*, Russula laurocerasi* (ECM)*, Russula pectinatoides* (ECM)*, Russula violeipes* (ECM)*, Strobilomyces confusus* (ECM)*, Trametes versicolor* (W)*, Trichaptum biforme* (W)*, Tylopilus ferrugineus* (ECM)*, Tylopilus otsuensis* (ECM)*, Volvariella subtaylori* (L)*, Xerocomus subtomentosus* (ECM)*, Xerula pudens* (W)*, Xerula radicata* (W) |
| 28-Aug-99 | *Agaricus abruptibulbus* (L)*, Agaricus praeclaresquamosus* (L)*, Agrocybe erebia* (W)*, Amanita alboflavescens* (ECM)*, Amanita citrina var. grisea* (ECM)*, Amanita excelsa* (ECM)*, Amanita farinosa* (ECM)*, Amanita fuliginea* (ECM)*, Amanita longistriata* (ECM)*, Amanita punctata* (ECM)*, Amanita rubescens* (ECM)*, Amanita similis* (ECM)*, Amanita spissacea* (ECM)*, Amanita sychnopyramis f. subannulata* (ECM)*, Amanita vaginata var. vaginata* (ECM)*, Amanita virgineoides* (ECM)*, Amanita virosa* (ECM)*, Antrodiella semisupina* (W)*, Aureoboletus thibetanus* (ECM)*, Austroboletus fusisporus* (ECM)*, Austroboletus subvirens* (ECM)*, Boletellus emodensis* (ECM)*, Boletellus russellii* (ECM)*, Boletus erythropus* (ECM)*, Boletus fraternus* (ECM)*, Boletus granulopunctatus* (ECM)*, Boletus obscureumbrinus* (ECM)*, Boletus ornatipes* (ECM)*, Boletus quercinus* (ECM)*, Boletus reticulatus* (ECM)*, Calostoma japonicum* (ECM)*, Clavulina cristata* (ECM)*, Coltricia montagnei* (ECM)*, Coltricia pusilla* (ECM)*, Cortinarius rubicundulus* (ECM)*, Craterellus cornucopioides* (ECM)*, Cryptoporus volvatus* (W)*, Cyclomyces fuscus* (W)*, Daedaleopsis styracina* (W)*, Echinochaete ruficeps* (W)*, Entoloma murraii* (L)*, Fomitopsis pinicola* (W)*, Ganoderma applanatum* (W)*, Gloeophyllum subferrugineum* (W)*, Gyroporus logicystidiatus* (ECM)*, Heimiella japonica* (ECM)*, Inocybe asterospora* (ECM)*, Kobayasia nipponica* (L)*, Laccaria vinaceoavellanea* (ECM)*, Lactarius camphoratus* (ECM)*, Lactarius gerardii* (ECM)*, Laetiporus versisporus* (W)*, Leccinum extremiorientale* (ECM)*, Leccinum hortonii* (ECM)*, Leotia lubrica* (L)*, Leucoagaricus rubrotinctus* (L)*, Microporus affinis* (W)*, Neolentinus lepideus* (W)*, Paxillus atrotomentosus* (W)*, Perenniporia ochroleuca* (W)*, Phaeolus schweinitzii* (W)*, Phellinus robustus* (W)*, Phylloporus bellus* (ECM)*, Pisolithus tinctorius* (ECM)*, Pulveroboletus auriflammeus* (ECM)*, Pulveroboletus ravenelii* (ECM)*, Russula alboareolata* (ECM)*, Russula castanopsidis* (ECM)*, Russula chloroides* (ECM)*, Russula cyanoxantha* (ECM)*, Russula densifolia* (ECM)*, Russula laurocerasi* (ECM)*, Russula lilacea* (ECM)*, Russula mariae* (ECM)*, Russula pectinatoides* (ECM)*, Russula rosacea* (ECM)*, Russula subnigricans* (ECM)*, Russula violeipes* (ECM)*, Russula virescens* (ECM)*, Stereum gausapatum* (W)*, Strobilomyces seminudus* (ECM)*, Trametes versicolor* (W)*, Tylopilus chromapes* (ECM)*, Tylopilus ferrugineus* (ECM)*, Tylopilus fumosipes* (ECM)*, Xerocomus nigromaculatus* (ECM)*, Xerocomus parvulus* (ECM)*, Xeromphalina campanella* (W)*, Xerula pudens* (W)*, Xylobolus spectabilis* (W) |
| 19-Sep-99 | *Agaricus abruptibulbus* (L)*, Agaricus subrutilescens* (L)*, Agrocybe cylindracea* (W)*, Amanita alboflavescens* (ECM)*, Amanita ceciliae* (ECM)*, Amanita farinosa* (ECM)*, Amanita vaginata var. vaginata* (ECM)*, Amanita virgineoides* (ECM)*, Amanita volvata* (ECM)*, Armillaria tabescens* (W)*, Aureoboletus thibetanus* (ECM)*, Austroboletus fusisporus* (ECM)*, Boletellus emodensis* (ECM)*, Boletellus russellii* (ECM)*, Boletus aokii* (ECM)*, Boletus fraternus* (ECM)*, Boletus subvelutipes* (ECM)*, Boletus umbriniporus* (ECM)*, Boletus violaceofuscus* (ECM)*, Callistosporium luteoolivaceum* (L)*, Calostoma japonicum* (ECM)*, Cantharellus minor* (ECM)*, Chalciporus piperatus* (ECM)*, Clavaria zollingeri* (L)*, Clavulina cristata* (ECM)*, Collybia neofusipes* (L)*, Collybia peronata* (L)*, Coltricia cinnamomea* (ECM)*, Coltricia pusilla* (ECM)*, Cortinarius rubicundulus* (ECM)*, Cortinarius salor* (ECM)*, Craterellus cornucopioides* (ECM)*, Crepidotus mollis* (W)*, Cryptoporus volvatus* (W)*, Cyclomyces fuscus* (W)*, Daedaleopsis purpurea* (W)*, Entoloma album* (L)*, Entoloma murraii* (L)*, Fomitopsis pinicola* (W)*, Fomitopsis spraguei* (W)*, Ganoderma applanatum* (W)*, Geastrum mirabile* (L)*, Geastrum triplex* (L)*, Gyroporus castaneus* (ECM)*, Gyroporus logicystidiatus* (ECM)*, Hebeloma vinosophyllum* (ECM)*, Hydnangium carneum* (ECM)*, Hygrocybe cuspidata* (L)*, Hymenochaete rubiginosa* (W)*, Hypholoma fasciculare* (W)*, Inocybe geophylla* (ECM)*, Inocybe lutea* (ECM)*, Inocybe maculata* (ECM)*, Inocybe praetervisa* (ECM)*, Kobayasia nipponica* (L)*, Laccaria bicolor* (ECM)*, Laccaria ohiensis* (ECM)*, Laccaria vinaceoavellanea* (ECM)*, Lactarius akahatsu* (ECM)*, Lactarius gerardii* (ECM)*, Lactarius piperatus* (ECM)*, Lactarius subplinthogalus* (ECM)*, Lactarius volemus* (ECM)*, Leccinum extremiorientale* (ECM)*, Leotia lubrica* (L)*, Lepiota acutesquamosa* (L)*, Lepiota pseudogranulosa* (L)*, Leucocoprinus birnbaumii* (L)*, Leucocoprinus otsuensis* (L)*, Loweporus pubertatis* (W)*, Marasmius aurantioferrugineus* (L)*, Paxillus atrotomentosus* (W)*, Phallus impudicus* (L)*, Pholiota malicola* (W)*, Phylloporus bellus* (ECM)*, Pisolithus tinctorius* (ECM)*, Polyporus arcularius* (W)*, Pulveroboletus auriflammeus* (ECM)*, Pulveroboletus ravenelii* (ECM)*, Russula alboareolata* (ECM)*, Russula amoena* (ECM)*, Russula castanopsidis* (ECM)*, Russula densifolia* (ECM)*, Russula earlei* (ECM)*, Russula eburneoareolata* (ECM)*, Russula japonica* (ECM)*, Russula kansaiensis* (ECM)*, Russula lilacea* (ECM)*, Russula mariae* (ECM)*, Russula rosacea* (ECM)*, Russula senecis* (ECM)*, Russula subnigricans* (ECM)*, Russula violeipes* (ECM)*, Skeletocutis nivea* (W)*, Stereopsis burtianum* (W)*, Strobilomyces confusus* (ECM)*, Thelephora palmata* (ECM)*, Tremella mesenterica* (W)*, Trichoglossum farlowi* (L)*, Tylopilus chromapes* (ECM)*, Tylopilus ferrugineus* (ECM)*, Tylopilus fumosipes* (ECM)*, Tylopilus nigropurpureus* (ECM)*, Tylopilus valens* (ECM)*, Tylopilus vinosobrunneus* (ECM)*, Xerocomus parvulus* (ECM)*, Xerula pudens* (W) |
| 31-Oct-99 | *Amanita ibotengutake* (ECM)*, Amanita vaginata var. vaginata* (ECM)*, Amanita virosa* (ECM)*, Armillaria mellea* (W)*, Calostoma japonicum* (ECM)*, Coprinus atramentarius* (L)*, Cortinarius purpurascens* (ECM)*, Cyclomyces fuscus* (W)*, Daedaleopsis tricolor* (W)*, Fomitopsis pinicola* (W)*, Ganoderma applanatum* (W)*, Grifola frondosa* (W)*, Gymnopilus liquiritiae* (W)*, Hygrocybe aurantia* (L)*, Hymenogaster arenarius* (ECM)*, Hypholoma fasciculare* (W)*, Inocybe lutea* (ECM)*, Inonotus vallatus* (W)*, Laccaria bicolor* (ECM)*, Lactarius chrysorrheus* (ECM)*, Lactarius gracilis* (ECM)*, Lactarius quietus* (ECM)*, Laetiporus versisporus* (W)*, Lentinula edodes* (W)*, Lycoperdon spadiceum* (L)*, Lyophyllum decastes* (L)*, Microporus affinis* (W)*, Mycena galericulata* (W)*, Mycena haematopoda* (W)*, Nidularia farcta* (W)*, Oligoporus caesius* (W)*, Phaeolepiota aurea* (W)*, Pholiota malicola* (W)*, Polyporus alveolarius* (W)*, Psathyrella piluliformis* (W)*, Russula compacta* (ECM)*, Russula cyanoxantha* (ECM)*, Stereum gausapatum* (W)*, Suillus luteus* (ECM)*, Trametes versicolor* (W)*, Tricholoma auratum* (ECM)*, Xerula radicata* (W) |
| 21-Nov-99 | *Amanita virosa* (ECM)*, Antrodiella zonata* (W)*, Calostoma japonicum* (ECM)*, Camarophyllus virgineus* (L)*, Cantharellus cinereus* (ECM)*, Coprinus atramentarius* (L)*, Cortinarius purpurascens* (ECM)*, Craterellus cornucopioides* (ECM)*, Cyclomyces fuscus* (W)*, Daedaleopsis purpurea* (W)*, Dermocybe cinnamomea* (ECM)*, Entoloma staurosporum* (L)*, Fomitopsis pinicola* (W)*, Ganoderma applanatum* (W)*, Gloeoporus dichrous* (W)*, Gymnopilus liquiritiae* (W)*, Hebeloma crustuliniforme* (ECM)*, Hygrocybe aurantia* (L)*, Hygrocybe conica* (L)*, Hygrocybe flavescens* (L)*, Hypholoma fasciculare* (W)*, Hypholoma sublateritium* (W)*, Inonotus ludovicianus* (W)*, Isaria takamizusanensis (NA), Kobayasia nipponica* (L)*, Lactarius chrysorrheus* (ECM)*, Lactarius gracilis* (ECM)*, Lactarius quietus* (ECM)*, Laetiporus sulphureus* (W)*, Lentinula edodes* (W)*, Lenzites betulinus* (W)*, Lepista nuda* (L)*, Lycoperdon spadiceum* (L)*, Lyophyllum decastes* (L)*, Microporus affinis* (W)*, Mycena haematopoda* (W)*, Mycena pura* (W)*, Neolecta irregularis* (L)*, Nidularia farcta* (W)*, Oligoporus caesius* (W)*, Panellus stypticus* (W)*, Perenniporia ochroleuca* (W)*, Perenniporia subacida* (W)*, Phaeolepiota aurea* (W)*, Phellinus gilvus* (W)*, Pleurocybella porrigens* (W)*, Pleurotus ostreatus* (W)*, Pluteus petasatus* (W)*, Polyporus alveolarius* (W)*, Psathyrella piluliformis* (W)*, Psathyrella velutina* (W)*, Pseudocolus schellenbergiae* (L)*, Pycnoporus coccineus* (W)*, Russula compacta* (ECM)*, Russula cyanoxantha* (ECM)*, Russula emetica* (ECM)*, Russula omiensis* (ECM)*, Stereum gausapatum* (W)*, Suillus bovinus* (ECM)*, Suillus luteus* (ECM)*, Trametes versicolor* (W)*, Tremella mesenterica* (W)*, Trichaptum abietinum* (W)*, Trichaptum biforme* (W)*, Trichaptum fuscoviolaceum* (W) |
| 19-Dec-99 | *Auricularia polytricha* (W)*, Calostoma japonicum* (ECM)*, Coltricia pusilla* (ECM)*, Cordyceps annullata (NA), Craterellus cornucopioides* (ECM)*, Cyclomyces fuscus* (W)*, Entoloma staurosporum* (L)*, Fomitopsis pinicola* (W)*, Ganoderma applanatum* (W)*, Hygrocybe aurantia* (L)*, Hygrocybe cantharellus* (L)*, Hypholoma fasciculare* (W)*, Hypholoma sublateritium* (W)*, Laetiporus sulphureus* (W)*, Laetiporus versisporus* (W)*, Lentinula edodes* (W)*, Merulius tremellosus* (W)*, Microporus affinis* (W)*, Morganella pyriformis* (L)*, Nidula niveo-tomentosa* (W)*, Oligoporus caesius* (W)*, Perenniporia ochroleuca* (W)*, Phellinus setifer* (W)*, Polyporus alveolarius* (W)*, Porodisculus pendulus* (W)*, Psathyrella piluliformis* (W)*, Stereum hirsutum* (W)*, Strobilurus stephanocystis (NA), Trametes versicolor* (W)*, Tremella mesenterica* (W)*, Trichoglossum farlowi* (L)*, Xylobolus spectabilis* (W) |
| 30-Jan-00 | *Antrodiella zonata* (W)*, Calostoma japonicum* (ECM)*, Ciborinia camelliae* (L)*, Coltricia pusilla* (ECM)*, Cordyceps ophioglossoides (NA), Craterellus cornucopioides* (ECM)*, Cyclomyces fuscus* (W)*, Daedaleopsis styracina* (W)*, Elaphocordyceps longisegmentis (NA), Exdia glandulosa* (W)*, Fomitopsis pinicola* (W)*, Hygrocybe aurantia* (L)*, Hymenochaete rubiginosa* (W)*, Hypholoma fasciculare* (W)*, Inonotus mikadoi* (W)*, Laetiporus versisporus* (W)*, Lentinula edodes* (W)*, Lenzites betulinus* (W)*, Mycena laevigata* (W)*, Nidularia farcta* (W)*, Panellus stypticus* (W)*, Perenniporia ochroleuca* (W)*, Polyporus alveolarius* (W)*, Psathyrella piluliformis* (W)*, Russula omiensis* (ECM)*, Strobilurus stephanocystis (NA), Trametes versicolor* (W)*, Trichaptum biforme* (W)*, Trichocoma paradoxa* (W)*, Xylobolus spectabilis* (W) |
| 20-Feb-00 | *Antrodiella zonata* (W)*, Caloscypha fulgens* (L)*, Calostoma japonicum* (ECM)*, Ciborinia camelliae* (L)*, Coltricia pusilla* (ECM)*, Cordyceps annullata (NA), Cordyceps ophioglossoides (NA), Cyclomyces fuscus* (W)*, Daedaleopsis styracina* (W)*, Elaphocordyceps longisegmentis (NA), Exdia glandulosa* (W)*, Hydnangium carneum* (ECM)*, Inonotus mikadoi* (W)*, Lentinula edodes* (W)*, Lenzites betulinus* (W)*, Lycoperdon spadiceum* (L)*, Mycena laevigata* (W)*, Psathyrella piluliformis* (W)*, Russula omiensis* (ECM)*, Trametes versicolor* (W)*, Tremella mesenterica* (W)*, Trichaptum biforme* (W) |
| 19-Mar-00 | *Antrodiella zonata* (W)*, Caloscypha fulgens* (L)*, Calostoma japonicum* (ECM)*, Ciborinia camelliae* (L)*, Cordyceps ophioglossoides (NA), Cyclomyces fuscus* (W)*, Elaphocordyceps longisegmentis (NA), Gloeophyllum sepiarium* (W)*, Hydnangium carneum* (ECM)*, Inonotus mikadoi* (W)*, Lentinula edodes* (W)*, Microporus affinis* (W)*, Psathyrella piluliformis* (W)*, Stereum gausapatum* (W)*, Trametes versicolor* (W)*, Trichaptum biforme* (W)*, Tricholoma ustale* (ECM) |
| 23-Apr-00 | *Antrodiella gypsea* (W)*, Antrodiella zonata* (W)*, Callistosporium luteoolivaceum* (L)*, Calostoma japonicum* (ECM)*, Ciborinia camelliae* (L)*, Coltricia pusilla* (ECM)*, Coprinus atramentarius* (L)*, Cordyceps annullata (NA), Cordyceps heteropoda (NA), Cordyceps ophioglossoides (NA), Cryptoporus volvatus* (W)*, Cyclomyces fuscus* (W)*, Daedaleopsis purpurea* (W)*, Daedaleopsis tricolor* (W)*, Elaphocordyceps longisegmentis (NA), Exdia glandulosa* (W)*, Exdia uvapassa* (W)*, Fistulina hepatica* (W)*, Hypholoma fasciculare* (W)*, Inonotus xeranticus* (W)*, Lentinellus ursinus* (W)*, Lentinula edodes* (W)*, Microporus affinis* (W)*, Morchella conica* (L)*, Mycena amygdalina* (W)*, Mycena galopus* (W)*, Mycena haematopoda* (W)*, Phellinus gilvus* (W)*, Pluteus atricapillus* (W)*, Polyporus alveolarius* (W)*, Porodisculus pendulus* (W)*, Psathyrella piluliformis* (W)*, Russula omiensis* (ECM)*, Stereum hirsutum* (W)*, Suillus luteus* (ECM)*, Tremella mesenterica* (W)*, Trichaptum biforme* (W)*, Trichocoma paradoxa* (W)*, Tricholoma ustale* (ECM)*, Xylobolus spectabilis* (W) |
| 21-May-00 | *Agaricus praeclaresquamosus* (L)*, Amanita citrina var. grisea* (ECM)*, Astraeus hygrometricus* (ECM)*, Auricularia auricula* (W)*, Bjerkandera adusta* (W)*, Cordyceps annullata (NA), Cordyceps heteropoda (NA), Cortinarius violaceus* (ECM)*, Cryptoporus volvatus* (W)*, Entoloma japonicus* (L)*, Entoloma staurosporum* (L)*, Exdia glandulosa* (W)*, Exdia uvapassa* (W)*, Fistulina hepatica* (W)*, Fomitopsis pinicola* (W)*, Ganoderma applanatum* (W)*, Gloeophyllum subferrugineum* (W)*, Gymnopilus aeruginosus* (W)*, Hydnotrya tulasnei* (ECM)*, Hygrocybe aurantia* (L)*, Lactarius camphoratus* (ECM)*, Lactarius gracilis* (ECM)*, Lyophyllum decastes* (L)*, Marasmius purpureostriatus* (L)*, Microporus affinis* (W)*, Mycena sanguinolenta* (W)*, Neolentinus lepideus* (W)*, Perenniporia ochroleuca* (W)*, Phaeomarasmius erinacella* (W)*, Phellinus gilvus* (W)*, Pluteus atricapillus* (W)*, Polyporus alveolarius* (W)*, Polyporus arcularius* (W)*, Porodisculus pendulus* (W)*, Pycnoporus coccineus* (W)*, Russula rosea* (ECM)*, Schizophyllum commune* (W)*, Stereum hirsutum* (W)*, Trichaptum abietinum* (W)*, Trichaptum biforme* (W)*, Trichocoma paradoxa* (W)*, Tyromyces chioneus* (W)*, Xeromphalina campanella* (W)*, Xerula radicata* (W)*, Xylobolus spectabilis* (W) |
| 18-Jun-00 | *Agaricus subrutilescens* (L)*, Amanita ceciliae* (ECM)*, Amanita vaginata var. vaginata* (ECM)*, Auricularia auricula* (W)*, Boletellus shichianus* (ECM)*, Chalciporus piperatus* (ECM)*, Clavulina rugosa* (ECM)*, Collybia neofusipes* (L)*, Cordyceps annullata (NA), Cortinarius galeroides* (ECM)*, Cortinarius subalboviolaceus* (ECM)*, Cyclomyces fuscus* (W)*, Fistulina hepatica* (W)*, Fomitopsis pinicola* (W)*, Gymnopilus liquiritiae* (W)*, Helvella lacunosa* (ECM)*, Hydnangium carneum* (ECM)*, Hydnotrya tulasnei* (ECM)*, Hygrocybe aurantia* (L)*, Hygrocybe psittacina* (L)*, Hypholoma fasciculare* (W)*, Laccaria bicolor* (ECM)*, Lactarius gracilis* (ECM)*, Marasmius maximus* (L)*, Microporus affinis* (W)*, Neolentinus lepideus* (W)*, Oligoporus caesius* (W)*, Phylloporus bellus* (ECM)*, Pluteus atricapillus* (W)*, Polyporus alveolarius* (W)*, Psathyrella candolliana* (W)*, Pycnoporus coccineus* (W)*, Russula alboareolata* (ECM)*, Russula laurocerasi* (ECM)*, Russula pectinatoides* (ECM)*, Russula sororia* (ECM)*, Russula violeipes* (ECM)*, Trametes versicolor* (W)*, Tremella mesenterica* (W)*, Trichaptum biforme* (W)*, Xerula pudens* (W) |
| 30-Jul-00 | *Agrocybe erebia* (W)*, Amanita citrina var. grisea* (ECM)*, Amanita farinosa* (ECM)*, Amanita fuliginea* (ECM)*, Amanita hongoi* (ECM)*, Amanita japonica* (ECM)*, Amanita punctata* (ECM)*, Amanita sychnopyramis f. subannulata* (ECM)*, Amanita virosa* (ECM)*, Armillaria tabescens* (W)*, Auricularia polytricha* (W)*, Coltricia pusilla* (ECM)*, Crepidotus mollis* (W)*, Cyclomyces fuscus* (W)*, Daldinia concentrica* (W)*, Dicephalospora rufocornea* (W)*, Fomitopsis pinicola* (W)*, Fomitopsis spraguei* (W)*, Ganoderma applanatum* (W)*, Ganoderma lucidum* (W)*, Hygrocybe cuspidata* (L)*, Inocybe lutea* (ECM)*, Inonotus vallatus* (W)*, Laccaria vinaceoavellanea* (ECM)*, Laetiporus versisporus* (W)*, Microporus affinis* (W)*, Microporus vernicipes* (W)*, Neolentinus lepideus* (W)*, Nomuraea atypicola (NA), Perenniporia ochroleuca* (W)*, Pleurotus pulmonarius* (W)*, Polyporus badius* (W)*, Punctularia strigosozonata* (W)*, Pycnoporus coccineus* (W)*, Russula castanopsidis* (ECM)*, Russula cyanoxantha* (ECM)*, Russula japonica* (ECM)*, Russula kansaiensis* (ECM)*, Russula lilacea* (ECM)*, Russula mariae* (ECM)*, Russula subnigricans* (ECM)*, Russula virescens* (ECM)*, Stereum gausapatum* (W)*, Trichaptum biforme* (W)*, Tylopilus ballouii* (ECM)*, Tyromyces chioneus* (W)*, Veluticeps berkeleyi* (W)*, Xerocomus parvulus* (ECM)*, Xerocomus subtomentosus* (ECM)*, Xeromphalina campanella* (W)*, Xerula pudens* (W) |
| 27-Aug-00 | *Amanita citrina var. grisea* (ECM)*, Amanita fuliginea* (ECM)*, Amanita spissacea* (ECM)*, Boletellus emodensis* (ECM)*, Boletellus obscurecoccineus* (ECM)*, Boletus fraternus* (ECM)*, Boletus umbriniporus* (ECM)*, Cryptoporus volvatus* (W)*, Cyclomyces fuscus* (W)*, Dicephalospora rufocornea* (W)*, Fomitopsis pinicola* (W)*, Fomitopsis spraguei* (W)*, Hygrocybe cuspidata* (L)*, Laccaria vinaceoavellanea* (ECM)*, Lactarius subvellereus* (ECM)*, Lenzites betulinus* (W)*, Marasmius crinisequi* (L)*, Neolentinus lepideus* (W)*, Nomuraea atypicola (NA), Paxillus atrotomentosus* (W)*, Perenniporia ochroleuca* (W)*, Pholiota malicola* (W)*, Phylloporus bellus* (ECM)*, Pisolithus tinctorius* (ECM)*, Russula eburneoareolata* (ECM)*, Russula japonica* (ECM)*, Russula subnigricans* (ECM)*, Scleroderma areolatum* (ECM)*, Thelephora terrestris* (ECM)*, Trametes versicolor* (W)*, Tylopilus ferrugineus* (ECM)*, Tyromyces chioneus* (W)*, Xeromphalina campanella* (W) |
| 17-Sep-00 | *Agaricus abruptibulbus* (L)*, Agaricus campestris* (L)*, Agaricus praeclaresquamosus* (L)*, Agaricus subrutilescens* (L)*, Amanita porphyria* (ECM)*, Amanita rubescens* (ECM)*, Amanita vaginata var. vaginata* (ECM)*, Astraeus hygrometricus* (ECM)*, Aureoboletus thibetanus* (ECM)*, Austroboletus fusisporus* (ECM)*, Boletellus emodensis* (ECM)*, Boletus aokii* (ECM)*, Callistosporium luteoolivaceum* (L)*, Calostoma japonicum* (ECM)*, Chalciporus piperatus* (ECM)*, Clavulina rugosa* (ECM)*, Coprinus disseminatus* (W)*, Cortinarius galeroides* (ECM)*, Crepidotus mollis* (W)*, Cryptoporus volvatus* (W)*, Cyclomyces fuscus* (W)*, Cyptotrama asprata* (W)*, Entoloma album* (L)*, Fomitopsis pinicola* (W)*, Fomitopsis spraguei* (W)*, Kobayasia nipponica* (L)*, Lactarius subzonarius* (ECM)*, Lenzites betulinus* (W)*, Lepiota cristata* (L)*, Leucoagaricus rubrotinctus* (L)*, Marasmius maximus* (L)*, Marasmius purpureostriatus* (L)*, Microporus affinis* (W)*, Mycena haematopoda* (W)*, Paxillus cutisii* (W)*, Phylloporus bellus* (ECM)*, Pisolithus tinctorius* (ECM)*, Pluteus leoninus* (W)*, Russula alboareolata* (ECM)*, Russula japonica* (ECM)*, Russula mariae* (ECM)*, Russula violeipes* (ECM)*, Stereum gausapatum* (W)*, Thelephora aurantiotincta* (ECM)*, Trametes versicolor* (W)*, Tremella mesenterica* (W)*, Trichaptum biforme* (W)*, Tylopilus ferrugineus* (ECM)*, Xerocomus parvulus* (ECM)*, Xeromphalina campanella* (W)*, Xerula pudens* (W)*, Xylobolus spectabilis* (W) |
| 29-Oct-00 | *Amanita abrupta* (ECM)*, Amanita pantherina* (ECM)*, Amanita virosa* (ECM)*, Auricularia auricula* (W)*, Calostoma japonicum* (ECM)*, Camarophyllus pratensis* (L)*, Camarophyllus virgineus* (L)*, Chalciporus piperatus* (ECM)*, Cortinarius bolaris* (ECM)*, Cortinarius galeroides* (ECM)*, Craterellus cornucopioides* (ECM)*, Fistulina hepatica* (W)*, Gomphidius roseus* (ECM)*, Gymnopilus liquiritiae* (W)*, Helvella atra* (ECM)*, Helvella crispa* (ECM)*, Helvella macropus* (ECM)*, Hydnangium carneum* (ECM)*, Hydnum repandum* (ECM)*, Hygrocybe aurantia* (L)*, Hygrocybe conica* (L)*, Hypholoma fasciculare* (W)*, Ileodictyon gracile* (L)*, Laccaria bicolor* (ECM)*, Laccaria nigra* (ECM)*, Laccaria ohiensis* (ECM)*, Lactarius akahatsu* (ECM)*, Lactarius gracilis* (ECM)*, Lactarius quietus* (ECM)*, Lactarius subvellereus* (ECM)*, Laetiporus sulphureus* (W)*, Leotia chlocephala* (L)*, Leotia stipitata* (L)*, Lyophyllum decastes* (L)*, Marasmius crinisequi* (L)*, Microporus affinis* (W)*, Mycena galericulata* (W)*, Mycena haematopoda* (W)*, Neolentinus lepideus* (W)*, Phaeolepiota aurea* (W)*, Phallus impudicus* (L)*, Pleurocybella porrigens* (W)*, Pleurotus djamor* (W)*, Pleurotus pulmonarius* (W)*, Pycnoporus coccineus* (W)*, Russula compacta* (ECM)*, Russula neoemetica* (ECM)*, Stereopsis burtianum* (W)*, Stereum gausapatum* (W)*, Trametes versicolor* (W)*, Tremella mesenterica* (W)*, Tyromyces chioneus* (W) |
| 26-Nov-00 | *Amanita virosa* (ECM)*, Antrodiella zonata* (W)*, Calostoma japonicum* (ECM)*, Calvatia craniiformis* (L)*, Clitocybe fragrans* (L)*, Coprinus atramentarius* (L)*, Cryptoporus volvatus* (W)*, Cyclomyces fuscus* (W)*, Entoloma staurosporum* (L)*, Fomitopsis pinicola* (W)*, Galiella celebica* (W)*, Geastrum triplex* (L)*, Gomphidius roseus* (ECM)*, Hydnangium carneum* (ECM)*, Hygrocybe aurantia* (L)*, Hypholoma fasciculare* (W)*, Hypholoma sublateritium* (W)*, Ileodictyon gracile* (L)*, Laccaria bicolor* (ECM)*, Laccaria laccata* (ECM)*, Lactarius chrysorrheus* (ECM)*, Lactarius gracilis* (ECM)*, Laetiporus sulphureus* (W)*, Lentinula edodes* (W)*, Lepista nuda* (L)*, Merulius tremellosus* (W)*, Neolecta vittelina* (L)*, Neolentinus lepideus* (W)*, Phellinus gilvus* (W)*, Psathyrella candolliana* (W)*, Psathyrella piluliformis* (W)*, Psathyrella velutina* (W)*, Pseudocolus schellenbergiae* (L)*, Russula emetica* (ECM)*, Russula omiensis* (ECM)*, Russula rosea* (ECM)*, Strobilurus stephanocystis (NA), Suillus bovinus* (ECM)*, Trametes versicolor* (W)*, Trichaptum biforme* (W)*, Trichocoma paradoxa* (W)*, Xylobolus spectabilis* (W) |
| 17-Dec-00 | *Antrodiella gypsea* (W)*, Antrodiella zonata* (W)*, Calostoma japonicum* (ECM)*, Collybia dryophila* (L)*, Coltricia pusilla* (ECM)*, Cyclomyces fuscus* (W)*, Daedaleopsis styracina* (W)*, Hydnangium carneum* (ECM)*, Hygrocybe aurantia* (L)*, Hypholoma fasciculare* (W)*, Hypholoma sublateritium* (W)*, Inonotus mikadoi* (W)*, Inonotus xeranticus* (W)*, Lactarius gracilis* (ECM)*, Lentinula edodes* (W)*, Lenzites betulinus* (W)*, Phellinus gilvus* (W)*, Psathyrella piluliformis* (W)*, Schizophyllum commune* (W)*, Trametes versicolor* (W)*, Trichaptum biforme* (W) |
| 25-Feb-01 | *Antrodiella zonata* (W)*, Caloscypha fulgens* (L)*, Calostoma japonicum* (ECM)*, Calvatia craniiformis* (L)*, Coltricia pusilla* (ECM)*, Cordyceps ophioglossoides (NA), Cryptoporus volvatus* (W)*, Cyclomyces fuscus* (W)*, Daedaleopsis purpurea* (W)*, Daedaleopsis styracina* (W)*, Exdia glandulosa* (W)*, Exdia uvapassa* (W)*, Gloeophyllum subferrugineum* (W)*, Hymenochaete rubiginosa* (W)*, Hypholoma fasciculare* (W)*, Inonotus mikadoi* (W)*, Lentinula edodes* (W)*, Lenzites betulinus* (W)*, Leotia lubrica* (L)*, Microporus affinis* (W)*, Mycena laevigata* (W)*, Perenniporia ochroleuca* (W)*, Polyporus alveolarius* (W)*, Psathyrella piluliformis* (W)*, Pycnoporus coccineus* (W)*, Russula omiensis* (ECM)*, Schizophyllum commune* (W)*, Stereum hirsutum* (W)*, Trametes orientalis* (W)*, Trametes versicolor* (W)*, Tremella mesenterica* (W)*, Trichaptum abietinum* (W)*, Trichaptum biforme* (W)*, Xylobolus spectabilis* (W) |
| 25-Mar-01 | *Caloscypha fulgens* (L)*, Ciborinia camelliae* (L)*, Cordyceps ophioglossoides (NA), Cryptoporus volvatus* (W)*, Cyclomyces fuscus* (W)*, Elaphocordyceps longisegmentis (NA), Fomitopsis pinicola* (W)*, Gloeophyllum sepiarium* (W)*, Hydnangium carneum* (ECM)*, Hypholoma fasciculare* (W)*, Lentinula edodes* (W)*, Microporus affinis* (W)*, Morchella conica* (L)*, Phellinus gilvus* (W)*, Psathyrella piluliformis* (W)*, Russula omiensis* (ECM)*, Stereum gausapatum* (W)*, Stereum hirsutum* (W)*, Trametes versicolor* (W)*, Trichaptum abietinum* (W)*, Trichaptum biforme* (W) |
| 29-Apr-01 | *Coltricia cinnamomea* (ECM)*, Coprinus atramentarius* (L)*, Cryptoporus volvatus* (W)*, Cyclomyces fuscus* (W)*, Lactarius camphoratus* (ECM)*, Lentinula edodes* (W)*, Phellinus gilvus* (W)*, Pluteus atricapillus* (W)*, Polyporus alveolarius* (W)*, Trametes versicolor* (W) |
| 27-May-01 | *Amanita citrina var. grisea* (ECM)*, Auricularia auricula* (W)*, Auricularia polytricha* (W)*, Calostoma japonicum* (ECM)*, Coltricia pusilla* (ECM)*, Cordyceps annullata (NA), Cordyceps heteropoda (NA), Cordyceps japonensis (NA), Cortinarius galeroides* (ECM)*, Cryptoporus volvatus* (W)*, Cyclomyces fuscus* (W)*, Daedaleopsis styracina* (W)*, Entoloma staurosporum* (L)*, Exdia uvapassa* (W)*, Fistulina hepatica* (W)*, Gymnopilus aeruginosus* (W)*, Hydnotrya tulasnei* (ECM)*, Hygrocybe aurantia* (L)*, Hypholoma fasciculare* (W)*, Lactarius gracilis* (ECM)*, Lentinula edodes* (W)*, Microporus affinis* (W)*, Mycena sanguinolenta* (W)*, Neolentinus lepideus* (W)*, Pluteus atricapillus* (W)*, Polyporus arcularius* (W)*, Psathyrella candolliana* (W)*, Psathyrella piluliformis* (W)*, Pseudocolus schellenbergiae* (L)*, Pycnoporus coccineus* (W)*, Russula kansaiensis* (ECM)*, Schizophyllum commune* (W)*, Stereum gausapatum* (W)*, Trametes versicolor* (W)*, Xeromphalina campanella* (W)*, Xerula pudens* (W) |
| 17-Jun-01 | *Agaricus subrutilescens* (L)*, Amanita citrina var. grisea* (ECM)*, Amanita rubescens* (ECM)*, Auricularia polytricha* (W)*, Boletellus shichianus* (ECM)*, Boletus pulverulentus* (ECM)*, Calvatia craniiformis* (L)*, Cordyceps annullata (NA), Cordyceps ophioglossoides (NA), Cortinarius subalboviolaceus* (ECM)*, Craterellus cornucopioides* (ECM)*, Cyptotrama asprata* (W)*, Fistulina hepatica* (W)*, Galiella celebica* (W)*, Gymnopilus liquiritiae* (W)*, Hebeloma vinosophyllum* (ECM)*, Helvella atra* (ECM)*, Hydnangium carneum* (ECM)*, Hydnotrya tulasnei* (ECM)*, Hypholoma fasciculare* (W)*, Laccaria vinaceoavellanea* (ECM)*, Lactarius gracilis* (ECM)*, Lactarius vellereus* (ECM)*, Marasmius pulcherripes* (L)*, Microporus vernicipes* (W)*, Neolentinus lepideus* (W)*, Pseudocolus schellenbergiae* (L)*, Pycnoporus coccineus* (W)*, Russula cyanoxantha* (ECM)*, Russula kansaiensis* (ECM)*, Russula mariae* (ECM)*, Russula pectinatoides* (ECM)*, Russula rosea* (ECM)*, Russula violeipes* (ECM)*, Suillus bovinus* (ECM)*, Xerula pudens* (W)*, Xerula radicata* (W) |
| 29-Jul-01 | *Amanita fuliginea* (ECM)*, Amanita longistriata* (ECM)*, Amanita punctata* (ECM)*, Amanita rubescens* (ECM)*, Amanita spissacea* (ECM)*, Amanita vaginata var. vaginata* (ECM)*, Amanita virosa* (ECM)*, Antrodiella gypsea* (W)*, Bjerkandera adusta* (W)*, Boletellus emodensis* (ECM)*, Boletus violaceofuscus* (ECM)*, Camarops petersii* (W)*, Coltricia pusilla* (ECM)*, Crepidotus mollis* (W)*, Cryptoporus volvatus* (W)*, Cyclomyces fuscus* (W)*, Hebeloma vinosophyllum* (ECM)*, Inocybe lutea* (ECM)*, Inonotus vallatus* (W)*, Lactarius gerardii* (ECM)*, Laetiporus versisporus* (W)*, Loweporus pubertatis* (W)*, Megacollybia platyphylla* (W)*, Microporus affinis* (W)*, Neolentinus lepideus* (W)*, Nomuraea atypicola (NA), Oligoporus caesius* (W)*, Paxillus atrotomentosus* (W)*, Perenniporia ochroleuca* (W)*, Phellinus gilvus* (W)*, Phellinus glaucescens* (W)*, Phylloporus orientalis* (ECM)*, Pleurotus pulmonarius* (W)*, Pluteus atricapillus* (W)*, Russula alboareolata* (ECM)*, Russula amoena* (ECM)*, Russula castanopsidis* (ECM)*, Russula kansaiensis* (ECM)*, Russula mariae* (ECM)*, Russula subnigricans* (ECM)*, Scleroderma areolatum* (ECM)*, Stereum ostrea* (W)*, Strobilomyces confusus* (ECM)*, Trametes villosa* (W)*, Trichaptum biforme* (W)*, Tylopilus fumosipes* (ECM)*, Xerocomus nigromaculatus* (ECM)*, Xerula pudens* (W) |
| 26-Aug-01 | *Amanita timida* (ECM)*, Amanita virgineoides* (ECM)*, Auricularia polytricha* (W)*, Boletus aokii* (ECM)*, Calostoma japonicum* (ECM)*, Camarops petersii* (W)*, Coltricia pusilla* (ECM)*, Crepidotus badiofloccosus* (W)*, Crepidotus mollis* (W)*, Cyclomyces fuscus* (W)*, Dicephalospora rufocornea* (W)*, Fomitopsis pinicola* (W)*, Fomitopsis spraguei* (W)*, Ganoderma applanatum* (W)*, Hypoxylon truncatum* (W)*, Isaria takamizusanensis (NA), Kobayasia nipponica* (L)*, Lactarius piperatus* (ECM)*, Lentinula edodes* (W)*, Leucocoprinus fragilissimus* (L)*, Microporus affinis* (W)*, Neolentinus lepideus* (W)*, Nidula niveo-tomentosa* (W)*, Paxillus atrotomentosus* (W)*, Perenniporia ochroleuca* (W)*, Phallus impudicus* (L)*, Pholiota malicola* (W)*, Pluteus atricapillus* (W)*, Pluteus leoninus* (W)*, Psathyrella piluliformis* (W)*, Pycnoporus coccineus* (W)*, Russula alboareolata* (ECM)*, Russula cyanoxantha* (ECM)*, Russula densifolia* (ECM)*, Russula japonica* (ECM)*, Russula rosacea* (ECM)*, Russula subnigricans* (ECM)*, Stereum hirsutum* (W)*, Trametes versicolor* (W)*, Trichaptum biforme* (W)*, Xeromphalina campanella* (W)*, Xylaria polymorpha* (W) |
| 16-Sep-01 | *Agaricus subrutilescens* (L)*, Agrocybe cylindracea* (W)*, Amanita ceciliae* (ECM)*, Amanita farinosa* (ECM)*, Amanita pantherina* (ECM)*, Amanita pseudoporphyria* (ECM)*, Amanita rubescens* (ECM)*, Amanita similis* (ECM)*, Amanita spissacea* (ECM)*, Amanita sychnopyramis f. subannulata* (ECM)*, Amanita vaginata var. vaginata* (ECM)*, Amanita virgineoides* (ECM)*, Amanita virosa* (ECM)*, Antrodiella fragrans* (W)*, Antrodiella semisupina* (W)*, Astraeus hygrometricus* (ECM)*, Aureoboletus thibetanus* (ECM)*, Austroboletus fusisporus* (ECM)*, Austroboletus gracilis* (ECM)*, Austroboletus subvirens* (ECM)*, Boletellus emodensis* (ECM)*, Boletellus russellii* (ECM)*, Boletus aokii* (ECM)*, Boletus fraternus* (ECM)*, Boletus ornatipes* (ECM)*, Boletus pseudocalopus* (ECM)*, Boletus reticulatus* (ECM)*, Boletus subvelutipes* (ECM)*, Boletus violaceofuscus* (ECM)*, Calostoma japonicum* (ECM)*, Calvatia craniiformis* (L)*, Collybia neofusipes* (L)*, Cordyceps sobolifera (NA), Cortinarius galeroides* (ECM)*, Cyclomyces fuscus* (W)*, Daedaleopsis purpurea* (W)*, Fistulina hepatica* (W)*, Fomitopsis spraguei* (W)*, Grifola frondosa* (W)*, Gymnopilus liquiritiae* (W)*, Gyroporus castaneus* (ECM)*, Gyroporus logicystidiatus* (ECM)*, Hebeloma sporliatum* (ECM)*, Helvella atra* (ECM)*, Helvella crispa* (ECM)*, Hygrocybe cantharellus* (L)*, Hygrocybe conica* (L)*, Hygrocybe unguinosa* (L)*, Hypholoma fasciculare* (W)*, Inocybe maculata* (ECM)*, Inonotus ludovicianus* (W)*, Inonotus mikadoi* (W)*, Kobayasia nipponica* (L)*, Lactarius akahatsu* (ECM)*, Lactarius gerardii* (ECM)*, Lactarius gracilis* (ECM)*, Leccinum extremiorientale* (ECM)*, Leotia lubrica* (L)*, Leucoagaricus rubrotinctus* (L)*, Leucocoprinus fragilissimus* (L)*, Megacollybia platyphylla* (W)*, Microporus affinis* (W)*, Mycena haematopoda* (W)*, Oligoporus caesius* (W)*, Paxillus atrotomentosus* (W)*, Penicilliopsis clavariaeformis (NA), Perenniporia ochroleuca* (W)*, Phallus impudicus* (L)*, Phellinus gilvus* (W)*, Pholiota malicola* (W)*, Phylloporus bellus* (ECM)*, Phylloporus orientalis* (ECM)*, Pisolithus tinctorius* (ECM)*, Pleurotus pulmonarius* (W)*, Pulveroboletus auriflammeus* (ECM)*, Pulveroboletus ravenelii* (ECM)*, Pycnoporus coccineus* (W)*, Russula densifolia* (ECM)*, Russula eburneoareolata* (ECM)*, Russula kansaiensis* (ECM)*, Russula mariae* (ECM)*, Russula pectinatoides* (ECM)*, Russula virescens* (ECM)*, Strobilomyces confusus* (ECM)*, Strobilomyces seminudus* (ECM)*, Trametes versicolor* (W)*, Trichaptum biforme* (W)*, Trichocoma paradoxa* (W)*, Tylopilus chromapes* (ECM)*, Tylopilus ferrugineus* (ECM)*, Tylopilus nigropurpureus* (ECM)*, Tylopilus vinosobrunneus* (ECM)*, Volvariella subtaylori* (L)*, Xerocomus nigromaculatus* (ECM)*, Xerocomus parvulus* (ECM)*, Xeromphalina campanella* (W)*, Xerula pudens* (W)*, Xylobolus spectabilis* (W) |
| 28-Oct-01 | *Amanita citrina var. citrina* (ECM)*, Astraeus hygrometricus* (ECM)*, Auricularia polytricha* (W)*, Camarophyllus pratensis* (L)*, Camarophyllus virgineus* (L)*, Cantharellus cinereus* (ECM)*, Chalciporus piperatus* (ECM)*, Coprinus atramentarius* (L)*, Cortinarius galeroides* (ECM)*, Cortinarius shigaensis* (ECM)*, Cyclomyces fuscus* (W)*, Daedaleopsis purpurea* (W)*, Daedaleopsis styracina* (W)*, Fistulina hepatica* (W)*, Fomitopsis pinicola* (W)*, Galiella celebica* (W)*, Ganoderma applanatum* (W)*, Grifola frondosa* (W)*, Gymnopilus liquiritiae* (W)*, Hebeloma sacchariolens* (ECM)*, Helvella atra* (ECM)*, Hydnangium carneum* (ECM)*, Hygrocybe aurantia* (L)*, Hypholoma fasciculare* (W)*, Kobayasia nipponica* (L)*, Laccaria ohiensis* (ECM)*, Lactarius gracilis* (ECM)*, Lactarius quietus* (ECM)*, Lentinula edodes* (W)*, Lenzites betulinus* (W)*, Leucocoprinus bresadolae* (L)*, Lycoperdon spadiceum* (L)*, Lyophyllum decastes* (L)*, Microporus affinis* (W)*, Mycena haematopoda* (W)*, Neolentinus lepideus* (W)*, Perenniporia ochroleuca* (W)*, Phaeomarasmius erinacella* (W)*, Pleurocybella porrigens* (W)*, Pluteus leoninus* (W)*, Psathyrella spadiceogrisea* (W)*, Psathyrella velutina* (W)*, Russula compacta* (ECM)*, Russula cyanoxantha* (ECM)*, Russula rosea* (ECM)*, Suillus luteus* (ECM)*, Trametes versicolor* (W)*, Trichaptum biforme* (W)*, Tyromyces chioneus* (W)*, Xerocomus subtomentosus* (ECM) |
| 27-Jan-02 | *Aleuria rhenana* (L)*, Cyclomyces fuscus* (W)*, Daedaleopsis styracina* (W)*, Elaphocordyceps longisegmentis (NA), Hydnangium carneum* (ECM)*, Microporus affinis* (W)*, Mycena laevigata* (W)*, Panellus stypticus* (W)*, Perenniporia ochroleuca* (W)*, Phellinus gilvus* (W)*, Psathyrella piluliformis* (W)*, Russula omiensis* (ECM)*, Schizophyllum commune* (W)*, Strobilurus stephanocystis (NA), Trametes versicolor* (W)*, Tremella mesenterica* (W)*, Xylobolus spectabilis* (W) |
| 24-Mar-02 | *Ciborinia camelliae* (L)*, Cordyceps heteropoda (NA), Cordyceps ophioglossoides (NA), Cryptoporus volvatus* (W)*, Cyclomyces fuscus* (W)*, Elaphocordyceps longisegmentis (NA), Hypholoma fasciculare* (W)*, Lentinula edodes* (W)*, Lycoperdon molle* (L)*, Morchella conica* (L)*, Russula omiensis* (ECM)*, Trichoglossum hirsutum* (L) |
| 21-Apr-02 | *Amanita citrina var. grisea* (ECM)*, Auricularia auricula* (W)*, Auricularia polytricha* (W)*, Coprinus atramentarius* (L)*, Coprinus radians* (W)*, Cordyceps annullata (NA), Cordyceps heteropoda (NA), Cryptoporus volvatus* (W)*, Daedaleopsis styracina* (W)*, Entoloma staurosporum* (L)*, Exdia glandulosa* (W)*, Fistulina hepatica* (W)*, Hydnangium carneum* (ECM)*, Hygrocybe aurantia* (L)*, Hypholoma fasciculare* (W)*, Lactarius camphoratus* (ECM)*, Lentinula edodes* (W)*, Loweporus pubertatis* (W)*, Mycena haematopoda* (W)*, Russula omiensis* (ECM) |
| 26-May-02 | *Auricularia polytricha* (W)*, Chalciporus piperatus* (ECM)*, Coltricia pusilla* (ECM)*, Cordyceps annullata (NA), Cortinarius galeroides* (ECM)*, Cortinarius subalboviolaceus* (ECM)*, Cortinarius violaceus* (ECM)*, Cryptoporus volvatus* (W)*, Dicephalospora rufocornea* (W)*, Fistulina hepatica* (W)*, Galiella celebica* (W)*, Hydnotrya tulasnei* (ECM)*, Hygrocybe aurantia* (L)*, Hypholoma fasciculare* (W)*, Lactarius gracilis* (ECM)*, Lentinula edodes* (W)*, Lyophyllum decastes* (L)*, Microporus affinis* (W)*, Neolentinus lepideus* (W)*, Pleurotus pulmonarius* (W)*, Psathyrella piluliformis* (W)*, Tremella mesenterica* (W)*, Trichocoma paradoxa* (W)*, Xeromphalina campanella* (W)*, Xerula pudens* (W) |
| 30-Jun-02 | *Amanita ceciliae* (ECM)*, Amanita vaginata var. vaginata* (ECM)*, Artomyces pyxidatus* (W)*, Auricularia auricula* (W)*, Boletellus shichianus* (ECM)*, Boletus subvelutipes* (ECM)*, Chalciporus piperatus* (ECM)*, Coprinus atramentarius* (L)*, Cordyceps annullata (NA), Cyptotrama asprata* (W)*, Dicephalospora rufocornea* (W)*, Galiella celebica* (W)*, Hebeloma vinosophyllum* (ECM)*, Hygrocybe aurantia* (L)*, Hypholoma fasciculare* (W)*, Laccaria bicolor* (ECM)*, Lactarius akahatsu* (ECM)*, Lactarius gracilis* (ECM)*, Lactarius subzonarius* (ECM)*, Marasmius pulcherripes* (L)*, Microporus affinis* (W)*, Mycena sanguinolenta* (W)*, Nomuraea atypicola (NA), Phylloporus bellus* (ECM)*, Pluteus atricapillus* (W)*, Psathyrella velutina* (W)*, Russula alboareolata* (ECM)*, Russula compacta* (ECM)*, Russula mariae* (ECM)*, Russula pectinatoides* (ECM)*, Tremella mesenterica* (W)*, Xerocomus nigromaculatus* (ECM)*, Xeromphalina campanella* (W)*, Xerula pudens* (W) |
| 27-Jul-02 | *Agaricus abruptibulbus* (L)*, Agrocybe cylindracea* (W)*, Agrocybe erebia* (W)*, Amanita citrina var. grisea* (ECM)*, Amanita excelsa* (ECM)*, Amanita farinosa* (ECM)*, Amanita fuliginea* (ECM)*, Amanita fulva* (ECM)*, Amanita griseofarinosa* (ECM)*, Amanita hongoi* (ECM)*, Amanita neoovoidea* (ECM)*, Amanita pantherina* (ECM)*, Amanita punctata* (ECM)*, Amanita rubescens* (ECM)*, Amanita similis* (ECM)*, Amanita spissacea* (ECM)*, Amanita sychnopyramis f. subannulata* (ECM)*, Amanita vaginata var. punctata* (ECM)*, Amanita vaginata var. vaginata* (ECM)*, Aureoboletus thibetanus* (ECM)*, Austroboletus fusisporus* (ECM)*, Austroboletus subvirens* (ECM)*, Boletellus emodensis* (ECM)*, Boletellus obscurecoccineus* (ECM)*, Boletus auripes* (ECM)*, Boletus fraternus* (ECM)*, Boletus granulopunctatus* (ECM)*, Boletus obscureumbrinus* (ECM)*, Boletus ornatipes* (ECM)*, Boletus reticulatus* (ECM)*, Boletus subcinnamomeus* (ECM)*, Boletus umbriniporus* (ECM)*, Boletus violaceofuscus* (ECM)*, Camarops petersii* (W)*, Coltricia pusilla* (ECM)*, Cyclomyces fuscus* (W)*, Entoloma murraii* (L)*, Gyroporus logicystidiatus* (ECM)*, Heimiella japonica* (ECM)*, Inocybe maculata* (ECM)*, Laccaria vinaceoavellanea* (ECM)*, Lactarius piperatus* (ECM)*, Lactarius subvellereus* (ECM)*, Leccinum extremiorientale* (ECM)*, Leccinum hortonii* (ECM)*, Microporus affinis* (W)*, Neolentinus lepideus* (W)*, Nomuraea atypicola (NA), Perenniporia ochroleuca* (W)*, Pholiota malicola* (W)*, Phylloporus orientalis* (ECM)*, Pulveroboletus ravenelii* (ECM)*, Russula amoena* (ECM)*, Russula foetens* (ECM)*, Russula japonica* (ECM)*, Russula lilacea* (ECM)*, Russula subnigricans* (ECM)*, Stereum hirsutum* (W)*, Strobilomyces seminudus* (ECM)*, Suillus granulatus* (ECM)*, Tylopilus alutaceoumbrinus* (ECM)*, Tylopilus argillaceus* (ECM)*, Tylopilus ballouii* (ECM)*, Tylopilus ferrugineus* (ECM)*, Tylopilus fumosipes* (ECM)*, Xerocomus nigromaculatus* (ECM)*, Xylobolus spectabilis* (W) |
| 31-Aug-02 | *Agaricus abruptibulbus* (L)*, Agaricus campestris* (L)*, Agrocybe cylindracea* (W)*, Armillaria tabescens* (W)*, Boletus ornatipes* (ECM)*, Crepidotus mollis* (W)*, Dicephalospora rufocornea* (W)*, Isaria takamizusanensis (NA), Leucocoprinus fragilissimus* (L)*, Loweporus pubertatis* (W)*, Microporus affinis* (W)*, Neolentinus lepideus* (W)*, Perenniporia ochroleuca* (W)*, Pholiota malicola* (W)*, Pleurotus pulmonarius* (W)*, Polyporus badius* (W)*, Psathyrella candolliana* (W)*, Pycnoporus coccineus* (W)*, Russula subnigricans* (ECM)*, Trametes versicolor* (W)*, Xeromphalina campanella* (W)*, Xylobolus spectabilis* (W) |
| 16-Sep-02 | *Agrocybe cylindracea* (W)*, Amanita farinosa* (ECM)*, Amanita hongoi* (ECM)*, Armillaria tabescens* (W)*, Aureoboletus thibetanus* (ECM)*, Boletellus emodensis* (ECM)*, Boletus fraternus* (ECM)*, Callistosporium luteoolivaceum* (L)*, Crepidotus badiofloccosus* (W)*, Crepidotus mollis* (W)*, Cyclomyces fuscus* (W)*, Dicephalospora rufocornea* (W)*, Fomitopsis pinicola* (W)*, Ganoderma applanatum* (W)*, Hypholoma fasciculare* (W)*, Kobayasia nipponica* (L)*, Laccaria vinaceoavellanea* (ECM)*, Leucocoprinus bresadolae* (L)*, Marasmius pulcherripes* (L)*, Neolentinus lepideus* (W)*, Perenniporia ochroleuca* (W)*, Pholiota malicola* (W)*, Phylloporus bellus* (ECM)*, Pluteus atricapillus* (W)*, Pluteus leoninus* (W)*, Radulodon capelandii* (W)*, Russula cyanoxantha* (ECM)*, Stereum ostrea* (W)*, Suillus granulatus* (ECM)*, Tylopilus ferrugineus* (ECM)*, Tylopilus fumosipes* (ECM)*, Tyromyces chioneus* (W)*, Xeromphalina campanella* (W) |
| 20-Oct-02 | *Agaricus campestris* (L)*, Amanita abrupta* (ECM)*, Amanita citrina var. citrina* (ECM)*, Amanita pantherina* (ECM)*, Amanita vaginata var. vaginata* (ECM)*, Amanita virosa* (ECM)*, Auricularia polytricha* (W)*, Boletus granulopunctatus* (ECM)*, Coprinus atramentarius* (L)*, Cyclomyces fuscus* (W)*, Ganoderma applanatum* (W)*, Gymnopilus liquiritiae* (W)*, Hygrocybe aurantia* (L)*, Lactarius akahatsu* (ECM)*, Lactarius chrysorrheus* (ECM)*, Lactarius gracilis* (ECM)*, Lactarius quietus* (ECM)*, Lentinula edodes* (W)*, Lepiota pseudogranulosa* (L)*, Lyophyllum decastes* (L)*, Mycena haematopoda* (W)*, Perenniporia ochroleuca* (W)*, Phellinus gilvus* (W)*, Pisolithus tinctorius* (ECM)*, Pleurotus pulmonarius* (W)*, Psathyrella velutina* (W)*, Pycnoporus coccineus* (W)*, Russula castanopsidis* (ECM)*, Russula emetica* (ECM)*, Russula mariae* (ECM)*, Russula violeipes* (ECM)*, Suillus bovinus* (ECM)*, Suillus luteus* (ECM)*, Tylopilus chromapes* (ECM)*, Tyromyces chioneus* (W)*, Xerocomus nigromaculatus* (ECM)*, Xeromphalina campanella* (W) |
| 24-Nov-02 | *Antrodiella zonata* (W)*, Coprinus atramentarius* (L)*, Cyclomyces fuscus* (W)*, Daedaleopsis tricolor* (W)*, Gloeophyllum subferrugineum* (W)*, Hydnangium carneum* (ECM)*, Hypholoma fasciculare* (W)*, Hypholoma sublateritium* (W)*, Inonotus xeranticus* (W)*, Laccaria bicolor* (ECM)*, Laetiporus sulphureus* (W)*, Lentinula edodes* (W)*, Lycoperdon perlatum* (L)*, Microporus affinis* (W)*, Nigroporus vinosus* (W)*, Oligoporus caesius* (W)*, Perenniporia ochroleuca* (W)*, Phellinus gilvus* (W)*, Phellinus robustus* (W)*, Pholiota terrestris* (W)*, Scleroderma areolatum* (ECM)*, Trametes versicolor* (W)*, Trichaptum biforme* (W) |
| 15-Dec-02 | *Calostoma japonicum* (ECM)*, Coltricia pusilla* (ECM)*, Cyclomyces fuscus* (W)*, Ganoderma applanatum* (W)*, Gloeophyllum subferrugineum* (W)*, Hydnangium carneum* (ECM)*, Hypholoma fasciculare* (W)*, Hypholoma sublateritium* (W)*, Laetiporus sulphureus* (W)*, Microporus affinis* (W)*, Oligoporus caesius* (W)*, Panellus stypticus* (W)*, Perenniporia ochroleuca* (W)*, Phellinus gilvus* (W)*, Psathyrella piluliformis* (W)*, Resupinatus trichotis* (W)*, Russula omiensis* (ECM)*, Stereum hirsutum* (W)*, Strobilurus ohshimae* (W)*, Strobilurus stephanocystis (NA), Trametes versicolor* (W)*, Trichaptum biforme* (W) |
| 26-Jan-03 | *Antrodiella zonata* (W)*, Ciborinia camelliae* (L)*, Cyclomyces fuscus* (W)*, Elaphocordyceps longisegmentis (NA), Hydnangium carneum* (ECM)*, Microporus affinis* (W)*, Mycena laevigata* (W)*, Perenniporia ochroleuca* (W)*, Russula omiensis* (ECM)*, Stereum gausapatum* (W) |
| 23-Feb-03 | *Auricularia polytricha* (W)*, Daedaleopsis styracina* (W)*, Exdia glandulosa* (W)*, Galerina heterocystis* (L)*, Hydnangium carneum* (ECM)*, Lentinula edodes* (W)*, Perenniporia ochroleuca* (W)*, Phellinus gilvus* (W)*, Pycnoporus coccineus* (W)*, Russula omiensis* (ECM)*, Thelephora multipartita* (ECM)*, Trametes versicolor* (W) |
| 23-Mar-03 | *Auricularia auricula* (W)*, Auricularia polytricha* (W)*, Caloscypha fulgens* (L)*, Ciborinia camelliae* (L)*, Cyclomyces fuscus* (W)*, Entoloma staurosporum* (L)*, Exdia glandulosa* (W)*, Exdia uvapassa* (W)*, Hypholoma fasciculare* (W)*, Lentinula edodes* (W)*, Morchella conica* (L)*, Russula omiensis* (ECM)*, Trametes versicolor* (W)*, Tremella mesenterica* (W) |
| 27-Apr-03 | *Auricularia auricula* (W)*, Chalciporus piperatus* (ECM)*, Ciborinia camelliae* (L)*, Coprinus atramentarius* (L)*, Cordyceps heteropoda (NA), Cordyceps ophioglossoides (NA), Cordyceps ormicarum (NA), Cyclomyces fuscus* (W)*, Elaphocordyceps longisegmentis (NA), Exdia glandulosa* (W)*, Exdia uvapassa* (W)*, Fistulina hepatica* (W)*, Hygrocybe aurantia* (L)*, Lactarius camphoratus* (ECM)*, Lenzites betulinus* (W)*, Microporus affinis* (W)*, Mycena sanguinolenta* (W)*, Pluteus atricapillus* (W)*, Pycnoporus coccineus* (W)*, Resupinatus trichotis* (W)*, Russula omiensis* (ECM)*, Russula rosea* (ECM)*, Suillus luteus* (ECM)*, Trametes hirsuta* (W)*, Tremella mesenterica* (W) |
| 25-May-03 | *Agrocybe cylindracea* (W)*, Amanita citrina var. grisea* (ECM)*, Amanita longistriata* (ECM)*, Amanita vaginata var. vaginata* (ECM)*, Boletus umbriniporus* (ECM)*, Calostoma japonicum* (ECM)*, Chalciporus piperatus* (ECM)*, Coprinus atramentarius* (L)*, Cordyceps annullata (NA), Cordyceps japonensis (NA), Cortinarius galeroides* (ECM)*, Cortinarius subalboviolaceus* (ECM)*, Cortinarius violaceus* (ECM)*, Craterellus cornucopioides* (ECM)*, Crepidotus badiofloccosus* (W)*, Cyptotrama asprata* (W)*, Dermocybe cinnamomea* (ECM)*, Dicephalospora rufocornea* (W)*, Fistulina hepatica* (W)*, Galiella celebica* (W)*, Hydnotrya tulasnei* (ECM)*, Hygrocybe aurantia* (L)*, Lactarius camphoratus* (ECM)*, Lactarius gracilis* (ECM)*, Lycoperdon pedicellatum* (L)*, Lyophyllum decastes* (L)*, Neolentinus lepideus* (W)*, Nidula niveo-tomentosa* (W)*, Pholiota astragalina* (W)*, Pluteus atricapillus* (W)*, Pluteus aurantiorugosus* (W)*, Psathyrella candolliana* (W)*, Pycnoporus coccineus* (W)*, Russula mariae* (ECM)*, Russula omiensis* (ECM)*, Russula violeipes* (ECM)*, Scleroderma verrucosum* (ECM)*, Xerocomus subtomentosus* (ECM) |
| 29-Jun-03 | *Agaricus placomyces* (L)*, Agrocybe cylindracea* (W)*, Amanita ceciliae* (ECM)*, Amanita farinosa* (ECM)*, Amanita pantherina* (ECM)*, Amanita pseudoporphyria* (ECM)*, Amanita sychnopyramis f. subannulata* (ECM)*, Amanita vaginata var. vaginata* (ECM)*, Antrodiella gypsea* (W)*, Boletellus shichianus* (ECM)*, Boletus aokii* (ECM)*, Boletus fraternus* (ECM)*, Boletus reticulatus* (ECM)*, Boletus subvelutipes* (ECM)*, Boletus umbriniporus* (ECM)*, Boletus violaceofuscus* (ECM)*, Clavulina rugosa* (ECM)*, Collybia neofusipes* (L)*, Coprinus atramentarius* (L)*, Coprinus plicatilis* (L)*, Cordyceps annullata (NA), Cordyceps japonensis (NA), Cortinarius galeroides* (ECM)*, Cortinarius salor* (ECM)*, Cyclomyces fuscus* (W)*, Cyptotrama asprata* (W)*, Entoloma album* (L)*, Entoloma murraii* (L)*, Galiella celebica* (W)*, Hebeloma sporliatum* (ECM)*, Helvella ephippium* (ECM)*, Helvella lacunosa* (ECM)*, Hygrocybe aurantia* (L)*, Hygrocybe conica* (L)*, Hygrocybe punicea* (L)*, Inocybe fastigiata* (ECM)*, Laccaria ohiensis* (ECM)*, Lactarius gerardii* (ECM)*, Lactarius gracilis* (ECM)*, Lactarius subzonarius* (ECM)*, Leccinum extremiorientale* (ECM)*, Lepiota praetervisa* (L)*, Lepiota pseudogranulosa* (L)*, Leucoagaricus rubrotinctus* (L)*, Marasmius pulcherripes* (L)*, Melanogaster intermedius* (ECM)*, Microporus affinis* (W)*, Neolentinus lepideus* (W)*, Nomuraea atypicola (NA), Pholiota highlandensis* (W)*, Phylloporus bellus* (ECM)*, Phylloporus orientalis* (ECM)*, Psathyrella candolliana* (W)*, Psilocybe subcaerulipes* (L)*, Pycnoporus coccineus* (W)*, Russula alboareolata* (ECM)*, Russula amoena* (ECM)*, Russula castanopsidis* (ECM)*, Russula lilacea* (ECM)*, Russula mariae* (ECM)*, Russula pectinatoides* (ECM)*, Russula violeipes* (ECM)*, Russula virescens* (ECM)*, Scleroderma verrucosum* (ECM)*, Strobilomyces confusus* (ECM)*, Strobilomyces seminudus* (ECM)*, Tylopilus argillaceus* (ECM)*, Tylopilus castaneiceps* (ECM)*, Tylopilus chromapes* (ECM)*, Tylopilus fumosipes* (ECM)*, Xerocomus nigromaculatus* (ECM)*, Xerocomus parvulus* (ECM)*, Xerula pudens* (W) |
| 13-Jul-03 | *Amanita citrina var. grisea* (ECM)*, Amanita farinosa* (ECM)*, Amanita pseudoporphyria* (ECM)*, Amanita punctata* (ECM)*, Amanita spissacea* (ECM)*, Amanita sychnopyramis f. subannulata* (ECM)*, Amanita vaginata var. vaginata* (ECM)*, Aureoboletus thibetanus* (ECM)*, Austroboletus fusisporus* (ECM)*, Austroboletus subvirens* (ECM)*, Boletus aokii* (ECM)*, Boletus pseudocalopus* (ECM)*, Cantharellus cibarius* (ECM)*, Clavaria aurantio-cinnabarina* (L)*, Clavulina rugosa* (ECM)*, Collybia butyracea* (L)*, Collybia neofusipes* (L)*, Collybia peronata* (L)*, Cortinarius subalboviolaceus* (ECM)*, Cyclomyces fuscus* (W)*, Dicephalospora rufocornea* (W)*, Entoloma murraii* (L)*, Gyroporus logicystidiatus* (ECM)*, Hebeloma sporliatum* (ECM)*, Helvella atra* (ECM)*, Helvella crispa* (ECM)*, Hygrocybe conica* (L)*, Hygrocybe cuspidata* (L)*, Inocybe lutea* (ECM)*, Laccaria bicolor* (ECM)*, Laccaria vinaceoavellanea* (ECM)*, Lactarius gerardii* (ECM)*, Lactarius gracilis* (ECM)*, Lactarius subvellereus* (ECM)*, Lactarius subzonarius* (ECM)*, Neolentinus lepideus* (W)*, Nigroporus vinosus* (W)*, Nomuraea atypicola (NA), Phylloporus bellus* (ECM)*, Phylloporus orientalis* (ECM)*, Polyporus alveolarius* (W)*, Psilocybe subcaerulipes* (L)*, Pycnoporus coccineus* (W)*, Ramariopsis fusiformis* (ECM)*, Russula alboareolata* (ECM)*, Russula amoena* (ECM)*, Russula castanopsidis* (ECM)*, Russula laurocerasi* (ECM)*, Russula lilacea* (ECM)*, Russula sororia* (ECM)*, Strobilomyces confusus* (ECM)*, Strobilomyces strobilaceus* (ECM)*, Suillus bovinus* (ECM)*, Trametes versicolor* (W)*, Tylopilus alutaceoumbrinus* (ECM)*, Tylopilus argillaceus* (ECM)*, Tylopilus nigropurpureus* (ECM)*, Xerocomus nigromaculatus* (ECM)*, Xerocomus parvulus* (ECM)*, Xerula radicata* (W) |
| 28-Sep-03 | *Agrocybe cylindracea* (W)*, Artomyces pyxidatus* (W)*, Callistosporium luteoolivaceum* (L)*, Calostoma japonicum* (ECM)*, Calvatia caelata* (L)*, Chalciporus piperatus* (ECM)*, Coltricia pusilla* (ECM)*, Crepidotus mollis* (W)*, Cryptoporus volvatus* (W)*, Cyclomyces fuscus* (W)*, Cyptotrama asprata* (W)*, Daedaleopsis styracina* (W)*, Dicephalospora rufocornea* (W)*, Galiella celebica* (W)*, Geastrum triplex* (L)*, Inocybe lutea* (ECM)*, Inonotus vallatus* (W)*, Kobayasia nipponica* (L)*, Kuehneromyces mutabilis* (W)*, Laccaria vinaceoavellanea* (ECM)*, Lactarius volemus* (ECM)*, Microporus affinis* (W)*, Mycena alcalina* (W)*, Neolentinus lepideus* (W)*, Perenniporia ochroleuca* (W)*, Polyporus badius* (W)*, Russula alboareolata* (ECM)*, Russula cyanoxantha* (ECM)*, Russula densifolia* (ECM)*, Russula japonica* (ECM)*, Russula mariae* (ECM)*, Russula sororia* (ECM)*, Russula subnigricans* (ECM)*, Russula vesca* (ECM)*, Russula violeipes* (ECM)*, Russula virescens* (ECM)*, Suillus granulatus* (ECM)*, Trametes versicolor* (W)*, Trichaptum elongatum* (W)*, Tyromyces chioneus* (W)*, Xerocomus parvulus* (ECM)*, Xeromphalina campanella* (W) |
| 26-Oct-03 | *Agrocybe cylindracea* (W)*, Amanita ibotengutake* (ECM)*, Amanita virosa* (ECM)*, Calostoma japonicum* (ECM)*, Calvatia craniiformis* (L)*, Chalciporus piperatus* (ECM)*, Collybia maculata* (L)*, Coprinus atramentarius* (L)*, Cortinarius alboviolaceus* (ECM)*, Cyclomyces fuscus* (W)*, Dermocybe cinnamomea* (ECM)*, Fomitopsis pinicola* (W)*, Hygrocybe aurantia* (L)*, Hypholoma fasciculare* (W)*, Kobayasia nipponica* (L)*, Lactarius chrysorrheus* (ECM)*, Lactarius gracilis* (ECM)*, Laetiporus versisporus* (W)*, Lyophyllum decastes* (L)*, Microporus affinis* (W)*, Mycena haematopoda* (W)*, Phaeolepiota aurea* (W)*, Phellinus gilvus* (W)*, Pleurocybella porrigens* (W)*, Psathyrella piluliformis* (W)*, Stereopsis burtianum* (W)*, Trametes versicolor* (W)*, Trichocoma paradoxa* (W) |
| 23-Nov-03 | *Amanita virosa* (ECM)*, Artomyces pyxidatus* (W)*, Astraeus hygrometricus* (ECM)*, Calostoma japonicum* (ECM)*, Calvatia craniiformis* (L)*, Chalciporus piperatus* (ECM)*, Clitocybe fragrans* (L)*, Coltricia perennis* (ECM)*, Coltricia pusilla* (ECM)*, Coprinus micaceus* (W)*, Craterellus cornucopioides* (ECM)*, Cyclomyces fuscus* (W)*, Daedaleopsis tricolor* (W)*, Entoloma staurosporum* (L)*, Exdia glandulosa* (W)*, Hygrocybe aurantia* (L)*, Hygrocybe conica* (L)*, Hypholoma fasciculare* (W)*, Hypholoma sublateritium* (W)*, Inonotus mikadoi* (W)*, Laccaria bicolor* (ECM)*, Lactarius gracilis* (ECM)*, Lepista nuda* (L)*, Lyophyllum decastes* (L)*, Microporus affinis* (W)*, Mycena haematopoda* (W)*, Nigroporus vinosus* (W)*, Perenniporia ochroleuca* (W)*, Psathyrella piluliformis* (W)*, Pycnoporus coccineus* (W)*, Russula alboareolata* (ECM)*, Russula emetica* (ECM)*, Russula omiensis* (ECM)*, Russula rosea* (ECM)*, Schizophyllum commune* (W)*, Scleroderma verrucosum* (ECM)*, Stereopsis burtianum* (W)*, Stereum gausapatum* (W)*, Stereum hirsutum* (W)*, Trametes versicolor* (W)*, Xylobolus spectabilis* (W) |
| 14-Dec-03 | *Astraeus hygrometricus* (ECM)*, Calostoma japonicum* (ECM)*, Coltricia pusilla* (ECM)*, Coprinus atramentarius* (L)*, Craterellus cornucopioides* (ECM)*, Cyclomyces fuscus* (W)*, Entoloma staurosporum* (L)*, Exdia glandulosa* (W)*, Hydnangium carneum* (ECM)*, Hygrocybe aurantia* (L)*, Hypholoma fasciculare* (W)*, Inocybe cincinnata* (ECM)*, Inonotus mikadoi* (W)*, Inonotus xeranticus* (W)*, Lactarius gracilis* (ECM)*, Mycena amygdalina* (W)*, Neolecta vittelina* (L)*, Oligoporus caesius* (W)*, Polyporus alveolarius* (W)*, Psathyrella piluliformis* (W)*, Russula omiensis* (ECM)*, Schizophyllum commune* (W)*, Scleroderma verrucosum* (ECM)*, Stereum gausapatum* (W)*, Strobilurus stephanocystis (NA), Trametes hirsuta* (W)*, Trametes versicolor* (W)*, Xylobolus spectabilis* (W) |
| 25-Jan-04 | *Calostoma japonicum* (ECM)*, Ciborinia camelliae* (L)*, Coltricia pusilla* (ECM)*, Cyclomyces fuscus* (W)*, Daedaleopsis purpurea* (W)*, Daedaleopsis styracina* (W)*, Exdia glandulosa* (W)*, Hydnangium carneum* (ECM)*, Psathyrella piluliformis* (W)*, Pycnoporus coccineus* (W)*, Schizophyllum commune* (W)*, Trametes versicolor* (W)*, Tremella mesenterica* (W)*, Trichaptum elongatum* (W) |
| 29-Feb-04 | *Astraeus hygrometricus* (ECM)*, Auricularia auricula* (W)*, Auricularia polytricha* (W)*, Calostoma japonicum* (ECM)*, Ciborinia camelliae* (L)*, Cyclomyces fuscus* (W)*, Daedaleopsis styracina* (W)*, Elaphocordyceps longisegmentis (NA), Ganoderma applanatum* (W)*, Hydnangium carneum* (ECM)*, Hypoxylon truncatum* (W)*, Lenzites betulinus* (W)*, Mycena laevigata* (W)*, Panellus stypticus* (W)*, Phellinus gilvus* (W)*, Polyporus alveolarius* (W)*, Pycnoporus coccineus* (W)*, Russula omiensis* (ECM)*, Schizophyllum commune* (W)*, Trametes versicolor* (W)*, Tremella mesenterica* (W)*, Trichaptum biforme* (W) |
| 21-Mar-04 | *Ciborinia camelliae* (L)*, Coltricia pusilla* (ECM)*, Cyclomyces fuscus* (W)*, Daldinia concentrica* (W)*, Datronia mollis* (W)*, Entoloma staurosporum* (L)*, Exdia uvapassa* (W)*, Ganoderma applanatum* (W)*, Hydnangium carneum* (ECM)*, Hypholoma fasciculare* (W)*, Inonotus mikadoi* (W)*, Lentinula edodes* (W)*, Lenzites betulinus* (W)*, Lycoperdon perlatum* (L)*, Microporus affinis* (W)*, Oligoporus caesius* (W)*, Perenniporia ochroleuca* (W)*, Pleurotus ostreatus* (W)*, Polyporus alveolarius* (W)*, Psathyrella piluliformis* (W)*, Pycnoporus coccineus* (W)*, Russula omiensis* (ECM)*, Schizophyllum commune* (W)*, Trametes hirsuta* (W)*, Trametes versicolor* (W)*, Xylobolus spectabilis* (W) |
| 25-Apr-04 | *Auricularia auricula* (W)*, Camarops petersii* (W)*, Coltricia pusilla* (ECM)*, Coprinus micaceus* (W)*, Cordyceps heteropoda (NA), Cyclomyces fuscus* (W)*, Daedaleopsis purpurea* (W)*, Entoloma staurosporum* (L)*, Exdia glandulosa* (W)*, Hygrocybe aurantia* (L)*, Laetiporus versisporus* (W)*, Neolentinus lepideus* (W)*, Phellinus gilvus* (W)*, Psathyrella piluliformis* (W)*, Russula omiensis* (ECM)*, Schizophyllum commune* (W)*, Trametes versicolor* (W) |
| 27-Jun-04 | *Amanita farinosa* (ECM)*, Amanita fulva* (ECM)*, Amanita pantherina* (ECM)*, Amanita rubescens* (ECM)*, Amanita sychnopyramis f. subannulata* (ECM)*, Amanita vaginata var. punctata* (ECM)*, Amanita vaginata var. vaginata* (ECM)*, Amanita volvata* (ECM)*, Armillaria tabescens* (W)*, Astraeus hygrometricus* (ECM)*, Auricularia auricula* (W)*, Boletellus obscurecoccineus* (ECM)*, Boletus fraternus* (ECM)*, Bovista plumbea* (L)*, Collybia confluens* (L)*, Cordyceps annullata (NA), Cordyceps japonensis (NA), Cyclomyces fuscus* (W)*, Cyptotrama asprata* (W)*, Dicephalospora rufocornea* (W)*, Ganoderma applanatum* (W)*, Helvella ephippioides* (ECM)*, Hydnotrya tulasnei* (ECM)*, Inocybe asterospora* (ECM)*, Inocybe fastigiata* (ECM)*, Laccaria vinaceoavellanea* (ECM)*, Lactarius gerardii* (ECM)*, Lactarius gracilis* (ECM)*, Lactarius subvellereus* (ECM)*, Lactarius subzonarius* (ECM)*, Lepiota cristata* (L)*, Lepiota cygnea* (L)*, Marasmiellus nigripes* (W)*, Marasmius pulcherripes* (L)*, Megacollybia platyphylla* (W)*, Microporus affinis* (W)*, Neolentinus lepideus* (W)*, Nomuraea atypicola (NA), Omphalina epichysium* (L)*, Phylloporus bellus* (ECM)*, Pluteus atricapillus* (W)*, Psathyrella candolliana* (W)*, Pycnoporus coccineus* (W)*, Resupinatus trichotis* (W)*, Russula alboareolata* (ECM)*, Russula castanopsidis* (ECM)*, Russula eburneoareolata* (ECM)*, Russula foetens* (ECM)*, Russula kansaiensis* (ECM)*, Russula lilacea* (ECM)*, Russula mariae* (ECM)*, Russula pectinatoides* (ECM)*, Russula violeipes* (ECM)*, Scleroderma verrucosum* (ECM)*, Volvariella pusilla* (L)*, Xerocomus nigromaculatus* (ECM)*, Xeromphalina campanella* (W)*, Xerula pudens* (W)*, Xylobolus spectabilis* (W) |
| 29-Aug-04 | *Agaricus subrutilescens* (L)*, Agrocybe cylindracea* (W)*, Amanita ceciliae* (ECM)*, Amanita farinosa* (ECM)*, Amanita fuliginea* (ECM)*, Amanita griseofarinosa* (ECM)*, Amanita neoovoidea* (ECM)*, Amanita pantherina* (ECM)*, Amanita pseudoporphyria* (ECM)*, Amanita punctata* (ECM)*, Amanita rubescens* (ECM)*, Amanita similis* (ECM)*, Amanita spissacea* (ECM)*, Amanita sychnopyramis f. subannulata* (ECM)*, Amanita timida* (ECM)*, Amanita vaginata var. vaginata* (ECM)*, Amanita virosa* (ECM)*, Armillaria tabescens* (W)*, Aureoboletus thibetanus* (ECM)*, Austroboletus subvirens* (ECM)*, Boletellus emodensis* (ECM)*, Boletellus russellii* (ECM)*, Boletus fraternus* (ECM)*, Boletus granulopunctatus* (ECM)*, Boletus obscureumbrinus* (ECM)*, Boletus pseudocalopus* (ECM)*, Boletus reticulatus* (ECM)*, Boletus subvelutipes* (ECM)*, Boletus violaceofuscus* (ECM)*, Calvatia craniiformis* (L)*, Coltricia pusilla* (ECM)*, Cortinarius rubicundulus* (ECM)*, Craterellus cornucopioides* (ECM)*, Crepidotus badiofloccosus* (W)*, Dicephalospora rufocornea* (W)*, Entoloma murraii* (L)*, Gyroporus logicystidiatus* (ECM)*, Gyroporus punctatus* (ECM)*, Hydnangium carneum* (ECM)*, Hygrocybe conica* (L)*, Laccaria vinaceoavellanea* (ECM)*, Lactarius gerardii* (ECM)*, Laetiporus versisporus* (W)*, Leccinum extremiorientale* (ECM)*, Leucoagaricus rubrotinctus* (L)*, Lycoperdon perlatum* (L)*, Phylloporus bellus* (ECM)*, Phylloporus orientalis* (ECM)*, Pisolithus tinctorius* (ECM)*, Pulveroboletus auriflammeus* (ECM)*, Pulveroboletus ravenelii* (ECM)*, Pulveroboletus viridis* (ECM)*, Russula castanopsidis* (ECM)*, Russula japonica* (ECM)*, Russula kansaiensis* (ECM)*, Russula lilacea* (ECM)*, Russula peltereaui* (ECM)*, Russula virescens* (ECM)*, Sparassis crispa* (W)*, Strobilomyces seminudus* (ECM)*, Trametes versicolor* (W)*, Tylopilus alutaceoumbrinus* (ECM)*, Tylopilus argillaceus* (ECM)*, Tylopilus chromapes* (ECM)*, Tylopilus ferrugineus* (ECM)*, Tylopilus fumosipes* (ECM)*, Tylopilus nigerrimus* (ECM)*, Tylopilus nigropurpureus* (ECM)*, Tylopilus valens* (ECM)*, Xerocomus nigromaculatus* (ECM) |
| 26-Sep-04 | *Amanita farinosa* (ECM)*, Amanita fuliginea* (ECM)*, Amanita fulva* (ECM)*, Amanita pseudoporphyria* (ECM)*, Amanita sychnopyramis f. subannulata* (ECM)*, Amanita vaginata var. punctata* (ECM)*, Amanita vaginata var. vaginata* (ECM)*, Amanita virosa* (ECM)*, Armillaria tabescens* (W)*, Aureoboletus thibetanus* (ECM)*, Boletellus emodensis* (ECM)*, Boletus fraternus* (ECM)*, Boletus violaceofuscus* (ECM)*, Calostoma japonicum* (ECM)*, Cantharellus cinereus* (ECM)*, Collybia confluens* (L)*, Collybia peronata* (L)*, Cordyceps militaris (NA), Cortinarius mucosus* (ECM)*, Cyclomyces fuscus* (W)*, Cyptotrama asprata* (W)*, Dicephalospora rufocornea* (W)*, Grifola frondosa* (W)*, Hydnangium carneum* (ECM)*, Hypholoma fasciculare* (W)*, Kobayasia nipponica* (L)*, Laccaria vinaceoavellanea* (ECM)*, Lactarius gerardii* (ECM)*, Lactarius quietus* (ECM)*, Leccinum extremiorientale* (ECM)*, Lepiota cygnea* (L)*, Marasmiellus nigripes* (W)*, Marasmius siccus* (L)*, Megacollybia platyphylla* (W)*, Microporus vernicipes* (W)*, Mycena haematopoda* (W)*, Oligoporus caesius* (W)*, Panellus stypticus* (W)*, Paxillus atrotomentosus* (W)*, Paxillus cutisii* (W)*, Perenniporia ochroleuca* (W)*, Pholiota astragalina* (W)*, Pholiota malicola* (W)*, Phylloporus bellus* (ECM)*, Pycnoporus coccineus* (W)*, Ramariopsis helvola* (ECM)*, Russula alboareolata* (ECM)*, Russula castanopsidis* (ECM)*, Russula japonica* (ECM)*, Russula kansaiensis* (ECM)*, Russula lilacea* (ECM)*, Russula mariae* (ECM)*, Russula vesca* (ECM)*, Sarcodon scabrosus* (ECM)*, Scleroderma areolatum* (ECM)*, Scleroderma verrucosum* (ECM)*, Stereopsis burtianum* (W)*, Stereum ostrea* (W)*, Strobilomyces seminudus* (ECM)*, Trametes versicolor* (W)*, Tremella mesenterica* (W)*, Trichaptum elongatum* (W)*, Tylopilus argillaceus* (ECM)*, Tylopilus ballouii* (ECM)*, Tylopilus nigropurpureus* (ECM)*, Tyromyces chioneus* (W)*, Xerocomus parvulus* (ECM)*, Xerula pudens* (W)*, Xerula radicata* (W) |
| 24-Oct-04 | *Agaricus subrutilescens* (L)*, Amanita ibotengutake* (ECM)*, Amanita vaginata var. vaginata* (ECM)*, Collybia neofusipes* (L)*, Cortinarius bolaris* (ECM)*, Cortinarius galeroides* (ECM)*, Cortinarius salor* (ECM)*, Cyclomyces fuscus* (W)*, Hebeloma sacchariolens* (ECM)*, Hydnangium carneum* (ECM)*, Hygrocybe aurantia* (L)*, Inonotus vallatus* (W)*, Lactarius gracilis* (ECM)*, Lactarius quietus* (ECM)*, Laetiporus versisporus* (W)*, Lyophyllum decastes* (L)*, Mycena haematopoda* (W)*, Oligoporus caesius* (W)*, Phellinus gilvus* (W)*, Psilocybe argentipes* (L)*, Russula alboareolata* (ECM)*, Russula cyanoxantha* (ECM)*, Russula emetica* (ECM)*, Russula mariae* (ECM)*, Russula pectinatoides* (ECM)*, Russula rosea* (ECM)*, Stereopsis burtianum* (W)*, Stereum gausapatum* (W)*, Trametes versicolor* (W)*, Trichaptum biforme* (W) |
| 28-Nov-04 | *Artomyces colensoi* (W)*, Calostoma japonicum* (ECM)*, Coltricia pusilla* (ECM)*, Craterellus cornucopioides* (ECM)*, Cyclomyces fuscus* (W)*, Daedalea dickinsii* (W)*, Daedaleopsis styracina* (W)*, Ganoderma applanatum* (W)*, Hydnangium carneum* (ECM)*, Hygrocybe aurantia* (L)*, Hypholoma fasciculare* (W)*, Hypholoma sublateritium* (W)*, Ileodictyon gracile* (L)*, Lactarius camphoratus* (ECM)*, Lactarius gracilis* (ECM)*, Laetiporus sulphureus* (W)*, Lycoperdon spadiceum* (L)*, Nigroporus vinosus* (W)*, Polyporus alveolarius* (W)*, Psathyrella candolliana* (W)*, Pycnoporellus fulgens* (W)*, Russula mariae* (ECM)*, Russula omiensis* (ECM)*, Trametes versicolor* (W)*, Tremella mesenterica* (W)*, Trichaptum abietinum* (W)*, Xylobolus spectabilis* (W) |
| 26-Dec-04 | *Coltricia pusilla* (ECM)*, Cordyceps sphecocephala (NA), Cyclomyces fuscus* (W)*, Elaphocordyceps capitata (NA), Entoloma staurosporum* (L)*, Hydnangium carneum* (ECM)*, Hygrocybe aurantia* (L)*, Hymenochaete rubiginosa* (W)*, Hypholoma fasciculare* (W)*, Lycoperdon perlatum* (L)*, Lycoperdon spadiceum* (L)*, Mycena amygdalina* (W)*, Mycena haematopoda* (W)*, Mycena polygramma* (W)*, Oligoporus caesius* (W)*, Phellinus gilvus* (W)*, Piptoporus soloniensis* (W)*, Psathyrella piluliformis* (W)*, Pycnoporus coccineus* (W)*, Russula omiensis* (ECM)*, Scleroderma verrucosum* (ECM)*, Strobilurus stephanocystis (NA), Trametes versicolor* (W)*, Trichaptum abietinum* (W)*, Trichaptum biforme* (W) |
| 23-Jan-05 | *Calostoma japonicum* (ECM)*, Cordyceps heteropoda (NA), Cyclomyces fuscus* (W)*, Daedaleopsis purpurea* (W)*, Daedaleopsis styracina* (W)*, Elaphocordyceps longisegmentis (NA), Exdia glandulosa* (W)*, Ganoderma applanatum* (W)*, Hydnangium carneum* (ECM)*, Hypholoma fasciculare* (W)*, Loweporus pubertatis* (W)*, Lycoperdon spadiceum* (L)*, Mycena laevigata* (W)*, Oligoporus caesius* (W)*, Phellinus gilvus* (W)*, Phellinus robustus* (W)*, Polyporus alveolarius* (W)*, Psathyrella piluliformis* (W)*, Russula omiensis* (ECM)*, Trametes versicolor* (W)*, Trichaptum biforme* (W)*, Trichoglossum walteri* (L)*, Xylobolus spectabilis* (W) |
| 20-Feb-05 | *Antrodiella fragrans* (W)*, Auricularia auricula* (W)*, Calostoma japonicum* (ECM)*, Ciborinia camelliae* (L)*, Coltricia pusilla* (ECM)*, Cordyceps heteropoda (NA), Cortinarius galeroides* (ECM)*, Cyclomyces fuscus* (W)*, Daedaleopsis purpurea* (W)*, Daedaleopsis styracina* (W)*, Elaphocordyceps longisegmentis (NA), Exdia glandulosa* (W)*, Exdia uvapassa* (W)*, Ganoderma applanatum* (W)*, Hydnangium carneum* (ECM)*, Lentinula edodes* (W)*, Microporus vernicipes* (W)*, Mycena laevigata* (W)*, Phanerochaete crossa* (W)*, Phellinus gilvus* (W)*, Polyporus alveolarius* (W)*, Russula omiensis* (ECM)*, Trametes hirsuta* (W)*, Trametes versicolor* (W)*, Trichaptum biforme* (W) |
| 27-Mar-05 | *Auricularia auricula* (W)*, Caloscypha fulgens* (L)*, Ciborinia camelliae* (L)*, Cordyceps heteropoda (NA), Cyclomyces fuscus* (W)*, Daedaleopsis styracina* (W)*, Elaphocordyceps longisegmentis (NA), Entoloma staurosporum* (L)*, Exdia glandulosa* (W)*, Hydnangium carneum* (ECM)*, Hypholoma fasciculare* (W)*, Lentinula edodes* (W)*, Morchella conica* (L)*, Mycena galopus* (W)*, Phanerochaete crossa* (W)*, Phellinus gilvus* (W)*, Russula omiensis* (ECM)*, Trametes versicolor* (W)*, Tremella mesenterica* (W)*, Trichaptum biforme* (W) |
| 24-Apr-05 | *Cordyceps heteropoda (NA), Daedaleopsis purpurea* (W)*, Entoloma staurosporum* (L)*, Hydnochaete tabacinoides* (W)*, Tyromyces chioneus* (W) |
| 29-May-05 | *Cordyceps heteropoda (NA), Cortinarius subalboviolaceus* (ECM)*, Cyclomyces fuscus* (W)*, Exdia glandulosa* (W)*, Fistulina hepatica* (W)*, Galiella celebica* (W)*, Ganoderma applanatum* (W)*, Gerronema fibula* (L)*, Lactarius gracilis* (ECM)*, Lyophyllum decastes* (L)*, Neolentinus lepideus* (W)*, Pholiota highlandensis* (W)*, Polyporus alveolarius* (W)*, Pycnoporus coccineus* (W)*, Russula omiensis* (ECM)*, Trametes versicolor* (W)*, Xerula pudens* (W)*, Xylobolus spectabilis* (W) |
| 19-Jun-05 | *Boletellus shichianus* (ECM)*, Boletus fraternus* (ECM)*, Clavulina rugosa* (ECM)*, Cordyceps annullata (NA), Cordyceps japonensis (NA), Cortinarius alboviolaceus* (ECM)*, Cyclomyces fuscus* (W)*, Cyptotrama asprata* (W)*, Daedaleopsis purpurea* (W)*, Dicephalospora rufocornea* (W)*, Fistulina hepatica* (W)*, Galiella celebica* (W)*, Ganoderma applanatum* (W)*, Hygrocybe aurantia* (L)*, Inonotus sciurinus* (W)*, Lactarius gracilis* (ECM)*, Marasmius crinisequi* (L)*, Megacollybia platyphylla* (W)*, Microporus affinis* (W)*, Microporus subaffinis* (W)*, Pholiota highlandensis* (W)*, Pholiota terrestris* (W)*, Polyporus alveolarius* (W)*, Polyporus varius* (W)*, Pycnoporus coccineus* (W)*, Russula cyanoxantha* (ECM)*, Russula mariae* (ECM)*, Trichocoma paradoxa* (W)*, Tyromyces incarnatus* (W) |
| 10-Jul-05 | *Agrocybe cylindracea* (W)*, Amanita ceciliae* (ECM)*, Amanita farinosa* (ECM)*, Amanita pantherina* (ECM)*, Amanita pseudoporphyria* (ECM)*, Amanita sychnopyramis f. subannulata* (ECM)*, Armillaria tabescens* (W)*, Austroboletus fusisporus* (ECM)*, Boletus fraternus* (ECM)*, Boletus subvelutipes* (ECM)*, Calvatia craniiformis* (L)*, Chalciporus piperatus* (ECM)*, Clavaria purpurea* (L)*, Coltricia perennis* (ECM)*, Cordyceps annullata (NA), Cordyceps heteropoda (NA), Cortinarius galeroides* (ECM)*, Cyptotrama asprata* (W)*, Dicephalospora rufocornea* (W)*, Entoloma pulchellus* (L)*, Gerronema fibula* (L)*, Hebeloma sporliatum* (ECM)*, Hebeloma vinosophyllum* (ECM)*, Holtermannia corniformis* (W)*, Laccaria vinaceoavellanea* (ECM)*, Lactarius gerardii* (ECM)*, Lactarius gracilis* (ECM)*, Lactarius piperatus* (ECM)*, Lactarius scrobiculatus* (ECM)*, Lactarius subzonarius* (ECM)*, Lactarius volemus* (ECM)*, Marasmius leveilleanus* (L)*, Marasmius maximus* (L)*, Marasmius pulcherripes* (L)*, Microporus affinis* (W)*, Nomuraea atypicola (NA), Omphalina epichysium* (L)*, Phylloporus bellus* (ECM)*, Phylloporus orientalis* (ECM)*, Pluteus atricapillus* (W)*, Psathyrella candolliana* (W)*, Resupinatus applicatus* (W)*, Russula amoena* (ECM)*, Russula castanopsidis* (ECM)*, Russula cyanoxantha* (ECM)*, Russula kansaiensis* (ECM)*, Russula lilacea* (ECM)*, Russula mariae* (ECM)*, Russula peltereaui* (ECM)*, Russula sororia* (ECM)*, Russula violeipes* (ECM)*, Russula virescens* (ECM)*, Scleroderma areolatum* (ECM)*, Scleroderma reae* (ECM)*, Tremella mesenterica* (W)*, Tylopilus argillaceus* (ECM)*, Tylopilus ferrugineus* (ECM)*, Tylopilus fumosipes* (ECM)*, Xerocomus parvulus* (ECM)*, Xerula pudens* (W) |
| 28-Aug-05 | *Amanita citrina var. grisea* (ECM)*, Amanita griseofarinosa* (ECM)*, Amanita pseudoporphyria* (ECM)*, Amanita pseudovaginata* (ECM)*, Amanita punctata* (ECM)*, Amanita similis* (ECM)*, Amanita spissacea* (ECM)*, Amanita sychnopyramis f. subannulata* (ECM)*, Amanita vaginata var. vaginata* (ECM)*, Amanita verna* (ECM)*, Austroboletus fusisporus* (ECM)*, Boletellus emodensis* (ECM)*, Boletellus obscurecoccineus* (ECM)*, Boletus granulopunctatus* (ECM)*, Boletus ornatipes* (ECM)*, Boletus quercinus* (ECM)*, Boletus subcinnamomeus* (ECM)*, Boletus subvelutipes* (ECM)*, Boletus umbriniporus* (ECM)*, Calvatia craniiformis* (L)*, Coltricia pusilla* (ECM)*, Coprinus atramentarius* (L)*, Crepidotus applanatus* (W)*, Crepidotus mollis* (W)*, Cryptoporus volvatus* (W)*, Cyclomyces fuscus* (W)*, Fomitopsis pinicola* (W)*, Ganoderma lucidum* (W)*, Lactarius gerardii* (ECM)*, Leccinum hortonii* (ECM)*, Lenzites betulinus* (W)*, Neolentinus lepideus* (W)*, Perenniporia ochroleuca* (W)*, Pholiota malicola* (W)*, Phylloporus bellus* (ECM)*, Polyporus varius* (W)*, Psathyrella candolliana* (W)*, Pulveroboletus auriflammeus* (ECM)*, Pulveroboletus ravenelii* (ECM)*, Russula alboareolata* (ECM)*, Russula castanopsidis* (ECM)*, Russula cyanoxantha* (ECM)*, Russula mariae* (ECM)*, Russula pectinatoides* (ECM)*, Russula subnigricans* (ECM)*, Russula virescens* (ECM)*, Strobilomyces seminudus* (ECM)*, Thelephora terrestris* (ECM)*, Trametes versicolor* (W)*, Trichoglossum walteri* (L)*, Tylopilus alutaceoumbrinus* (ECM)*, Tylopilus argillaceus* (ECM)*, Tylopilus castaneiceps* (ECM)*, Tylopilus ferrugineus* (ECM)*, Tylopilus fumosipes* (ECM)*, Tylopilus nigerrimus* (ECM)*, Tylopilus nigropurpureus* (ECM)*, Xerocomus parvulus* (ECM)*, Xeromphalina campanella* (W)*, Xerula pudens* (W)*, Xerula radicata* (W)*, Xylobolus spectabilis* (W) |
| 11-Sep-05 | *Agaricus abruptibulbus* (L)*, Agaricus praeclaresquamosus* (L)*, Agaricus subrutilescens* (L)*, Agrocybe cylindracea* (W)*, Amanita ceciliae* (ECM)*, Amanita farinosa* (ECM)*, Amanita fuliginea* (ECM)*, Amanita pantherina* (ECM)*, Amanita rubescens* (ECM)*, Amanita spissacea* (ECM)*, Amanita sychnopyramis f. subannulata* (ECM)*, Amanita vaginata var. vaginata* (ECM)*, Amanita virosa* (ECM)*, Aureoboletus thibetanus* (ECM)*, Austroboletus fusisporus* (ECM)*, Boletus aokii* (ECM)*, Boletus sensibilis* (ECM)*, Calostoma japonicum* (ECM)*, Calvatia craniiformis* (L)*, Chalciporus piperatus* (ECM)*, Clavulina rugosa* (ECM)*, Collybia confluens* (L)*, Collybia peronata* (L)*, Coltricia cinnamomea* (ECM)*, Cortinarius rubicundulus* (ECM)*, Crepidotus applanatus* (W)*, Crepidotus badiofloccosus* (W)*, Cyclomyces fuscus* (W)*, Daedaleopsis styracina* (W)*, Entoloma album* (L)*, Entoloma pulchellus* (L)*, Galiella celebica* (W)*, Gyroporus punctatus* (ECM)*, Hebeloma vinosophyllum* (ECM)*, Hydnangium carneum* (ECM)*, Hygrocybe conica* (L)*, Inocybe geophylla* (ECM)*, Inocybe lutea* (ECM)*, Inocybe maculata* (ECM)*, Laccaria vinaceoavellanea* (ECM)*, Lactarius gerardii* (ECM)*, Lactarius piperatus* (ECM)*, Lactarius subvellereus* (ECM)*, Lactarius volemus* (ECM)*, Laeticorticium roseocarneum* (W)*, Lentinellus ursinus* (W)*, Leotia lubrica* (L)*, Lepiota cristata* (L)*, Leucoagaricus rubrotinctus* (L)*, Leucocoprinus fragilissimus* (L)*, Marasmius leveilleanus* (L)*, Marasmius pulcherripes* (L)*, Marasmius purpureostriatus* (L)*, Microporus affinis* (W)*, Neolentinus lepideus* (W)*, Penicilliopsis clavariaeformis (NA), Perenniporia fraxinea* (W)*, Perenniporia ochroleuca* (W)*, Phylloporus bellus* (ECM)*, Pisolithus tinctorius* (ECM)*, Pluteus atricapillus* (W)*, Polyporus alveolarius* (W)*, Pycnoporus coccineus* (W)*, Russula alboareolata* (ECM)*, Russula castanopsidis* (ECM)*, Russula cutefracta* (ECM)*, Russula japonica* (ECM)*, Russula lilacea* (ECM)*, Russula mariae* (ECM)*, Russula neoemetica* (ECM)*, Russula rosacea* (ECM)*, Russula subnigricans* (ECM)*, Russula vesca* (ECM)*, Russula virescens* (ECM)*, Scleroderma verrucosum* (ECM)*, Sparassis crispa* (W)*, Stereum ostrea* (W)*, Trametes versicolor* (W)*, Tremella mesenterica* (W)*, Tylopilus alutaceoumbrinus* (ECM)*, Tylopilus argillaceus* (ECM)*, Xerocomus nigromaculatus* (ECM)*, Xerocomus parvulus* (ECM) |
| 23-Oct-05 | *Agrocybe cylindracea* (W)*, Amanita citrina var. citrina* (ECM)*, Amanita ibotengutake* (ECM)*, Amanita vaginata var. vaginata* (ECM)*, Amanita virosa* (ECM)*, Auricularia polytricha* (W)*, Calostoma japonicum* (ECM)*, Calvatia craniiformis* (L)*, Chalciporus piperatus* (ECM)*, Collybia butyracea* (L)*, Coprinus atramentarius* (L)*, Cortinarius anomalus* (ECM)*, Cortinarius salor* (ECM)*, Cyclomyces fuscus* (W)*, Daedalea dickinsii* (W)*, Daedaleopsis styracina* (W)*, Daedaleopsis tricolor* (W)*, Dicephalospora rufocornea* (W)*, Entoloma murraii* (L)*, Fistulina hepatica* (W)*, Fomitopsis pinicola* (W)*, Ganoderma applanatum* (W)*, Gomphidius roseus* (ECM)*, Gymnopilus liquiritiae* (W)*, Hebeloma crustuliniforme* (ECM)*, Hebeloma radicosum* (ECM)*, Hebeloma sacchariolens* (ECM)*, Helvella atra* (ECM)*, Hygrocybe aurantia* (L)*, Hypholoma fasciculare* (W)*, Kobayasia nipponica* (L)*, Laccaria bicolor* (ECM)*, Lactarius akahatsu* (ECM)*, Lactarius chrysorrheus* (ECM)*, Lactarius gracilis* (ECM)*, Lactarius lividatus* (ECM)*, Lactarius quietus* (ECM)*, Laetiporus versisporus* (W)*, Lycoperdon spadiceum* (L)*, Lyophyllum decastes* (L)*, Marasmius leveilleanus* (L)*, Microporus affinis* (W)*, Mycena haematopoda* (W)*, Neolentinus lepideus* (W)*, Oligoporus caesius* (W)*, Omphalina epichysium* (L)*, Perenniporia ochroleuca* (W)*, Pholiota malicola* (W)*, Piptoporus soloniensis* (W)*, Pisolithus tinctorius* (ECM)*, Pleurotus ostreatus* (W)*, Polyporus alveolarius* (W)*, Pseudoclitocybe obbata* (L)*, Psilocybe argentipes* (L)*, Russula emetica* (ECM)*, Russula mariae* (ECM)*, Russula violeipes* (ECM)*, Schizophyllum commune* (W)*, Stereum hirsutum* (W)*, Suillus bovinus* (ECM)*, Suillus granulatus* (ECM)*, Trametes versicolor* (W)*, Tremella mesenterica* (W)*, Tylopilus castaneiceps* (ECM)*, Xeromphalina cauticinalis* (W)*, Xerula pudens* (W) |
| 20-Nov-05 | *Chroogomphus rutilus* (ECM)*, Coltricia montagnei* (ECM)*, Coltricia pusilla* (ECM)*, Coprinus atramentarius* (L)*, Cortinarius pseudopurpurascens* (ECM)*, Cyclomyces fuscus* (W)*, Daedaleopsis styracina* (W)*, Daedaleopsis tricolor* (W)*, Exdia glandulosa* (W)*, Fistulina hepatica* (W)*, Ganoderma applanatum* (W)*, Gymnopilus liquiritiae* (W)*, Hebeloma radicosum* (ECM)*, Hydnangium carneum* (ECM)*, Hygrocybe aurantia* (L)*, Hygrocybe conica* (L)*, Hypholoma fasciculare* (W)*, Hypholoma sublateritium* (W)*, Inonotus vallatus* (W)*, Lactarius chrysorrheus* (ECM)*, Lactarius gracilis* (ECM)*, Lepista nuda* (L)*, Lycoperdon perlatum* (L)*, Lycoperdon spadiceum* (L)*, Mycena amygdalina* (W)*, Mycena sanguinolenta* (W)*, Neolecta vittelina* (L)*, Oligoporus caesius* (W)*, Perenniporia ochroleuca* (W)*, Phaeolepiota aurea* (W)*, Phellinus gilvus* (W)*, Pleurotus ostreatus* (W)*, Polyporus alveolarius* (W)*, Polyporus varius* (W)*, Pycnoporus coccineus* (W)*, Russula atropurpurea* (ECM)*, Russula compacta* (ECM)*, Russula cyanoxantha* (ECM)*, Russula emetica* (ECM)*, Russula omiensis* (ECM)*, Scleroderma verrucosum* (ECM)*, Stereopsis burtianum* (W)*, Stereum gausapatum* (W)*, Strobilurus stephanocystis (NA), Suillus bovinus* (ECM)*, Trametes versicolor* (W)*, Tremella mesenterica* (W)*, Trichaptum biforme* (W)*, Xylobolus spectabilis* (W) |
| 11-Dec-05 | *Bjerkandera adusta* (W)*, Calostoma japonicum* (ECM)*, Coprinus atramentarius* (L)*, Cyclomyces fuscus* (W)*, Daedalea dickinsii* (W)*, Daedaleopsis styracina* (W)*, Daedaleopsis tricolor* (W)*, Exdia glandulosa* (W)*, Fistulina hepatica* (W)*, Hydnangium carneum* (ECM)*, Hypholoma fasciculare* (W)*, Hypholoma sublateritium* (W)*, Lactarius gracilis* (ECM)*, Lentinula edodes* (W)*, Lenzites betulinus* (W)*, Lycoperdon molle* (L)*, Lyophyllum decastes* (L)*, Microporus affinis* (W)*, Oligoporus caesius* (W)*, Panellus stypticus* (W)*, Perenniporia ochroleuca* (W)*, Phellinus gilvus* (W)*, Psathyrella piluliformis* (W)*, Russula omiensis* (ECM)*, Schizophyllum commune* (W)*, Sparassis crispa* (W)*, Stereum hirsutum* (W)*, Stereum ostrea* (W)*, Strobilurus ohshimae* (W)*, Trametes orientalis* (W)*, Trametes versicolor* (W)*, Trichaptum biforme* (W) |
| 29-Jan-06 | *Auricularia auricula* (W)*, Cordyceps sphecocephala (NA), Cyclomyces fuscus* (W)*, Daedaleopsis styracina* (W)*, Daedaleopsis tricolor* (W)*, Elaphocordyceps longisegmentis (NA), Exdia glandulosa* (W)*, Hydnangium carneum* (ECM)*, Hygrocybe aurantia* (L)*, Hypholoma fasciculare* (W)*, Lentinula edodes* (W)*, Mycena laevigata* (W)*, Perenniporia ochroleuca* (W)*, Phellinus gilvus* (W)*, Polyporus alveolarius* (W)*, Trametes versicolor* (W)*, Tremella mesenterica* (W)*, Trichaptum biforme* (W) |
| 19-Feb-06 | *Ciborinia camelliae* (L)*, Cyclomyces fuscus* (W)*, Daedalea dickinsii* (W)*, Daedaleopsis styracina* (W)*, Daedaleopsis tricolor* (W)*, Elaphocordyceps longisegmentis (NA), Exdia uvapassa* (W)*, Hydnangium carneum* (ECM)*, Hypholoma fasciculare* (W)*, Laetiporus versisporus* (W)*, Lentinula edodes* (W)*, Microporus vernicipes* (W)*, Mycena laevigata* (W)*, Perenniporia ochroleuca* (W)*, Phellinus setifer* (W)*, Polyporus alveolarius* (W)*, Psathyrella piluliformis* (W)*, Russula omiensis* (ECM)*, Trametes versicolor* (W)*, Trichaptum biforme* (W) |
| 19-Mar-06 | *Ciborinia camelliae* (L)*, Elaphocordyceps longisegmentis (NA), Entoloma staurosporum* (L)*, Exdia glandulosa* (W)*, Exdia uvapassa* (W)*, Geastrum triplex* (L)*, Hydnangium carneum* (ECM)*, Hypholoma fasciculare* (W)*, Lentinula edodes* (W)*, Mycena laevigata* (W)*, Polyporus alveolarius* (W)*, Psathyrella piluliformis* (W)*, Pycnoporus coccineus* (W)*, Russula omiensis* (ECM)*, Trametes versicolor* (W)*, Tremella brasiliensis* (W)*, Trichocoma paradoxa* (W)*, Tubaria furfuracea* (L) |
| 23-Apr-06 | *Auricularia auricula* (W)*, Ciborinia camelliae* (L)*, Coltricia perennis* (ECM)*, Coprinus micaceus* (W)*, Cordyceps heteropoda (NA), Cyclomyces fuscus* (W)*, Daedaleopsis styracina* (W)*, Elaphocordyceps longisegmentis (NA), Entoloma staurosporum* (L)*, Exdia glandulosa* (W)*, Ganoderma applanatum* (W)*, Helvella leucomelaena* (ECM)*, Hydnangium carneum* (ECM)*, Lactarius camphoratus* (ECM)*, Microporus affinis* (W)*, Morchella conica* (L)*, Russula omiensis* (ECM)*, Trametes versicolor* (W)*, Tremella mesenterica* (W) |
| 28-May-06 | *Amanita citrina var. grisea* (ECM)*, Amanita ibotengutake* (ECM)*, Auricularia auricula* (W)*, Cantharellus cinereus* (ECM)*, Coprinus plicatilis* (L)*, Cordyceps annullata (NA), Cordyceps heteropoda (NA), Cordyceps japonensis (NA), Cortinarius violaceus* (ECM)*, Cyptotrama asprata* (W)*, Fistulina hepatica* (W)*, Galiella celebica* (W)*, Hydnotrya tulasnei* (ECM)*, Hygrocybe aurantia* (L)*, Laccaria bicolor* (ECM)*, Lactarius akahatsu* (ECM)*, Lactarius camphoratus* (ECM)*, Lactarius gracilis* (ECM)*, Lactarius necator* (ECM)*, Lentinula edodes* (W)*, Lyophyllum decastes* (L)*, Mycena laevigata* (W)*, Phellinus gilvus* (W)*, Pluteus atricapillus* (W)*, Polyporus tuberaster* (W)*, Psathyrella velutina* (W)*, Russula alboareolata* (ECM)*, Russula rosea* (ECM)*, Stereum gausapatum* (W)*, Trametes versicolor* (W)*, Tremella mesenterica* (W)*, Xeromphalina campanella* (W)*, Xerula pudens* (W) |
| 18-Jun-06 | *Amanita vaginata var. vaginata* (ECM)*, Artomyces pyxidatus* (W)*, Auricularia auricula* (W)*, Boletellus shichianus* (ECM)*, Boletus umbriniporus* (ECM)*, Chalciporus piperatus* (ECM)*, Cordyceps annullata (NA), Cortinarius galeroides* (ECM)*, Cortinarius subalboviolaceus* (ECM)*, Cyptotrama asprata* (W)*, Dacrymyces palmatus* (W)*, Dicephalospora rufocornea* (W)*, Fistulina hepatica* (W)*, Galiella celebica* (W)*, Ganoderma applanatum* (W)*, Gerronema fibula* (L)*, Hydnotrya tulasnei* (ECM)*, Hygrocybe aurantia* (L)*, Lactarius akahatsu* (ECM)*, Lactarius gracilis* (ECM)*, Lyophyllum decastes* (L)*, Marasmius crinisequi* (L)*, Marasmius maximus* (L)*, Microporus affinis* (W)*, Neolentinus lepideus* (W)*, Omphalina epichysium* (L)*, Phellinus gilvus* (W)*, Polyporus arcularius* (W)*, Russula alboareolata* (ECM)*, Russula compacta* (ECM)*, Russula cyanoxantha* (ECM)*, Russula kansaiensis* (ECM)*, Russula laurocerasi* (ECM)*, Russula lilacea* (ECM)*, Russula mariae* (ECM)*, Russula vesca* (ECM)*, Russula virescens* (ECM)*, Stereum gausapatum* (W) |
| 23-Jul-06 | *Amanita ceciliae* (ECM)*, Amanita citrina var. grisea* (ECM)*, Amanita fuliginea* (ECM)*, Amanita imazekii* (ECM)*, Amanita japonica* (ECM)*, Amanita pulchella* (ECM)*, Amanita rubescens* (ECM)*, Amanita similis* (ECM)*, Amanita spissacea* (ECM)*, Amanita sychnopyramis f. subannulata* (ECM)*, Amanita virgineoides* (ECM)*, Armillaria tabescens* (W)*, Artomyces pyxidatus* (W)*, Astraeus hygrometricus* (ECM)*, Aureoboletus thibetanus* (ECM)*, Calvatia craniiformis* (L)*, Clavaria zollingeri* (L)*, Clavulina rugosa* (ECM)*, Coltricia pusilla* (ECM)*, Coprinus disseminatus* (W)*, Cordyceps annullata (NA), Cordyceps sobolifera (NA), Daedaleopsis styracina* (W)*, Fistulina hepatica* (W)*, Fomitopsis pinicola* (W)*, Ganoderma applanatum* (W)*, Hebeloma vinosophyllum* (ECM)*, Helvella atra* (ECM)*, Helvella ephippioides* (ECM)*, Hygrocybe cuspidata* (L)*, Inocybe lutea* (ECM)*, Inocybe maculata* (ECM)*, Laccaria vinaceoavellanea* (ECM)*, Lactarius piperatus* (ECM)*, Lactarius subvellereus* (ECM)*, Marasmius maximus* (L)*, Microporus affinis* (W)*, Nomuraea atypicola (NA), Omphalina epichysium* (L)*, Paxillus atrotomentosus* (W)*, Pisolithus tinctorius* (ECM)*, Polyporus badius* (W)*, Pulveroboletus auriflammeus* (ECM)*, Ramariopsis fusiformis* (ECM)*, Russula alboareolata* (ECM)*, Russula castanopsidis* (ECM)*, Russula cyanoxantha* (ECM)*, Russula densifolia* (ECM)*, Russula japonica* (ECM)*, Russula kansaiensis* (ECM)*, Russula laurocerasi* (ECM)*, Russula lilacea* (ECM)*, Russula mariae* (ECM)*, Russula nigricans* (ECM)*, Russula violeipes* (ECM)*, Russula virescens* (ECM)*, Scleroderma verrucosum* (ECM)*, Stereum ostrea* (W)*, Thelephora palmata* (ECM)*, Trametes versicolor* (W)*, Trichoglossum hirsutum* (L)*, Tylopilus argillaceus* (ECM)*, Tylopilus ballouii* (ECM)*, Xerula pudens* (W) |
| 27-Aug-06 | *Agaricus praeclaresquamosus* (L)*, Amanita fuliginea* (ECM)*, Amanita virosa* (ECM)*, Boletellus emodensis* (ECM)*, Calostoma japonicum* (ECM)*, Cyclomyces fuscus* (W)*, Daedaleopsis styracina* (W)*, Laetiporus versisporus* (W)*, Leccinum intusrubens* (ECM)*, Lenzites betulinus* (W)*, Marasmiellus chamaecyparidis* (W)*, Marasmius siccus* (L)*, Neolentinus lepideus* (W)*, Pluteus atricapillus* (W)*, Pluteus leoninus* (W)*, Russula cyanoxantha* (ECM)*, Russula pectinatoides* (ECM)*, Russula rosacea* (ECM)*, Russula subnigricans* (ECM)*, Trametes versicolor* (W)*, Xerocomus parvulus* (ECM)*, Xeromphalina campanella* (W)*, Xerula radicata* (W) |
| 24-Sep-06 | *Agaricus abruptibulbus* (L)*, Agrocybe cylindracea* (W)*, Amanita ceciliae* (ECM)*, Amanita citrina var. citrina* (ECM)*, Amanita esculenta* (ECM)*, Amanita flavipes* (ECM)*, Amanita fuliginea* (ECM)*, Amanita fulva* (ECM)*, Amanita ibotengutake* (ECM)*, Amanita pseudoporphyria* (ECM)*, Amanita rubescens* (ECM)*, Amanita rufoferruginea* (ECM)*, Amanita spissacea* (ECM)*, Amanita spreta* (ECM)*, Amanita sychnopyramis f. subannulata* (ECM)*, Amanita vaginata var. punctata* (ECM)*, Amanita vaginata var. vaginata* (ECM)*, Amanita virgineoides* (ECM)*, Amanita virosa* (ECM)*, Amanita volvata* (ECM)*, Armillaria tabescens* (W)*, Astraeus hygrometricus* (ECM)*, Boletellus emodensis* (ECM)*, Boletellus russellii* (ECM)*, Boletus obscureumbrinus* (ECM)*, Boletus reticulatus* (ECM)*, Boletus umbriniporus* (ECM)*, Calostoma japonicum* (ECM)*, Calvatia craniiformis* (L)*, Climacodon pulcherrimus* (W)*, Collybia peronata* (L)*, Cyclomyces fuscus* (W)*, Daedaleopsis tricolor* (W)*, Dicephalospora rufocornea* (W)*, Fomitopsis pinicola* (W)*, Fomitopsis spraguei* (W)*, Galiella celebica* (W)*, Ganoderma applanatum* (W)*, Ganoderma lucidum* (W)*, Gyroporus logicystidiatus* (ECM)*, Hypholoma fasciculare* (W)*, Inonotus mikadoi* (W)*, Inonotus vallatus* (W)*, Kobayasia nipponica* (L)*, Laccaria vinaceoavellanea* (ECM)*, Lactarius akahatsu* (ECM)*, Lactarius subvellereus* (ECM)*, Laetiporus versisporus* (W)*, Lentinula edodes* (W)*, Lepiota acutesquamosa* (L)*, Macrolepiota procena* (L)*, Neolentinus lepideus* (W)*, Paxillus atrotomentosus* (W)*, Pholiota malicola* (W)*, Pisolithus tinctorius* (ECM)*, Pluteus atricapillus* (W)*, Psathyrella piluliformis* (W)*, Psathyrella velutina* (W)*, Psilocybe subcaerulipes* (L)*, Pulveroboletus auriflammeus* (ECM)*, Pulveroboletus ravenelii* (ECM)*, Pycnoporus coccineus* (W)*, Russula alboareolata* (ECM)*, Russula castanopsidis* (ECM)*, Russula cyanoxantha* (ECM)*, Russula eburneoareolata* (ECM)*, Russula laurocerasi* (ECM)*, Russula pectinatoides* (ECM)*, Russula rubescens* (ECM)*, Russula vesca* (ECM)*, Russula violeipes* (ECM)*, Russula virescens* (ECM)*, Scleroderma areolatum* (ECM)*, Scleroderma verrucosum* (ECM)*, Strobilomyces seminudus* (ECM)*, Suillus granulatus* (ECM)*, Trametes versicolor* (W)*, Tylopilus argillaceus* (ECM)*, Tylopilus valens* (ECM) |
| 21-Oct-06 | *Agrocybe cylindracea* (W)*, Amanita abrupta* (ECM)*, Amanita ceciliae* (ECM)*, Amanita ibotengutake* (ECM)*, Amanita vaginata var. vaginata* (ECM)*, Amanita virosa* (ECM)*, Calvatia craniiformis* (L)*, Cyclomyces fuscus* (W)*, Daedalea dickinsii* (W)*, Dicephalospora rufocornea* (W)*, Fistulina hepatica* (W)*, Galiella celebica* (W)*, Gymnopilus liquiritiae* (W)*, Hebeloma sacchariolens* (ECM)*, Hydnangium carneum* (ECM)*, Hymenogaster pacificus* (ECM)*, Hypholoma fasciculare* (W)*, Kobayasia nipponica* (L)*, Laccaria vinaceoavellanea* (ECM)*, Lactarius akahatsu* (ECM)*, Lactarius lividatus* (ECM)*, Lactarius quietus* (ECM)*, Laetiporus sulphureus* (W)*, Laetiporus versisporus* (W)*, Lycoperdon echinatum* (L)*, Lycoperdon spadiceum* (L)*, Lyophyllum decastes* (L)*, Mycena haematopoda* (W)*, Oligoporus caesius* (W)*, Phallus impudicus* (L)*, Phellinus gilvus* (W)*, Pisolithus tinctorius* (ECM)*, Pleurotus pulmonarius* (W)*, Psilocybe subcaerulipes* (L)*, Russula cyanoxantha* (ECM)*, Russula foetens* (ECM)*, Russula kansaiensis* (ECM)*, Russula mariae* (ECM)*, Strobilurus ohshimae* (W)*, Suillus bovinus* (ECM)*, Suillus granulatus* (ECM)*, Trametes versicolor* (W)*, Xerula radicata* (W) |
| 26-Nov-06 | *Clitocybe fragrans* (L)*, Coprinus atramentarius* (L)*, Cyclomyces fuscus* (W)*, Daedalea dickinsii* (W)*, Hydnangium carneum* (ECM)*, Hypholoma fasciculare* (W)*, Hypholoma sublateritium* (W)*, Inonotus xeranticus* (W)*, Lactarius gracilis* (ECM)*, Lentinula edodes* (W)*, Lenzites betulinus* (W)*, Lycoperdon spadiceum* (L)*, Lyophyllum decastes* (L)*, Phaeolepiota aurea* (W)*, Phellinus gilvus* (W)*, Polyporus alveolarius* (W)*, Scleroderma verrucosum* (ECM)*, Trametes versicolor* (W) |
| 10-Dec-06 | *Antrodiella zonata* (W)*, Auricularia auricula* (W)*, Coltricia perennis* (ECM)*, Coprinus atramentarius* (L)*, Cordyceps heteropoda (NA), Cyclomyces fuscus* (W)*, Daedaleopsis styracina* (W)*, Entoloma staurosporum* (L)*, Exdia uvapassa* (W)*, Ganoderma applanatum* (W)*, Hydnangium carneum* (ECM)*, Hygrocybe aurantia* (L)*, Hypholoma fasciculare* (W)*, Hypholoma sublateritium* (W)*, Inonotus mikadoi* (W)*, Lentinellus ursinus* (W)*, Lentinula edodes* (W)*, Lyophyllum decastes* (L)*, Microporus affinis* (W)*, Mycena amygdalina* (W)*, Neolecta vittelina* (L)*, Oligoporus caesius* (W)*, Phellinus gilvus* (W)*, Pleurotus ostreatus* (W)*, Russula omiensis* (ECM)*, Schizophyllum commune* (W)*, Scleroderma verrucosum* (ECM)*, Trametes versicolor* (W)*, Tremella mesenterica* (W) |
| 28-Jan-07 | *Antrodiella zonata* (W)*, Ciborinia camelliae* (L)*, Cyclomyces fuscus* (W)*, Daedaleopsis tricolor* (W)*, Ganoderma applanatum* (W)*, Hydnangium carneum* (ECM)*, Hypholoma fasciculare* (W)*, Inonotus xeranticus* (W)*, Lentinula edodes* (W)*, Lenzites betulinus* (W)*, Perenniporia ochroleuca* (W)*, Phellinus gilvus* (W)*, Psathyrella piluliformis* (W)*, Pycnoporus coccineus* (W)*, Russula omiensis* (ECM)*, Schizophyllum commune* (W)*, Stereum gausapatum* (W)*, Trametes versicolor* (W)*, Xeromphalina campanella* (W)*, Xylobolus spectabilis* (W) |
| 25-Feb-07 | *Auricularia auricula* (W)*, Ciborinia camelliae* (L)*, Coltricia pusilla* (ECM)*, Cordyceps annullata (NA), Cyclomyces fuscus* (W)*, Daedaleopsis tricolor* (W)*, Ganoderma applanatum* (W)*, Hydnangium carneum* (ECM)*, Hypholoma fasciculare* (W)*, Lentinula edodes* (W)*, Lenzites betulinus* (W)*, Micromphale foetidum* (W)*, Mycena laevigata* (W)*, Phellinus gilvus* (W)*, Polyporus alveolarius* (W)*, Psathyrella piluliformis* (W)*, Russula omiensis* (ECM) |
| 25-Mar-07 | *Astraeus hygrometricus* (ECM)*, Auricularia auricula* (W)*, Ciborinia camelliae* (L)*, Cordyceps heteropoda (NA), Crepidotus mollis* (W)*, Elaphocordyceps longisegmentis (NA), Exdia glandulosa* (W)*, Hydnangium carneum* (ECM)*, Hypholoma fasciculare* (W)*, Lentinula edodes* (W)*, Lenzites betulinus* (W)*, Morchella conica* (L)*, Pleurotus ostreatus* (W)*, Russula omiensis* (ECM)*, Schizophyllum commune* (W)*, Stereum gausapatum* (W)*, Trametes versicolor* (W) |
| 29-Apr-07 | *Auricularia auricula* (W)*, Ciborinia camelliae* (L)*, Coprinus atramentarius* (L)*, Cordyceps heteropoda (NA), Daedaleopsis purpurea* (W)*, Entoloma staurosporum* (L)*, Galerina helvoliceps* (W)*, Hydnangium carneum* (ECM)*, Hygrocybe aurantia* (L)*, Hypholoma fasciculare* (W)*, Lactarius camphoratus* (ECM)*, Lentinula edodes* (W)*, Phellinus gilvus* (W)*, Pluteus atricapillus* (W) |
| 20-May-07 | *Amanita citrina var. grisea* (ECM)*, Amanita spissacea* (ECM)*, Auricularia auricula* (W)*, Auricularia polytricha* (W)*, Coprinus atramentarius* (L)*, Cordyceps ophioglossoides (NA), Cryptoporus volvatus* (W)*, Cyclomyces fuscus* (W)*, Daedaleopsis styracina* (W)*, Exdia glandulosa* (W)*, Ganoderma applanatum* (W)*, Lactarius gracilis* (ECM)*, Lyophyllum decastes* (L)*, Microporus affinis* (W)*, Pleurotus pulmonarius* (W)*, Psathyrella piluliformis* (W)*, Russula kansaiensis* (ECM)*, Schizophyllum commune* (W)*, Scutellinia scutellata* (W)*, Suillus luteus* (ECM)*, Trametes versicolor* (W) |
| 17-Jun-07 | *Agaricus subrutilescens* (L)*, Agrocybe cylindracea* (W)*, Agrocybe erebia* (W)*, Amanita farinosa* (ECM)*, Amanita ibotengutake* (ECM)*, Amanita pantherina* (ECM)*, Astraeus hygrometricus* (ECM)*, Boletellus shichianus* (ECM)*, Boletus pulverulentus* (ECM)*, Chalciporus piperatus* (ECM)*, Clavulina rugosa* (ECM)*, Coltricia cinnamomea* (ECM)*, Cordyceps annullata (NA), Cordyceps ophioglossoides (NA), Cortinarius galeroides* (ECM)*, Cortinarius subalboviolaceus* (ECM)*, Cortinarius violaceus* (ECM)*, Cryptoporus volvatus* (W)*, Cyclomyces fuscus* (W)*, Cyptotrama asprata* (W)*, Daedaleopsis styracina* (W)*, Dicephalospora rufocornea* (W)*, Fistulina hepatica* (W)*, Ganoderma applanatum* (W)*, Gerronema fibula* (L)*, Hydnangium carneum* (ECM)*, Hygrocybe aurantia* (L)*, Hypholoma fasciculare* (W)*, Inocybe asterospora* (ECM)*, Laccaria bicolor* (ECM)*, Laccaria vinaceoavellanea* (ECM)*, Lactarius camphoratus* (ECM)*, Lactarius gracilis* (ECM)*, Lactarius omphaliformis* (ECM)*, Microporus affinis* (W)*, Mycena haematopoda* (W)*, Neolentinus lepideus* (W)*, Oligoporus caesius* (W)*, Phylloporus bellus* (ECM)*, Pluteus leoninus* (W)*, Psilocybe subcaerulipes* (L)*, Pycnoporus coccineus* (W)*, Russula alboareolata* (ECM)*, Russula cyanoxantha* (ECM)*, Russula laurocerasi* (ECM)*, Russula mariae* (ECM)*, Russula sororia* (ECM)*, Russula violeipes* (ECM)*, Schizophyllum commune* (W)*, Stereum ostrea* (W)*, Trametes versicolor* (W)*, Tylopilus castaneiceps* (ECM)*, Xeromphalina campanella* (W)*, Xerula pudens* (W) |
| 22-Jul-07 | *Agrocybe cylindracea* (W)*, Amanita citrina var. citrina* (ECM)*, Amanita citrina var. grisea* (ECM)*, Amanita farinosa* (ECM)*, Amanita fuliginea* (ECM)*, Amanita fulva* (ECM)*, Amanita ibotengutake* (ECM)*, Amanita pseudoporphyria* (ECM)*, Amanita punctata* (ECM)*, Amanita rubescens* (ECM)*, Amanita similis* (ECM)*, Amanita spissacea* (ECM)*, Amanita sychnopyramis f. subannulata* (ECM)*, Amanita timida* (ECM)*, Amanita vaginata var. punctata* (ECM)*, Amanita vaginata var. vaginata* (ECM)*, Amanita virosa* (ECM)*, Amanita volvata* (ECM)*, Armillaria tabescens* (W)*, Astraeus hygrometricus* (ECM)*, Aureoboletus thibetanus* (ECM)*, Boletellus emodensis* (ECM)*, Boletellus russellii* (ECM)*, Boletellus shichianus* (ECM)*, Boletus aokii* (ECM)*, Boletus fraternus* (ECM)*, Boletus subcinnamomeus* (ECM)*, Boletus umbriniporus* (ECM)*, Callistosporium luteoolivaceum* (L)*, Cantharellus cinereus* (ECM)*, Clavaria zollingeri* (L)*, Clavulina cristata* (ECM)*, Clavulina rugosa* (ECM)*, Coltricia pusilla* (ECM)*, Coprinus disseminatus* (W)*, Cordyceps annullata (NA), Cordyceps sobolifera (NA), Cortinarius galeroides* (ECM)*, Cortinarius salor* (ECM)*, Craterellus cornucopioides* (ECM)*, Cyptotrama asprata* (W)*, Entoloma murraii* (L)*, Entoloma pulchellus* (L)*, Geastrum mirabile* (L)*, Gerronema fibula* (L)*, Gyroporus logicystidiatus* (ECM)*, Helvella atra* (ECM)*, Helvella lacunosa* (ECM)*, Hydnangium carneum* (ECM)*, Hygrocybe aurantia* (L)*, Hygrocybe cantharellus* (L)*, Hygrocybe conica* (L)*, Hygrocybe cuspidata* (L)*, Inocybe geophylla* (ECM)*, Inocybe lutea* (ECM)*, Kobayasia nipponica* (L)*, Laccaria vinaceoavellanea* (ECM)*, Lactarius akahatsu* (ECM)*, Lactarius castanopsidis* (ECM)*, Lactarius gerardii* (ECM)*, Lactarius quietus* (ECM)*, Lactarius subvellereus* (ECM)*, Leucoagaricus rubrotinctus* (L)*, Marasmius maximus* (L)*, Melanogaster intermedius* (ECM)*, Neolentinus lepideus* (W)*, Nomuraea atypicola (NA), Paxillus atrotomentosus* (W)*, Phylloporus bellus* (ECM)*, Phylloporus orientalis* (ECM)*, Psathyrella candolliana* (W)*, Psilocybe subcaerulipes* (L)*, Pycnoporus coccineus* (W)*, Russula castanopsidis* (ECM)*, Russula japonica* (ECM)*, Russula kansaiensis* (ECM)*, Russula laurocerasi* (ECM)*, Russula rubescens* (ECM)*, Russula viridirubrolimbata* (ECM)*, Stereum gausapatum* (W)*, Strobilomyces confusus* (ECM)*, Strobilomyces seminudus* (ECM)*, Strobilomyces strobilaceus* (ECM)*, Suillus granulatus* (ECM)*, Trichoglossum walteri* (L)*, Tylopilus alutaceoumbrinus* (ECM)*, Tylopilus argillaceus* (ECM)*, Tylopilus ballouii* (ECM)*, Tylopilus ferrugineus* (ECM)*, Tylopilus fumosipes* (ECM)*, Tylopilus nigerrimus* (ECM)*, Tylopilus nigropurpureus* (ECM)*, Tylopilus vinosobrunneus* (ECM)*, Volvariella hypopithys* (L)*, Xerocomus nigromaculatus* (ECM)*, Xerocomus parvulus* (ECM)*, Xerula pudens* (W) |
| 26-Aug-07 | *Amanita fuliginea* (ECM)*, Amanita sychnopyramis f. subannulata* (ECM)*, Amanita virosa* (ECM)*, Calostoma japonicum* (ECM)*, Coltricia pusilla* (ECM)*, Daedaleopsis styracina* (W)*, Dicephalospora rufocornea* (W)*, Hydnangium carneum* (ECM)*, Laetiporus versisporus* (W)*, Leccinum intusrubens* (ECM)*, Polyporus varius* (W)*, Psathyrella piluliformis* (W)*, Pycnoporus coccineus* (W)*, Russula subnigricans* (ECM)*, Trametes versicolor* (W)*, Xerocomus chrysenteron* (ECM) |
| 23-Sep-07 | *Agaricus abruptibulbus* (L)*, Agrocybe cylindracea* (W)*, Amanita citrina var. citrina* (ECM)*, Amanita farinosa* (ECM)*, Amanita fuliginea* (ECM)*, Amanita fulva* (ECM)*, Amanita hongoi* (ECM)*, Amanita neoovoidea* (ECM)*, Amanita pantherina* (ECM)*, Amanita pseudoporphyria* (ECM)*, Amanita punctata* (ECM)*, Amanita rubescens* (ECM)*, Amanita rufoferruginea* (ECM)*, Amanita spissacea* (ECM)*, Amanita sychnopyramis f. subannulata* (ECM)*, Amanita timida* (ECM)*, Amanita vaginata var. punctata* (ECM)*, Amanita vaginata var. vaginata* (ECM)*, Amanita virgineoides* (ECM)*, Amanita virosa* (ECM)*, Amanita volvata* (ECM)*, Armillaria tabescens* (W)*, Aureoboletus thibetanus* (ECM)*, Austroboletus fusisporus* (ECM)*, Austroboletus subvirens* (ECM)*, Boletellus emodensis* (ECM)*, Boletellus obscurecoccineus* (ECM)*, Boletellus russellii* (ECM)*, Boletus aokii* (ECM)*, Boletus griseus* (ECM)*, Boletus obscureumbrinus* (ECM)*, Boletus ornatipes* (ECM)*, Boletus pseudocalopus* (ECM)*, Boletus reticulatus* (ECM)*, Boletus subcinnamomeus* (ECM)*, Boletus subvelutipes* (ECM)*, Boletus umbriniporus* (ECM)*, Calvatia craniiformis* (L)*, Coltricia pusilla* (ECM)*, Cortinarius rubicundulus* (ECM)*, Craterellus cornucopioides* (ECM)*, Crepidotus mollis* (W)*, Cyclomyces fuscus* (W)*, Daedalea dickinsii* (W)*, Dicephalospora rufocornea* (W)*, Entoloma album* (L)*, Entoloma murraii* (L)*, Fistulina hepatica* (W)*, Ganoderma applanatum* (W)*, Gyroporus logicystidiatus* (ECM)*, Gyroporus punctatus* (ECM)*, Hydnangium carneum* (ECM)*, Hygrocybe conica* (L)*, Inocybe lutea* (ECM)*, Inocybe maculata* (ECM)*, Kobayasia nipponica* (L)*, Laccaria vinaceoavellanea* (ECM)*, Lactarius akahatsu* (ECM)*, Leccinum extremiorientale* (ECM)*, Leccinum intusrubens* (ECM)*, Microporus affinis* (W)*, Neolentinus lepideus* (W)*, Oligoporus caesius* (W)*, Paxillus atrotomentosus* (W)*, Perenniporia minutissima* (W)*, Phellinus gilvus* (W)*, Phylloporus bellus* (ECM)*, Phylloporus orientalis* (ECM)*, Pisolithus tinctorius* (ECM)*, Psilocybe subcaerulipes* (L)*, Pulveroboletus auriflammeus* (ECM)*, Pulveroboletus ravenelii* (ECM)*, Russula castanopsidis* (ECM)*, Russula cyanoxantha* (ECM)*, Russula eburneoareolata* (ECM)*, Russula foetens* (ECM)*, Russula japonica* (ECM)*, Russula nigricans* (ECM)*, Russula pectinatoides* (ECM)*, Russula rosacea* (ECM)*, Russula sororia* (ECM)*, Russula subnigricans* (ECM)*, Russula vesca* (ECM)*, Russula violeipes* (ECM)*, Stereum ostrea* (W)*, Strobilomyces confusus* (ECM)*, Strobilomyces seminudus* (ECM)*, Suillus granulatus* (ECM)*, Trametes versicolor* (W)*, Tylopilus alutaceoumbrinus* (ECM)*, Tylopilus argillaceus* (ECM)*, Tylopilus castaneiceps* (ECM)*, Tylopilus ferrugineus* (ECM)*, Tylopilus fumosipes* (ECM)*, Tylopilus nigerrimus* (ECM)*, Tylopilus valens* (ECM)*, Volvariella subtaylori* (L)*, Xerocomus nigromaculatus* (ECM)*, Xerocomus subtomentosus* (ECM)*, Xerula radicata* (W) |
| 21-Oct-07 | *Agaricus subrutilescens* (L)*, Agrocybe cylindracea* (W)*, Amanita ibotengutake* (ECM)*, Amanita punctata* (ECM)*, Amanita vaginata var. vaginata* (ECM)*, Armillaria tabescens* (W)*, Boletus subcinnamomeus* (ECM)*, Calostoma japonicum* (ECM)*, Collybia neofusipes* (L)*, Coprinus atramentarius* (L)*, Craterellus cornucopioides* (ECM)*, Crinipellis stipitaria* (L)*, Cyclomyces fuscus* (W)*, Daedaleopsis styracina* (W)*, Dicephalospora rufocornea* (W)*, Entoloma murraii* (L)*, Fistulina hepatica* (W)*, Fomitopsis pinicola* (W)*, Galiella celebica* (W)*, Ganoderma applanatum* (W)*, Ganoderma lucidum* (W)*, Gymnopilus liquiritiae* (W)*, Hydnangium carneum* (ECM)*, Hygrocybe aurantia* (L)*, Hypholoma fasciculare* (W)*, Inocybe lutea* (ECM)*, Inocybe maculata* (ECM)*, Kobayasia nipponica* (L)*, Laccaria bicolor* (ECM)*, Lactarius akahatsu* (ECM)*, Lactarius lividatus* (ECM)*, Lactarius quietus* (ECM)*, Laetiporus versisporus* (W)*, Lentinula edodes* (W)*, Lenzites betulinus* (W)*, Lycoperdon spadiceum* (L)*, Lyophyllum decastes* (L)*, Mycena haematopoda* (W)*, Neolentinus lepideus* (W)*, Oligoporus caesius* (W)*, Phellinus gilvus* (W)*, Pholiota malicola* (W)*, Psathyrella candolliana* (W)*, Psathyrella piluliformis* (W)*, Psilocybe subcaerulipes* (L)*, Russula cyanoxantha* (ECM)*, Russula pectinatoides* (ECM)*, Russula sororia* (ECM)*, Stereopsis burtianum* (W)*, Stereum hirsutum* (W)*, Suillus granulatus* (ECM)*, Trametes versicolor* (W)*, Tremella mesenterica* (W)*, Tylopilus valens* (ECM) |
| 25-Nov-07 | *Astraeus hygrometricus* (ECM)*, Calostoma japonicum* (ECM)*, Clitocybe fragrans* (L)*, Coltricia pusilla* (ECM)*, Cortinarius alboviolaceus* (ECM)*, Cyclomyces fuscus* (W)*, Daedalea dickinsii* (W)*, Daedaleopsis purpurea* (W)*, Daedaleopsis styracina* (W)*, Entoloma japonicus* (L)*, Entoloma papillatus* (L)*, Galerina heterocystis* (L)*, Hydnangium carneum* (ECM)*, Hygrocybe aurantia* (L)*, Hypholoma fasciculare* (W)*, Hypholoma sublateritium* (W)*, Isaria takamizusanensis (NA), Lactarius gracilis* (ECM)*, Laetiporus sulphureus* (W)*, Laetiporus versisporus* (W)*, Lentinula edodes* (W)*, Loweporus tephroporus* (W)*, Lycoperdon spadiceum* (L)*, Lyophyllum decastes* (L)*, Mycena haematopoda* (W)*, Phellinus gilvus* (W)*, Pleurotus pulmonarius* (W)*, Psathyrella delineata* (W)*, Psathyrella piluliformis* (W)*, Russula alboareolata* (ECM)*, Russula mariae* (ECM)*, Russula omiensis* (ECM)*, Scleroderma verrucosum* (ECM)*, Trametes versicolor* (W)*, Tremella mesenterica* (W) |
| 9-Dec-07 | *Cyclomyces fuscus* (W)*, Hydnangium carneum* (ECM)*, Hygrocybe aurantia* (L)*, Hypholoma fasciculare* (W)*, Hypholoma sublateritium* (W)*, Jansia boninensis* (L)*, Lepista nuda* (L)*, Lycoperdon spadiceum* (L)*, Lyophyllum decastes* (L)*, Mycena haematopoda* (W)*, Trametes versicolor* (W) |
| 27-Jan-08 | *Antrodiella gypsea* (W)*, Antrodiella zonata* (W)*, Auricularia auricula* (W)*, Cyclomyces fuscus* (W)*, Daedaleopsis styracina* (W)*, Exdia glandulosa* (W)*, Exdia uvapassa* (W)*, Ganoderma applanatum* (W)*, Hydnangium carneum* (ECM)*, Hypholoma fasciculare* (W)*, Marasmiellus nigripes* (W)*, Microporus affinis* (W)*, Phellinus gilvus* (W)*, Pisolithus tinctorius* (ECM)*, Pleurotus ostreatus* (W)*, Polyporus alveolarius* (W)*, Porodisculus pendulus* (W)*, Russula omiensis* (ECM)*, Trametes versicolor* (W)*, Tremella mesenterica* (W)*, Xylobolus spectabilis* (W) |
| 24-Feb-08 | *Hydnangium carneum* (ECM)*, Lentinula edodes* (W)*, Psathyrella delineata* (W)*, Trametes versicolor* (W)*, Tremella mesenterica* (W)*, Xeromphalina campanella* (W) |
| 23-Mar-08 | *Ciborinia camelliae* (L)*, Coltricia pusilla* (ECM)*, Cordyceps heteropoda (NA), Cyclomyces fuscus* (W)*, Daedaleopsis styracina* (W)*, Elaphocordyceps longisegmentis (NA), Entoloma staurosporum* (L)*, Exdia glandulosa* (W)*, Exdia uvapassa* (W)*, Ganoderma applanatum* (W)*, Hypholoma fasciculare* (W)*, Lentinula edodes* (W)*, Lenzites betulinus* (W)*, Loweporus pubertatis* (W)*, Marasmius crinisequi* (L)*, Morchella conica* (L)*, Phellinus gilvus* (W)*, Psathyrella piluliformis* (W)*, Pycnoporus coccineus* (W)*, Russula omiensis* (ECM)*, Trametes versicolor* (W)*, Tricholoma ustale* (ECM)*, Xeromphalina campanella* (W) |
| 27-Apr-08 | *Artomyces pyxidatus* (W)*, Auricularia auricula* (W)*, Coprinus micaceus* (W)*, Cordyceps heteropoda (NA), Cordyceps japonensis (NA), Cordyceps ophioglossoides (NA), Daedaleopsis purpurea* (W)*, Elaphocordyceps longisegmentis (NA), Entoloma staurosporum* (L)*, Hydnotrya tulasnei* (ECM)*, Hygrocybe aurantia* (L)*, Hypholoma fasciculare* (W)*, Lactarius camphoratus* (ECM)*, Laetiporus versisporus* (W)*, Lentinula edodes* (W)*, Lenzites betulinus* (W)*, Morchella conica* (L)*, Mycena galopus* (W)*, Mycena sanguinolenta* (W)*, Phellinus gilvus* (W)*, Phellinus setifer* (W)*, Pluteus atricapillus* (W)*, Russula omiensis* (ECM)*, Stereum ostrea* (W)*, Trametes hirsuta* (W)*, Trametes versicolor* (W) |
| 18-May-08 | *Cordyceps heteropoda (NA), Dicephalospora rufocornea* (W)*, Fistulina hepatica* (W)*, Galiella celebica* (W)*, Hygrocybe aurantia* (L)*, Lactarius gracilis* (ECM)*, Laeticorticium roseocarneum* (W)*, Lentinula edodes* (W)*, Neolentinus lepideus* (W)*, Phellinus gilvus* (W)*, Polyporus arcularius* (W)*, Russula omiensis* (ECM) |
| 27-Jul-08 | *Amanita citrina var. citrina* (ECM)*, Amanita citrina var. grisea* (ECM)*, Amanita lutescens* (ECM)*, Amanita pseudoporphyria* (ECM)*, Amanita punctata* (ECM)*, Amanita rufoferruginea* (ECM)*, Amanita spissacea* (ECM)*, Amanita timida* (ECM)*, Amanita volvata* (ECM)*, Armillaria tabescens* (W)*, Aureoboletus thibetanus* (ECM)*, Boletus auripes* (ECM)*, Boletus granulopunctatus* (ECM)*, Boletus subvelutipes* (ECM)*, Boletus umbriniporus* (ECM)*, Coltricia cinnamomea* (ECM)*, Coprinus atramentarius* (L)*, Cordyceps sphecocephala (NA), Cyclomyces fuscus* (W)*, Ganoderma applanatum* (W)*, Gymnopilus aeruginosus* (W)*, Inonotus mikadoi* (W)*, Laccaria vinaceoavellanea* (ECM)*, Leccinum extremiorientale* (ECM)*, Loweporus pubertatis* (W)*, Megacollybia platyphylla* (W)*, Microporus affinis* (W)*, Neolentinus lepideus* (W)*, Nomuraea atypicola (NA), Paxillus atrotomentosus* (W)*, Perenniporia ochroleuca* (W)*, Phylloporus bellus* (ECM)*, Polyporus badius* (W)*, Psilocybe subcaerulipes* (L)*, Pycnoporus coccineus* (W)*, Russula alboareolata* (ECM)*, Russula castanopsidis* (ECM)*, Russula japonica* (ECM)*, Tylopilus argillaceus* (ECM)*, Tylopilus ferrugineus* (ECM)*, Tylopilus fumosipes* (ECM)*, Tylopilus valens* (ECM)*, Xerocomus chrysenteron* (ECM)*, Xerocomus nigromaculatus* (ECM)*, Xerocomus parvulus* (ECM)*, Xerula pudens* (W)*, Xylobolus spectabilis* (W) |
| 31-Aug-08 | *Agaricus abruptibulbus* (L)*, Agaricus praeclaresquamosus* (L)*, Agrocybe cylindracea* (W)*, Amanita ceciliae* (ECM)*, Amanita citrina var. grisea* (ECM)*, Amanita farinosa* (ECM)*, Amanita fuliginea* (ECM)*, Amanita oberwinklerana* (ECM)*, Amanita pseudoporphyria* (ECM)*, Amanita vaginata var. vaginata* (ECM)*, Armillaria tabescens* (W)*, Austroboletus fusisporus* (ECM)*, Boletus ornatipes* (ECM)*, Calostoma japonicum* (ECM)*, Calvatia craniiformis* (L)*, Chalciporus piperatus* (ECM)*, Chlorophyllum molybdites* (L)*, Collybia dryophila* (L)*, Crepidotus applanatus* (W)*, Crepidotus mollis* (W)*, Cyptotrama asprata* (W)*, Daedaleopsis styracina* (W)*, Daedaleopsis tricolor* (W)*, Dicephalospora rufocornea* (W)*, Ganoderma applanatum* (W)*, Ganoderma lucidum* (W)*, Geastrum mirabile* (L)*, Hebeloma vinosophyllum* (ECM)*, Hydnangium carneum* (ECM)*, Inonotus vallatus* (W)*, Isaria takamizusanensis (NA), Laccaria laccata* (ECM)*, Laccaria vinaceoavellanea* (ECM)*, Lactarius subvellereus* (ECM)*, Lentinula edodes* (W)*, Leucoagaricus rubrotinctus* (L)*, Leucocoprinus fragilissimus* (L)*, Marasmiellus candidus* (W)*, Microporus affinis* (W)*, Nidula niveo-tomentosa* (W)*, Omphalina epichysium* (L)*, Penicilliopsis clavariaeformis (NA), Perenniporia ochroleuca* (W)*, Pholiota malicola* (W)*, Phylloporus bellus* (ECM)*, Pleurotus pulmonarius* (W)*, Pluteus leoninus* (W)*, Psathyrella candolliana* (W)*, Psathyrella piluliformis* (W)*, Russula alboareolata* (ECM)*, Russula amoena* (ECM)*, Russula crustosa* (ECM)*, Russula japonica* (ECM)*, Russula kansaiensis* (ECM)*, Russula lilacea* (ECM)*, Russula subnigricans* (ECM)*, Russula virescens* (ECM)*, Schizophyllum commune* (W)*, Tremella foliacea* (W)*, Tricholoma vaccinum* (ECM)*, Tylopilus ferrugineus* (ECM)*, Tylopilus valens* (ECM)*, Xerocomus parvulus* (ECM)*, Xerula radicata* (W) |
| 28-Sep-08 | *Agaricus abruptibulbus* (L)*, Amanita fuliginea* (ECM)*, Amanita oberwinklerana* (ECM)*, Amanita sychnopyramis f. subannulata* (ECM)*, Amanita virosa* (ECM)*, Amanita volvata* (ECM)*, Antrodiella zonata* (W)*, Astraeus hygrometricus* (ECM)*, Aureoboletus thibetanus* (ECM)*, Boletus fraternus* (ECM)*, Boletus laetissimus* (ECM)*, Calostoma japonicum* (ECM)*, Calvatia craniiformis* (L)*, Chalciporus piperatus* (ECM)*, Chlorophyllum molybdites* (L)*, Clavaria aurantio-cinnabarina* (L)*, Collybia butyracea* (L)*, Collybia peronata* (L)*, Coltricia pusilla* (ECM)*, Coprinus disseminatus* (W)*, Cortinarius alboviolaceus* (ECM)*, Cortinarius aureobrunneus* (ECM)*, Cortinarius salor* (ECM)*, Cryptoporus volvatus* (W)*, Cyclomyces fuscus* (W)*, Cyptotrama asprata* (W)*, Daedalea dickinsii* (W)*, Daedaleopsis purpurea* (W)*, Dicephalospora rufocornea* (W)*, Echinochaete ruficeps* (W)*, Entoloma quadratus* (L)*, Galerina heterocystis* (L)*, Ganoderma applanatum* (W)*, Ganoderma lucidum* (W)*, Gloeostereum incarnatum* (W)*, Gymnopilus liquiritiae* (W)*, Hydnangium carneum* (ECM)*, Hypholoma fasciculare* (W)*, Inonotus vallatus* (W)*, Laccaria vinaceoavellanea* (ECM)*, Lactarius akahatsu* (ECM)*, Lactarius gracilis* (ECM)*, Lactarius subvellereus* (ECM)*, Laetiporus versisporus* (W)*, Lentinula edodes* (W)*, Lenzites betulinus* (W)*, Leucocoprinus fragilissimus* (L)*, Lyophyllum decastes* (L)*, Microporus affinis* (W)*, Mycena haematopoda* (W)*, Nidula niveo-tomentosa* (W)*, Oligoporus caesius* (W)*, Panellus stypticus* (W)*, Paxillus atrotomentosus* (W)*, Penicilliopsis clavariaeformis (NA), Perenniporia ochroleuca* (W)*, Phellinus gilvus* (W)*, Phellodon niger* (ECM)*, Phylloporus bellus* (ECM)*, Polyporus badius* (W)*, Psathyrella candolliana* (W)*, Psathyrella piluliformis* (W)*, Pycnoporus coccineus* (W)*, Russula alboareolata* (ECM)*, Russula castanopsidis* (ECM)*, Russula cyanoxantha* (ECM)*, Russula eburneoareolata* (ECM)*, Russula japonica* (ECM)*, Russula nigricans* (ECM)*, Russula vesca* (ECM)*, Russula violeipes* (ECM)*, Scleroderma areolatum* (ECM)*, Stereopsis burtianum* (W)*, Suillus granulatus* (ECM)*, Tremella mesenterica* (W)*, Tylopilus valens* (ECM)*, Xerocomus chrysenteron* (ECM)*, Xerocomus subtomentosus* (ECM)*, Xerula pudens* (W)*, Xylobolus spectabilis* (W) |
| 19-Oct-08 | *Agaricus abruptibulbus* (L)*, Amanita citrina var. citrina* (ECM)*, Amanita esculenta* (ECM)*, Amanita ibotengutake* (ECM)*, Amanita oberwinklerana* (ECM)*, Amanita rubescens* (ECM)*, Amanita vaginata var. vaginata* (ECM)*, Amanita volvata* (ECM)*, Aureoboletus thibetanus* (ECM)*, Boletus subvelutipes* (ECM)*, Boletus umbriniporus* (ECM)*, Callistosporium luteoolivaceum* (L)*, Calostoma japonicum* (ECM)*, Cantharellus infundibuliformis* (ECM)*, Chalciporus piperatus* (ECM)*, Collybia maculata* (L)*, Coprinus micaceus* (W)*, Craterellus cornucopioides* (ECM)*, Cyclomyces fuscus* (W)*, Flammulina velutipes* (W)*, Ganoderma applanatum* (W)*, Helvella atra* (ECM)*, Helvella ephippium* (ECM)*, Hydnangium carneum* (ECM)*, Hygrocybe aurantia* (L)*, Hygrocybe conica* (L)*, Hypholoma fasciculare* (W)*, Inocybe maculata* (ECM)*, Laccaria bicolor* (ECM)*, Laccaria vinaceoavellanea* (ECM)*, Lactarius akahatsu* (ECM)*, Lactarius gracilis* (ECM)*, Lactarius lividatus* (ECM)*, Lactarius quietus* (ECM)*, Lentinellus ursinus* (W)*, Lenzites betulinus* (W)*, Lepista nuda* (L)*, Lycoperdon spadiceum* (L)*, Lyophyllum decastes* (L)*, Nigroporus vinosus* (W)*, Oligoporus caesius* (W)*, Penicilliopsis clavariaeformis (NA), Perenniporia ochroleuca* (W)*, Pholiota malicola* (W)*, Pisolithus tinctorius* (ECM)*, Polyporus alveolarius* (W)*, Psilocybe subcaerulipes* (L)*, Russula alboareolata* (ECM)*, Russula castanopsidis* (ECM)*, Russula cyanoxantha* (ECM)*, Russula mariae* (ECM)*, Russula pectinatoides* (ECM)*, Russula sororia* (ECM)*, Stereopsis burtianum* (W)*, Suillus bovinus* (ECM)*, Suillus luteus* (ECM)*, Trametes versicolor* (W)*, Tylopilus castaneiceps* (ECM)*, Xerocomus nigromaculatus* (ECM) |
| 30-Nov-08 | *Calostoma japonicum* (ECM)*, Camarophyllus pratensis* (L)*, Collybia butyracea* (L)*, Cyclomyces fuscus* (W)*, Hydnangium carneum* (ECM)*, Hygrocybe aurantia* (L)*, Hypholoma fasciculare* (W)*, Hypholoma sublateritium* (W)*, Inonotus mikadoi* (W)*, Inonotus xeranticus* (W)*, Lactarius gracilis* (ECM)*, Lentinellus ursinus* (W)*, Lentinula edodes* (W)*, Lenzites betulinus* (W)*, Lepista nuda* (L)*, Linderia bicolumnata* (L)*, Lyophyllum decastes* (L)*, Microporus affinis* (W)*, Mycena amygdalina* (W)*, Mycena polygramma* (W)*, Neolecta vittelina* (L)*, Panellus stypticus* (W)*, Perenniporia minutissima* (W)*, Perenniporia ochroleuca* (W)*, Phellinus gilvus* (W)*, Pleurotus pulmonarius* (W)*, Psathyrella piluliformis* (W)*, Russula cyanoxantha* (ECM)*, Russula omiensis* (ECM)*, Russula rosea* (ECM)*, Stereum gausapatum* (W)*, Strobilurus ohshimae* (W)*, Strobilurus stephanocystis (NA), Trametes versicolor* (W) |
| 13-Dec-08 | *Auricularia auricula* (W)*, Auricularia polytricha* (W)*, Calostoma japonicum* (ECM)*, Coprinus atramentarius* (L)*, Cordyceps annullata (NA), Cyclomyces fuscus* (W)*, Exdia glandulosa* (W)*, Ganoderma applanatum* (W)*, Hydnangium carneum* (ECM)*, Hygrocybe aurantia* (L)*, Hypholoma fasciculare* (W)*, Hypholoma sublateritium* (W)*, Inonotus xeranticus* (W)*, Lentinula edodes* (W)*, Lenzites betulinus* (W)*, Lepista nuda* (L)*, Lyophyllum decastes* (L)*, Panellus stypticus* (W)*, Pleurotus ostreatus* (W)*, Polyporus alveolarius* (W)*, Psathyrella piluliformis* (W)*, Pycnoporus coccineus* (W)*, Russula omiensis* (ECM)*, Scleroderma verrucosum* (ECM)*, Strobilurus stephanocystis (NA), Trametes versicolor* (W) |
| 25-Jan-09 | *Auricularia polytricha* (W)*, Bjerkandera adusta* (W)*, Calostoma japonicum* (ECM)*, Cerrena unicolor* (W)*, Ciborinia camelliae* (L)*, Cordyceps annullata (NA), Cordyceps heteropoda (NA), Cordyceps ophioglossoides (NA), Cyclomyces fuscus* (W)*, Daedalea dickinsii* (W)*, Exdia glandulosa* (W)*, Exdia uvapassa* (W)*, Flammulina velutipes* (W)*, Fomitopsis sensitiva* (W)*, Hydnangium carneum* (ECM)*, Hydnochaete tabacinoides* (W)*, Hygrocybe aurantia* (L)*, Lentinellus ursinus* (W)*, Lentinula edodes* (W)*, Lenzites betulinus* (W)*, Lepista nuda* (L)*, Mycena galopus* (W)*, Mycena laevigata* (W)*, Oligoporus caesius* (W)*, Panellus stypticus* (W)*, Phanerochaete crossa* (W)*, Phellinus gilvus* (W)*, Phellinus substygius* (W)*, Pleurotus ostreatus* (W)*, Polyporus alveolarius* (W)*, Psathyrella piluliformis* (W)*, Pycnoporus coccineus* (W)*, Russula omiensis* (ECM)*, Scleroderma verrucosum* (ECM)*, Stereum gausapatum* (W)*, Trametes villosa* (W) |
| 22-Feb-09 | *Aleurodiscus amorphus* (W)*, Antrodiella gypsea* (W)*, Antrodiella zonata* (W)*, Artomyces colensoi* (W)*, Auricularia auricula* (W)*, Auricularia polytricha* (W)*, Caloscypha fulgens* (L)*, Chlorencoelia versiformis* (W)*, Ciborinia camelliae* (L)*, Cordyceps annullata (NA), Cordyceps heteropoda (NA), Cordyceps ophioglossoides (NA), Cyclomyces fuscus* (W)*, Elaphocordyceps capitata (NA), Exdia glandulosa* (W)*, Exdia uvapassa* (W)*, Flammulina velutipes* (W)*, Hydnangium carneum* (ECM)*, Hypholoma fasciculare* (W)*, Irpex lacteus* (W)*, Lactarius camphoratus* (ECM)*, Lentinula edodes* (W)*, Lycoperdon perlatum* (L)*, Microporus affinis* (W)*, Mycena galopus* (W)*, Panellus stypticus* (W)*, Phanerochaete crossa* (W)*, Phellinus gilvus* (W)*, Russula omiensis* (ECM)*, Scleroderma verrucosum* (ECM)*, Trametes hirsuta* (W)*, Tremella mesenterica* (W)*, Tricholoma ustale* (ECM)*, Tubaria furfuracea* (L) |
| 22-Mar-09 | *Caloscypha fulgens* (L)*, Calostoma japonicum* (ECM)*, Ciborinia camelliae* (L)*, Coltricia pusilla* (ECM)*, Cordyceps heteropoda (NA), Cordyceps ophioglossoides (NA), Cryptoporus volvatus* (W)*, Cyclomyces fuscus* (W)*, Daedaleopsis styracina* (W)*, Entoloma staurosporum* (L)*, Exdia glandulosa* (W)*, Exdia uvapassa* (W)*, Ganoderma applanatum* (W)*, Gloiocephala cryptomeriae* (W)*, Hydnangium carneum* (ECM)*, Hypholoma fasciculare* (W)*, Irpex lacteus* (W)*, Lactarius camphoratus* (ECM)*, Lentinellus ursinus* (W)*, Lentinula edodes* (W)*, Lenzites betulinus* (W)*, Morchella conica* (L)*, Mycena alcalina* (W)*, Mycena galopus* (W)*, Phellinus gilvus* (W)*, Pleurotus ostreatus* (W)*, Porodisculus pendulus* (W)*, Postia japonica* (W)*, Psathyrella piluliformis* (W)*, Pseudoplectania nigrella* (L)*, Pycnoporus coccineus* (W)*, Russula omiensis* (ECM)*, Trametes versicolor* (W)*, Trichoglossum hirsutum* (L)*, Tricholoma ustale* (ECM)*, Tubaria furfuracea* (L) |
| 25-Apr-09 | *Auricularia auricula* (W)*, Auricularia polytricha* (W)*, Calostoma japonicum* (ECM)*, Cordyceps heteropoda (NA), Cryptoporus volvatus* (W)*, Entoloma staurosporum* (L)*, Exdia glandulosa* (W)*, Exdia uvapassa* (W)*, Hygrocybe aurantia* (L)*, Hypholoma fasciculare* (W)*, Lactarius camphoratus* (ECM)*, Lentinula edodes* (W)*, Microporus affinis* (W)*, Panellus stypticus* (W)*, Pluteus atricapillus* (W)*, Pycnoporus coccineus* (W)*, Russula omiensis* (ECM)*, Tremella fimbriata* (W)*, Trichaptum biforme* (W)*, Xerula pudens* (W) |
| 31-May-09 | *Agrocybe cylindracea* (W)*, Aleurina imaii* (L)*, Amanita citrina var. grisea* (ECM)*, Amanita ibotengutake* (ECM)*, Auricularia auricula* (W)*, Auricularia polytricha* (W)*, Boletellus shichianus* (ECM)*, Boletus subvelutipes* (ECM)*, Boletus umbriniporus* (ECM)*, Chalciporus piperatus* (ECM)*, Collybia peronata* (L)*, Cordyceps annullata (NA), Cordyceps intermedia (NA), Cordyceps japonensis (NA), Cortinarius alboviolaceus* (ECM)*, Cortinarius galeroides* (ECM)*, Cortinarius violaceus* (ECM)*, Craterellus cornucopioides* (ECM)*, Cryptoporus volvatus* (W)*, Dicephalospora rufocornea* (W)*, Exdia uvapassa* (W)*, Fistulina hepatica* (W)*, Galiella celebica* (W)*, Hydnotrya tulasnei* (ECM)*, Hypholoma fasciculare* (W)*, Lactarius akahatsu* (ECM)*, Lactarius camphoratus* (ECM)*, Lactarius gracilis* (ECM)*, Lentinellus ursinus* (W)*, Lyophyllum decastes* (L)*, Marasmius purpureostriatus* (L)*, Microporus affinis* (W)*, Pluteus atricapillus* (W)*, Polyporus alveolarius* (W)*, Polyporus arcularius* (W)*, Polyporus badius* (W)*, Porodisculus pendulus* (W)*, Postia japonica* (W)*, Russula alboareolata* (ECM)*, Russula rosacea* (ECM)*, Schizophyllum commune* (W)*, Suillus granulatus* (ECM)*, Trametes hirsuta* (W)*, Xerula pudens* (W) |
| 21-Jun-09 | *Agrocybe cylindracea* (W)*, Amanita citrina var. grisea* (ECM)*, Astraeus hygrometricus* (ECM)*, Auricularia auricula* (W)*, Boletellus shichianus* (ECM)*, Calocera cornea* (W)*, Coltricia cinnamomea* (ECM)*, Cordyceps annullata (NA), Cordyceps ormicarum (NA), Cortinarius subalboviolaceus* (ECM)*, Crepidotus sulphurinus* (W)*, Crinipellis stipitaria* (L)*, Cyptotrama asprata* (W)*, Daedalea dickinsii* (W)*, Dicephalospora rufocornea* (W)*, Exdia uvapassa* (W)*, Gerronema fibula* (L)*, Hydnangium carneum* (ECM)*, Hypholoma fasciculare* (W)*, Inocybe maculata* (ECM)*, Lactarius akahatsu* (ECM)*, Lactarius gracilis* (ECM)*, Marasmiellus nigripes* (W)*, Marasmius maximus* (L)*, Mycena osmundicola* (W)*, Neolentinus lepideus* (W)*, Pleurotus pulmonarius* (W)*, Polyporus badius* (W)*, Porodisculus pendulus* (W)*, Pycnoporus coccineus* (W)*, Russula alboareolata* (ECM)*, Russula cyanoxantha* (ECM)*, Russula mariae* (ECM)*, Russula pectinatoides* (ECM)*, Russula rosacea* (ECM)*, Russula rubescens* (ECM)*, Tylopilus castaneiceps* (ECM)*, Xeromphalina campanella* (W)*, Xeromphalina cauticinalis* (W) |
| 16-Aug-09 | *Amanita alboflavescens* (ECM)*, Amanita citrina var. grisea* (ECM)*, Amanita farinosa* (ECM)*, Amanita fuliginea* (ECM)*, Amanita hongoi* (ECM)*, Amanita pseudoporphyria* (ECM)*, Amanita rufoferruginea* (ECM)*, Amanita similis* (ECM)*, Amanita spissacea* (ECM)*, Amanita sychnopyramis f. subannulata* (ECM)*, Amanita timida* (ECM)*, Amanita vaginata var. vaginata* (ECM)*, Amanita virgineoides* (ECM)*, Amanita volvata* (ECM)*, Armillaria tabescens* (W)*, Aureoboletus thibetanus* (ECM)*, Auricularia polytricha* (W)*, Boletellus emodensis* (ECM)*, Boletellus russellii* (ECM)*, Boletus aokii* (ECM)*, Boletus fraternus* (ECM)*, Boletus ornatipes* (ECM)*, Boletus pseudocalopus* (ECM)*, Boletus subcinnamomeus* (ECM)*, Boletus umbriniporus* (ECM)*, Calostoma japonicum* (ECM)*, Calvatia craniiformis* (L)*, Camarops petersii* (W)*, Cantharellus cinereus* (ECM)*, Clavulina cristata* (ECM)*, Coltricia pusilla* (ECM)*, Cortinarius galeroides* (ECM)*, Cortinarius rubicundulus* (ECM)*, Cryptoporus volvatus* (W)*, Cyclomyces fuscus* (W)*, Entoloma omiensis* (L)*, Ganoderma applanatum* (W)*, Hygrocybe turunda* (L)*, Inocybe asterospora* (ECM)*, Inocybe lutea* (ECM)*, Inonotus vallatus* (W)*, Laccaria vinaceoavellanea* (ECM)*, Lactarius castanopsidis* (ECM)*, Lactarius gerardii* (ECM)*, Laetiporus versisporus* (W)*, Marasmius pulcherripes* (L)*, Marasmius siccus* (L)*, Microporus affinis* (W)*, Neolentinus lepideus* (W)*, Perenniporia minutissima* (W)*, Perenniporia ochroleuca* (W)*, Phellinus gilvus* (W)*, Piptoporus soloniensis* (W)*, Polyporus alveolarius* (W)*, Ramariopsis helvola* (ECM)*, Russula alboareolata* (ECM)*, Russula castanopsidis* (ECM)*, Russula compacta* (ECM)*, Russula cyanoxantha* (ECM)*, Russula eburneoareolata* (ECM)*, Russula foetens* (ECM)*, Russula kansaiensis* (ECM)*, Russula lilacea* (ECM)*, Russula mariae* (ECM)*, Russula sororia* (ECM)*, Russula vesca* (ECM)*, Trichaptum elongatum* (W)*, Trichaptum fuscoviolaceum* (W)*, Tylopilus argillaceus* (ECM)*, Tylopilus ballouii* (ECM)*, Tylopilus fumosipes* (ECM)*, Tylopilus nigropurpureus* (ECM)*, Tylopilus valens* (ECM)*, Tylopilus vinosobrunneus* (ECM)*, Tyromyces chioneus* (W)*, Xerocomus parvulus* (ECM)*, Xeromphalina campanella* (W)*, Xylobolus spectabilis* (W) |
| 27-Sep-09 | *Agrocybe cylindracea* (W)*, Amanita ibotengutake* (ECM)*, Calostoma japonicum* (ECM)*, Chalciporus piperatus* (ECM)*, Dicephalospora rufocornea* (W)*, Ganoderma applanatum* (W)*, Hypholoma fasciculare* (W)*, Hypoxylon truncatum* (W)*, Isaria takamizusanensis (NA), Microporus affinis* (W)*, Perenniporia ochroleuca* (W)*, Pholiota malicola* (W)*, Pleurotus pulmonarius* (W)*, Polyporus badius* (W)*, Pycnoporus coccineus* (W)*, Russula eburneoareolata* (ECM)*, Russula japonica* (ECM)*, Suillus luteus* (ECM)*, Trichaptum biforme* (W)*, Xylaria polymorpha* (W) |
| 18-Oct-09 | *Agaricus abruptibulbus* (L)*, Agrocybe cylindracea* (W)*, Aleuria aurantia* (L)*, Amanita ceciliae* (ECM)*, Amanita esculenta* (ECM)*, Amanita fulva* (ECM)*, Amanita ibotengutake* (ECM)*, Amanita vaginata var. punctata* (ECM)*, Amanita virosa* (ECM)*, Callistosporium luteoolivaceum* (L)*, Calostoma japonicum* (ECM)*, Cantharellus cinereus* (ECM)*, Chalciporus piperatus* (ECM)*, Chlorophyllum molybdites* (L)*, Clavulina rugosa* (ECM)*, Collybia neofusipes* (L)*, Cyclomyces fuscus* (W)*, Dicephalospora rufocornea* (W)*, Flammulina velutipes* (W)*, Ganoderma applanatum* (W)*, Gerronema fibula* (L)*, Hebeloma vinosophyllum* (ECM)*, Hygrocybe aurantia* (L)*, Hypholoma fasciculare* (W)*, Inocybe maculata* (ECM)*, Inonotus vallatus* (W)*, Laccaria bicolor* (ECM)*, Laccaria vinaceoavellanea* (ECM)*, Lactarius akahatsu* (ECM)*, Lactarius gracilis* (ECM)*, Lactarius lividatus* (ECM)*, Laetiporus sulphureus* (W)*, Laetiporus versisporus* (W)*, Lentinula edodes* (W)*, Lenzites betulinus* (W)*, Lepista nuda* (L)*, Lycoperdon pedicellatum* (L)*, Lycoperdon perlatum* (L)*, Lyophyllum decastes* (L)*, Mycena haematopoda* (W)*, Mycena pura* (W)*, Oligoporus caesius* (W)*, Phallus impudicus* (L)*, Phellinus gilvus* (W)*, Pleurotus pulmonarius* (W)*, Pluteus atricapillus* (W)*, Polyporus alveolarius* (W)*, Psathyrella candolliana* (W)*, Psathyrella piluliformis* (W)*, Psathyrella velutina* (W)*, Psilocybe subcaerulipes* (L)*, Russula mariae* (ECM)*, Suillus granulatus* (ECM)*, Suillus luteus* (ECM)*, Trametes versicolor* (W)*, Xerocomus nigromaculatus* (ECM)*, Xeromphalina campanella* (W)*, Xerula radicata* (W) |
| 15-Nov-09 | *Amanita abrupta* (ECM)*, Amanita subjunquillea var. alba* (ECM)*, Astraeus hygrometricus* (ECM)*, Auricularia auricula* (W)*, Auricularia polytricha* (W)*, Calostoma japonicum* (ECM)*, Calvatia craniiformis* (L)*, Camarophyllus pratensis* (L)*, Camarophyllus virgineus* (L)*, Chalciporus piperatus* (ECM)*, Clitocybe fragrans* (L)*, Collybia neofusipes* (L)*, Coprinus atramentarius* (L)*, Cortinarius purpurascens* (ECM)*, Cyclomyces fuscus* (W)*, Entoloma japonicus* (L)*, Entoloma staurosporum* (L)*, Exdia glandulosa* (W)*, Grifola frondosa* (W)*, Hebeloma vinosophyllum* (ECM)*, Hydnangium carneum* (ECM)*, Hygrocybe aurantia* (L)*, Hygrocybe conica* (L)*, Hypholoma fasciculare* (W)*, Hypholoma sublateritium* (W)*, Laccaria amethystea* (ECM)*, Lactarius chrysorrheus* (ECM)*, Lactarius gracilis* (ECM)*, Lactarius quietus* (ECM)*, Lentinula edodes* (W)*, Lenzites betulinus* (W)*, Lycoperdon perlatum* (L)*, Lycoperdon spadiceum* (L)*, Lyophyllum decastes* (L)*, Melanogaster intermedius* (ECM)*, Mycena haematopoda* (W)*, Panellus stypticus* (W)*, Perenniporia ochroleuca* (W)*, Phellinus gilvus* (W)*, Pleurocybella porrigens* (W)*, Polyporus alveolarius* (W)*, Psathyrella piluliformis* (W)*, Psathyrella velutina* (W)*, Pycnoporus coccineus* (W)*, Russula alboareolata* (ECM)*, Russula compacta* (ECM)*, Russula cyanoxantha* (ECM)*, Russula kansaiensis* (ECM)*, Russula pectinatoides* (ECM)*, Strobilurus ohshimae* (W)*, Suillus luteus* (ECM)*, Tremella foliacea* (W)*, Xeromphalina cauticinalis* (W)*, Xylaria polymorpha* (W) |
| 13-Dec-09 | *Aleurina imaii* (L)*, Aleurodiscus amorphus* (W)*, Antrodiella zonata* (W)*, Astraeus hygrometricus* (ECM)*, Auricularia auricula* (W)*, Auricularia polytricha* (W)*, Calostoma japonicum* (ECM)*, Camarophyllus pratensis* (L)*, Camarophyllus virgineus* (L)*, Coprinus atramentarius* (L)*, Coriolus subradiatus* (W)*, Cortinarius galeroides* (ECM)*, Craterellus cornucopioides* (ECM)*, Daedalea dickinsii* (W)*, Daedaleopsis purpurea* (W)*, Ganoderma applanata (NA), Entoloma japonicus* (L)*, Exdia glandulosa* (W)*, Exdia uvapassa* (W)*, Fistulina hepatica* (W)*, Hydnangium carneum* (ECM)*, Hygrocybe aurantia* (L)*, Hygrocybe conica* (L)*, Hypholoma fasciculare* (W)*, Hypholoma sublateritium* (W)*, Ileodictyon gracile* (L)*, Inonotus xeranticus* (W)*, Isaria farinosa (NA), Lactarius camphoratus* (ECM)*, Lactarius chrysorrheus* (ECM)*, Lactarius gracilis* (ECM)*, Lentinula edodes* (W)*, Lenzites betulinus* (W)*, Lepista nuda* (L)*, Lyophyllum decastes* (L)*, Marasmiellus foetidus* (W)*, Merulius tremellosus* (W)*, Mycena haematopoda* (W)*, Mycena laevigata* (W)*, Oligoporus caesius* (W)*, Panellus stypticus* (W)*, Phanerochaete crossa* (W)*, Phellinus gilvus* (W)*, Pisolithus tinctorius* (ECM)*, Pleurotus ostreatus* (W)*, Pleurotus pulmonarius* (W)*, Polyporus alveolarius* (W)*, Postia japonica* (W)*, Psathyrella piluliformis* (W)*, Pycnoporus coccineus* (W)*, Russula alboareolata* (ECM)*, Russula omiensis* (ECM)*, Schizophyllum commune* (W)*, Scleroderma citrinum* (ECM)*, Stereopsis burtianum* (W)*, Stereum gausapatum* (W)*, Stereum ostrea* (W)*, Strobilurus ohshimae* (W)*, Strobilurus stephanocystis (NA), Suillus luteus* (ECM)*, Tremella fimbriata* (W)*, Tremella mesenterica* (W)*, Tricholoma myomyces* (ECM) |
| 24-Jan-10 | *Aleurodiscus aponicus* (W)*, Antrodiella zonata* (W)*, Auricularia polytricha* (W)*, Calostoma japonicum* (ECM)*, Coltriciella pusilla* (ECM)*, Coriolus subradiatus* (W)*, Cyclomyces fuscus* (W)*, Ganoderma applanata (NA), Exdia glandulosa* (W)*, Gloeoporus dichrous* (W)*, Hydnangium carneum* (ECM)*, Hypholoma fasciculare* (W)*, Inonotus xeranticus* (W)*, Isaria farinosa (NA), Lenzites betulinus* (W)*, Lopharia mirabilis* (W)*, Lycoperdon perlatum* (L)*, Microporus affinis* (W)*, Microporus vernicipes* (W)*, Mycena laevigata* (W)*, Panellus stypticus* (W)*, Perenniporia ochroleuca* (W)*, Psathyrella piluliformis* (W) |
| 28-Feb-10 | *Aleurodiscus mirabilis* (W)*, Antrodiella zonata* (W)*, Astraeus hygrometricus* (ECM)*, Auricularia auricula* (W)*, Auricularia polytricha* (W)*, Caloscypha fulgens* (L)*, Calostoma japonicum* (ECM)*, Cordyceps annullata (NA), Cordyceps canadensis (NA), Cordyceps heteropoda (NA), Cordyceps ophioglossoides (NA), Cyclomyces fuscus* (W)*, Daedalea dickinsii* (W)*, Exdia glandulosa* (W)*, Exdia uvapassa* (W)*, Flammulina velutipes* (W)*, Hydnangium carneum* (ECM)*, Hydnochaete tabacinoides* (W)*, Hypholoma fasciculare* (W)*, Lentinula edodes* (W)*, Lenzites betulinus* (W)*, Lycoperdon perlatum* (L)*, Marasmiellus foetidus* (W)*, Microporus vernicipes* (W)*, Morchella conica* (L)*, Mycena laevigata* (W)*, Panellus stypticus* (W)*, Phellinus gilvus* (W)*, Pleurotus ostreatus* (W)*, Polyporus alveolarius* (W)*, Postia japonica* (W)*, Psathyrella piluliformis* (W)*, Russula omiensis* (ECM)*, Schizophyllum commune* (W)*, Trametes orientalis* (W)*, Trametes versicolor* (W)*, Trichaptum biforme* (W) |
| 28-Mar-10 | *Antrodiella zonata* (W)*, Arachnopeziza aurelia* (L)*, Auricularia auricula* (W)*, Caloscypha fulgens* (L)*, Cordyceps annullata (NA), Cordyceps heteropoda (NA), Cryptoporus volvatus* (W)*, Cyclomyces fuscus* (W)*, Entoloma staurosporum* (L)*, Exdia glandulosa* (W)*, Exdia uvapassa* (W)*, Hydnangium carneum* (ECM)*, Hypholoma fasciculare* (W)*, Isaria farinosa (NA), Lactarius camphoratus* (ECM)*, Lentinula edodes* (W)*, Lycoperdon perlatum* (L)*, Microporus affinis* (W)*, Morchella conica* (L)*, Phellinus gilvus* (W)*, Pluteus atricapillus* (W)*, Postia japonica* (W)*, Psathyrella piluliformis* (W)*, Russula omiensis* (ECM)*, Trametes orientalis* (W)*, Trametes versicolor* (W)*, Tremella mesenterica* (W)*, Trichaptum biforme* (W)*, Trichoglossum hirsutum* (L)*, Tricholoma ustale* (ECM) |
| 25-Apr-10 | *Agaricus aestivalis　var. veneris* (L)*, Auricularia auricula* (W)*, Auricularia polytricha* (W)*, Coprinus atramentarius* (L)*, Cordyceps annullata (NA), Cordyceps heteropoda (NA), Exdia glandulosa* (W)*, Exdia uvapassa* (W)*, Fistulina hepatica* (W)*, Hygrocybe aurantia* (L)*, Hypholoma fasciculare* (W)*, Lactarius camphoratus* (ECM)*, Lentinula edodes* (W)*, Lenzites betulinus* (W)*, Lycoperdon perlatum* (L)*, Morchella conica* (L)*, Mycena pelianthina* (W)*, Pluteus atricapillus* (W)*, Polyporus alveolarius* (W)*, Psathyrella piluliformis* (W)*, Russula fragilis* (ECM)*, Russula omiensis* (ECM)*, Suillus luteus* (ECM) |
| 16-May-10 | *Agrocybe cylindracea* (W)*, Aleurina imaii* (L)*, Amanita fuliginea* (ECM)*, Auricularia auricula* (W)*, Auricularia polytricha* (W)*, Collybia dryophila* (L)*, Cordyceps annullata (NA), Cordyceps formicarum (NA), Cordyceps heteropoda (NA), Cordyceps ophioglossoides (NA), Cortinarius violaceus* (ECM)*, Cryptoporus volvatus* (W)*, Daedaleopsis purpurea* (W)*, Fistulina hepatica* (W)*, Helvella acetabulum* (ECM)*, Hygrocybe aurantia* (L)*, Hypholoma fasciculare* (W)*, Isaria farinosa (NA), Laccaria bicolor* (ECM)*, Laccaria nigra* (ECM)*, Lactarius camphoratus* (ECM)*, Lactarius gracilis* (ECM)*, Mycena galericulata* (W)*, Neolentinus lepideus* (W)*, Pluteus atricapillus* (W)*, Postia japonica* (W)*, Psathyrella piluliformis* (W)*, Psathyrella velutina* (W)*, Pseudoplectania nigrella* (L)*, Pycnoporus coccineus* (W)*, Russula fragilis* (ECM) |
| 20-Jun-10 | *Agaricus subrutilescens* (L)*, Agrocybe erebia* (W)*, Amanita farinosa* (ECM)*, Amanita vaginata var. vaginata* (ECM)*, Auricularia auricula* (W)*, Auricularia polytricha* (W)*, Boletus fraternus* (ECM)*, Chalciporus piperatus* (ECM)*, Collybia confluens* (L)*, Collybia dryophila* (L)*, Collybia neofusipes* (L)*, Coltriciella pusilla* (ECM)*, Coprinus leiocephalus* (L)*, Cortinarius galeroides* (ECM)*, Cortinarius subalboviolaceus* (ECM)*, Cyptotrama asprata* (W)*, Dicephalospora rufocornea* (W)*, Fistulina hepatica* (W)*, Gerronema fibula* (L)*, Gymnopilus aeruginosus* (W)*, Hyphoderma setigerum* (W)*, Isaria japonica (NA), Lactarius camphoratus* (ECM)*, Lactarius gracilis* (ECM)*, Lepiota pseudogranulosa* (L)*, Marasmius pulcherripes* (L)*, Microporus affinis* (W)*, Microporus vernicipes* (W)*, Mycena rorida* (W)*, Pholiota malicola var. macropoda* (W)*, Phylloporus bellus var. cyanescens* (ECM)*, Pleurotus pulmonarius* (W)*, Pluteus atricapillus* (W)*, Polyporus alveolarius* (W)*, Polyporus arcularius* (W)*, Polyporus badius* (W)*, Pseudocolus schellenbergiae* (L)*, Pycnoporus coccineus* (W)*, Russula alboareolata* (ECM)*, Scleroderma verrucosum* (ECM)*, Trametes orientalis* (W)*, Trichaptum abietinum* (W)*, Xerula pudens* (W)*, Xerula radicata* (W) |
| 25-Jul-10 | *Agrocybe cylindracea* (W)*, Amanita citrina var. grisea* (ECM)*, Amanita farinosa* (ECM)*, Amanita fuliginea* (ECM)*, Amanita griseofarinosa* (ECM)*, Amanita pseudoporphyria* (ECM)*, Amanita rubescens* (ECM)*, Amanita ｒufoferruginea* (ECM)*, Amanita spissacea* (ECM)*, Amanita sychnopyramis f. subannulata* (ECM)*, Amanita vaginata var. vaginata* (ECM)*, Aureoboletus thibetanus* (ECM)*, Austroboletus gracilis* (ECM)*, Austroboletus subvirens* (ECM)*, Boletus fraternus* (ECM)*, Boletus granulopunctatus* (ECM)*, Boletus griseus var. fuscus* (ECM)*, Boletus pseudocalopus* (ECM)*, Boletus subcinnamomeus* (ECM)*, Cryptoporus volvatus* (W)*, Ganoderma applanata (NA), Gyroporus longicystidiatus* (ECM)*, Hygrocybe cuspidata* (L)*, Inocybe lacera* (ECM)*, Inocybe lutea* (ECM)*, Inocybe maculata* (ECM)*, Inonotus vallatus* (W)*, Laccaria vinaceoavellanea* (ECM)*, Lactarius gerardii* (ECM)*, Lactarius subvellereus* (ECM)*, Paxillus atrotomentosus* (W)*, Polyporus badius* (W)*, Psathyrella candolliana* (W)*, Pulveroboletus ravenelii* (ECM)*, Pycnoporus coccineus* (W)*, Russula castanopsidis* (ECM)*, Russula cyanoxantha* (ECM)*, Russula eburneoareolata* (ECM)*, Russula foetens* (ECM)*, Russula lilacea* (ECM)*, Russula mariae* (ECM)*, Russula senecis* (ECM)*, Russula vesca* (ECM)*, Russula violeipes* (ECM)*, Tylopilus argillaceus* (ECM)*, Tylopilus ballouii* (ECM)*, Tylopilus ferrugineus* (ECM)*, Tylopilus fumosipes* (ECM)*, Tylopilus nigerrimus* (ECM)*, Tylopilus nigropurpureus* (ECM)*, Tylopilus valens* (ECM)*, Xerocomus nigromaculatus* (ECM)*, Xerula pudens* (W)*, Xerula radicata* (W) |
| 29-Aug-10 | *Amanita fuliginea* (ECM)*, Amanita hongoi* (ECM)*, Amanita oberwinklerana* (ECM)*, Amanita spissacea* (ECM)*, Amanita sychnopyramis f. subannulata* (ECM)*, Amanita virgineoides* (ECM)*, Armillariella tabescens* (W)*, Aureoboletus thibetanus* (ECM)*, Austroboletus fusisporus* (ECM)*, Boletellus emodensis* (ECM)*, Boletus obscureumbrinus* (ECM)*, Boletus ornatipes* (ECM)*, Boletus quercinus* (ECM)*, Boletus subcinnamomeus* (ECM)*, Boletus umbriniporus* (ECM)*, Cerrena unicolor* (W)*, Cryptoporus volvatus* (W)*, Cyclomyces fuscus* (W)*, Dicephalospora rufocornea* (W)*, Heimiella japonica* (ECM)*, Hygrocybe cuspidata* (L)*, Inocybe lutea* (ECM)*, Inonotus vallatus* (W)*, Leccinum intusrubens* (ECM)*, Marasmius maximus* (L)*, Microporus affinis* (W)*, Neolentinus lepideus* (W)*, Paxillus atrotomentosus* (W)*, Phellinus gilvus* (W)*, Pholiota malicola var. macropoda* (W)*, Phylloporus bellus* (ECM)*, Pluteus leoninus* (W)*, Polyporus badius* (W)*, Pulveroboletus auriflammeus* (ECM)*, Pycnoporus coccineus* (W)*, Russula amoena* (ECM)*, Russula subnigricans* (ECM)*, Russula vesca* (ECM)*, Russula viridirubrolimbata* (ECM)*, Strobilomyces verruculosus* (ECM)*, Trametes orientalis* (W)*, Trichaptum elongatum* (W)*, Tylopilus ballouii* (ECM)*, Tylopilus ferrugineus* (ECM)*, Tylopilus nigerrimus* (ECM) |
| 19-Sep-10 | *Agrocybe cylindracea* (W)*, Amanita spissacea* (ECM)*, Amanita vaginata var. vaginata* (ECM)*, Auricularia polytricha* (W)*, Austroboletus fusisporus* (ECM)*, Cryptoporus volvatus* (W)*, Cyclomyces fuscus* (W)*, Dicephalospora rufocornea* (W)*, Entoloma japonicus* (L)*, Gerronema nemorale* (L)*, Hypholoma fasciculare* (W)*, Inocybe cookei* (ECM)*, Inonotus vallatus* (W)*, Isaria sinclairii (NA), Lepiota hetieri* (L)*, Leucocoprinus fragilissimus* (L)*, Megacollybia platyphylla* (W)*, Pleurotus ostreatus* (W)*, Polyporus badius* (W)*, Postia japonica* (W)*, Russula alboareolata* (ECM)*, Russula eburneoareolata* (ECM)*, Russula japonica* (ECM)*, Russula subnigricans* (ECM)*, Russula violeipes* (ECM)*, Xerocomus nigromaculatus* (ECM)*, Xeromphalina campanella* (W)*, Xerula pudens* (W)*, Xylaria polymorpha* (W) |
| 31-Oct-10 | *Agaricus subrutilescens* (L)*, Agrocybe erebia* (W)*, Amanita citrina var. citrina* (ECM)*, Amanita subjunquillea var. alba* (ECM)*, Amanita sychnopyramis f. subannulata* (ECM)*, Amanita vaginata var. vaginata* (ECM)*, Amanita virgineoides* (ECM)*, Amanita volvata* (ECM)*, Aureoboletus thibetanus* (ECM)*, Auricularia polytricha* (W)*, Calostoma japonicum* (ECM)*, Calvatia rubroflava* (L)*, Collybia dryophila* (L)*, Collybia neofusipes* (L)*, Coprinus atramentarius* (L)*, Coprinus micaceus* (W)*, Cortinarius alboviolaceus* (ECM)*, Cortinarius aureobrunneus* (ECM)*, Cortinarius salor* (ECM)*, Cyptotrama asprata* (W)*, Dicephalospora rufocornea* (W)*, Fistulina hepatica* (W)*, Ganoderma lucidum* (W)*, Geastrum fimbriatum* (L)*, Hydnangium carneum* (ECM)*, Hygrocybe aurantia* (L)*, Hypholoma fasciculare* (W)*, Inonotus xeranticus* (W)*, Kobayasia nipponica* (L)*, Laccaria bicolor* (ECM)*, Lactarius camphoratus* (ECM)*, Lactarius gracilis* (ECM)*, Lactarius hatsudake* (ECM)*, Lactarius violascens* (ECM)*, Leotia lubrica* (L)*, Lepista nuda* (L)*, Linderia bicolumnata* (L)*, Lycoperdon perlatum* (L)*, Marasmius siccus* (L)*, Microporus affinis* (W)*, Mycena haematopoda* (W)*, Oligoporus caesius* (W)*, Phallus impudicus* (L)*, Pholiota terrestris* (W)*, Pisolithus tinctorius* (ECM)*, Pleurotus ostreatus* (W)*, Postia japonica* (W)*, Psathyrella piluliformis* (W)*, Psathyrella velutina* (W)*, Pycnoporus coccineus* (W)*, Russula alboareolata* (ECM)*, Russula compacta* (ECM)*, Russula cyanoxantha* (ECM)*, Russula omiensis* (ECM)*, Russula violeipes* (ECM)*, Tremella foliacea* (W)*, Trichaptum abietinum* (W)*, Tylopilus castaneiceps* (ECM)*, Xerocomus parvulus* (ECM)*, Xerula radicata* (W) |
| 28-Nov-10 | *Agrocybe erebia* (W)*, Aleurina imaii* (L)*, Auricularia polytricha* (W)*, Baeospora myosura (NA), Calvatia rubroflava* (L)*, Camarophyllus pratensis* (L)*, Clitocybe fragrans* (L)*, Coprinus atramentarius* (L)*, Cyclomyces fuscus* (W)*, Dacrymyces aurantius* (W)*, Daedaleopsis styracina* (W)*, Daedaleopsis tricolor* (W)*, Dermocybe cinnamomea* (ECM)*, Ganoderma applanata (NA), Hydnangium carneum* (ECM)*, Hygrocybe aurantia* (L)*, Hypholoma fasciculare* (W)*, Hypholoma sublateritium* (W)*, Inonotus vallatus* (W)*, Inonotus xeranticus* (W)*, Isaria farinosa (NA), Laccaria vinaceoavellanea* (ECM)*, Lentinula edodes* (W)*, Lenzites betulinus* (W)*, Lepista nuda* (L)*, Lycoperdon bispinosum* (L)*, Lyophyllum decastes* (L)*, Mycena haematopoda* (W)*, Neolecta vittelina* (L)*, Phellinus gilvoides* (W)*, Pholiota terrestris* (W)*, Pisolithus tinctorius* (ECM)*, Pleurotus ostreatus* (W)*, Postia japonica* (W)*, Psathyrella piluliformis* (W)*, Pulcherricium caeruleum* (W)*, Pycnoporus coccineus* (W)*, Russula compacta* (ECM)*, Russula omiensis* (ECM)*, Scleroderma verrucosum* (ECM)*, Sparassis crispa* (W)*, Strobilurus stephanocystis (NA), Trametes versicolor* (W) |
| 12-Dec-10 | *Auricularia polytricha* (W)*, Camarophyllus pratensis* (L)*, Coriolus subradiatus* (W)*, Cryptoporus volvatus* (W)*, Cyclomyces fuscus* (W)*, Daedaleopsis tricolor* (W)*, Hydnangium carneum* (ECM)*, Hygrocybe aurantia* (L)*, Hygrocybe conica* (L)*, Hypholoma fasciculare* (W)*, Hypholoma sublateritium* (W)*, Hypoxylon truncatum* (W)*, Isaria farinosa (NA), Laetiporus versisporus* (W)*, Lentinula edodes* (W)*, Lenzites betulinus* (W)*, Microporus affinis* (W)*, Neolecta vittelina* (L)*, Panellus stypticus* (W)*, Phanerochaete crossa* (W)*, Phellinus gilvus* (W)*, Pisolithus tinctorius* (ECM)*, Pleurotus ostreatus* (W)*, Porodisculus pendulus* (W)*, Postia japonica* (W)*, Pycnoporus coccineus* (W)*, Resupinatus trichotis* (W)*, Schizophyllum commune* (W)*, Strobilurus stephanocystis (NA), Trametes versicolor* (W)*, Tremella foliacea* (W)*, Trichaptum elongatum* (W) |
| 30-Jan-11 | *Auricularia auricula* (W)*, Coltriciella pusilla* (ECM)*, Cordyceps canadensis (NA), Cryptoporus volvatus* (W)*, Cyclomyces fuscus* (W)*, Daedalea dickinsii* (W)*, Daedaleopsis tricolor* (W)*, Ganoderma applanata (NA), Exdia glandulosa* (W)*, Lenzites betulinus* (W)*, Perenniporia minutissima* (W)*, Phellinus gilvoides* (W)*, Phellinus gilvus* (W)*, Pleurotus ostreatus* (W)*, Polyporus alveolarius* (W)*, Postia japonica* (W)*, Psathyrella piluliformis* (W)*, Pycnoporus coccineus* (W)*, Resupinatus trichotis* (W)*, Scleroderma reae* (ECM)*, Trametes versicolor* (W)*, Trichaptum biforme* (W) |
| 27-Feb-11 | *Bjerkandera adusta* (W)*, Bjerkandera fumosa* (W)*, Calvatia craniiformis* (L)*, Ciborinia camelliae* (L)*, Coltricia dependens* (ECM)*, Coltriciella pusilla* (ECM)*, Cordyceps canadensis (NA), Cordyceps heteropoda (NA), Coriolus subradiatus* (W)*, Cryptoporus volvatus* (W)*, Daedalea dickinsii* (W)*, Daedaleopsis styracina* (W)*, Ganoderma applanata (NA), Exdia glandulosa* (W)*, Heterochaete delicata* (W)*, Hydnangium carneum* (ECM)*, Hypholoma fasciculare* (W)*, Inonotus xeranticus* (W)*, Lentinula edodes* (W)*, Lenzites betulinus* (W)*, Melanoporia purpurascens* (W)*, Microporus affinis* (W)*, Panellus stypticus* (W)*, Phanerochaete crossa* (W)*, Phellinus gilvus* (W)*, Pleurotus ostreatus* (W)*, Polyporus alveolarius* (W)*, Psathyrella piluliformis* (W)*, Pulcherricium caeruleum* (W)*, Resupinatus trichotis* (W)*, Russula omiensis* (ECM)*, Stereum hirsutum* (W)*, Trametes versicolor* (W)*, Trichaptum abietinum* (W)*, Trichaptum biforme* (W)*, Xeromphalina curtipes* (W)*, Xylaria polymorpha* (W) |
| 27-Mar-11 | *Antrodiella zonata* (W)*, Bjerkandera adusta* (W)*, Ciborinia camelliae* (L)*, Cordyceps canadensis (NA), Cordyceps heteropoda (NA), Cordyceps ophioglossoides (NA), Cordyceps sobolifera (NA), Cryptoporus volvatus* (W)*, Cyclomyces fuscus* (W)*, Entoloma staurosporum* (L)*, Exdia glandulosa* (W)*, Geastrum fornicatum* (L)*, Hypholoma fasciculare* (W)*, Ionomidotis frondosa* (W)*, Lentinula edodes* (W)*, Lenzites betulinus* (W)*, Morchella conica* (L)*, Phellinus gilvus* (W)*, Pleurotus ostreatus* (W)*, Postia japonica* (W)*, Psathyrella piluliformis* (W)*, Russula omiensis* (ECM)*, Trametes versicolor* (W)*, Trichaptum biforme* (W)*, Tricholoma ustale* (ECM)*, Tubaria furfuracea* (L) |
