# Supplementary material for: A Thirty-Year Survey Reveals That Ecosystem Function of Fungi Predicts Phenology of Mushroom Fruiting
Source: PLoS One. 2012 Nov 27;7(11):e49777. doi: 10.1371/journal.pone.0049777 (PMC3507881; doi:10.1371/journal.pone.0049777)
Supplement: Text S1 — BUGS code used in this study. (DOC) [file pone.0049777.s006.doc]

**Text S1** BUGS code used in this study.

#Model description

model

{

for (j in 1:24)

{

a[j]~ dnorm(0,1.0E-6) #Random intercepts

for (i in 1:4)

{

b[i,j]~dnorm(0,1.0E-6) #Partial regression coefficient for each explanatory variable

bs[i,j]<- b[i,j]*SD[i]/sd_p[j] #Standard partial regression coefficients for explanatory variables

}

}

for (i in 1:24)

{sd_p[i]<- sd(lp[,i])}

mTm<- mean(Tm[]) #Mean of monthly average temperature

mMR<- mean(MR[]) #Mean of monthly accumulated rainfall

mWR<- mean(WR[]) #Mean of weekly accumulated rainfall

mY<-mean(Y[]) #Mean of number of years elapsed

SD[1]<-sd(Tm[]) #Standard deviation of monthly average temperature

SD[2]<-sd(MR[]) #Standard deviation of monthly accumulated rainfall

SD[3]<-sd(WR[]) #Standard deviation of weekly accumulated rainfall

SD[4]<-sd(Y[]) #Standard deviation of number of years elapsed

for (i in 1:274)

{

d[i]~dunif(0,1) #Detection rate of each survey

for (j in 1:24)

{

logit(p[i,j])<- lp[i,j]

lp[i,j]<- a[j]+b[1,j]*(Tm[i]-mTm)+b[2,j]*(MR[i]-mMR)+b[3,j]*(WR[i]-mWR)+b[4,j]*(Y[i]-mY) #Prior to the occurring probability of species

present[i,j]~dbin(p[i,j],N[j]) #Actual presence of species

detected[i,j]~ dbin(d[i],present[i,j]) #Number of detection

}

}

}

#Dataset

list(detected=structure(.Data=c(0,0,0,0,0,0,0,0,0,0,0,0,0,0,0,0,5,0,0,0,0,0,0,0,10,0,0,0,0,0,1,3,2,0,0,0,0,0,0,0,9,0,0,1,2,0,0,0,9,1,0,0,0,0,0,2,1,0,0,0,0,0,0,1,9,0,0,0,1,0,0,0,54,13,2,3,1,1,3,10,11,0,0,0,4,1,0,0,7,0,0,0,0,0,0,1,23,4,3,0,1,1,0,2,1,0,0,0,1,0,0,0,3,0,0,0,0,0,0,0,19,5,0,0,0,0,3,6,5,0,0,1,1,0,0,1,10,0,0,1,1,0,0,0,2,0,0,0,0,0,0,0,0,0,0,0,0,0,0,0,3,0,0,0,1,0,0,0,5,0,0,0,0,0,0,0,2,0,0,1,0,1,0,0,5,0,0,0,0,0,0,1,3,0,0,0,0,0,0,1,1,0,0,1,0,0,0,0,5,0,1,0,0,0,0,0,1,0,0,0,0,0,0,0,0,0,0,0,0,0,0,0,4,0,0,0,0,0,0,0,0,0,0,0,0,0,0,0,1,0,0,0,1,0,0,0,2,0,0,0,0,0,0,0,4,0,0,0,0,0,1,2,1,0,0,0,1,0,0,0,6,0,1,0,0,0,0,0,4,0,0,0,1,0,2,0,1,0,0,0,1,0,0,0,3,0,1,0,0,0,0,0,0,0,0,0,0,0,0,0,0,0,0,0,0,0,0,0,1,0,0,0,0,0,0,0,41,10,3,4,2,0,1,8,3,0,0,0,1,0,0,0,2,0,0,0,1,0,0,0,22,7,0,1,0,1,3,5,4,1,0,0,0,0,0,0,7,1,0,0,0,0,0,0,28,11,0,1,1,1,0,5,10,0,0,1,0,0,1,2,5,0,0,0,0,0,1,0,15,2,0,0,1,0,1,1,7,0,0,0,0,0,0,0,2,0,0,0,0,0,0,0,4,1,0,0,0,0,1,0,3,0,0,0,0,0,0,0,4,0,0,0,0,0,0,0,1,0,0,0,0,0,0,0,0,0,0,0,0,0,0,0,5,0,0,0,0,0,0,0,1,0,0,0,0,0,0,0,0,0,0,0,0,0,0,0,3,0,0,0,0,0,0,0,2,0,0,0,0,0,0,0,0,0,0,0,0,0,0,0,2,0,1,0,0,0,0,0,1,0,0,0,0,0,0,0,1,0,0,0,0,0,0,0,2,0,0,0,0,0,0,0,1,0,0,0,0,0,0,1,0,0,0,0,0,0,0,0,3,0,0,0,0,0,0,0,3,0,0,0,0,0,1,1,1,0,0,0,0,0,0,0,7,0,2,2,0,0,0,0,3,0,0,0,0,0,1,1,1,0,0,0,0,0,0,1,4,0,0,1,0,0,0,0,32,7,3,2,1,2,3,4,4,0,1,0,1,0,1,0,4,0,0,0,0,0,0,0,41,10,5,2,0,0,2,8,1,0,0,0,1,0,0,0,6,0,0,0,1,0,0,0,20,5,2,0,1,0,0,5,4,0,0,1,0,0,1,1,7,0,0,0,0,0,0,0,9,2,0,0,0,0,1,2,0,0,0,0,0,0,0,0,1,0,0,0,0,0,0,0,3,0,0,0,0,0,0,0,2,0,0,0,1,0,0,0,7,0,0,0,0,0,0,0,1,0,0,0,0,0,0,0,0,0,0,0,0,0,0,0,4,0,0,0,1,0,0,0,1,0,0,0,0,0,0,0,0,0,0,0,0,0,0,0,8,0,0,0,0,0,0,0,2,0,0,0,0,0,0,1,0,0,0,0,0,0,0,0,9,0,1,0,0,0,1,0,2,0,0,0,0,0,0,1,2,0,0,1,1,0,0,0,8,0,1,0,0,0,0,0,1,0,0,0,0,0,1,0,2,0,0,0,1,0,0,0,11,0,2,0,1,1,1,0,3,0,0,0,0,0,2,0,2,0,0,0,1,0,0,0,7,0,0,0,0,2,0,0,24,4,1,1,2,0,4,3,5,0,0,0,1,1,2,0,8,0,0,0,0,1,1,0,26,6,1,2,0,1,2,3,2,0,0,0,0,1,0,0,5,0,0,0,1,0,0,0,9,1,0,0,0,0,0,2,0,0,0,0,0,0,0,0,6,0,0,0,0,0,0,0,28,5,3,1,1,0,0,3,8,0,1,1,0,0,0,2,13,0,0,0,0,0,0,0,32,9,2,0,2,0,2,4,4,0,0,0,1,0,0,0,5,0,0,0,1,0,0,0,7,0,0,0,1,0,1,1,2,0,0,0,0,0,0,0,16,1,0,0,0,0,1,0,3,0,0,0,0,0,0,1,1,0,0,0,0,0,0,0,10,0,0,0,0,0,0,0,2,0,0,0,0,0,0,1,0,0,0,0,0,0,0,0,6,0,0,0,0,0,0,0,3,0,0,0,0,0,0,1,0,0,0,0,0,0,0,0,8,0,0,0,0,0,0,0,2,0,0,0,0,0,0,1,0,0,0,0,0,0,0,0,9,0,0,0,0,0,0,0,3,0,0,0,0,0,0,1,0,0,0,0,0,0,0,0,8,0,0,0,0,0,0,0,3,0,0,0,0,0,1,2,3,0,0,0,0,1,0,1,5,0,0,1,0,0,0,0,39,4,1,1,3,1,1,10,8,0,0,0,2,2,1,0,15,0,0,0,0,0,0,1,17,2,2,1,0,0,0,4,1,0,0,0,0,0,0,0,8,0,0,0,0,0,0,0,6,0,1,0,0,1,0,1,1,0,0,0,0,0,0,0,14,0,0,0,0,0,0,0,10,2,0,0,1,0,1,2,1,0,1,0,0,0,0,0,10,0,0,0,0,0,0,0,5,1,0,0,0,0,0,3,1,0,0,0,0,0,0,0,3,0,0,0,0,0,0,0,2,0,0,0,0,0,0,0,1,0,0,0,0,0,0,0,5,0,0,0,0,0,0,0,2,0,0,0,0,0,0,1,1,0,0,0,0,0,0,0,4,0,0,0,0,0,0,0,3,0,0,0,0,0,0,1,0,0,0,0,0,0,0,0,3,0,0,0,0,0,0,0,2,0,0,0,0,0,0,1,1,0,0,1,0,0,0,0,6,0,1,0,1,0,0,0,3,0,0,0,0,0,0,2,0,0,0,0,0,0,0,0,8,1,0,0,0,0,0,0,8,2,0,0,1,0,1,2,1,0,0,0,0,0,0,1,7,0,0,0,1,0,0,0,21,4,2,1,2,1,2,4,6,0,1,0,2,0,1,0,12,1,0,1,0,0,0,0,43,9,5,2,2,3,2,7,7,1,3,0,1,0,0,0,10,0,0,0,0,1,0,0,39,8,5,1,0,0,2,9,2,0,0,0,1,0,0,0,11,0,0,1,0,0,0,0,10,1,0,1,0,0,0,4,5,0,0,1,0,0,0,0,18,0,0,0,0,0,0,0,17,2,0,0,2,0,2,3,6,0,0,1,0,0,0,0,18,0,2,2,0,1,0,0,15,1,0,0,4,0,1,1,5,0,0,0,1,0,0,0,12,0,1,0,0,0,0,0,8,0,0,0,1,0,2,2,3,0,0,1,1,0,0,0,13,0,1,0,0,1,0,1,3,0,0,0,0,0,0,2,2,0,0,1,0,0,0,0,11,0,1,0,1,0,0,0,3,0,0,0,1,0,0,2,3,0,0,0,1,0,0,0,6,0,1,0,0,0,0,0,5,1,0,0,1,0,1,1,3,0,0,0,2,0,0,0,6,0,0,1,0,0,0,1,17,4,0,1,4,0,1,4,8,0,1,0,1,1,0,2,9,0,0,0,0,0,0,0,14,1,0,0,2,0,1,5,10,0,0,0,2,0,0,2,14,0,1,0,1,0,0,1,15,1,0,0,2,0,1,2,3,0,0,0,1,0,0,0,17,0,0,1,1,0,0,1,31,5,1,0,2,0,3,7,5,0,0,0,0,0,3,0,11,0,0,0,0,0,0,0,34,8,3,3,0,1,1,9,1,0,0,0,0,0,0,0,11,0,0,0,0,0,0,0,40,11,0,1,1,0,5,5,12,0,0,2,0,0,1,0,22,0,1,0,2,1,1,1,2,0,0,0,0,0,0,1,4,0,0,1,1,0,0,0,11,0,0,1,1,0,1,0,1,0,0,0,0,0,0,1,1,0,0,0,0,0,0,0,0,0,0,0,0,0,0,0,6,1,0,0,2,0,1,1,2,0,0,0,1,0,0,0,10,0,0,0,0,0,0,1,38,8,2,2,2,1,2,8,7,0,0,0,1,1,0,1,18,0,0,0,1,0,1,0,35,7,3,3,1,0,3,9,3,0,0,0,0,2,0,0,16,1,0,1,1,0,2,1,19,6,0,0,0,0,0,4,12,1,2,1,2,0,0,1,32,0,1,1,0,1,0,0,24,5,0,0,2,0,4,7,10,0,0,2,2,1,0,2,23,1,1,0,0,0,1,0,3,0,0,0,0,0,0,1,5,0,0,1,1,0,0,0,16,0,0,0,0,0,0,1,4,0,0,0,0,0,0,2,5,0,0,1,1,0,0,0,24,0,2,0,0,0,0,0,3,0,0,0,0,0,0,1,1,0,0,0,1,0,0,0,13,0,0,0,1,0,1,0,1,0,0,0,0,0,0,1,2,0,0,0,1,0,0,0,10,0,0,0,0,0,1,0,13,2,0,0,3,0,1,5,4,0,0,0,2,0,0,1,18,0,0,1,0,1,1,1,12,0,0,2,3,0,0,3,5,0,1,0,2,1,1,0,11,0,0,0,1,0,0,0,40,10,7,2,1,1,0,4,4,2,1,0,1,0,0,0,8,1,0,0,0,0,0,0,6,0,0,0,1,0,0,3,2,0,0,0,0,0,0,0,24,0,1,0,1,1,0,0,7,0,0,0,0,0,1,1,6,0,0,0,1,0,0,0,17,0,1,0,1,2,0,1,1,0,0,0,0,0,0,1,1,0,0,1,0,0,0,0,30,0,1,0,1,0,1,0,3,0,0,0,0,0,0,1,3,0,0,0,1,0,0,0,14,0,2,0,0,0,0,0,6,1,0,0,1,0,1,2,3,0,0,0,1,0,1,0,14,0,0,0,0,1,1,1,30,4,2,0,2,3,2,5,17,2,1,0,1,1,2,2,11,0,0,1,1,0,0,2,36,10,1,1,2,1,2,6,5,0,1,0,1,0,0,1,15,0,0,0,0,0,1,0,5,0,0,0,0,0,0,3,5,0,0,0,1,0,0,0,9,0,2,1,0,0,0,1,2,0,0,0,0,0,0,1,2,0,0,0,0,0,0,0,16,0,1,0,0,0,1,0,1,0,0,0,0,0,0,1,4,0,0,0,1,0,0,0,7,0,0,0,0,0,0,0,23,1,1,0,2,1,2,6,9,0,0,0,2,2,0,1,24,1,2,1,2,0,1,1,23,5,2,2,0,1,2,6,0,0,0,0,0,0,0,0,12,1,0,0,1,0,0,0,4,0,1,0,0,0,0,1,7,1,3,0,0,0,1,0,12,0,0,1,1,0,0,0,2,0,0,0,0,0,0,1,8,1,2,0,1,0,1,0,21,0,1,0,2,1,0,0,32,6,1,1,5,0,5,6,12,1,1,0,0,0,2,1,34,0,1,1,2,1,1,1,2,1,0,0,0,0,0,0,1,0,0,1,0,0,0,0,17,0,0,0,1,1,1,0,1,0,0,0,0,0,0,1,1,0,0,0,0,0,0,0,7,0,0,0,1,0,0,0,1,0,0,0,0,0,0,1,6,0,0,0,1,0,0,0,15,0,0,0,1,0,1,0,5,1,0,0,0,0,1,2,5,0,0,1,0,0,0,0,7,0,1,1,0,0,0,1,8,1,1,2,0,0,0,0,1,1,0,0,0,0,0,0,22,0,0,2,1,0,0,0,4,1,0,0,0,0,1,1,3,0,0,0,0,0,0,0,27,0,1,1,1,1,1,1,1,0,0,0,0,0,0,1,4,0,0,0,2,0,0,0,14,0,0,0,0,0,1,0,3,0,0,0,1,0,0,1,2,0,0,1,0,0,0,0,14,0,0,0,2,0,1,0,29,3,3,0,3,0,3,8,14,1,1,0,0,0,3,1,26,0,1,0,1,0,1,1,43,12,2,3,0,1,3,7,1,0,0,0,0,0,1,0,13,0,0,0,0,0,2,0,6,3,0,0,0,0,0,1,3,1,0,0,2,0,0,0,16,1,0,0,0,0,0,0,57,13,2,2,3,1,5,13,13,0,1,1,2,1,0,0,21,0,1,0,0,0,1,1,30,3,0,0,4,1,3,6,13,1,0,1,1,1,0,1,20,0,2,0,1,0,0,3,4,0,0,0,0,0,0,1,8,0,0,0,1,0,0,0,24,0,0,1,2,0,2,1,13,4,1,1,0,0,0,4,2,0,0,0,0,0,1,0,17,0,0,0,1,1,0,0,3,0,0,0,0,0,0,1,3,0,0,0,0,0,0,0,21,0,0,0,1,0,1,0,0,0,0,0,0,0,0,0,0,0,0,0,0,0,0,0,0,0,0,0,0,0,0,0,1,0,0,0,0,0,0,1,3,0,0,0,1,0,0,0,11,0,0,1,0,0,0,0,22,2,2,0,1,0,2,9,6,1,0,0,1,1,1,0,21,0,0,1,1,0,1,1,14,5,0,0,1,0,2,1,8,1,0,0,2,0,0,0,14,0,0,1,1,0,0,0,7,0,0,0,0,0,2,1,2,0,0,0,0,1,1,0,23,0,1,1,1,0,0,0,3,0,0,0,0,0,0,1,5,0,0,0,1,0,0,0,20,0,1,0,1,1,1,0,3,0,0,0,0,0,0,1,1,0,0,0,0,0,0,0,13,0,1,0,1,0,0,0,5,0,0,0,0,0,0,1,3,0,0,1,0,0,0,0,16,0,1,0,1,0,1,0,3,0,0,0,0,0,0,1,5,0,0,0,1,0,0,0,19,0,0,0,1,0,3,0,12,1,0,0,1,0,3,4,5,0,0,0,2,0,0,0,19,0,1,1,2,1,1,1,19,2,0,0,2,0,2,8,4,0,0,0,2,0,0,1,24,0,0,2,1,1,1,1,37,6,1,2,4,0,2,8,10,0,1,0,2,2,0,1,13,0,0,0,1,0,1,2,39,8,3,4,0,1,1,5,8,0,0,1,1,3,0,0,20,0,0,0,0,0,0,1,24,7,1,1,0,1,1,6,2,0,0,0,0,0,2,0,20,0,0,1,0,0,0,0,13,3,0,0,0,0,1,4,5,1,0,0,1,0,0,1,12,1,0,0,1,0,0,0,42,10,5,2,2,0,2,7,5,0,0,0,1,1,0,0,8,0,1,0,2,0,0,0,4,0,0,0,1,0,0,1,2,0,0,0,0,0,0,0,23,0,0,0,1,0,0,1,3,0,0,0,0,0,0,1,1,0,0,0,0,0,0,0,16,0,1,0,0,0,1,0,1,0,0,0,0,0,0,1,1,0,0,0,0,0,0,0,19,0,1,0,1,0,1,0,3,0,0,0,0,0,0,1,7,0,0,0,1,0,0,0,10,0,0,0,1,0,0,0,4,0,0,0,0,0,1,2,6,0,0,1,1,1,0,0,23,0,2,1,1,0,1,2,30,9,2,2,1,1,2,7,8,2,0,0,2,1,0,0,14,1,0,0,0,0,0,0,63,14,7,3,1,1,2,13,6,1,1,0,1,0,0,0,21,0,0,0,0,0,0,0,68,6,5,6,2,4,5,14,20,1,1,0,1,1,1,2,19,1,0,0,0,1,1,0,15,3,0,0,1,1,3,2,4,0,0,1,0,0,0,0,23,0,2,0,1,1,0,1,16,1,0,0,1,0,3,4,12,0,0,1,1,2,0,0,36,0,2,1,2,0,1,3,3,0,0,0,0,0,0,0,5,0,0,0,1,1,0,0,22,0,0,0,1,0,1,2,4,0,0,0,0,0,0,1,2,0,0,0,0,0,0,0,21,0,1,0,1,0,0,1,4,0,0,0,0,0,0,1,3,0,0,1,0,0,0,0,12,0,1,0,1,0,1,0,3,0,0,0,0,0,0,0,2,0,0,0,0,0,0,0,10,0,0,0,1,0,0,0,5,0,0,0,0,0,0,1,4,0,0,0,1,0,0,0,27,0,3,1,1,0,1,0,7,1,0,0,1,0,2,1,6,1,0,0,3,0,1,0,30,1,1,1,0,0,0,1,18,2,0,0,2,0,1,5,5,1,0,0,1,1,1,1,17,0,0,1,1,0,1,1,22,8,0,1,0,1,0,8,1,0,0,0,0,1,0,0,27,1,0,0,0,0,0,0,17,3,2,1,0,0,1,3,2,0,0,0,0,1,1,0,13,0,0,0,0,1,0,0,22,3,1,1,1,0,1,4,11,3,2,0,0,0,2,0,19,1,1,1,0,0,1,0,23,3,0,0,2,0,4,2,10,0,0,0,1,1,1,0,19,0,2,0,0,0,1,4,12,1,0,0,0,0,2,3,10,0,0,0,1,0,0,0,19,0,0,0,3,0,0,1,4,0,0,0,0,0,1,0,2,0,0,0,0,0,0,1,15,0,0,0,1,0,0,1,3,0,0,0,0,0,0,1,3,0,0,0,0,0,0,0,27,0,1,0,1,0,1,0,2,0,0,0,0,0,0,1,3,0,0,0,0,0,0,0,14,0,0,0,1,0,0,0,2,0,0,0,0,0,1,0,1,0,0,0,0,0,0,0,7,0,0,1,0,0,0,0,7,1,0,0,1,0,1,1,3,0,0,0,2,0,0,0,23,0,1,1,2,0,0,1,20,2,1,0,1,0,2,6,4,1,0,0,1,0,1,0,11,0,0,0,0,0,0,0,24,7,1,1,0,1,1,6,0,0,0,0,0,0,0,0,23,1,0,1,0,0,0,0,12,2,1,0,0,0,1,6,3,0,0,0,0,0,0,0,26,2,0,2,1,1,0,0,56,11,7,4,1,1,3,6,12,1,1,0,1,3,0,1,28,0,1,0,0,1,0,2,17,1,0,0,2,0,2,3,8,0,0,1,1,0,0,0,25,1,1,1,2,0,0,3,2,0,0,0,0,0,0,1,1,0,0,0,0,0,0,0,12,0,1,0,1,0,1,0,1,0,0,0,0,0,0,1,4,0,0,1,0,0,0,0,4,0,0,0,0,0,0,0,4,1,0,0,0,0,1,1,3,0,0,0,2,0,0,0,11,0,1,0,0,0,0,1,7,0,0,0,3,0,1,0,2,0,0,0,1,0,0,0,15,0,0,0,1,0,1,1,16,2,1,0,0,0,3,4,3,0,0,0,0,0,1,0,13,0,1,1,1,0,1,1,54,16,9,5,0,1,2,5,2,0,0,0,1,0,0,0,10,0,0,0,0,1,0,0,2,0,1,0,0,0,0,1,3,1,0,0,0,0,0,0,16,1,0,0,1,1,0,0,11,2,1,2,0,0,0,1,4,0,0,0,0,0,1,0,18,2,0,2,0,1,0,0,19,5,1,1,0,0,4,4,5,1,1,0,0,0,0,0,13,0,1,0,1,0,0,1,3,0,0,0,0,0,0,0,2,0,0,1,0,0,0,0,18,0,0,0,0,1,0,0,4,0,0,0,0,0,0,1,0,0,0,0,0,0,0,0,17,0,0,0,1,0,0,0,2,0,0,0,0,0,0,1,1,0,0,0,0,0,0,0,6,0,1,0,0,0,0,0,3,0,0,0,0,0,0,1,1,0,0,0,0,0,0,0,8,0,0,0,0,0,0,0,1,0,0,0,0,0,0,1,4,0,0,0,1,0,0,0,9,0,0,0,0,0,1,0,5,0,0,0,0,0,1,2,3,0,0,0,1,0,0,0,13,0,1,1,0,0,1,1,19,3,1,0,3,0,2,3,4,0,0,1,1,0,0,0,13,1,0,2,1,1,0,1,45,6,6,4,2,1,3,8,14,1,3,0,1,2,1,1,11,0,0,0,1,1,0,2,43,7,2,3,1,1,4,6,8,0,0,0,1,2,0,3,8,0,0,0,0,0,0,1,18,0,0,0,0,1,1,10,4,0,0,0,0,0,0,0,20,1,1,0,0,0,0,0,8,2,0,0,1,0,2,0,6,0,0,0,0,0,0,1,14,0,1,0,1,0,0,1,14,1,0,0,0,0,1,4,7,0,0,0,1,1,0,0,20,0,1,0,1,0,0,2,9,0,0,0,0,1,1,1,4,0,0,0,1,0,0,0,14,0,1,0,1,0,0,1,3,0,0,0,0,0,0,0,1,0,0,0,0,0,0,0,10,0,0,0,1,0,1,0,4,0,0,0,0,0,0,1,1,0,0,0,0,0,0,0,16,0,1,0,0,0,1,0,3,0,0,0,0,0,0,1,3,0,0,1,1,0,0,0,20,0,0,0,1,0,0,0,2,0,0,0,0,0,0,1,2,0,0,0,1,0,0,0,12,0,0,0,1,0,0,1,32,8,1,0,0,2,4,9,7,1,2,0,0,0,1,1,17,0,0,1,1,0,0,0,57,15,7,8,1,0,1,6,6,1,1,1,1,1,0,0,7,1,0,0,0,0,0,1,37,8,2,3,1,0,2,7,5,0,1,0,0,0,1,2,26,0,1,0,0,2,1,0,15,2,0,0,3,0,2,6,5,1,0,0,0,0,0,1,10,0,1,0,0,0,0,1,8,0,0,0,0,0,2,2,3,0,0,1,0,0,0,0,16,0,0,0,1,0,1,1,4,0,0,0,0,0,0,1,4,0,0,2,1,0,0,0,14,0,3,0,1,0,0,1,3,0,0,0,0,0,0,1,2,0,0,1,0,0,0,0,16,0,1,0,1,0,0,0,5,0,0,0,1,0,0,1,1,0,0,0,0,0,0,0,17,0,1,0,0,0,0,0,2,0,0,0,0,0,0,1,4,0,0,0,1,0,0,0,12,0,1,0,0,0,1,0,0,0,0,0,0,0,0,0,1,0,0,0,1,0,0,0,3,0,0,0,0,0,0,0,3,0,0,0,1,0,1,1,2,0,0,0,1,0,0,0,12,0,0,0,0,1,0,0,7,0,1,0,1,0,1,2,2,0,0,0,1,0,1,0,18,0,0,0,0,2,0,1,38,5,2,3,1,0,6,10,8,0,0,0,1,0,3,0,11,0,0,1,1,0,1,0,42,10,6,7,0,0,1,7,3,0,0,0,0,0,0,0,17,1,0,0,1,1,0,0,47,9,2,2,1,3,4,11,16,2,2,0,1,1,3,2,19,1,0,1,0,0,1,1,26,4,0,1,2,0,5,3,12,0,0,1,2,0,1,1,28,0,1,0,0,1,1,1,15,0,0,0,1,0,2,5,7,0,0,2,1,1,0,0,26,0,2,0,0,0,1,2,4,0,0,0,0,0,1,1,3,0,0,1,1,0,0,0,25,0,0,0,1,0,0,0,1,0,0,0,0,0,0,0,1,0,0,0,0,0,0,0,14,0,1,0,0,0,1,1,2,0,0,0,0,0,0,1,1,0,0,0,0,0,0,0,16,0,1,0,1,0,0,0,2,0,0,0,0,0,0,1,4,0,0,0,1,0,0,0,11,0,1,0,1,0,1,0,5,0,0,0,0,0,1,1,3,0,0,0,1,0,0,0,9,0,0,0,0,0,1,0,12,2,0,0,1,0,4,2,3,0,0,0,1,0,0,0,15,0,1,1,1,0,1,1,18,1,1,0,2,0,2,9,6,0,0,0,1,0,2,0,13,0,0,0,0,0,0,1,42,11,0,2,0,2,2,12,6,0,0,0,1,1,1,0,13,0,0,0,0,0,0,0,10,2,0,0,0,0,0,4,2,1,0,0,0,0,1,0,11,0,0,2,0,0,0,0,48,18,3,2,0,0,2,10,7,0,0,1,0,0,0,1,24,0,0,1,2,1,0,0,19,5,0,0,0,0,3,4,7,0,0,2,1,0,0,0,17,0,1,0,0,0,0,0,3,0,0,0,0,0,1,0,4,0,0,1,0,0,0,0,11,0,0,0,0,0,0,0,4,0,0,0,0,0,0,1,5,0,0,0,1,0,0,0,19,0,1,0,0,0,1,1,2,0,0,0,0,0,0,1,1,0,0,0,0,0,0,0,17,0,0,0,1,0,0,0,3,0,0,0,0,0,0,1,1,0,0,0,0,0,0,0,12,0,1,0,1,0,0,0,3,0,0,0,0,0,0,1,2,0,0,0,0,0,0,0,10,1,0,0,0,0,0,0,2,0,0,0,0,0,1,0,4,0,0,0,1,0,0,0,7,0,0,1,0,0,0,1,5,2,0,0,0,0,1,1,2,0,0,0,0,0,0,0,13,0,0,0,1,0,0,0,27,3,1,1,3,1,3,6,4,1,0,0,1,0,0,0,21,0,1,1,0,0,0,1,68,17,4,8,2,2,5,6,16,0,1,0,2,3,1,0,10,0,0,0,1,0,0,3,9,3,0,0,0,0,0,1,0,0,0,0,0,0,0,0,7,0,0,0,1,0,0,0,75,19,9,7,1,2,1,12,8,0,0,0,2,1,0,0,17,1,0,0,0,0,0,1,18,3,1,1,0,2,3,3,10,1,0,1,2,0,0,1,26,0,1,0,2,1,1,1,10,0,0,0,1,0,1,3,7,0,0,1,2,0,0,0,17,0,1,0,2,0,1,1,1,0,0,0,0,0,0,0,5,0,0,1,0,0,0,0,5,0,1,0,0,0,0,1,3,0,0,0,0,0,0,1,0,0,0,0,0,0,0,0,18,0,0,0,0,0,1,0,1,0,0,0,0,0,0,0,0,0,0,0,0,0,0,0,5,0,0,0,1,0,1,0,3,0,0,0,0,0,0,1,4,0,0,0,1,0,1,0,14,0,0,0,1,0,0,0,3,0,0,0,0,0,1,1,3,0,0,0,1,0,0,0,16,0,2,1,0,0,0,1,2,0,0,0,0,0,1,1,1,0,0,0,1,0,0,0,8,0,0,0,0,0,0,1,28,9,4,4,0,0,0,3,2,0,0,0,0,0,0,0,15,0,0,0,0,0,0,0,29,7,1,2,0,0,1,8,9,1,1,0,0,0,0,1,24,1,0,1,2,1,1,0,35,5,2,1,3,0,3,8,10,0,0,0,1,0,0,2,34,0,1,0,2,0,1,0,35,7,2,1,0,1,4,6,9,0,0,1,0,1,0,1,14,0,0,0,0,1,0,2,6,0,0,0,0,0,1,3,7,0,0,0,0,0,0,1,20,0,2,0,1,0,0,2,4,0,0,0,0,0,0,1,4,0,0,0,0,0,0,0,16,0,0,0,1,0,0,1,4,0,0,0,0,0,0,1,3,0,0,0,0,0,0,0,26,0,2,0,1,0,0,1,5,0,0,0,0,0,1,1,4,0,0,1,0,0,0,0,21,0,1,0,0,0,1,0,6,0,0,0,0,0,1,1,7,0,0,0,1,0,0,0,21,0,2,0,1,0,0,0,3,0,0,0,0,0,1,1,2,0,0,0,1,0,0,0,14,0,0,1,0,0,1,1,17,2,2,0,3,0,3,2,4,0,0,0,1,0,1,1,20,0,0,1,0,0,0,0,16,1,0,1,1,1,2,6,3,0,0,0,0,0,1,0,18,1,1,0,0,0,0,0,53,14,6,6,2,2,2,11,5,0,0,0,1,1,2,0,20,0,0,0,0,0,0,0,6,1,0,0,0,0,0,2,0,0,0,0,0,0,0,0,13,0,0,0,0,1,0,0,21,6,0,0,0,1,3,1,13,0,0,2,0,0,0,1,24,0,2,1,3,0,0,1,19,2,0,0,1,0,3,5,13,0,0,2,2,1,0,1,22,0,1,0,2,0,1,4,14,0,0,0,1,0,3,2,10,0,0,0,2,1,0,0,36,0,2,0,1,0,2,4,3,0,0,0,0,0,0,0,1,0,0,1,0,0,0,0,17,0,1,0,1,0,0,0,4,0,0,0,0,0,0,1,3,0,0,1,0,0,0,0,26,0,1,0,1,0,0,0,4,0,0,0,0,0,1,1,6,0,0,1,1,0,0,0,17,0,0,1,1,0,1,0,4,0,0,0,0,0,1,2,5,1,0,1,1,0,0,0,12,0,1,1,1,0,0,1,8,1,0,0,1,0,2,1,4,0,0,0,1,0,0,1,14,0,1,1,2,0,0,1,12,2,1,0,2,0,2,1,9,1,1,0,1,0,1,3,22,0,1,1,0,1,0,0,43,10,5,7,0,3,2,9,1,0,0,0,0,1,0,0,9,0,0,0,1,0,0,0,27,6,5,3,0,1,0,4,2,0,0,0,0,1,1,0,16,0,0,1,0,1,0,0,10,2,0,0,0,1,0,5,4,0,1,0,1,0,0,0,14,0,0,0,0,0,0,0,25,6,0,1,3,0,4,5,14,1,0,1,1,0,1,2,21,0,1,0,2,1,1,1,7,0,0,0,0,0,0,2,10,0,0,0,0,0,0,0,22,0,1,0,1,1,0,2,2,0,0,0,0,0,0,0,4,0,0,0,0,1,0,0,24,0,0,0,0,0,1,3,2,0,0,0,0,0,0,0,0,0,0,0,0,0,0,0,18,0,0,0,1,0,0,0,4,0,0,0,0,0,0,1,2,0,0,0,0,0,0,0,28,0,0,0,1,0,0,0,2,0,0,0,0,0,0,1,5,0,0,0,1,0,0,0,15,0,0,0,1,0,0,0),.Dim=c(274,24)),

N=c(271,44,20,14,16,11,22,39,137,6,9,8,14,12,8,7,236,5,14,5,7,5,6,6),

Y=c(0,0,0,0,0,0,0,0,1,1,1,1,1,1,1,1,1,1,1,1,2,2,2,2,2,2,2,2,2,2,2,2,3,3,3,3,3,3,3,3,3,3,3,3,4,4,4,4,4,4,4,4,4,4,4,5,5,5,5,5,5,5,5,5,5,5,5,6,6,6,6,6,6,7,8,8,9,9,9,9,9,9,9,9,9,10,10,10,10,10,10,10,11,11,11,11,11,11,12,12,12,12,12,12,12,12,13,13,13,13,13,14,14,14,14,14,14,14,14,15,15,15,15,15,15,15,15,16,16,16,16,16,16,16,16,16,16,16,17,17,17,17,17,17,17,17,17,17,18,18,18,18,18,18,18,18,18,18,18,18,19,19,19,19,19,19,19,19,19,20,20,20,20,20,20,20,20,20,20,20,21,21,21,21,21,21,21,21,21,21,21,22,22,22,22,22,22,22,22,22,22,23,23,23,23,23,23,23,23,23,23,23,23,24,24,24,24,24,24,24,24,24,24,24,24,25,25,25,25,25,25,25,25,25,25,25,25,26,26,26,26,26,26,26,26,26,26,26,27,27,27,27,27,27,27,27,27,27,27,28,28,28,28,28,28,28,28,28,28,28,28,29,29,29),

MR=c(94,133,64,402,188.5,89.5,22.5,112,50,29,125.5,152,182,100.5,330.5,135.5,163.5,137.5,89,30.5,26,77.5,78,74.5,96,117.5,292.5,194,109,120.5,44.5,41,25,104.5,156,187.5,82.5,338.5,390,76,190,160,46,56.5,38.5,0,31,148,208.5,386.5,221,30,85.5,47,17,81.5,65.5,103.5,36.5,163,180,164.5,167.5,63.5,97,122,35.5,36,109.5,136.5,298.5,119,43,259,223,171,177,206,81,288,311,92,249,53.5,84,44,138,168,149.5,165.5,32.5,74.5,84,63,86,256.5,596,130.5,89,32.5,101,82,39.5,99,141,23,19,44.5,89.5,82,50.5,136.5,111,153,217.5,153,185.5,140,72.5,33,44,61.5,126.5,56.5,168.5,58,160.5,149,39,126,178,232.5,232,262,90,57.5,255.5,32.5,34.5,57.5,153,78.5,506.5,99.5,154,100.5,94,19,66.5,39.5,62.5,87,123.5,135.5,49,76.5,283,158.5,194.5,40,85.5,108.5,27.5,127.5,186,110,87.5,217.5,172.5,60,73.5,145.5,114,57,172.5,66.5,72,91.5,31,13.5,56.5,105,119,168.5,123,296.5,377.5,141.5,52.5,86.5,140.5,27,42,73,106,166.5,196,157,359.5,92,82,60.5,52,74.5,39.5,94.5,47,214,65.5,127,75,76.5,60,36.5,46.5,152,139,158.5,172,376,33,185,84,47.5,95,24,63.5,59.5,37.5,89,130.5,343.5,70,141.5,113.5,46,16.5,81,44,112.5,195.5,92,75,87.5,182.5,273,66.5,71,45.5,119.5,174.5,38,100,68.5,386,33,136.5,139,92.5,17.5,118.5,222,169,142.5,295.5,465,175,113.5,112,64.5,50.5,20,45.5,95),

WR=c(1,11.5,30.5,153.5,1.5,3,5.5,51.5,0,7,55,93,98,0,55,55.5,60.5,19.5,1.5,1,3.5,22.5,29.5,42,1,75.5,0,69.5,38,16,32,4.5,0,30.5,67,24,16,238,53,10.5,92.5,10.5,1,6,0,0,0,23.5,54.5,45.5,0,8,30,3,1,24.5,18.5,20.5,7,1.5,91,16,10.5,1,1,3,7.5,9,28,17.5,10,8.5,0,105.5,14.5,24.5,28.5,15.5,0,27,174,5.5,32.5,0,0,0.5,16,22.5,75,8.5,22,12,39.5,20,0.5,77,35,3.5,29,5,18,6,19.5,10.5,14.5,0,0,1,11.5,30,0,45.5,35,92,26.5,106,11.5,44,20,0,14,21.5,0,18,3,39,19.5,20.5,32,34.5,9.5,65,87,37.5,47.5,9,34.5,1,14,23.5,23.5,26,0,7.5,45.5,31,12.5,0,18,1.5,18.5,30,44.5,37,43.5,0,247.5,59.5,17,2,24,3.5,11,75,57.5,18,83.5,53.5,19,34.5,1,70.5,3.5,13.5,0,39,1.5,5.5,0,1.5,25,44,12.5,81,13.5,149,72,110.5,28,16.5,18.5,10,36,20.5,51.5,81,95.5,38,160,0,1.5,15,34.5,24.5,4,7,12,119.5,5,71,0,0,32.5,1.5,13.5,43.5,21.5,9,77,250.5,2.5,1.5,0,25.5,24,0.5,23,15.5,8.5,21,27.5,45.5,61.5,1,53.5,1.5,5.5,15.5,3,41.5,17.5,40,0,34.5,149,18.5,37,2.5,24,26.5,4.5,7,18.5,6,34,0,0,99.5,23.5,10,36.5,70,67.5,11.5,131.5,24.5,0,58.5,23.5,10.5,0.5,0,0,30.5),

Tm=c(19.11,22.02,22.49,25.68,26.54,21.57,16.86,12.82,5.73,4.47,5.47,12.20,18.65,20.76,23.12,28.59,27.55,16.96,13.97,8.92,2.67,1.99,3.96,10.86,17.74,20.44,24.50,27.06,27.85,21.65,14.09,10.63,4.23,4.89,6.84,12.54,18.32,21.76,26.05,29.02,24.90,21.19,14.29,6.32,3.59,2.92,3.19,9.54,16.12,23.61,27.77,27.40,22.84,13.98,10.17,5.13,5.44,7.72,12.87,18.70,22.34,23.88,27.82,26.25,21.64,15.24,10.26,6.97,10.12,17.22,20.95,18.84,13.35,23.24,26.39,18.77,9.42,13.65,18.05,23.73,26.04,25.31,18.64,13.84,11.26,5.26,9.50,17.05,21.53,27.78,25.02,11.36,5.87,7.30,18.03,21.86,23.18,15.04,4.38,6.43,22.54,29.33,29.81,26.53,23.05,12.74,4.32,7.96,11.96,26.84,20.37,6.15,9.74,22.68,25.55,29.23,22.89,18.72,15.32,26.54,3.91,8.32,12.24,21.21,20.67,13.10,9.97,5.76,5.45,8.81,16.42,21.17,21.97,25.76,29.01,26.49,23.96,9.15,5.13,4.58,9.84,13.08,23.56,28.33,27.19,19.03,14.50,9.28,6.16,3.74,5.05,12.14,17.63,21.68,28.19,28.95,27.71,19.04,13.97,9.27,5.24,7.88,13.91,19.39,21.77,28.57,28.96,25.82,18.59,5.87,9.79,13.63,18.51,23.03,27.33,28.20,26.71,20.51,10.38,8.47,3.99,4.86,5.87,14.13,18.85,22.55,23.72,25.83,17.66,15.15,11.37,4.69,6.27,8.31,14.87,24.02,27.72,26.16,20.04,14.15,9.74,4.52,4.96,6.83,13.01,18.53,21.67,25.23,28.56,27.31,20.96,13.77,8.79,3.78,4.29,6.97,10.94,18.42,21.54,25.46,29.39,25.09,20.39,14.00,11.01,5.49,7.08,7.54,13.15,17.50,20.73,24.72,29.05,27.32,21.18,13.41,10.25,5.13,3.58,7.78,13.21,17.35,27.89,28.04,24.96,20.14,12.09,9.68,4.79,6.41,8.63,13.45,19.36,21.64,27.40,23.96,20.65,16.15,10.47,4.98,6.63,9.10,11.11,15.02,21.05,26.95,29.98,29.03,19.22,12.44,10.65,2.88,5.38,7.06))

#initial value for chain 1

list(a=c(-2.611,-3.502,-5.775,-6.412,-2.884,-5.247,-2.525,-1.944,-2.843,-3.433,-5.252,-2.871,-2.386,-4.26,-3.815,-2.974,-2.039,-3.922,-2.839,-2.131,-1.658,-3.045,-2.468,-1.865),

b=structure(.Data=c(0.1097,0.2446,0.3924,0.4863,0.04602,0.2659,0.08384,0.08675,0.02683,0.1158,0.254,-0.02422,-0.01566,0.1125,0.1788,0.06505,0.01085,0.1713,-0.05516,0.05812,-0.01667,0.1318,-0.05861,-0.003291,0.00884,0.01447,0.01846,0.0219,0.01617,0.01178,0.007165,0.006877,0.004923,0.002006,0.004203,-0.002161,0.008223,0.0124,-3.383E-4,0.004431,0.001419,-0.00487,0.002612,0.001322,0.001814,-0.00239,0.006622,0.008835,-0.005379,-0.01315,-0.01234,-0.01636,-0.008395,-0.008092,-0.001839,-0.003146,0.001162,6.412E-4,0.004257,-0.002859,-0.005118,-0.00367,0.01385,0.006379,-0.001477,5.471E-4,5.766E-4,0.005837,-0.004666,-0.00213,0.003583,1.997E-4,0.01309,0.009808,0.04548,0.09118,0.008967,0.03041,0.01067,0.004464,0.02394,0.02619,0.005054,0.01054,-0.02746,0.02439,0.04078,0.03459,0.02779,-0.02335,0.02381,-0.0161,0.02485,0.02737,0.01715,0.07578),.Dim=c(4,24)),

d=c(0.1032,0.3886,0.4004,0.3231,0.2777,0.8772,0.1226,0.3096,0.4136,0.1347,0.06658,0.3613,0.1728,0.1095,0.3119,0.3434,0.4457,0.5716,0.2413,0.2722,0.1782,0.209,0.1785,0.2044,0.2381,0.1441,0.2326,0.4424,0.497,0.1776,0.3047,0.252,0.2935,0.3729,0.549,0.3616,0.3333,0.3891,0.1582,0.2296,0.6405,0.5698,0.6661,0.5124,0.5676,0.4817,0.5499,0.2518,0.3235,0.2475,0.1503,0.3055,0.3451,0.3188,0.2979,0.3862,0.1464,0.3206,0.6083,0.2228,0.5618,0.7384,0.4089,0.5874,0.7326,0.8298,0.9619,0.5197,0.2632,0.3065,0.2571,0.8373,0.7404,0.5254,0.3641,0.9672,0.4429,0.04455,0.4493,0.3692,0.4068,0.7897,0.591,0.7647,0.6828,0.9341,0.4803,0.7671,0.4852,0.3719,0.6287,0.8692,0.8089,0.574,0.5749,0.495,0.152,0.4362,0.7771,0.5317,0.7532,0.3441,0.315,0.511,0.9256,0.8231,0.4108,0.7161,0.5886,0.3196,0.7147,0.4532,0.6374,0.9867,0.3699,0.1746,0.8282,0.9886,0.7919,0.5305,0.7379,0.02267,0.2864,0.9136,0.2762,0.8787,0.7595,0.3226,0.7114,0.7259,0.6789,0.4087,0.68,0.3459,0.5382,0.4593,0.4419,0.7819,0.5305,0.6701,0.4209,0.7771,0.1428,0.9946,0.9447,0.7738,0.988,0.7757,0.8191,0.6337,0.4364,0.7938,0.8461,0.6983,0.782,0.3591,0.3375,0.7831,0.5028,0.8052,0.5381,0.4601,0.4127,0.5008,0.4104,0.4059,0.5118,0.6541,0.5887,0.5214,0.1954,0.5053,0.3142,0.647,0.4928,0.2551,0.3328,0.6209,0.7387,0.4665,0.1562,0.2608,0.2355,0.5365,0.6295,0.5224,0.2013,0.3883,0.6464,0.89,0.5475,0.2393,0.6445,0.5137,0.2353,0.6921,0.5504,0.4985,0.1487,0.5186,0.4071,0.6272,0.5921,0.4486,0.1451,0.2594,0.3866,0.5621,0.7567,0.9276,0.9579,0.9902,0.7106,0.4478,0.6028,0.4581,0.2433,0.3469,0.6489,0.3658,0.2472,0.5634,0.7351,0.4194,0.5823,0.7145,0.3114,0.2833,0.3057,0.2482,0.6363,0.3787,0.1105,0.7579,0.9333,0.9425,0.225,0.5461,0.1744,0.5559,0.2531,0.1696,0.431,0.5749,0.8292,0.3541,0.7895,0.6299,0.7154,0.6052,0.4655,0.4476,0.661,0.6759,0.3056,0.3326,0.5034,0.968,0.9261,0.5433,0.5769,0.3836,0.3553,0.5002,0.2257,0.2035,0.2262,0.2119,0.8548,0.6301,0.6444,0.5633,0.7693,0.4504),

present=structure(.Data=c(15.0,1.0,0.0,0.0,0.0,0.0,3.0,6.0,3.0,0.0,0.0,2.0,0.0,0.0,0.0,0.0,13.0,0.0,0.0,0.0,1.0,0.0,0.0,2.0,31.0,3.0,0.0,1.0,0.0,0.0,4.0,7.0,3.0,0.0,0.0,0.0,1.0,1.0,0.0,0.0,21.0,0.0,1.0,1.0,2.0,0.0,1.0,0.0,18.0,2.0,1.0,0.0,0.0,0.0,0.0,6.0,1.0,0.0,1.0,0.0,2.0,0.0,0.0,1.0,19.0,0.0,1.0,1.0,2.0,0.0,0.0,0.0,144.0,33.0,14.0,6.0,9.0,3.0,5.0,22.0,29.0,0.0,2.0,1.0,4.0,4.0,1.0,4.0,28.0,0.0,0.0,2.0,0.0,0.0,1.0,3.0,74.0,17.0,9.0,4.0,4.0,1.0,3.0,13.0,8.0,0.0,1.0,0.0,4.0,3.0,0.0,0.0,15.0,0.0,0.0,0.0,2.0,0.0,0.0,0.0,23.0,5.0,0.0,0.0,0.0,0.0,3.0,6.0,5.0,0.0,0.0,1.0,1.0,0.0,0.0,1.0,12.0,0.0,0.0,1.0,1.0,0.0,0.0,0.0,9.0,0.0,0.0,0.0,0.0,0.0,2.0,0.0,2.0,0.0,0.0,0.0,0.0,0.0,0.0,1.0,22.0,0.0,0.0,1.0,1.0,1.0,0.0,0.0,11.0,0.0,0.0,0.0,0.0,0.0,4.0,1.0,6.0,0.0,0.0,1.0,0.0,1.0,0.0,0.0,18.0,0.0,2.0,2.0,0.0,0.0,0.0,1.0,6.0,0.0,0.0,0.0,0.0,0.0,1.0,1.0,3.0,0.0,0.0,1.0,3.0,1.0,0.0,0.0,18.0,0.0,1.0,0.0,0.0,0.0,0.0,0.0,3.0,0.0,0.0,0.0,0.0,0.0,0.0,3.0,2.0,0.0,0.0,0.0,3.0,0.0,0.0,0.0,22.0,1.0,2.0,0.0,1.0,0.0,0.0,0.0,3.0,0.0,0.0,0.0,0.0,0.0,2.0,0.0,7.0,0.0,0.0,1.0,1.0,0.0,0.0,0.0,15.0,0.0,2.0,1.0,1.0,0.0,2.0,0.0,11.0,0.0,0.0,0.0,0.0,0.0,3.0,4.0,3.0,0.0,0.0,0.0,2.0,0.0,0.0,0.0,23.0,0.0,1.0,0.0,0.0,0.0,0.0,0.0,30.0,2.0,0.0,0.0,3.0,0.0,2.0,5.0,7.0,0.0,0.0,1.0,1.0,1.0,0.0,0.0,14.0,0.0,2.0,1.0,0.0,0.0,0.0,1.0,17.0,4.0,0.0,0.0,0.0,0.0,4.0,6.0,2.0,0.0,0.0,1.0,1.0,0.0,0.0,1.0,26.0,0.0,0.0,1.0,0.0,1.0,0.0,0.0,118.0,29.0,9.0,9.0,8.0,0.0,9.0,21.0,19.0,0.0,0.0,0.0,6.0,0.0,0.0,1.0,21.0,0.0,1.0,2.0,1.0,0.0,0.0,0.0,58.0,12.0,1.0,2.0,1.0,1.0,4.0,15.0,12.0,1.0,0.0,0.0,1.0,2.0,0.0,0.0,19.0,3.0,0.0,2.0,1.0,0.0,0.0,0.0,63.0,22.0,3.0,3.0,3.0,4.0,2.0,13.0,17.0,1.0,0.0,1.0,4.0,0.0,2.0,4.0,13.0,0.0,1.0,1.0,0.0,0.0,1.0,1.0,21.0,2.0,0.0,0.0,3.0,0.0,1.0,3.0,10.0,0.0,0.0,0.0,0.0,1.0,0.0,0.0,7.0,1.0,0.0,0.0,1.0,0.0,0.0,0.0,14.0,1.0,0.0,0.0,1.0,0.0,2.0,4.0,9.0,0.0,0.0,0.0,3.0,0.0,1.0,1.0,20.0,0.0,0.0,0.0,2.0,0.0,0.0,1.0,4.0,0.0,0.0,0.0,0.0,0.0,0.0,0.0,0.0,0.0,0.0,0.0,0.0,0.0,0.0,0.0,15.0,0.0,0.0,0.0,1.0,0.0,0.0,0.0,2.0,0.0,0.0,0.0,0.0,0.0,0.0,1.0,3.0,0.0,0.0,1.0,1.0,0.0,0.0,0.0,17.0,0.0,0.0,1.0,0.0,0.0,0.0,0.0,2.0,0.0,0.0,0.0,1.0,0.0,0.0,2.0,1.0,0.0,0.0,0.0,0.0,0.0,0.0,0.0,13.0,0.0,1.0,0.0,2.0,0.0,0.0,0.0,4.0,0.0,0.0,0.0,0.0,0.0,0.0,1.0,7.0,0.0,0.0,1.0,2.0,0.0,0.0,0.0,15.0,0.0,0.0,0.0,2.0,0.0,0.0,1.0,6.0,0.0,0.0,0.0,0.0,0.0,0.0,2.0,4.0,0.0,0.0,1.0,1.0,0.0,0.0,0.0,10.0,1.0,0.0,0.0,1.0,0.0,0.0,0.0,13.0,1.0,0.0,0.0,0.0,0.0,2.0,7.0,5.0,0.0,0.0,0.0,1.0,0.0,0.0,0.0,21.0,0.0,2.0,2.0,1.0,0.0,0.0,0.0,21.0,1.0,0.0,0.0,1.0,0.0,1.0,12.0,6.0,0.0,0.0,0.0,0.0,0.0,1.0,1.0,20.0,0.0,0.0,2.0,2.0,0.0,0.0,0.0,133.0,33.0,14.0,8.0,10.0,2.0,8.0,17.0,16.0,0.0,2.0,0.0,3.0,3.0,1.0,1.0,22.0,0.0,1.0,2.0,1.0,1.0,0.0,3.0,67.0,16.0,6.0,4.0,2.0,0.0,3.0,14.0,15.0,0.0,1.0,0.0,4.0,0.0,1.0,0.0,19.0,0.0,0.0,1.0,1.0,0.0,1.0,1.0,41.0,10.0,2.0,0.0,2.0,0.0,2.0,10.0,8.0,1.0,0.0,1.0,1.0,1.0,1.0,2.0,17.0,2.0,0.0,0.0,1.0,0.0,0.0,0.0,35.0,5.0,0.0,0.0,2.0,0.0,3.0,9.0,5.0,0.0,0.0,1.0,0.0,0.0,0.0,0.0,19.0,1.0,0.0,1.0,1.0,1.0,0.0,0.0,7.0,0.0,0.0,0.0,0.0,0.0,0.0,1.0,2.0,0.0,0.0,0.0,1.0,0.0,0.0,0.0,20.0,0.0,0.0,0.0,0.0,0.0,0.0,0.0,2.0,0.0,0.0,0.0,0.0,0.0,1.0,2.0,2.0,0.0,0.0,0.0,1.0,0.0,0.0,0.0,13.0,0.0,1.0,0.0,3.0,0.0,0.0,0.0,6.0,0.0,0.0,0.0,0.0,0.0,0.0,0.0,3.0,0.0,0.0,1.0,2.0,0.0,0.0,1.0,17.0,0.0,1.0,0.0,0.0,0.0,0.0,1.0,3.0,0.0,0.0,0.0,0.0,0.0,0.0,3.0,3.0,0.0,0.0,1.0,0.0,0.0,0.0,0.0,20.0,0.0,1.0,1.0,1.0,0.0,1.0,0.0,6.0,0.0,0.0,0.0,0.0,0.0,1.0,3.0,5.0,0.0,0.0,1.0,4.0,0.0,0.0,0.0,23.0,0.0,2.0,0.0,1.0,0.0,0.0,0.0,16.0,0.0,0.0,0.0,1.0,0.0,5.0,5.0,4.0,0.0,0.0,0.0,5.0,0.0,0.0,0.0,25.0,0.0,2.0,1.0,3.0,1.0,1.0,1.0,13.0,0.0,0.0,0.0,0.0,0.0,2.0,1.0,7.0,0.0,0.0,2.0,2.0,0.0,0.0,0.0,25.0,0.0,0.0,0.0,0.0,2.0,0.0,0.0,59.0,6.0,1.0,2.0,5.0,0.0,7.0,12.0,21.0,0.0,0.0,0.0,4.0,2.0,2.0,2.0,23.0,0.0,1.0,2.0,0.0,1.0,2.0,0.0,180.0,37.0,19.0,14.0,14.0,6.0,10.0,24.0,25.0,1.0,2.0,0.0,5.0,7.0,2.0,0.0,34.0,0.0,0.0,1.0,1.0,1.0,1.0,3.0,41.0,8.0,3.0,1.0,0.0,2.0,8.0,13.0,5.0,0.0,1.0,1.0,3.0,0.0,1.0,0.0,33.0,0.0,0.0,1.0,1.0,0.0,0.0,0.0,36.0,7.0,4.0,1.0,3.0,0.0,1.0,5.0,12.0,0.0,1.0,1.0,1.0,0.0,0.0,3.0,22.0,0.0,0.0,1.0,0.0,0.0,0.0,0.0,47.0,14.0,2.0,0.0,3.0,0.0,3.0,8.0,6.0,0.0,0.0,1.0,2.0,0.0,0.0,0.0,11.0,0.0,0.0,1.0,1.0,1.0,0.0,0.0,7.0,0.0,0.0,0.0,2.0,0.0,1.0,3.0,4.0,0.0,0.0,1.0,0.0,0.0,0.0,0.0,20.0,1.0,0.0,0.0,0.0,0.0,1.0,0.0,5.0,0.0,0.0,0.0,0.0,0.0,0.0,1.0,2.0,0.0,0.0,0.0,0.0,0.0,0.0,0.0,18.0,0.0,0.0,0.0,1.0,0.0,1.0,0.0,2.0,0.0,0.0,0.0,0.0,0.0,0.0,1.0,1.0,0.0,0.0,0.0,1.0,0.0,0.0,0.0,12.0,0.0,1.0,0.0,1.0,0.0,0.0,0.0,3.0,0.0,0.0,0.0,0.0,0.0,0.0,4.0,1.0,0.0,0.0,0.0,0.0,0.0,0.0,0.0,14.0,0.0,1.0,0.0,1.0,0.0,0.0,0.0,3.0,0.0,0.0,0.0,0.0,0.0,0.0,1.0,2.0,0.0,0.0,0.0,0.0,0.0,0.0,0.0,18.0,0.0,0.0,0.0,1.0,0.0,1.0,1.0,14.0,0.0,0.0,0.0,0.0,0.0,0.0,5.0,6.0,0.0,0.0,0.0,0.0,0.0,0.0,0.0,29.0,0.0,1.0,0.0,0.0,1.0,1.0,0.0,26.0,2.0,1.0,0.0,1.0,0.0,2.0,7.0,5.0,0.0,0.0,1.0,3.0,1.0,0.0,1.0,18.0,0.0,1.0,1.0,1.0,0.0,0.0,0.0,152.0,36.0,14.0,13.0,14.0,4.0,9.0,27.0,32.0,1.0,0.0,0.0,5.0,5.0,1.0,1.0,44.0,0.0,0.0,0.0,0.0,0.0,0.0,1.0,104.0,36.0,10.0,7.0,7.0,1.0,7.0,17.0,15.0,0.0,1.0,0.0,2.0,0.0,0.0,0.0,30.0,0.0,0.0,0.0,0.0,1.0,0.0,0.0,25.0,3.0,1.0,0.0,1.0,1.0,3.0,8.0,3.0,0.0,0.0,0.0,0.0,0.0,0.0,0.0,27.0,1.0,0.0,2.0,0.0,2.0,0.0,0.0,23.0,3.0,0.0,0.0,1.0,0.0,3.0,6.0,5.0,0.0,1.0,0.0,0.0,0.0,0.0,0.0,30.0,0.0,0.0,0.0,0.0,0.0,0.0,0.0,11.0,1.0,0.0,0.0,0.0,0.0,0.0,7.0,4.0,0.0,0.0,0.0,2.0,0.0,0.0,0.0,14.0,0.0,1.0,0.0,0.0,0.0,0.0,0.0,3.0,1.0,0.0,0.0,0.0,0.0,0.0,0.0,2.0,0.0,0.0,0.0,2.0,0.0,0.0,0.0,14.0,0.0,0.0,0.0,1.0,0.0,0.0,0.0,3.0,0.0,0.0,0.0,0.0,0.0,0.0,2.0,1.0,0.0,0.0,0.0,1.0,0.0,0.0,0.0,10.0,0.0,0.0,1.0,0.0,0.0,1.0,0.0,7.0,0.0,0.0,0.0,1.0,0.0,0.0,1.0,4.0,0.0,0.0,2.0,1.0,0.0,0.0,0.0,11.0,0.0,0.0,0.0,4.0,0.0,0.0,0.0,4.0,0.0,0.0,0.0,0.0,0.0,0.0,3.0,3.0,0.0,0.0,1.0,1.0,0.0,0.0,0.0,17.0,0.0,2.0,0.0,2.0,0.0,1.0,0.0,5.0,0.0,0.0,0.0,0.0,0.0,0.0,3.0,1.0,0.0,0.0,1.0,0.0,1.0,0.0,0.0,14.0,1.0,1.0,0.0,0.0,1.0,0.0,0.0,37.0,5.0,2.0,0.0,3.0,1.0,2.0,9.0,6.0,0.0,0.0,0.0,0.0,1.0,0.0,1.0,31.0,0.0,0.0,1.0,3.0,1.0,0.0,1.0,31.0,6.0,2.0,1.0,3.0,2.0,2.0,9.0,13.0,0.0,2.0,1.0,3.0,1.0,1.0,1.0,22.0,1.0,0.0,1.0,0.0,0.0,1.0,1.0,53.0,13.0,5.0,2.0,2.0,3.0,4.0,8.0,10.0,1.0,3.0,1.0,1.0,0.0,0.0,0.0,18.0,0.0,1.0,0.0,1.0,1.0,0.0,0.0,86.0,22.0,7.0,4.0,3.0,0.0,3.0,18.0,7.0,1.0,0.0,0.0,1.0,1.0,0.0,0.0,32.0,2.0,0.0,1.0,1.0,0.0,0.0,0.0,24.0,5.0,1.0,1.0,0.0,0.0,0.0,7.0,5.0,0.0,0.0,1.0,1.0,0.0,0.0,0.0,26.0,0.0,0.0,0.0,0.0,0.0,0.0,0.0,22.0,3.0,0.0,0.0,2.0,0.0,2.0,4.0,7.0,0.0,0.0,1.0,0.0,0.0,0.0,0.0,21.0,1.0,3.0,2.0,0.0,1.0,0.0,1.0,16.0,1.0,0.0,0.0,4.0,0.0,1.0,3.0,5.0,0.0,0.0,0.0,1.0,0.0,0.0,0.0,14.0,0.0,1.0,0.0,0.0,0.0,0.0,0.0,8.0,0.0,0.0,0.0,1.0,0.0,2.0,2.0,3.0,0.0,0.0,1.0,1.0,0.0,0.0,0.0,15.0,0.0,1.0,0.0,0.0,1.0,0.0,1.0,4.0,0.0,0.0,0.0,0.0,0.0,0.0,2.0,4.0,0.0,0.0,1.0,1.0,0.0,0.0,0.0,18.0,0.0,2.0,0.0,1.0,0.0,0.0,0.0,12.0,0.0,0.0,0.0,1.0,0.0,1.0,4.0,7.0,0.0,0.0,0.0,2.0,0.0,0.0,1.0,20.0,0.0,1.0,0.0,1.0,0.0,0.0,0.0,20.0,4.0,0.0,0.0,1.0,0.0,3.0,4.0,12.0,0.0,1.0,0.0,3.0,0.0,0.0,0.0,31.0,0.0,0.0,1.0,0.0,0.0,0.0,1.0,99.0,25.0,6.0,4.0,10.0,0.0,4.0,15.0,21.0,0.0,2.0,0.0,5.0,3.0,0.0,4.0,26.0,0.0,1.0,0.0,1.0,0.0,1.0,0.0,20.0,2.0,0.0,0.0,2.0,0.0,1.0,5.0,10.0,0.0,0.0,0.0,2.0,0.0,0.0,2.0,16.0,0.0,1.0,0.0,1.0,0.0,0.0,1.0,18.0,1.0,0.0,0.0,2.0,0.0,2.0,3.0,5.0,0.0,0.0,0.0,1.0,0.0,0.0,0.0,19.0,0.0,0.0,1.0,1.0,0.0,0.0,1.0,59.0,13.0,2.0,1.0,4.0,0.0,6.0,15.0,11.0,0.0,0.0,0.0,1.0,2.0,3.0,0.0,20.0,0.0,0.0,0.0,0.0,0.0,0.0,0.0,92.0,33.0,8.0,7.0,3.0,2.0,6.0,18.0,12.0,0.0,1.0,0.0,1.0,0.0,0.0,0.0,33.0,0.0,0.0,1.0,0.0,0.0,0.0,0.0,41.0,11.0,0.0,1.0,1.0,0.0,5.0,5.0,12.0,0.0,0.0,2.0,0.0,0.0,1.0,0.0,23.0,0.0,1.0,0.0,2.0,1.0,1.0,1.0,11.0,0.0,0.0,0.0,0.0,0.0,0.0,2.0,7.0,0.0,0.0,1.0,2.0,0.0,0.0,0.0,26.0,0.0,0.0,1.0,2.0,0.0,1.0,0.0,25.0,1.0,0.0,0.0,2.0,1.0,1.0,9.0,9.0,0.0,0.0,1.0,3.0,0.0,0.0,0.0,19.0,0.0,5.0,0.0,1.0,0.0,0.0,3.0,9.0,3.0,1.0,0.0,2.0,0.0,5.0,2.0,5.0,0.0,0.0,0.0,3.0,1.0,0.0,0.0,24.0,0.0,0.0,0.0,0.0,0.0,1.0,2.0,105.0,25.0,7.0,6.0,7.0,1.0,9.0,17.0,23.0,0.0,0.0,1.0,1.0,2.0,0.0,3.0,40.0,0.0,0.0,2.0,3.0,0.0,1.0,2.0,84.0,24.0,7.0,8.0,6.0,2.0,7.0,21.0,11.0,0.0,2.0,0.0,1.0,2.0,3.0,0.0,36.0,1.0,0.0,1.0,1.0,1.0,2.0,2.0,26.0,7.0,0.0,0.0,1.0,0.0,0.0,6.0,14.0,1.0,2.0,1.0,3.0,0.0,0.0,1.0,36.0,0.0,1.0,1.0,0.0,2.0,0.0,1.0,49.0,9.0,0.0,0.0,5.0,0.0,6.0,13.0,12.0,0.0,0.0,3.0,4.0,2.0,0.0,3.0,33.0,1.0,2.0,0.0,0.0,0.0,1.0,0.0,5.0,0.0,0.0,0.0,0.0,0.0,1.0,2.0,6.0,0.0,0.0,1.0,1.0,1.0,0.0,0.0,18.0,0.0,0.0,1.0,1.0,0.0,0.0,1.0,7.0,0.0,0.0,0.0,0.0,0.0,0.0,2.0,7.0,0.0,0.0,1.0,2.0,0.0,0.0,0.0,33.0,0.0,2.0,0.0,0.0,0.0,0.0,0.0,3.0,0.0,0.0,0.0,0.0,0.0,0.0,1.0,1.0,0.0,0.0,1.0,1.0,0.0,0.0,0.0,13.0,0.0,0.0,0.0,1.0,0.0,1.0,0.0,5.0,0.0,0.0,0.0,0.0,0.0,2.0,3.0,4.0,0.0,0.0,1.0,3.0,0.0,0.0,0.0,20.0,0.0,2.0,2.0,1.0,0.0,1.0,3.0,22.0,4.0,0.0,0.0,3.0,0.0,3.0,8.0,4.0,0.0,0.0,0.0,2.0,0.0,1.0,1.0,24.0,0.0,0.0,1.0,1.0,1.0,1.0,1.0,26.0,4.0,0.0,2.0,4.0,0.0,1.0,5.0,12.0,0.0,1.0,1.0,2.0,1.0,3.0,0.0,17.0,0.0,0.0,2.0,1.0,0.0,0.0,0.0,76.0,26.0,10.0,8.0,5.0,2.0,3.0,15.0,10.0,2.0,1.0,0.0,2.0,1.0,0.0,0.0,28.0,1.0,0.0,1.0,1.0,0.0,0.0,0.0,12.0,2.0,0.0,0.0,1.0,0.0,0.0,3.0,2.0,0.0,0.0,0.0,1.0,0.0,0.0,0.0,34.0,0.0,1.0,0.0,1.0,1.0,0.0,0.0,9.0,0.0,0.0,0.0,0.0,0.0,1.0,1.0,7.0,0.0,0.0,0.0,1.0,0.0,0.0,0.0,19.0,0.0,1.0,0.0,1.0,2.0,0.0,1.0,2.0,0.0,0.0,0.0,0.0,0.0,0.0,1.0,3.0,0.0,0.0,1.0,0.0,0.0,0.0,0.0,35.0,0.0,2.0,0.0,1.0,0.0,1.0,0.0,4.0,0.0,0.0,0.0,0.0,0.0,1.0,2.0,3.0,0.0,0.0,0.0,1.0,0.0,0.0,0.0,25.0,0.0,2.0,0.0,0.0,0.0,1.0,0.0,15.0,1.0,0.0,0.0,1.0,0.0,2.0,3.0,4.0,0.0,0.0,0.0,1.0,0.0,1.0,0.0,25.0,0.0,0.0,0.0,1.0,1.0,1.0,1.0,60.0,13.0,2.0,3.0,3.0,3.0,2.0,14.0,23.0,2.0,1.0,0.0,3.0,1.0,2.0,2.0,27.0,0.0,0.0,2.0,1.0,0.0,0.0,2.0,250.0,43.0,20.0,14.0,16.0,10.0,19.0,35.0,58.0,2.0,4.0,0.0,10.0,10.0,1.0,3.0,46.0,0.0,2.0,1.0,0.0,0.0,3.0,5.0,14.0,1.0,0.0,0.0,2.0,1.0,1.0,4.0,6.0,0.0,0.0,0.0,1.0,0.0,0.0,0.0,19.0,0.0,2.0,1.0,1.0,1.0,1.0,1.0,3.0,0.0,0.0,0.0,0.0,0.0,0.0,2.0,2.0,0.0,0.0,0.0,0.0,0.0,0.0,0.0,19.0,0.0,1.0,0.0,0.0,0.0,2.0,0.0,1.0,0.0,0.0,0.0,0.0,0.0,0.0,1.0,6.0,0.0,0.0,0.0,1.0,0.0,0.0,0.0,17.0,0.0,1.0,0.0,1.0,0.0,0.0,0.0,32.0,2.0,1.0,0.0,2.0,1.0,2.0,6.0,9.0,0.0,0.0,0.0,2.0,2.0,0.0,1.0,28.0,1.0,2.0,1.0,3.0,0.0,1.0,1.0,59.0,13.0,7.0,4.0,0.0,2.0,4.0,15.0,6.0,0.0,0.0,1.0,1.0,2.0,2.0,0.0,28.0,1.0,0.0,0.0,1.0,1.0,1.0,0.0,33.0,3.0,3.0,1.0,1.0,2.0,3.0,7.0,13.0,2.0,3.0,1.0,0.0,0.0,1.0,0.0,24.0,0.0,0.0,2.0,2.0,2.0,0.0,0.0,29.0,6.0,1.0,1.0,0.0,0.0,1.0,4.0,8.0,2.0,2.0,0.0,2.0,0.0,2.0,0.0,40.0,0.0,1.0,0.0,3.0,1.0,0.0,0.0,37.0,7.0,1.0,1.0,5.0,0.0,5.0,6.0,13.0,1.0,1.0,0.0,0.0,0.0,2.0,1.0,35.0,0.0,1.0,1.0,2.0,2.0,1.0,1.0,5.0,1.0,0.0,0.0,0.0,0.0,0.0,0.0,1.0,0.0,0.0,1.0,0.0,0.0,0.0,0.0,19.0,0.0,0.0,0.0,1.0,1.0,1.0,0.0,3.0,0.0,0.0,0.0,1.0,0.0,0.0,2.0,6.0,0.0,0.0,0.0,0.0,0.0,0.0,0.0,21.0,0.0,0.0,0.0,2.0,0.0,0.0,0.0,1.0,0.0,0.0,0.0,0.0,0.0,0.0,2.0,6.0,1.0,0.0,1.0,2.0,0.0,0.0,0.0,23.0,0.0,2.0,0.0,1.0,0.0,1.0,0.0,8.0,1.0,0.0,0.0,0.0,0.0,1.0,3.0,5.0,0.0,0.0,1.0,1.0,0.0,0.0,1.0,21.0,0.0,1.0,1.0,0.0,0.0,0.0,1.0,35.0,3.0,2.0,3.0,0.0,0.0,4.0,4.0,9.0,1.0,0.0,1.0,1.0,0.0,0.0,1.0,39.0,2.0,1.0,2.0,1.0,0.0,1.0,0.0,10.0,1.0,0.0,0.0,0.0,1.0,1.0,2.0,5.0,0.0,0.0,0.0,0.0,0.0,0.0,0.0,34.0,0.0,1.0,1.0,1.0,1.0,1.0,1.0,4.0,0.0,0.0,0.0,0.0,0.0,0.0,1.0,9.0,0.0,0.0,0.0,4.0,0.0,0.0,0.0,27.0,0.0,1.0,1.0,0.0,0.0,1.0,0.0,6.0,0.0,0.0,0.0,1.0,0.0,0.0,3.0,4.0,0.0,0.0,1.0,0.0,0.0,0.0,0.0,21.0,0.0,0.0,0.0,2.0,0.0,1.0,0.0,29.0,3.0,3.0,0.0,3.0,0.0,3.0,8.0,14.0,1.0,1.0,0.0,0.0,0.0,3.0,1.0,26.0,0.0,1.0,0.0,1.0,0.0,1.0,1.0,96.0,25.0,5.0,7.0,2.0,3.0,6.0,17.0,9.0,0.0,1.0,2.0,0.0,0.0,1.0,1.0,31.0,0.0,0.0,4.0,2.0,2.0,2.0,0.0,48.0,13.0,5.0,5.0,0.0,1.0,5.0,9.0,15.0,1.0,1.0,2.0,2.0,0.0,4.0,1.0,37.0,1.0,0.0,0.0,1.0,1.0,0.0,2.0,72.0,15.0,2.0,2.0,3.0,1.0,5.0,14.0,14.0,1.0,1.0,1.0,2.0,1.0,0.0,0.0,25.0,0.0,1.0,0.0,1.0,0.0,1.0,1.0,30.0,3.0,0.0,0.0,4.0,1.0,3.0,6.0,14.0,1.0,0.0,1.0,1.0,1.0,0.0,1.0,20.0,0.0,2.0,0.0,1.0,0.0,0.0,3.0,5.0,0.0,0.0,0.0,0.0,0.0,0.0,1.0,9.0,0.0,0.0,0.0,1.0,0.0,0.0,0.0,30.0,0.0,0.0,1.0,2.0,0.0,2.0,1.0,29.0,4.0,1.0,1.0,0.0,0.0,0.0,4.0,5.0,0.0,1.0,1.0,0.0,0.0,1.0,0.0,30.0,0.0,0.0,1.0,1.0,1.0,0.0,0.0,3.0,0.0,0.0,0.0,0.0,0.0,0.0,1.0,4.0,0.0,0.0,0.0,0.0,0.0,0.0,0.0,31.0,0.0,1.0,0.0,1.0,0.0,1.0,0.0,2.0,0.0,0.0,0.0,0.0,0.0,2.0,2.0,10.0,0.0,0.0,0.0,2.0,0.0,0.0,0.0,23.0,0.0,0.0,0.0,2.0,0.0,0.0,0.0,12.0,0.0,0.0,0.0,0.0,0.0,2.0,5.0,7.0,0.0,0.0,0.0,1.0,0.0,0.0,0.0,34.0,0.0,0.0,1.0,1.0,0.0,1.0,0.0,23.0,3.0,2.0,0.0,1.0,0.0,2.0,11.0,6.0,1.0,0.0,0.0,1.0,1.0,1.0,0.0,22.0,0.0,0.0,1.0,1.0,0.0,1.0,1.0,50.0,8.0,1.0,0.0,3.0,0.0,5.0,10.0,10.0,1.0,0.0,1.0,4.0,2.0,0.0,0.0,45.0,1.0,0.0,1.0,1.0,1.0,0.0,0.0,8.0,0.0,0.0,0.0,0.0,0.0,2.0,1.0,4.0,0.0,0.0,0.0,0.0,1.0,1.0,0.0,29.0,0.0,2.0,1.0,1.0,0.0,0.0,0.0,7.0,0.0,0.0,0.0,0.0,0.0,3.0,4.0,8.0,0.0,0.0,0.0,1.0,0.0,0.0,0.0,23.0,0.0,1.0,0.0,1.0,1.0,1.0,2.0,5.0,0.0,0.0,0.0,0.0,0.0,0.0,5.0,9.0,0.0,0.0,0.0,1.0,0.0,0.0,0.0,30.0,0.0,2.0,0.0,1.0,0.0,0.0,0.0,5.0,0.0,0.0,0.0,0.0,0.0,0.0,1.0,3.0,0.0,0.0,1.0,2.0,0.0,0.0,0.0,24.0,0.0,1.0,0.0,1.0,0.0,1.0,0.0,5.0,0.0,0.0,0.0,0.0,0.0,0.0,1.0,5.0,0.0,0.0,0.0,1.0,0.0,0.0,0.0,25.0,0.0,1.0,0.0,1.0,0.0,3.0,0.0,21.0,5.0,0.0,0.0,1.0,0.0,4.0,6.0,5.0,0.0,0.0,1.0,3.0,0.0,0.0,0.0,25.0,0.0,1.0,1.0,2.0,1.0,1.0,2.0,51.0,6.0,3.0,1.0,6.0,1.0,3.0,13.0,14.0,0.0,0.0,0.0,3.0,0.0,0.0,1.0,44.0,0.0,1.0,2.0,3.0,1.0,3.0,2.0,54.0,6.0,1.0,2.0,5.0,0.0,3.0,13.0,13.0,0.0,1.0,0.0,3.0,3.0,0.0,2.0,19.0,0.0,0.0,0.0,1.0,0.0,1.0,2.0,106.0,31.0,13.0,9.0,6.0,4.0,4.0,15.0,18.0,0.0,0.0,1.0,4.0,5.0,0.0,1.0,36.0,0.0,2.0,1.0,2.0,0.0,0.0,3.0,43.0,13.0,2.0,3.0,0.0,1.0,5.0,9.0,4.0,0.0,1.0,0.0,0.0,0.0,2.0,0.0,31.0,0.0,2.0,4.0,1.0,1.0,1.0,1.0,30.0,4.0,0.0,1.0,0.0,0.0,2.0,8.0,7.0,1.0,1.0,0.0,2.0,0.0,1.0,1.0,35.0,1.0,0.0,1.0,1.0,1.0,0.0,1.0,95.0,23.0,12.0,6.0,8.0,1.0,8.0,14.0,15.0,0.0,0.0,0.0,2.0,1.0,0.0,1.0,29.0,0.0,2.0,1.0,3.0,0.0,1.0,2.0,5.0,0.0,0.0,0.0,1.0,0.0,1.0,1.0,4.0,0.0,0.0,1.0,0.0,0.0,0.0,0.0,29.0,0.0,0.0,0.0,1.0,0.0,0.0,1.0,3.0,0.0,0.0,0.0,0.0,0.0,0.0,1.0,3.0,0.0,0.0,0.0,0.0,0.0,0.0,0.0,24.0,0.0,2.0,0.0,0.0,0.0,3.0,0.0,1.0,0.0,0.0,0.0,0.0,0.0,0.0,1.0,1.0,0.0,0.0,1.0,1.0,0.0,0.0,0.0,26.0,0.0,2.0,0.0,2.0,0.0,1.0,1.0,9.0,2.0,0.0,0.0,1.0,0.0,1.0,3.0,12.0,0.0,0.0,0.0,1.0,0.0,0.0,0.0,24.0,0.0,1.0,0.0,1.0,0.0,0.0,0.0,9.0,0.0,0.0,0.0,0.0,0.0,1.0,2.0,6.0,0.0,0.0,1.0,1.0,1.0,0.0,0.0,38.0,0.0,2.0,1.0,2.0,0.0,1.0,2.0,235.0,44.0,20.0,14.0,16.0,9.0,15.0,33.0,53.0,2.0,1.0,0.0,12.0,9.0,0.0,0.0,48.0,1.0,0.0,1.0,0.0,0.0,2.0,6.0,63.0,14.0,7.0,3.0,1.0,1.0,2.0,13.0,6.0,1.0,1.0,0.0,1.0,0.0,0.0,0.0,21.0,0.0,0.0,0.0,0.0,1.0,0.0,0.0,72.0,8.0,5.0,6.0,2.0,5.0,5.0,14.0,21.0,1.0,1.0,0.0,1.0,1.0,1.0,2.0,20.0,1.0,0.0,0.0,0.0,1.0,1.0,0.0,21.0,3.0,0.0,0.0,1.0,1.0,3.0,3.0,4.0,0.0,0.0,1.0,0.0,0.0,0.0,0.0,29.0,0.0,2.0,0.0,1.0,1.0,0.0,1.0,17.0,1.0,0.0,0.0,1.0,0.0,3.0,4.0,12.0,0.0,0.0,1.0,1.0,2.0,0.0,0.0,36.0,0.0,2.0,1.0,2.0,0.0,1.0,3.0,3.0,0.0,0.0,0.0,0.0,0.0,0.0,1.0,5.0,0.0,0.0,0.0,1.0,1.0,0.0,0.0,24.0,0.0,0.0,0.0,1.0,0.0,1.0,2.0,6.0,0.0,0.0,0.0,0.0,0.0,0.0,1.0,2.0,0.0,0.0,0.0,0.0,0.0,0.0,0.0,26.0,0.0,1.0,0.0,1.0,0.0,0.0,1.0,5.0,0.0,0.0,0.0,0.0,0.0,0.0,1.0,4.0,0.0,0.0,1.0,0.0,0.0,0.0,0.0,21.0,0.0,1.0,0.0,1.0,0.0,1.0,0.0,4.0,0.0,0.0,0.0,0.0,0.0,1.0,0.0,4.0,0.0,0.0,1.0,0.0,0.0,0.0,0.0,20.0,0.0,0.0,0.0,1.0,0.0,0.0,0.0,7.0,0.0,0.0,0.0,0.0,0.0,0.0,3.0,6.0,0.0,0.0,0.0,2.0,0.0,0.0,0.0,32.0,0.0,3.0,1.0,1.0,0.0,1.0,0.0,12.0,1.0,0.0,0.0,1.0,0.0,2.0,3.0,9.0,1.0,0.0,0.0,4.0,0.0,1.0,0.0,35.0,1.0,1.0,1.0,0.0,0.0,0.0,1.0,27.0,2.0,0.0,0.0,2.0,0.0,2.0,6.0,13.0,1.0,0.0,0.0,1.0,1.0,1.0,1.0,30.0,0.0,1.0,1.0,1.0,0.0,1.0,1.0,26.0,8.0,1.0,3.0,0.0,2.0,0.0,9.0,2.0,0.0,1.0,0.0,0.0,1.0,0.0,0.0,36.0,1.0,0.0,1.0,1.0,0.0,0.0,0.0,55.0,13.0,6.0,5.0,1.0,1.0,2.0,11.0,5.0,1.0,0.0,0.0,1.0,1.0,1.0,1.0,32.0,0.0,0.0,1.0,0.0,2.0,0.0,1.0,66.0,7.0,8.0,5.0,3.0,1.0,6.0,16.0,31.0,4.0,4.0,0.0,0.0,0.0,5.0,4.0,38.0,1.0,1.0,3.0,0.0,2.0,2.0,3.0,30.0,3.0,0.0,0.0,2.0,0.0,4.0,4.0,14.0,0.0,0.0,1.0,1.0,1.0,1.0,0.0,26.0,0.0,2.0,1.0,0.0,0.0,1.0,4.0,28.0,4.0,0.0,0.0,2.0,0.0,2.0,7.0,15.0,0.0,0.0,0.0,2.0,0.0,0.0,0.0,32.0,0.0,1.0,0.0,3.0,0.0,1.0,2.0,4.0,0.0,0.0,0.0,0.0,0.0,1.0,1.0,2.0,0.0,0.0,0.0,0.0,0.0,1.0,1.0,21.0,0.0,1.0,0.0,1.0,0.0,0.0,3.0,5.0,0.0,0.0,0.0,0.0,0.0,0.0,2.0,4.0,0.0,0.0,0.0,0.0,0.0,0.0,0.0,35.0,0.0,1.0,0.0,2.0,0.0,1.0,0.0,5.0,0.0,0.0,0.0,0.0,0.0,1.0,5.0,6.0,0.0,0.0,1.0,1.0,0.0,0.0,0.0,26.0,0.0,1.0,0.0,1.0,0.0,1.0,0.0,8.0,0.0,0.0,0.0,0.0,0.0,3.0,3.0,3.0,0.0,0.0,0.0,0.0,0.0,0.0,0.0,24.0,0.0,1.0,1.0,2.0,0.0,0.0,0.0,20.0,3.0,1.0,0.0,1.0,0.0,1.0,1.0,6.0,0.0,0.0,1.0,2.0,1.0,2.0,1.0,29.0,0.0,2.0,2.0,4.0,0.0,2.0,2.0,43.0,8.0,1.0,0.0,3.0,1.0,5.0,15.0,9.0,1.0,0.0,1.0,1.0,0.0,4.0,0.0,34.0,0.0,0.0,1.0,0.0,0.0,0.0,3.0,61.0,14.0,4.0,6.0,0.0,1.0,2.0,12.0,2.0,0.0,0.0,0.0,0.0,1.0,2.0,0.0,41.0,1.0,3.0,2.0,1.0,1.0,0.0,0.0,36.0,10.0,1.0,1.0,1.0,0.0,2.0,12.0,5.0,0.0,1.0,0.0,1.0,0.0,3.0,0.0,36.0,2.0,0.0,4.0,1.0,1.0,0.0,0.0,88.0,19.0,13.0,7.0,6.0,1.0,3.0,9.0,16.0,2.0,1.0,0.0,2.0,3.0,1.0,1.0,38.0,0.0,1.0,0.0,2.0,1.0,0.0,2.0,33.0,4.0,0.0,0.0,4.0,0.0,2.0,9.0,11.0,0.0,0.0,1.0,1.0,0.0,1.0,0.0,34.0,1.0,3.0,2.0,5.0,0.0,0.0,4.0,5.0,0.0,0.0,0.0,0.0,0.0,0.0,1.0,2.0,0.0,0.0,0.0,0.0,0.0,0.0,0.0,22.0,0.0,2.0,0.0,1.0,0.0,2.0,0.0,11.0,0.0,0.0,0.0,1.0,0.0,0.0,2.0,8.0,0.0,0.0,1.0,1.0,0.0,0.0,0.0,24.0,0.0,0.0,0.0,3.0,0.0,0.0,0.0,11.0,1.0,0.0,0.0,1.0,0.0,2.0,3.0,7.0,0.0,0.0,0.0,3.0,0.0,0.0,0.0,27.0,0.0,1.0,0.0,0.0,0.0,0.0,1.0,26.0,3.0,0.0,0.0,3.0,0.0,2.0,1.0,5.0,0.0,0.0,0.0,2.0,1.0,1.0,1.0,40.0,0.0,0.0,0.0,2.0,1.0,1.0,2.0,20.0,4.0,1.0,0.0,1.0,0.0,4.0,5.0,5.0,0.0,0.0,0.0,0.0,0.0,1.0,0.0,25.0,0.0,1.0,1.0,2.0,1.0,1.0,1.0,83.0,30.0,10.0,10.0,2.0,3.0,4.0,19.0,11.0,0.0,0.0,0.0,2.0,0.0,0.0,1.0,34.0,0.0,0.0,1.0,0.0,2.0,0.0,0.0,44.0,10.0,2.0,4.0,0.0,0.0,1.0,8.0,13.0,1.0,0.0,0.0,1.0,0.0,1.0,3.0,36.0,1.0,1.0,2.0,2.0,1.0,0.0,1.0,36.0,8.0,3.0,4.0,1.0,0.0,0.0,8.0,8.0,1.0,1.0,0.0,1.0,1.0,1.0,1.0,35.0,2.0,0.0,2.0,2.0,1.0,0.0,2.0,34.0,7.0,1.0,1.0,0.0,0.0,5.0,10.0,7.0,1.0,2.0,0.0,0.0,0.0,0.0,0.0,30.0,0.0,2.0,0.0,1.0,0.0,0.0,1.0,5.0,0.0,0.0,0.0,0.0,0.0,0.0,0.0,3.0,0.0,0.0,2.0,1.0,0.0,0.0,0.0,22.0,0.0,0.0,0.0,0.0,1.0,0.0,0.0,6.0,0.0,0.0,0.0,0.0,0.0,0.0,1.0,1.0,0.0,0.0,1.0,0.0,0.0,0.0,0.0,32.0,0.0,0.0,0.0,1.0,0.0,0.0,0.0,7.0,0.0,0.0,0.0,0.0,0.0,0.0,1.0,3.0,0.0,0.0,1.0,4.0,0.0,0.0,0.0,27.0,0.0,3.0,0.0,1.0,0.0,0.0,2.0,6.0,0.0,0.0,0.0,0.0,0.0,0.0,3.0,9.0,0.0,0.0,0.0,1.0,0.0,0.0,0.0,30.0,0.0,1.0,0.0,0.0,0.0,0.0,2.0,4.0,0.0,0.0,0.0,0.0,0.0,0.0,2.0,7.0,0.0,0.0,0.0,2.0,0.0,0.0,0.0,27.0,0.0,1.0,0.0,1.0,0.0,1.0,1.0,10.0,0.0,0.0,0.0,0.0,0.0,2.0,7.0,6.0,0.0,0.0,0.0,2.0,0.0,0.0,0.0,30.0,0.0,1.0,2.0,0.0,0.0,1.0,3.0,20.0,6.0,1.0,0.0,3.0,0.0,3.0,7.0,7.0,0.0,0.0,1.0,2.0,0.0,0.0,0.0,29.0,1.0,0.0,2.0,2.0,1.0,0.0,1.0,70.0,13.0,8.0,6.0,5.0,1.0,8.0,17.0,26.0,1.0,5.0,0.0,1.0,2.0,3.0,2.0,28.0,0.0,0.0,1.0,1.0,1.0,0.0,4.0,167.0,35.0,17.0,12.0,9.0,3.0,12.0,20.0,32.0,1.0,4.0,0.0,5.0,2.0,3.0,5.0,35.0,0.0,0.0,0.0,1.0,0.0,3.0,3.0,38.0,4.0,1.0,1.0,0.0,1.0,5.0,14.0,13.0,2.0,0.0,0.0,0.0,0.0,3.0,0.0,41.0,1.0,3.0,1.0,0.0,0.0,0.0,1.0,13.0,2.0,0.0,0.0,1.0,0.0,2.0,2.0,7.0,0.0,0.0,0.0,0.0,0.0,0.0,1.0,26.0,0.0,1.0,0.0,1.0,0.0,0.0,1.0,15.0,1.0,0.0,0.0,0.0,0.0,1.0,4.0,8.0,0.0,0.0,0.0,1.0,1.0,1.0,0.0,24.0,0.0,1.0,0.0,2.0,0.0,0.0,2.0,13.0,1.0,0.0,0.0,0.0,1.0,4.0,1.0,9.0,0.0,0.0,1.0,1.0,0.0,0.0,0.0,26.0,0.0,2.0,0.0,2.0,0.0,1.0,3.0,7.0,0.0,0.0,0.0,0.0,0.0,0.0,3.0,3.0,0.0,0.0,0.0,0.0,0.0,0.0,0.0,31.0,0.0,0.0,0.0,1.0,0.0,2.0,0.0,4.0,0.0,0.0,0.0,0.0,0.0,0.0,1.0,2.0,0.0,0.0,0.0,0.0,0.0,0.0,0.0,23.0,0.0,1.0,0.0,0.0,0.0,1.0,0.0,5.0,0.0,0.0,0.0,0.0,0.0,0.0,1.0,8.0,0.0,0.0,1.0,1.0,0.0,0.0,1.0,42.0,0.0,1.0,1.0,1.0,0.0,0.0,0.0,11.0,0.0,0.0,0.0,0.0,0.0,0.0,3.0,10.0,0.0,0.0,0.0,2.0,1.0,0.0,0.0,31.0,0.0,1.0,0.0,1.0,0.0,0.0,2.0,45.0,9.0,1.0,0.0,0.0,2.0,4.0,12.0,10.0,1.0,2.0,0.0,0.0,0.0,1.0,2.0,23.0,0.0,2.0,1.0,2.0,0.0,0.0,0.0,98.0,27.0,8.0,8.0,2.0,0.0,4.0,10.0,11.0,1.0,2.0,1.0,2.0,1.0,1.0,0.0,26.0,1.0,0.0,0.0,1.0,0.0,0.0,2.0,65.0,14.0,6.0,7.0,3.0,3.0,4.0,13.0,11.0,1.0,1.0,0.0,0.0,0.0,2.0,2.0,58.0,0.0,1.0,0.0,0.0,2.0,1.0,1.0,83.0,11.0,1.0,3.0,10.0,0.0,8.0,27.0,31.0,1.0,1.0,0.0,5.0,0.0,2.0,4.0,46.0,0.0,5.0,1.0,2.0,1.0,1.0,5.0,18.0,0.0,0.0,0.0,0.0,0.0,3.0,2.0,6.0,1.0,0.0,1.0,2.0,0.0,0.0,1.0,31.0,0.0,0.0,0.0,1.0,0.0,1.0,1.0,7.0,0.0,0.0,0.0,0.0,0.0,0.0,1.0,8.0,0.0,0.0,2.0,2.0,0.0,0.0,1.0,37.0,0.0,5.0,0.0,4.0,0.0,0.0,1.0,5.0,0.0,0.0,0.0,0.0,0.0,0.0,2.0,3.0,0.0,0.0,1.0,0.0,0.0,0.0,0.0,28.0,0.0,1.0,0.0,2.0,0.0,1.0,0.0,5.0,1.0,0.0,0.0,1.0,0.0,0.0,1.0,1.0,0.0,0.0,0.0,0.0,0.0,0.0,0.0,25.0,0.0,1.0,0.0,0.0,0.0,0.0,0.0,2.0,0.0,0.0,0.0,1.0,0.0,0.0,1.0,7.0,0.0,0.0,0.0,2.0,0.0,0.0,0.0,26.0,0.0,1.0,0.0,1.0,0.0,1.0,1.0,5.0,0.0,0.0,0.0,0.0,0.0,1.0,0.0,2.0,1.0,0.0,0.0,1.0,0.0,0.0,0.0,33.0,0.0,3.0,0.0,1.0,0.0,1.0,0.0,21.0,2.0,0.0,0.0,2.0,0.0,2.0,6.0,6.0,0.0,0.0,0.0,2.0,0.0,1.0,0.0,32.0,0.0,0.0,0.0,0.0,1.0,1.0,2.0,16.0,1.0,1.0,0.0,1.0,0.0,2.0,4.0,10.0,0.0,0.0,1.0,1.0,1.0,1.0,1.0,33.0,0.0,1.0,1.0,1.0,2.0,0.0,1.0,68.0,11.0,6.0,5.0,4.0,1.0,7.0,16.0,12.0,0.0,0.0,0.0,2.0,0.0,5.0,1.0,29.0,0.0,1.0,2.0,1.0,1.0,1.0,1.0,54.0,12.0,7.0,7.0,0.0,1.0,1.0,8.0,7.0,0.0,1.0,0.0,0.0,0.0,0.0,0.0,20.0,1.0,0.0,0.0,1.0,2.0,1.0,1.0,48.0,9.0,2.0,2.0,1.0,3.0,4.0,14.0,17.0,2.0,2.0,0.0,1.0,1.0,3.0,2.0,22.0,1.0,1.0,1.0,0.0,0.0,1.0,1.0,27.0,4.0,0.0,1.0,2.0,0.0,5.0,4.0,12.0,0.0,0.0,1.0,2.0,0.0,1.0,1.0,30.0,0.0,1.0,0.0,0.0,1.0,1.0,1.0,15.0,0.0,0.0,0.0,1.0,0.0,2.0,5.0,7.0,0.0,0.0,2.0,1.0,1.0,0.0,0.0,26.0,0.0,2.0,0.0,0.0,0.0,1.0,2.0,8.0,0.0,0.0,0.0,0.0,0.0,1.0,2.0,4.0,0.0,0.0,1.0,1.0,0.0,0.0,0.0,32.0,0.0,0.0,0.0,2.0,0.0,0.0,0.0,3.0,0.0,0.0,0.0,0.0,0.0,0.0,0.0,2.0,0.0,0.0,1.0,0.0,0.0,0.0,0.0,26.0,0.0,1.0,0.0,0.0,0.0,2.0,4.0,3.0,0.0,0.0,0.0,0.0,0.0,0.0,2.0,5.0,0.0,0.0,0.0,0.0,0.0,0.0,0.0,30.0,0.0,1.0,0.0,1.0,0.0,1.0,0.0,7.0,1.0,0.0,0.0,0.0,0.0,0.0,2.0,7.0,0.0,0.0,0.0,2.0,0.0,0.0,0.0,30.0,0.0,1.0,0.0,2.0,0.0,2.0,1.0,13.0,0.0,0.0,0.0,0.0,0.0,1.0,8.0,5.0,0.0,0.0,0.0,2.0,0.0,0.0,0.0,41.0,0.0,0.0,1.0,0.0,0.0,1.0,3.0,28.0,2.0,0.0,0.0,2.0,0.0,7.0,5.0,7.0,0.0,0.0,1.0,1.0,0.0,0.0,1.0,39.0,0.0,1.0,1.0,3.0,0.0,1.0,1.0,34.0,4.0,1.0,0.0,3.0,0.0,3.0,9.0,11.0,0.0,0.0,0.0,1.0,0.0,2.0,1.0,27.0,0.0,0.0,1.0,3.0,0.0,0.0,2.0,107.0,17.0,6.0,8.0,5.0,3.0,7.0,22.0,31.0,1.0,1.0,0.0,3.0,3.0,5.0,4.0,42.0,0.0,1.0,2.0,0.0,1.0,1.0,4.0,41.0,11.0,2.0,3.0,0.0,0.0,2.0,14.0,8.0,1.0,1.0,0.0,0.0,0.0,1.0,0.0,34.0,1.0,0.0,2.0,0.0,1.0,0.0,1.0,91.0,25.0,10.0,8.0,0.0,1.0,4.0,13.0,13.0,0.0,0.0,1.0,0.0,0.0,0.0,1.0,44.0,0.0,0.0,1.0,2.0,1.0,0.0,0.0,27.0,7.0,0.0,0.0,1.0,0.0,3.0,8.0,8.0,0.0,0.0,2.0,1.0,0.0,0.0,0.0,29.0,0.0,1.0,0.0,0.0,0.0,1.0,0.0,9.0,0.0,0.0,0.0,0.0,0.0,2.0,0.0,6.0,0.0,0.0,1.0,0.0,0.0,0.0,0.0,28.0,0.0,0.0,1.0,0.0,0.0,0.0,0.0,8.0,0.0,0.0,0.0,0.0,0.0,0.0,2.0,7.0,0.0,0.0,0.0,1.0,0.0,0.0,0.0,34.0,0.0,2.0,0.0,0.0,0.0,1.0,1.0,2.0,0.0,0.0,0.0,0.0,0.0,0.0,2.0,2.0,0.0,0.0,0.0,0.0,0.0,0.0,0.0,27.0,0.0,1.0,0.0,1.0,0.0,0.0,0.0,6.0,0.0,0.0,0.0,0.0,0.0,0.0,1.0,3.0,0.0,0.0,0.0,0.0,0.0,0.0,0.0,39.0,0.0,1.0,0.0,3.0,0.0,0.0,0.0,7.0,0.0,0.0,0.0,2.0,0.0,0.0,2.0,5.0,0.0,0.0,1.0,1.0,0.0,0.0,2.0,33.0,1.0,3.0,1.0,2.0,0.0,0.0,1.0,10.0,0.0,0.0,0.0,0.0,0.0,1.0,3.0,8.0,0.0,0.0,0.0,1.0,0.0,0.0,0.0,33.0,0.0,1.0,1.0,2.0,0.0,0.0,1.0,22.0,3.0,1.0,0.0,2.0,0.0,3.0,3.0,8.0,0.0,0.0,0.0,0.0,0.0,0.0,1.0,38.0,0.0,2.0,0.0,1.0,0.0,0.0,0.0,38.0,4.0,1.0,2.0,4.0,1.0,3.0,7.0,11.0,1.0,0.0,0.0,1.0,0.0,0.0,0.0,36.0,0.0,1.0,1.0,0.0,0.0,0.0,1.0,175.0,37.0,18.0,14.0,13.0,6.0,12.0,21.0,34.0,2.0,3.0,0.0,5.0,5.0,1.0,2.0,41.0,0.0,0.0,0.0,3.0,0.0,0.0,5.0,49.0,12.0,2.0,3.0,0.0,1.0,3.0,11.0,7.0,0.0,1.0,0.0,1.0,1.0,6.0,1.0,31.0,1.0,0.0,2.0,1.0,0.0,0.0,1.0,93.0,25.0,10.0,8.0,1.0,3.0,2.0,14.0,11.0,0.0,0.0,0.0,2.0,1.0,0.0,0.0,31.0,1.0,0.0,1.0,0.0,0.0,0.0,1.0,20.0,4.0,1.0,1.0,0.0,2.0,4.0,3.0,10.0,1.0,0.0,1.0,2.0,0.0,0.0,1.0,28.0,0.0,1.0,0.0,2.0,1.0,1.0,1.0,11.0,0.0,0.0,0.0,1.0,0.0,1.0,3.0,7.0,0.0,0.0,1.0,2.0,0.0,0.0,0.0,19.0,0.0,1.0,0.0,2.0,0.0,1.0,1.0,5.0,0.0,0.0,0.0,0.0,0.0,0.0,0.0,10.0,0.0,0.0,1.0,0.0,0.0,0.0,0.0,27.0,0.0,1.0,0.0,2.0,1.0,1.0,2.0,6.0,0.0,0.0,0.0,0.0,0.0,0.0,3.0,3.0,0.0,0.0,0.0,0.0,0.0,0.0,0.0,28.0,0.0,0.0,1.0,1.0,0.0,1.0,0.0,5.0,0.0,0.0,0.0,0.0,0.0,0.0,1.0,4.0,0.0,0.0,0.0,0.0,0.0,0.0,0.0,25.0,0.0,2.0,0.0,4.0,0.0,1.0,0.0,3.0,0.0,0.0,0.0,0.0,0.0,1.0,1.0,11.0,0.0,0.0,1.0,1.0,0.0,1.0,0.0,36.0,0.0,3.0,0.0,1.0,0.0,0.0,1.0,27.0,0.0,0.0,0.0,2.0,0.0,2.0,3.0,16.0,0.0,0.0,0.0,2.0,0.0,0.0,0.0,34.0,0.0,2.0,1.0,1.0,0.0,0.0,3.0,18.0,2.0,0.0,0.0,0.0,0.0,3.0,6.0,13.0,0.0,0.0,1.0,4.0,0.0,0.0,0.0,35.0,0.0,1.0,0.0,2.0,0.0,0.0,2.0,58.0,20.0,6.0,4.0,3.0,1.0,4.0,7.0,10.0,2.0,1.0,0.0,0.0,0.0,1.0,1.0,44.0,1.0,0.0,0.0,0.0,1.0,0.0,1.0,46.0,11.0,2.0,5.0,0.0,0.0,2.0,10.0,12.0,1.0,2.0,1.0,0.0,0.0,1.0,2.0,42.0,3.0,0.0,1.0,3.0,1.0,1.0,1.0,43.0,6.0,2.0,1.0,3.0,1.0,3.0,9.0,19.0,0.0,0.0,0.0,1.0,1.0,2.0,2.0,40.0,0.0,1.0,0.0,2.0,0.0,1.0,0.0,92.0,23.0,7.0,5.0,7.0,1.0,10.0,15.0,26.0,0.0,0.0,1.0,4.0,4.0,1.0,2.0,40.0,0.0,0.0,0.0,0.0,1.0,0.0,4.0,8.0,0.0,0.0,0.0,0.0,0.0,1.0,3.0,8.0,0.0,0.0,0.0,1.0,0.0,0.0,1.0,28.0,0.0,2.0,0.0,1.0,0.0,0.0,2.0,8.0,1.0,0.0,0.0,1.0,0.0,0.0,3.0,5.0,0.0,0.0,1.0,0.0,0.0,0.0,0.0,27.0,0.0,1.0,0.0,2.0,0.0,0.0,3.0,7.0,0.0,0.0,0.0,0.0,0.0,0.0,3.0,5.0,0.0,0.0,0.0,0.0,0.0,0.0,0.0,31.0,0.0,2.0,0.0,2.0,0.0,0.0,1.0,10.0,0.0,0.0,0.0,0.0,0.0,1.0,1.0,9.0,1.0,0.0,1.0,0.0,0.0,0.0,0.0,35.0,0.0,1.0,1.0,2.0,0.0,1.0,0.0,17.0,2.0,0.0,0.0,1.0,0.0,1.0,2.0,11.0,0.0,0.0,0.0,2.0,0.0,0.0,0.0,39.0,0.0,2.0,0.0,1.0,0.0,1.0,3.0,8.0,0.0,0.0,0.0,0.0,0.0,3.0,5.0,8.0,0.0,0.0,0.0,2.0,0.0,0.0,0.0,38.0,0.0,1.0,1.0,1.0,0.0,1.0,1.0,29.0,2.0,2.0,0.0,3.0,0.0,3.0,3.0,9.0,0.0,0.0,0.0,1.0,0.0,1.0,1.0,29.0,0.0,1.0,1.0,0.0,0.0,0.0,0.0,27.0,2.0,0.0,1.0,1.0,1.0,2.0,6.0,6.0,0.0,0.0,1.0,0.0,0.0,2.0,0.0,32.0,1.0,1.0,0.0,0.0,1.0,0.0,1.0,191.0,41.0,20.0,14.0,14.0,9.0,13.0,29.0,39.0,2.0,3.0,0.0,5.0,5.0,2.0,1.0,57.0,0.0,1.0,1.0,0.0,0.0,1.0,3.0,20.0,4.0,1.0,0.0,0.0,0.0,0.0,8.0,8.0,0.0,0.0,0.0,0.0,0.0,0.0,0.0,40.0,0.0,0.0,0.0,1.0,1.0,0.0,0.0,40.0,7.0,0.0,0.0,1.0,2.0,4.0,6.0,19.0,0.0,0.0,2.0,1.0,1.0,0.0,2.0,43.0,0.0,2.0,1.0,3.0,0.0,0.0,3.0,19.0,2.0,0.0,0.0,1.0,0.0,3.0,5.0,13.0,0.0,0.0,2.0,2.0,1.0,0.0,1.0,23.0,0.0,1.0,0.0,2.0,0.0,1.0,4.0,14.0,0.0,0.0,0.0,1.0,0.0,3.0,2.0,11.0,0.0,0.0,0.0,2.0,1.0,0.0,0.0,38.0,0.0,2.0,0.0,1.0,0.0,2.0,4.0,4.0,0.0,0.0,0.0,0.0,0.0,0.0,2.0,3.0,0.0,0.0,1.0,0.0,0.0,0.0,0.0,33.0,0.0,2.0,0.0,2.0,0.0,0.0,0.0,7.0,0.0,0.0,0.0,0.0,0.0,0.0,3.0,7.0,0.0,0.0,1.0,0.0,0.0,0.0,1.0,38.0,0.0,2.0,0.0,1.0,1.0,0.0,1.0,20.0,1.0,0.0,0.0,1.0,0.0,1.0,2.0,18.0,0.0,0.0,2.0,2.0,0.0,0.0,1.0,40.0,0.0,1.0,2.0,4.0,0.0,1.0,0.0,14.0,0.0,0.0,0.0,2.0,0.0,2.0,3.0,13.0,1.0,0.0,1.0,2.0,0.0,0.0,2.0,30.0,0.0,2.0,1.0,2.0,0.0,1.0,6.0,23.0,1.0,0.0,0.0,4.0,0.0,3.0,5.0,13.0,0.0,0.0,0.0,1.0,1.0,0.0,1.0,35.0,0.0,2.0,1.0,3.0,1.0,0.0,1.0,90.0,9.0,4.0,4.0,6.0,0.0,4.0,15.0,27.0,1.0,1.0,0.0,2.0,0.0,2.0,4.0,41.0,0.0,1.0,1.0,1.0,2.0,2.0,5.0,237.0,43.0,20.0,14.0,16.0,10.0,18.0,33.0,46.0,1.0,2.0,0.0,5.0,11.0,0.0,2.0,60.0,0.0,2.0,2.0,3.0,2.0,2.0,5.0,111.0,33.0,14.0,13.0,2.0,5.0,4.0,18.0,10.0,2.0,1.0,0.0,0.0,2.0,1.0,1.0,46.0,0.0,0.0,1.0,1.0,1.0,0.0,1.0,56.0,17.0,8.0,9.0,0.0,2.0,3.0,10.0,14.0,0.0,2.0,0.0,1.0,1.0,1.0,3.0,48.0,0.0,0.0,0.0,1.0,1.0,0.0,1.0,28.0,6.0,0.0,1.0,3.0,0.0,4.0,5.0,18.0,1.0,0.0,1.0,1.0,0.0,1.0,2.0,26.0,0.0,1.0,0.0,3.0,1.0,1.0,1.0,11.0,0.0,0.0,0.0,0.0,0.0,0.0,3.0,14.0,1.0,0.0,0.0,0.0,0.0,0.0,0.0,33.0,0.0,2.0,1.0,1.0,2.0,1.0,3.0,4.0,0.0,0.0,0.0,1.0,0.0,0.0,0.0,4.0,0.0,0.0,0.0,0.0,1.0,0.0,0.0,38.0,0.0,0.0,0.0,1.0,0.0,1.0,3.0,3.0,0.0,0.0,0.0,0.0,0.0,0.0,0.0,2.0,0.0,0.0,0.0,0.0,0.0,0.0,0.0,34.0,0.0,0.0,0.0,1.0,0.0,2.0,0.0,4.0,0.0,0.0,0.0,0.0,0.0,0.0,2.0,4.0,0.0,0.0,0.0,0.0,0.0,0.0,0.0,40.0,0.0,0.0,0.0,1.0,0.0,0.0,0.0,4.0,0.0,0.0,0.0,0.0,0.0,0.0,1.0,10.0,0.0,0.0,0.0,1.0,0.0,0.0,0.0,31.0,0.0,2.0,0.0,3.0,0.0,1.0,0.0),.Dim=c(274,24)))

#initial value for chain 2

list(a=c(-2.65,-3.471,-5.098,-6.338,-2.761,-4.259,-2.611,-2.083,-2.951,-4.222,-5.477,-2.893,-2.488,-3.728,-3.876,-3.35,-2.038,-4.188,-2.811,-2.364,-1.682,-3.051,-2.339,-1.774),

b=structure(.Data=c(0.1216,0.2276,0.3482,0.4647,0.04957,0.2064,0.07824,0.09823,0.03412,0.1987,0.316,-0.06069,-0.0141,0.04476,0.1753,0.1077,0.01258,0.2075,-0.06274,0.08664,3.541E-4,0.1211,-0.07716,0.003957,0.008313,0.01503,0.01225,0.01765,0.01038,0.009035,0.007097,0.007452,0.005342,0.00447,0.002952,0.004729,0.009116,0.009607,-0.002049,0.006524,0.001059,-0.003893,0.003216,0.003482,0.003338,-0.002737,0.006485,0.00492,-0.005612,-0.01338,-0.01013,-0.01351,-0.00412,-0.00428,-1.48E-4,-0.002197,-1.74E-4,9.652E-4,0.007629,-0.01267,-0.00409,-8.665E-4,0.01268,0.005548,-8.218E-4,0.00599,8.167E-4,-0.004441,-0.002774,-0.004756,4.182E-4,-0.003205,0.01982,0.03473,0.0461,0.1296,-0.01124,0.03718,0.05558,0.02086,0.03058,0.07734,-0.009829,0.03203,0.008808,0.01136,0.08892,0.05265,0.03654,7.033E-5,0.05313,0.02707,0.05728,-0.01463,0.02624,0.07393),.Dim=c(4,24)),

d=c(0.05138,0.4834,0.4026,0.3764,0.2259,0.9116,0.2465,0.531,0.4521,0.3291,0.2294,0.6054,0.1778,0.01612,0.3625,0.4507,0.5409,0.4676,0.2442,0.3611,0.1746,0.2477,0.1628,0.2353,0.3732,0.2426,0.2134,0.5719,0.5188,0.1672,0.5519,0.5154,0.4354,0.5671,0.6101,0.4046,0.4993,0.4783,0.1429,0.1824,0.6946,0.6793,0.8707,0.7041,0.2805,0.2702,0.7446,0.3102,0.2498,0.2607,0.1468,0.3278,0.3576,0.5844,0.3292,0.5293,0.2316,0.3334,0.5316,0.2002,0.6724,0.7934,0.43,0.5217,0.7042,0.9221,0.9679,0.6416,0.3304,0.4006,0.2285,0.8938,0.9838,0.4835,0.3147,0.9902,0.6103,0.0109,0.4568,0.3929,0.3682,0.8832,0.6236,0.6103,0.89,0.7318,0.2906,0.8566,0.5556,0.52,0.7097,0.9154,0.9465,0.8665,0.6108,0.5824,0.1549,0.514,0.863,0.4371,0.9827,0.2982,0.3082,0.3839,0.9598,0.5391,0.4015,0.5394,0.5144,0.3409,0.9211,0.5037,0.5816,0.9806,0.4627,0.1703,0.9789,0.9569,0.804,0.572,0.9562,0.03579,0.2963,0.9656,0.4675,0.8982,0.6091,0.3138,0.9519,0.6966,0.4943,0.4166,0.6654,0.3844,0.511,0.4273,0.3568,0.8361,0.6692,0.7689,0.3275,0.8567,0.1773,0.9323,0.8343,0.9325,0.9973,0.7492,0.7674,0.8041,0.3356,0.9266,0.7121,0.5395,0.678,0.2767,0.3556,0.7439,0.535,0.5804,0.6926,0.4595,0.3249,0.457,0.2628,0.3588,0.4758,0.7336,0.6812,0.4613,0.2151,0.3905,0.4039,0.5062,0.4093,0.2106,0.3555,0.6358,0.5142,0.4956,0.2725,0.3218,0.2784,0.5198,0.6134,0.6985,0.2722,0.4359,0.4966,0.6536,0.5752,0.4988,0.5492,0.6444,0.2712,0.5786,0.5351,0.64,0.232,0.5055,0.6663,0.4458,0.3795,0.4638,0.07821,0.1904,0.4129,0.4816,0.5778,0.7803,0.8128,0.7944,0.7398,0.4788,0.6203,0.2939,0.4217,0.3273,0.5682,0.2909,0.2157,0.5455,0.6532,0.2648,0.6208,0.2541,0.2793,0.4624,0.3656,0.2989,0.6098,0.4406,0.1286,0.6787,0.7774,0.9008,0.4282,0.411,0.1369,0.4523,0.2876,0.1815,0.3644,0.5296,0.6632,0.3928,0.7196,0.3999,0.9355,0.4922,0.3286,0.5428,0.5906,0.5869,0.3324,0.1798,0.6296,0.8223,0.9791,0.4606,0.4929,0.3239,0.3038,0.4695,0.2928,0.162,0.1875,0.2172,0.8546,0.4715,0.7071,0.4846,0.5201,0.382),

present=structure(.Data=c(14.0,2.0,1.0,0.0,1.0,0.0,0.0,4.0,4.0,1.0,0.0,0.0,1.0,0.0,0.0,0.0,21.0,0.0,1.0,1.0,0.0,2.0,0.0,0.0,25.0,1.0,0.0,0.0,1.0,0.0,1.0,7.0,7.0,0.0,0.0,0.0,1.0,0.0,0.0,0.0,18.0,0.0,2.0,1.0,2.0,0.0,0.0,0.0,26.0,1.0,0.0,0.0,0.0,0.0,1.0,5.0,3.0,1.0,0.0,1.0,0.0,0.0,1.0,1.0,21.0,0.0,0.0,0.0,1.0,0.0,0.0,0.0,125.0,25.0,8.0,4.0,10.0,1.0,8.0,27.0,27.0,1.0,1.0,0.0,9.0,2.0,0.0,3.0,20.0,1.0,0.0,1.0,2.0,0.0,0.0,1.0,65.0,15.0,5.0,2.0,2.0,2.0,2.0,9.0,7.0,0.0,0.0,0.0,1.0,2.0,0.0,0.0,20.0,1.0,0.0,0.0,0.0,0.0,1.0,0.0,21.0,5.0,0.0,0.0,0.0,0.0,3.0,6.0,5.0,0.0,0.0,1.0,1.0,0.0,0.0,1.0,13.0,0.0,0.0,1.0,1.0,0.0,0.0,0.0,10.0,0.0,0.0,0.0,0.0,0.0,0.0,1.0,1.0,0.0,0.0,0.0,0.0,0.0,0.0,0.0,15.0,0.0,0.0,2.0,1.0,0.0,0.0,0.0,11.0,0.0,0.0,0.0,2.0,0.0,0.0,0.0,4.0,0.0,0.0,1.0,0.0,1.0,0.0,0.0,12.0,0.0,0.0,0.0,0.0,0.0,0.0,2.0,5.0,0.0,0.0,0.0,1.0,0.0,0.0,1.0,1.0,0.0,0.0,1.0,0.0,0.0,0.0,0.0,8.0,0.0,1.0,0.0,0.0,0.0,1.0,1.0,2.0,0.0,0.0,0.0,0.0,0.0,1.0,1.0,0.0,0.0,0.0,0.0,1.0,0.0,0.0,0.0,12.0,0.0,0.0,0.0,0.0,0.0,1.0,1.0,2.0,0.0,0.0,0.0,0.0,0.0,0.0,1.0,5.0,0.0,0.0,0.0,2.0,0.0,0.0,0.0,19.0,0.0,1.0,0.0,0.0,0.0,1.0,0.0,9.0,0.0,0.0,0.0,0.0,0.0,1.0,3.0,3.0,0.0,0.0,0.0,2.0,0.0,0.0,0.0,17.0,0.0,1.0,0.0,1.0,0.0,1.0,0.0,21.0,4.0,0.0,0.0,2.0,0.0,3.0,5.0,4.0,0.0,0.0,0.0,3.0,0.0,0.0,0.0,13.0,0.0,2.0,1.0,1.0,1.0,0.0,1.0,16.0,5.0,0.0,0.0,0.0,0.0,0.0,6.0,4.0,0.0,0.0,0.0,0.0,0.0,0.0,0.0,12.0,0.0,0.0,0.0,0.0,1.0,0.0,0.0,107.0,26.0,7.0,4.0,8.0,2.0,7.0,17.0,10.0,0.0,0.0,0.0,3.0,2.0,0.0,0.0,25.0,0.0,0.0,0.0,1.0,0.0,1.0,0.0,55.0,16.0,2.0,2.0,2.0,3.0,4.0,12.0,7.0,1.0,2.0,0.0,0.0,0.0,0.0,1.0,21.0,1.0,0.0,0.0,0.0,0.0,0.0,1.0,57.0,15.0,2.0,1.0,2.0,1.0,1.0,8.0,13.0,0.0,1.0,1.0,1.0,0.0,1.0,2.0,12.0,0.0,0.0,0.0,0.0,0.0,1.0,1.0,21.0,4.0,0.0,0.0,1.0,0.0,1.0,4.0,8.0,0.0,0.0,0.0,1.0,1.0,0.0,0.0,15.0,1.0,0.0,1.0,2.0,0.0,0.0,0.0,17.0,1.0,0.0,0.0,1.0,0.0,1.0,5.0,6.0,0.0,0.0,0.0,1.0,1.0,0.0,0.0,22.0,0.0,0.0,0.0,0.0,0.0,0.0,1.0,6.0,0.0,0.0,0.0,3.0,0.0,0.0,0.0,0.0,0.0,0.0,0.0,1.0,0.0,0.0,0.0,14.0,0.0,0.0,0.0,0.0,0.0,0.0,0.0,3.0,0.0,0.0,0.0,0.0,0.0,0.0,0.0,2.0,0.0,0.0,1.0,2.0,0.0,0.0,0.0,15.0,0.0,0.0,0.0,0.0,0.0,0.0,1.0,2.0,0.0,0.0,0.0,2.0,0.0,0.0,0.0,0.0,0.0,0.0,2.0,1.0,0.0,0.0,0.0,10.0,0.0,1.0,0.0,1.0,0.0,0.0,0.0,4.0,0.0,0.0,0.0,0.0,0.0,0.0,2.0,3.0,0.0,0.0,1.0,4.0,0.0,0.0,0.0,13.0,0.0,0.0,0.0,0.0,0.0,1.0,0.0,9.0,0.0,0.0,0.0,0.0,0.0,1.0,4.0,2.0,0.0,0.0,0.0,2.0,1.0,0.0,0.0,18.0,0.0,0.0,0.0,0.0,0.0,0.0,0.0,11.0,1.0,0.0,0.0,1.0,0.0,1.0,1.0,8.0,0.0,0.0,0.0,0.0,0.0,0.0,0.0,15.0,0.0,2.0,2.0,0.0,0.0,0.0,1.0,19.0,1.0,0.0,0.0,3.0,0.0,1.0,3.0,6.0,0.0,1.0,1.0,0.0,0.0,0.0,1.0,17.0,0.0,0.0,1.0,0.0,0.0,1.0,1.0,131.0,32.0,6.0,5.0,4.0,3.0,5.0,25.0,15.0,1.0,1.0,1.0,5.0,2.0,1.0,0.0,23.0,0.0,0.0,0.0,0.0,1.0,1.0,2.0,70.0,20.0,6.0,2.0,1.0,0.0,2.0,17.0,6.0,0.0,1.0,0.0,1.0,0.0,0.0,0.0,18.0,0.0,0.0,0.0,1.0,0.0,0.0,0.0,47.0,8.0,5.0,1.0,3.0,0.0,1.0,9.0,6.0,0.0,1.0,1.0,0.0,0.0,1.0,2.0,14.0,0.0,0.0,0.0,0.0,0.0,0.0,0.0,31.0,6.0,0.0,0.0,1.0,0.0,2.0,6.0,8.0,0.0,0.0,0.0,2.0,0.0,0.0,0.0,18.0,0.0,0.0,0.0,0.0,1.0,1.0,0.0,5.0,0.0,0.0,0.0,1.0,0.0,0.0,0.0,4.0,0.0,0.0,0.0,1.0,0.0,0.0,0.0,14.0,0.0,0.0,0.0,0.0,0.0,0.0,1.0,1.0,0.0,0.0,0.0,0.0,0.0,0.0,1.0,1.0,0.0,0.0,0.0,0.0,0.0,0.0,0.0,8.0,0.0,0.0,0.0,1.0,0.0,0.0,0.0,1.0,0.0,0.0,0.0,0.0,0.0,0.0,0.0,2.0,0.0,0.0,2.0,0.0,0.0,0.0,0.0,20.0,0.0,0.0,1.0,0.0,0.0,0.0,0.0,7.0,0.0,0.0,0.0,0.0,0.0,0.0,1.0,1.0,0.0,0.0,0.0,0.0,0.0,0.0,0.0,14.0,0.0,1.0,0.0,0.0,0.0,1.0,0.0,4.0,0.0,0.0,0.0,0.0,0.0,0.0,1.0,3.0,0.0,0.0,1.0,4.0,1.0,0.0,0.0,16.0,0.0,2.0,0.0,1.0,0.0,0.0,0.0,14.0,1.0,0.0,0.0,0.0,0.0,1.0,4.0,6.0,0.0,0.0,0.0,2.0,0.0,0.0,0.0,25.0,0.0,2.0,0.0,1.0,1.0,1.0,0.0,5.0,0.0,0.0,0.0,0.0,0.0,2.0,1.0,3.0,0.0,1.0,1.0,1.0,0.0,0.0,0.0,14.0,0.0,0.0,0.0,1.0,2.0,1.0,0.0,44.0,6.0,1.0,1.0,5.0,0.0,6.0,6.0,16.0,0.0,0.0,0.0,1.0,1.0,2.0,3.0,16.0,1.0,1.0,1.0,0.0,1.0,2.0,1.0,166.0,41.0,13.0,11.0,9.0,3.0,10.0,27.0,21.0,1.0,2.0,1.0,1.0,2.0,0.0,0.0,31.0,1.0,1.0,2.0,2.0,1.0,0.0,2.0,52.0,6.0,1.0,0.0,2.0,1.0,1.0,11.0,3.0,0.0,1.0,0.0,0.0,0.0,0.0,0.0,22.0,0.0,1.0,2.0,2.0,2.0,0.0,0.0,42.0,8.0,3.0,1.0,1.0,0.0,1.0,7.0,10.0,0.0,1.0,1.0,0.0,0.0,0.0,2.0,14.0,0.0,0.0,0.0,0.0,0.0,0.0,0.0,44.0,12.0,4.0,0.0,3.0,0.0,2.0,8.0,9.0,0.0,0.0,0.0,1.0,1.0,0.0,0.0,12.0,0.0,0.0,0.0,1.0,0.0,0.0,0.0,8.0,0.0,0.0,0.0,1.0,0.0,1.0,1.0,2.0,0.0,0.0,0.0,0.0,0.0,0.0,0.0,19.0,1.0,0.0,0.0,0.0,0.0,1.0,0.0,3.0,0.0,0.0,0.0,0.0,0.0,0.0,1.0,2.0,0.0,0.0,0.0,0.0,0.0,0.0,0.0,12.0,0.0,0.0,0.0,0.0,0.0,0.0,0.0,3.0,0.0,0.0,0.0,0.0,0.0,0.0,3.0,2.0,0.0,0.0,0.0,0.0,0.0,0.0,0.0,18.0,0.0,1.0,0.0,0.0,0.0,0.0,0.0,5.0,0.0,0.0,0.0,0.0,0.0,0.0,3.0,2.0,0.0,0.0,0.0,0.0,0.0,0.0,0.0,20.0,0.0,0.0,0.0,0.0,0.0,0.0,0.0,3.0,0.0,0.0,0.0,0.0,0.0,0.0,1.0,1.0,0.0,0.0,0.0,0.0,0.0,0.0,0.0,13.0,0.0,0.0,0.0,0.0,0.0,0.0,0.0,10.0,0.0,0.0,0.0,3.0,0.0,0.0,5.0,2.0,0.0,0.0,1.0,0.0,0.0,0.0,0.0,17.0,0.0,2.0,0.0,0.0,0.0,0.0,1.0,22.0,0.0,0.0,0.0,1.0,0.0,4.0,4.0,7.0,0.0,0.0,0.0,2.0,1.0,0.0,2.0,23.0,0.0,0.0,1.0,1.0,0.0,2.0,0.0,163.0,31.0,12.0,7.0,11.0,3.0,5.0,25.0,31.0,1.0,2.0,1.0,4.0,4.0,1.0,0.0,33.0,0.0,0.0,1.0,1.0,0.0,3.0,3.0,91.0,31.0,6.0,5.0,1.0,1.0,7.0,17.0,13.0,0.0,1.0,0.0,0.0,0.0,0.0,0.0,24.0,1.0,0.0,0.0,2.0,2.0,1.0,0.0,30.0,2.0,1.0,1.0,1.0,1.0,0.0,2.0,2.0,0.0,1.0,0.0,0.0,0.0,1.0,0.0,31.0,0.0,1.0,0.0,0.0,1.0,0.0,0.0,24.0,5.0,0.0,0.0,1.0,0.0,2.0,7.0,3.0,0.0,1.0,1.0,1.0,2.0,0.0,1.0,21.0,0.0,0.0,0.0,0.0,0.0,0.0,0.0,6.0,1.0,0.0,0.0,0.0,0.0,0.0,3.0,3.0,0.0,0.0,0.0,0.0,0.0,0.0,0.0,12.0,0.0,0.0,0.0,0.0,0.0,0.0,0.0,5.0,0.0,0.0,0.0,1.0,0.0,1.0,0.0,3.0,0.0,0.0,1.0,1.0,0.0,0.0,0.0,15.0,0.0,0.0,0.0,0.0,0.0,0.0,1.0,3.0,0.0,0.0,0.0,1.0,0.0,0.0,1.0,2.0,0.0,0.0,0.0,1.0,1.0,0.0,0.0,12.0,0.0,0.0,0.0,0.0,0.0,0.0,0.0,3.0,0.0,0.0,0.0,0.0,0.0,0.0,1.0,0.0,0.0,0.0,0.0,1.0,1.0,0.0,0.0,17.0,0.0,1.0,0.0,1.0,0.0,0.0,0.0,5.0,0.0,0.0,0.0,1.0,0.0,1.0,2.0,3.0,0.0,0.0,1.0,1.0,0.0,0.0,0.0,18.0,0.0,1.0,0.0,1.0,0.0,1.0,0.0,8.0,0.0,0.0,0.0,0.0,0.0,0.0,2.0,0.0,0.0,0.0,0.0,0.0,0.0,0.0,0.0,16.0,1.0,0.0,0.0,1.0,0.0,0.0,0.0,34.0,5.0,0.0,1.0,2.0,0.0,4.0,6.0,7.0,0.0,0.0,1.0,1.0,2.0,0.0,1.0,24.0,0.0,2.0,1.0,1.0,0.0,2.0,0.0,28.0,4.0,4.0,1.0,3.0,1.0,2.0,6.0,8.0,0.0,1.0,0.0,3.0,0.0,1.0,0.0,24.0,1.0,0.0,1.0,1.0,0.0,0.0,0.0,53.0,11.0,6.0,2.0,2.0,3.0,3.0,8.0,7.0,2.0,3.0,0.0,1.0,0.0,0.0,0.0,11.0,0.0,0.0,1.0,0.0,1.0,0.0,1.0,81.0,19.0,9.0,3.0,2.0,3.0,2.0,16.0,12.0,0.0,1.0,0.0,2.0,0.0,0.0,1.0,28.0,0.0,0.0,1.0,0.0,1.0,0.0,1.0,30.0,3.0,0.0,2.0,0.0,0.0,0.0,6.0,7.0,0.0,0.0,1.0,0.0,1.0,0.0,1.0,28.0,0.0,0.0,0.0,0.0,1.0,0.0,0.0,23.0,2.0,0.0,0.0,2.0,0.0,2.0,4.0,10.0,0.0,0.0,1.0,1.0,0.0,0.0,0.0,25.0,0.0,2.0,2.0,1.0,1.0,0.0,2.0,18.0,1.0,0.0,0.0,4.0,0.0,1.0,1.0,5.0,0.0,0.0,0.0,1.0,0.0,0.0,0.0,14.0,0.0,1.0,0.0,0.0,0.0,0.0,0.0,8.0,0.0,0.0,0.0,1.0,0.0,2.0,2.0,3.0,0.0,0.0,1.0,1.0,0.0,0.0,0.0,14.0,0.0,1.0,0.0,0.0,1.0,0.0,1.0,6.0,0.0,0.0,0.0,0.0,0.0,0.0,2.0,3.0,0.0,0.0,1.0,1.0,0.0,0.0,0.0,16.0,0.0,1.0,0.0,2.0,0.0,0.0,0.0,6.0,0.0,0.0,0.0,3.0,0.0,1.0,4.0,4.0,0.0,0.0,0.0,3.0,2.0,0.0,0.0,18.0,0.0,1.0,0.0,0.0,0.0,0.0,0.0,23.0,2.0,0.0,0.0,2.0,0.0,4.0,2.0,5.0,0.0,0.0,0.0,3.0,0.0,0.0,0.0,17.0,0.0,1.0,1.0,1.0,0.0,0.0,1.0,84.0,24.0,2.0,2.0,10.0,1.0,2.0,18.0,20.0,1.0,2.0,0.0,4.0,2.0,0.0,3.0,34.0,0.0,2.0,1.0,1.0,0.0,2.0,1.0,14.0,2.0,0.0,0.0,2.0,0.0,1.0,5.0,12.0,0.0,0.0,0.0,2.0,0.0,0.0,2.0,17.0,0.0,1.0,0.0,1.0,0.0,0.0,1.0,15.0,1.0,0.0,0.0,2.0,0.0,1.0,2.0,4.0,0.0,0.0,0.0,1.0,0.0,0.0,0.0,17.0,0.0,0.0,1.0,1.0,0.0,0.0,1.0,55.0,9.0,1.0,1.0,5.0,0.0,5.0,13.0,10.0,0.0,0.0,0.0,0.0,1.0,3.0,0.0,25.0,1.0,2.0,0.0,0.0,0.0,0.0,0.0,94.0,25.0,9.0,8.0,2.0,3.0,5.0,23.0,8.0,1.0,2.0,0.0,0.0,1.0,0.0,0.0,25.0,0.0,1.0,0.0,2.0,1.0,0.0,1.0,42.0,11.0,0.0,1.0,1.0,0.0,5.0,5.0,12.0,0.0,0.0,2.0,0.0,0.0,1.0,0.0,22.0,0.0,1.0,0.0,2.0,1.0,1.0,1.0,8.0,0.0,0.0,0.0,0.0,0.0,0.0,2.0,5.0,0.0,0.0,1.0,2.0,0.0,0.0,0.0,18.0,0.0,1.0,1.0,1.0,0.0,1.0,1.0,32.0,0.0,1.0,0.0,0.0,0.0,2.0,4.0,6.0,0.0,0.0,1.0,4.0,0.0,0.0,0.0,27.0,0.0,1.0,1.0,0.0,0.0,0.0,0.0,7.0,2.0,0.0,0.0,2.0,0.0,2.0,3.0,4.0,0.0,0.0,0.0,2.0,0.0,0.0,0.0,23.0,0.0,0.0,0.0,0.0,0.0,0.0,1.0,94.0,23.0,4.0,5.0,5.0,2.0,7.0,16.0,15.0,1.0,0.0,0.0,2.0,2.0,0.0,2.0,37.0,1.0,0.0,1.0,1.0,0.0,1.0,1.0,88.0,20.0,7.0,5.0,4.0,1.0,7.0,19.0,17.0,0.0,1.0,0.0,4.0,3.0,2.0,1.0,32.0,1.0,0.0,1.0,1.0,0.0,2.0,1.0,22.0,7.0,0.0,0.0,0.0,0.0,0.0,7.0,12.0,1.0,3.0,1.0,2.0,0.0,0.0,1.0,33.0,0.0,1.0,1.0,0.0,1.0,0.0,0.0,43.0,11.0,0.0,0.0,4.0,0.0,6.0,12.0,15.0,0.0,0.0,3.0,3.0,1.0,0.0,2.0,39.0,1.0,2.0,0.0,0.0,0.0,2.0,1.0,10.0,0.0,0.0,0.0,0.0,0.0,0.0,1.0,6.0,0.0,0.0,1.0,2.0,0.0,0.0,0.0,27.0,0.0,0.0,0.0,0.0,0.0,0.0,1.0,4.0,0.0,0.0,0.0,0.0,0.0,0.0,2.0,6.0,0.0,0.0,1.0,1.0,0.0,0.0,0.0,28.0,0.0,2.0,0.0,0.0,0.0,0.0,0.0,4.0,0.0,0.0,0.0,0.0,0.0,0.0,1.0,2.0,0.0,0.0,0.0,1.0,0.0,0.0,0.0,15.0,0.0,1.0,0.0,1.0,0.0,2.0,0.0,8.0,0.0,0.0,0.0,0.0,0.0,1.0,3.0,3.0,0.0,0.0,0.0,4.0,0.0,0.0,0.0,23.0,0.0,0.0,0.0,0.0,0.0,2.0,0.0,18.0,2.0,0.0,0.0,3.0,0.0,2.0,6.0,6.0,0.0,0.0,0.0,2.0,0.0,0.0,1.0,21.0,0.0,0.0,1.0,0.0,2.0,1.0,1.0,23.0,0.0,1.0,2.0,4.0,0.0,0.0,5.0,6.0,0.0,1.0,0.0,2.0,1.0,1.0,0.0,20.0,0.0,0.0,0.0,3.0,0.0,0.0,0.0,80.0,16.0,10.0,5.0,3.0,3.0,1.0,10.0,12.0,2.0,1.0,0.0,4.0,0.0,0.0,0.0,21.0,1.0,0.0,1.0,2.0,0.0,0.0,0.0,20.0,0.0,1.0,0.0,1.0,0.0,1.0,3.0,5.0,0.0,0.0,0.0,0.0,0.0,0.0,0.0,35.0,0.0,1.0,0.0,1.0,1.0,0.0,0.0,7.0,0.0,0.0,0.0,0.0,0.0,1.0,1.0,8.0,0.0,0.0,0.0,1.0,0.0,0.0,0.0,19.0,0.0,1.0,0.0,1.0,2.0,0.0,1.0,1.0,0.0,0.0,0.0,0.0,0.0,0.0,1.0,2.0,0.0,0.0,1.0,0.0,0.0,0.0,0.0,32.0,0.0,1.0,0.0,1.0,0.0,1.0,0.0,3.0,0.0,0.0,0.0,0.0,0.0,0.0,1.0,3.0,0.0,0.0,0.0,1.0,0.0,0.0,0.0,18.0,0.0,2.0,0.0,0.0,0.0,0.0,0.0,11.0,2.0,0.0,0.0,1.0,1.0,2.0,3.0,4.0,0.0,0.0,0.0,3.0,0.0,1.0,0.0,21.0,0.0,0.0,0.0,0.0,1.0,1.0,1.0,67.0,10.0,5.0,0.0,4.0,3.0,2.0,11.0,24.0,2.0,1.0,0.0,2.0,2.0,3.0,3.0,27.0,0.0,0.0,1.0,1.0,0.0,0.0,3.0,234.0,43.0,19.0,14.0,15.0,9.0,18.0,34.0,53.0,0.0,1.0,1.0,12.0,10.0,0.0,6.0,44.0,0.0,1.0,1.0,2.0,0.0,3.0,3.0,11.0,2.0,0.0,0.0,0.0,0.0,2.0,7.0,8.0,0.0,0.0,0.0,1.0,0.0,0.0,0.0,16.0,0.0,2.0,3.0,0.0,1.0,0.0,1.0,2.0,0.0,0.0,0.0,0.0,0.0,0.0,1.0,3.0,0.0,0.0,0.0,0.0,0.0,0.0,0.0,18.0,0.0,1.0,0.0,0.0,0.0,1.0,0.0,2.0,0.0,0.0,0.0,0.0,0.0,0.0,1.0,7.0,0.0,0.0,0.0,1.0,0.0,0.0,0.0,20.0,0.0,1.0,0.0,1.0,1.0,0.0,0.0,24.0,1.0,1.0,0.0,2.0,1.0,2.0,6.0,9.0,0.0,0.0,0.0,2.0,2.0,0.0,1.0,24.0,1.0,2.0,1.0,2.0,0.0,1.0,1.0,58.0,14.0,4.0,4.0,2.0,2.0,2.0,12.0,6.0,1.0,1.0,0.0,0.0,0.0,1.0,0.0,25.0,1.0,1.0,2.0,2.0,1.0,0.0,1.0,36.0,3.0,2.0,2.0,0.0,1.0,0.0,8.0,10.0,1.0,3.0,0.0,0.0,0.0,3.0,0.0,35.0,2.0,0.0,2.0,1.0,0.0,0.0,0.0,30.0,12.0,3.0,0.0,2.0,1.0,2.0,3.0,10.0,1.0,3.0,0.0,1.0,2.0,1.0,1.0,36.0,0.0,1.0,1.0,2.0,1.0,0.0,1.0,32.0,6.0,1.0,1.0,5.0,0.0,5.0,6.0,12.0,1.0,1.0,0.0,0.0,0.0,3.0,1.0,35.0,0.0,1.0,2.0,2.0,1.0,1.0,1.0,3.0,1.0,0.0,0.0,1.0,0.0,0.0,0.0,6.0,0.0,0.0,1.0,0.0,1.0,0.0,0.0,26.0,0.0,1.0,1.0,1.0,1.0,1.0,0.0,2.0,0.0,0.0,0.0,0.0,0.0,0.0,2.0,4.0,0.0,0.0,0.0,1.0,0.0,0.0,0.0,21.0,0.0,2.0,0.0,3.0,0.0,0.0,0.0,2.0,0.0,0.0,0.0,0.0,0.0,0.0,1.0,8.0,0.0,0.0,1.0,1.0,0.0,0.0,0.0,24.0,0.0,3.0,0.0,1.0,0.0,1.0,1.0,8.0,1.0,0.0,0.0,0.0,0.0,1.0,2.0,6.0,0.0,0.0,1.0,0.0,0.0,0.0,0.0,24.0,0.0,1.0,2.0,0.0,0.0,0.0,1.0,36.0,5.0,4.0,2.0,3.0,0.0,1.0,4.0,6.0,1.0,0.0,0.0,0.0,1.0,0.0,0.0,37.0,1.0,0.0,2.0,2.0,0.0,0.0,0.0,10.0,1.0,0.0,0.0,0.0,0.0,1.0,1.0,3.0,0.0,0.0,0.0,0.0,0.0,0.0,0.0,30.0,0.0,1.0,1.0,1.0,2.0,1.0,1.0,3.0,0.0,0.0,0.0,0.0,0.0,0.0,1.0,5.0,0.0,0.0,2.0,2.0,0.0,0.0,0.0,22.0,0.0,1.0,0.0,0.0,0.0,1.0,1.0,7.0,0.0,0.0,0.0,2.0,0.0,0.0,3.0,5.0,0.0,0.0,1.0,0.0,0.0,1.0,0.0,21.0,0.0,1.0,0.0,2.0,0.0,1.0,0.0,29.0,3.0,3.0,0.0,3.0,0.0,3.0,8.0,15.0,1.0,1.0,0.0,0.0,0.0,3.0,1.0,26.0,0.0,1.0,0.0,1.0,0.0,1.0,1.0,88.0,22.0,5.0,10.0,1.0,2.0,4.0,12.0,10.0,0.0,0.0,0.0,0.0,0.0,2.0,1.0,31.0,0.0,0.0,0.0,0.0,0.0,2.0,0.0,48.0,9.0,3.0,3.0,3.0,2.0,1.0,6.0,13.0,2.0,2.0,2.0,2.0,0.0,2.0,2.0,33.0,2.0,0.0,2.0,1.0,0.0,0.0,1.0,59.0,13.0,2.0,2.0,3.0,1.0,5.0,13.0,13.0,0.0,1.0,1.0,2.0,1.0,0.0,0.0,21.0,0.0,1.0,0.0,0.0,0.0,1.0,1.0,31.0,3.0,0.0,0.0,4.0,1.0,3.0,7.0,13.0,1.0,0.0,1.0,1.0,2.0,0.0,1.0,23.0,0.0,2.0,0.0,1.0,0.0,0.0,3.0,7.0,0.0,0.0,0.0,0.0,0.0,1.0,1.0,8.0,0.0,0.0,0.0,2.0,0.0,0.0,0.0,28.0,0.0,0.0,1.0,2.0,0.0,3.0,1.0,26.0,5.0,3.0,1.0,0.0,1.0,0.0,4.0,7.0,0.0,1.0,0.0,0.0,0.0,1.0,0.0,34.0,0.0,0.0,0.0,3.0,1.0,0.0,1.0,3.0,0.0,0.0,0.0,0.0,0.0,0.0,1.0,3.0,0.0,0.0,0.0,0.0,0.0,0.0,0.0,22.0,0.0,1.0,0.0,1.0,0.0,1.0,0.0,8.0,0.0,0.0,0.0,0.0,0.0,1.0,1.0,4.0,0.0,0.0,0.0,0.0,0.0,0.0,0.0,26.0,0.0,0.0,0.0,1.0,0.0,0.0,0.0,9.0,0.0,0.0,0.0,0.0,1.0,0.0,1.0,5.0,0.0,0.0,0.0,1.0,0.0,1.0,0.0,24.0,0.0,1.0,1.0,0.0,0.0,1.0,1.0,24.0,2.0,2.0,0.0,1.0,0.0,2.0,9.0,6.0,1.0,0.0,0.0,1.0,1.0,1.0,0.0,25.0,0.0,0.0,1.0,1.0,0.0,1.0,1.0,37.0,5.0,1.0,0.0,2.0,0.0,2.0,3.0,12.0,2.0,1.0,0.0,2.0,0.0,0.0,0.0,31.0,0.0,1.0,2.0,2.0,0.0,0.0,0.0,8.0,0.0,0.0,0.0,0.0,0.0,2.0,1.0,2.0,0.0,0.0,1.0,0.0,1.0,1.0,0.0,23.0,0.0,1.0,1.0,1.0,0.0,0.0,0.0,7.0,0.0,0.0,0.0,1.0,0.0,1.0,1.0,10.0,0.0,0.0,0.0,1.0,0.0,0.0,0.0,34.0,0.0,1.0,0.0,1.0,1.0,2.0,0.0,7.0,0.0,0.0,0.0,0.0,0.0,0.0,1.0,5.0,0.0,0.0,0.0,1.0,0.0,0.0,0.0,27.0,0.0,2.0,0.0,2.0,0.0,0.0,0.0,5.0,0.0,0.0,0.0,0.0,0.0,0.0,1.0,3.0,0.0,0.0,1.0,0.0,0.0,0.0,0.0,16.0,0.0,1.0,0.0,1.0,0.0,1.0,0.0,5.0,0.0,0.0,0.0,0.0,0.0,1.0,2.0,6.0,0.0,0.0,0.0,1.0,0.0,0.0,0.0,31.0,0.0,0.0,0.0,1.0,0.0,3.0,1.0,33.0,5.0,0.0,0.0,3.0,0.0,3.0,5.0,11.0,0.0,0.0,0.0,2.0,0.0,0.0,0.0,34.0,0.0,4.0,1.0,2.0,1.0,1.0,1.0,52.0,8.0,2.0,1.0,2.0,0.0,3.0,15.0,11.0,0.0,1.0,1.0,4.0,0.0,1.0,1.0,49.0,0.0,0.0,2.0,1.0,1.0,1.0,1.0,55.0,9.0,1.0,2.0,5.0,1.0,5.0,10.0,16.0,0.0,1.0,0.0,3.0,2.0,0.0,1.0,17.0,0.0,0.0,1.0,1.0,0.0,1.0,4.0,115.0,25.0,10.0,10.0,3.0,1.0,5.0,19.0,21.0,0.0,0.0,1.0,3.0,5.0,1.0,0.0,39.0,0.0,0.0,0.0,3.0,1.0,1.0,1.0,40.0,10.0,4.0,4.0,0.0,2.0,3.0,13.0,7.0,0.0,2.0,0.0,0.0,0.0,5.0,1.0,29.0,0.0,0.0,2.0,0.0,1.0,0.0,0.0,38.0,4.0,0.0,0.0,1.0,2.0,3.0,8.0,8.0,1.0,0.0,0.0,1.0,0.0,1.0,2.0,27.0,1.0,0.0,0.0,1.0,0.0,0.0,0.0,101.0,26.0,9.0,6.0,6.0,2.0,5.0,17.0,17.0,0.0,0.0,0.0,2.0,7.0,0.0,1.0,26.0,1.0,1.0,1.0,4.0,0.0,3.0,2.0,5.0,0.0,0.0,0.0,1.0,0.0,0.0,1.0,2.0,0.0,0.0,0.0,0.0,0.0,0.0,0.0,26.0,0.0,0.0,0.0,1.0,0.0,0.0,1.0,4.0,0.0,0.0,0.0,0.0,0.0,0.0,1.0,2.0,0.0,0.0,0.0,0.0,0.0,0.0,0.0,22.0,0.0,1.0,0.0,0.0,0.0,1.0,0.0,4.0,0.0,0.0,0.0,0.0,0.0,0.0,2.0,2.0,0.0,0.0,0.0,1.0,0.0,0.0,0.0,26.0,0.0,2.0,0.0,2.0,0.0,1.0,0.0,10.0,0.0,0.0,0.0,2.0,0.0,3.0,5.0,10.0,0.0,0.0,1.0,1.0,0.0,0.0,0.0,24.0,0.0,2.0,0.0,1.0,0.0,1.0,1.0,8.0,0.0,0.0,0.0,0.0,0.0,1.0,4.0,7.0,0.0,0.0,1.0,1.0,1.0,0.0,0.0,29.0,0.0,3.0,1.0,1.0,0.0,1.0,2.0,227.0,43.0,19.0,14.0,12.0,8.0,16.0,31.0,56.0,4.0,1.0,1.0,12.0,9.0,0.0,3.0,50.0,1.0,0.0,2.0,5.0,1.0,3.0,4.0,67.0,15.0,7.0,3.0,1.0,1.0,2.0,14.0,7.0,1.0,1.0,0.0,1.0,0.0,0.0,0.0,25.0,1.0,0.0,0.0,0.0,0.0,0.0,0.0,76.0,8.0,5.0,6.0,2.0,4.0,5.0,16.0,20.0,2.0,1.0,0.0,1.0,1.0,1.0,2.0,27.0,1.0,0.0,0.0,1.0,1.0,1.0,0.0,18.0,4.0,0.0,0.0,1.0,1.0,3.0,2.0,4.0,0.0,0.0,1.0,0.0,0.0,0.0,0.0,26.0,0.0,2.0,0.0,1.0,1.0,0.0,1.0,16.0,1.0,0.0,0.0,1.0,0.0,3.0,4.0,12.0,0.0,0.0,1.0,1.0,2.0,0.0,0.0,36.0,0.0,2.0,1.0,2.0,0.0,1.0,3.0,4.0,0.0,0.0,0.0,0.0,0.0,0.0,0.0,5.0,0.0,0.0,0.0,1.0,1.0,0.0,0.0,27.0,0.0,0.0,0.0,1.0,0.0,2.0,2.0,5.0,0.0,0.0,0.0,0.0,0.0,0.0,1.0,4.0,0.0,0.0,0.0,0.0,0.0,0.0,0.0,30.0,0.0,1.0,0.0,3.0,0.0,1.0,1.0,5.0,0.0,0.0,0.0,0.0,0.0,1.0,2.0,3.0,0.0,0.0,1.0,0.0,0.0,0.0,0.0,18.0,0.0,2.0,0.0,1.0,0.0,1.0,1.0,10.0,0.0,0.0,0.0,0.0,0.0,1.0,0.0,2.0,0.0,0.0,0.0,0.0,0.0,0.0,0.0,25.0,0.0,1.0,1.0,3.0,0.0,2.0,1.0,6.0,0.0,0.0,0.0,0.0,0.0,0.0,1.0,4.0,0.0,0.0,0.0,1.0,0.0,0.0,0.0,28.0,0.0,3.0,1.0,2.0,0.0,1.0,0.0,13.0,1.0,0.0,0.0,1.0,0.0,2.0,2.0,10.0,1.0,0.0,1.0,4.0,0.0,1.0,0.0,41.0,1.0,1.0,2.0,1.0,0.0,0.0,1.0,32.0,4.0,0.0,0.0,2.0,0.0,3.0,9.0,11.0,1.0,0.0,0.0,1.0,1.0,1.0,1.0,34.0,0.0,0.0,1.0,1.0,0.0,2.0,2.0,37.0,10.0,3.0,2.0,0.0,1.0,1.0,8.0,2.0,0.0,0.0,0.0,0.0,1.0,0.0,0.0,43.0,1.0,0.0,0.0,0.0,1.0,0.0,0.0,58.0,11.0,9.0,4.0,0.0,1.0,2.0,11.0,7.0,1.0,0.0,0.0,0.0,1.0,2.0,2.0,34.0,0.0,0.0,1.0,2.0,3.0,0.0,1.0,65.0,12.0,6.0,6.0,5.0,2.0,8.0,17.0,28.0,3.0,6.0,0.0,0.0,0.0,6.0,3.0,35.0,2.0,1.0,1.0,2.0,0.0,1.0,1.0,28.0,3.0,0.0,0.0,2.0,0.0,4.0,4.0,13.0,0.0,0.0,0.0,1.0,3.0,1.0,0.0,24.0,0.0,2.0,1.0,1.0,0.0,1.0,4.0,23.0,1.0,0.0,0.0,0.0,0.0,2.0,6.0,14.0,0.0,0.0,0.0,1.0,0.0,0.0,0.0,32.0,0.0,1.0,0.0,4.0,0.0,0.0,2.0,4.0,0.0,0.0,0.0,1.0,0.0,1.0,0.0,5.0,0.0,0.0,0.0,0.0,0.0,0.0,1.0,26.0,0.0,0.0,0.0,1.0,0.0,0.0,1.0,4.0,0.0,0.0,0.0,0.0,0.0,0.0,2.0,5.0,0.0,0.0,0.0,0.0,0.0,0.0,0.0,36.0,0.0,1.0,0.0,1.0,0.0,1.0,1.0,3.0,0.0,0.0,0.0,0.0,0.0,0.0,1.0,6.0,0.0,0.0,0.0,0.0,1.0,0.0,0.0,28.0,0.0,4.0,1.0,1.0,0.0,0.0,0.0,8.0,0.0,0.0,0.0,0.0,0.0,2.0,2.0,4.0,0.0,0.0,0.0,0.0,0.0,0.0,0.0,30.0,0.0,0.0,1.0,1.0,0.0,0.0,1.0,20.0,2.0,1.0,0.0,1.0,1.0,2.0,9.0,8.0,0.0,0.0,0.0,2.0,0.0,1.0,0.0,42.0,1.0,3.0,2.0,2.0,0.0,0.0,2.0,56.0,8.0,1.0,1.0,1.0,0.0,4.0,13.0,10.0,3.0,0.0,1.0,1.0,1.0,1.0,0.0,31.0,0.0,1.0,0.0,1.0,0.0,1.0,1.0,76.0,18.0,5.0,6.0,1.0,2.0,2.0,14.0,8.0,0.0,1.0,0.0,0.0,0.0,0.0,2.0,49.0,1.0,0.0,2.0,3.0,0.0,0.0,2.0,29.0,7.0,4.0,3.0,1.0,0.0,3.0,13.0,11.0,0.0,0.0,0.0,1.0,0.0,3.0,0.0,41.0,2.0,2.0,2.0,1.0,1.0,0.0,0.0,80.0,20.0,7.0,9.0,2.0,2.0,3.0,11.0,16.0,1.0,2.0,0.0,1.0,3.0,1.0,2.0,33.0,0.0,1.0,1.0,0.0,1.0,0.0,3.0,37.0,4.0,0.0,1.0,2.0,0.0,2.0,4.0,13.0,0.0,0.0,1.0,2.0,0.0,0.0,0.0,34.0,1.0,1.0,1.0,3.0,0.0,0.0,3.0,2.0,0.0,0.0,0.0,1.0,0.0,0.0,1.0,5.0,0.0,0.0,0.0,1.0,0.0,0.0,0.0,24.0,0.0,1.0,0.0,1.0,0.0,2.0,0.0,5.0,0.0,0.0,0.0,0.0,0.0,0.0,2.0,10.0,0.0,0.0,2.0,0.0,2.0,0.0,0.0,30.0,0.0,0.0,0.0,0.0,0.0,0.0,0.0,12.0,2.0,0.0,0.0,0.0,0.0,2.0,4.0,7.0,0.0,0.0,0.0,2.0,0.0,0.0,1.0,28.0,0.0,3.0,0.0,4.0,0.0,1.0,2.0,22.0,2.0,1.0,0.0,3.0,0.0,1.0,2.0,8.0,0.0,0.0,0.0,1.0,0.0,0.0,0.0,33.0,0.0,2.0,0.0,1.0,0.0,1.0,1.0,27.0,6.0,1.0,0.0,0.0,1.0,3.0,6.0,7.0,0.0,0.0,0.0,0.0,0.0,1.0,0.0,32.0,0.0,2.0,1.0,1.0,0.0,1.0,1.0,111.0,28.0,13.0,10.0,3.0,5.0,4.0,15.0,14.0,0.0,1.0,0.0,1.0,0.0,1.0,1.0,34.0,1.0,1.0,2.0,1.0,1.0,1.0,3.0,44.0,5.0,8.0,2.0,0.0,2.0,3.0,9.0,15.0,2.0,1.0,0.0,0.0,0.0,0.0,0.0,47.0,2.0,2.0,1.0,1.0,1.0,1.0,3.0,37.0,10.0,2.0,3.0,0.0,1.0,1.0,9.0,7.0,1.0,0.0,1.0,3.0,0.0,1.0,1.0,45.0,2.0,0.0,3.0,2.0,1.0,1.0,1.0,34.0,6.0,1.0,1.0,0.0,0.0,5.0,4.0,6.0,1.0,1.0,0.0,0.0,0.0,1.0,0.0,27.0,0.0,1.0,0.0,2.0,1.0,0.0,1.0,5.0,0.0,0.0,0.0,0.0,0.0,0.0,0.0,5.0,0.0,0.0,2.0,0.0,0.0,0.0,0.0,31.0,0.0,0.0,0.0,0.0,2.0,0.0,0.0,4.0,0.0,0.0,0.0,0.0,0.0,2.0,2.0,5.0,0.0,0.0,0.0,0.0,0.0,0.0,0.0,34.0,0.0,0.0,0.0,1.0,0.0,2.0,1.0,2.0,0.0,0.0,0.0,1.0,0.0,3.0,2.0,4.0,0.0,0.0,1.0,0.0,0.0,0.0,0.0,24.0,0.0,2.0,0.0,0.0,0.0,1.0,1.0,5.0,1.0,0.0,0.0,0.0,0.0,1.0,3.0,7.0,0.0,0.0,0.0,1.0,1.0,0.0,0.0,34.0,0.0,1.0,0.0,0.0,0.0,1.0,0.0,5.0,0.0,0.0,0.0,1.0,0.0,1.0,3.0,7.0,0.0,0.0,1.0,1.0,0.0,0.0,0.0,27.0,0.0,1.0,0.0,0.0,0.0,2.0,3.0,15.0,1.0,0.0,0.0,2.0,0.0,1.0,6.0,9.0,0.0,0.0,0.0,2.0,0.0,0.0,0.0,33.0,0.0,2.0,2.0,2.0,0.0,1.0,1.0,27.0,4.0,1.0,0.0,4.0,0.0,3.0,8.0,10.0,0.0,0.0,1.0,4.0,0.0,0.0,0.0,26.0,1.0,1.0,2.0,2.0,1.0,0.0,2.0,68.0,12.0,6.0,5.0,4.0,1.0,5.0,18.0,24.0,1.0,4.0,0.0,1.0,2.0,2.0,1.0,25.0,0.0,1.0,0.0,1.0,1.0,0.0,2.0,153.0,34.0,13.0,12.0,10.0,4.0,11.0,21.0,31.0,1.0,0.0,1.0,8.0,2.0,0.0,3.0,41.0,0.0,1.0,1.0,4.0,0.0,2.0,5.0,43.0,2.0,2.0,1.0,0.0,2.0,3.0,14.0,8.0,0.0,0.0,0.0,2.0,0.0,1.0,0.0,38.0,1.0,1.0,0.0,1.0,1.0,0.0,0.0,13.0,2.0,0.0,0.0,2.0,0.0,3.0,3.0,7.0,0.0,0.0,0.0,1.0,0.0,0.0,1.0,25.0,0.0,1.0,0.0,1.0,0.0,1.0,1.0,20.0,1.0,0.0,0.0,0.0,0.0,4.0,4.0,7.0,0.0,0.0,0.0,1.0,1.0,0.0,0.0,31.0,0.0,2.0,0.0,2.0,0.0,0.0,3.0,18.0,0.0,1.0,0.0,1.0,1.0,1.0,3.0,6.0,0.0,0.0,0.0,1.0,0.0,0.0,1.0,28.0,0.0,1.0,0.0,2.0,0.0,0.0,2.0,4.0,0.0,0.0,0.0,0.0,0.0,0.0,0.0,1.0,0.0,0.0,0.0,1.0,0.0,0.0,0.0,27.0,0.0,2.0,0.0,1.0,0.0,3.0,1.0,4.0,0.0,0.0,0.0,1.0,0.0,0.0,1.0,1.0,0.0,0.0,0.0,1.0,0.0,0.0,0.0,23.0,0.0,1.0,0.0,0.0,0.0,1.0,0.0,6.0,0.0,0.0,0.0,1.0,0.0,0.0,1.0,5.0,0.0,0.0,1.0,1.0,0.0,0.0,0.0,30.0,0.0,2.0,0.0,1.0,0.0,0.0,0.0,18.0,1.0,0.0,0.0,2.0,0.0,3.0,2.0,12.0,0.0,0.0,0.0,2.0,0.0,0.0,0.0,35.0,1.0,2.0,0.0,1.0,0.0,1.0,3.0,51.0,10.0,2.0,2.0,2.0,2.0,7.0,14.0,17.0,1.0,2.0,0.0,0.0,0.0,2.0,1.0,34.0,1.0,1.0,1.0,1.0,0.0,0.0,1.0,96.0,22.0,9.0,12.0,2.0,0.0,5.0,15.0,14.0,3.0,2.0,1.0,1.0,2.0,4.0,1.0,27.0,1.0,0.0,2.0,1.0,0.0,0.0,1.0,56.0,12.0,4.0,7.0,1.0,0.0,4.0,13.0,10.0,0.0,1.0,0.0,1.0,0.0,2.0,2.0,32.0,0.0,1.0,0.0,0.0,2.0,1.0,0.0,89.0,15.0,2.0,2.0,8.0,0.0,6.0,16.0,22.0,3.0,0.0,0.0,3.0,2.0,1.0,3.0,39.0,1.0,3.0,0.0,1.0,0.0,2.0,2.0,16.0,0.0,0.0,0.0,0.0,0.0,3.0,5.0,7.0,0.0,0.0,2.0,0.0,0.0,0.0,0.0,30.0,0.0,1.0,0.0,1.0,0.0,1.0,1.0,6.0,0.0,0.0,0.0,1.0,0.0,0.0,1.0,5.0,0.0,0.0,2.0,1.0,0.0,0.0,0.0,18.0,0.0,3.0,0.0,1.0,0.0,0.0,2.0,6.0,0.0,0.0,0.0,1.0,0.0,1.0,1.0,4.0,0.0,0.0,1.0,0.0,0.0,0.0,0.0,36.0,0.0,3.0,0.0,2.0,0.0,0.0,0.0,7.0,0.0,0.0,0.0,1.0,0.0,0.0,1.0,3.0,0.0,0.0,0.0,0.0,0.0,0.0,0.0,35.0,0.0,1.0,0.0,0.0,0.0,1.0,2.0,4.0,0.0,0.0,0.0,1.0,0.0,0.0,2.0,6.0,0.0,0.0,0.0,3.0,0.0,0.0,0.0,33.0,0.0,1.0,0.0,2.0,0.0,1.0,0.0,4.0,1.0,0.0,0.0,0.0,0.0,1.0,0.0,7.0,0.0,0.0,0.0,1.0,0.0,0.0,0.0,20.0,0.0,1.0,0.0,1.0,1.0,0.0,0.0,21.0,1.0,0.0,0.0,1.0,0.0,2.0,5.0,9.0,1.0,0.0,1.0,1.0,1.0,0.0,0.0,40.0,0.0,0.0,0.0,2.0,1.0,0.0,1.0,22.0,1.0,1.0,0.0,2.0,0.0,2.0,5.0,7.0,0.0,0.0,0.0,1.0,0.0,1.0,0.0,42.0,0.0,1.0,0.0,0.0,3.0,0.0,1.0,77.0,12.0,3.0,6.0,1.0,1.0,8.0,16.0,14.0,0.0,1.0,0.0,2.0,1.0,6.0,1.0,26.0,0.0,0.0,1.0,3.0,0.0,1.0,1.0,69.0,13.0,8.0,9.0,2.0,1.0,3.0,11.0,6.0,1.0,0.0,0.0,0.0,0.0,1.0,0.0,28.0,2.0,1.0,0.0,3.0,1.0,0.0,0.0,58.0,10.0,2.0,2.0,1.0,3.0,5.0,14.0,18.0,2.0,2.0,0.0,1.0,1.0,5.0,2.0,32.0,1.0,0.0,1.0,0.0,0.0,1.0,2.0,32.0,4.0,0.0,1.0,2.0,0.0,5.0,6.0,16.0,0.0,0.0,1.0,2.0,0.0,1.0,1.0,35.0,0.0,1.0,1.0,0.0,1.0,1.0,1.0,17.0,0.0,0.0,0.0,1.0,0.0,2.0,7.0,9.0,0.0,0.0,2.0,1.0,1.0,0.0,0.0,30.0,0.0,3.0,0.0,0.0,0.0,1.0,3.0,5.0,0.0,0.0,0.0,0.0,0.0,1.0,2.0,8.0,0.0,0.0,1.0,1.0,0.0,0.0,0.0,37.0,0.0,1.0,0.0,1.0,0.0,0.0,0.0,5.0,0.0,0.0,0.0,0.0,0.0,0.0,0.0,2.0,0.0,0.0,0.0,0.0,0.0,0.0,0.0,28.0,0.0,4.0,0.0,0.0,0.0,1.0,1.0,3.0,0.0,0.0,0.0,0.0,0.0,0.0,1.0,2.0,0.0,0.0,0.0,0.0,0.0,0.0,0.0,27.0,0.0,3.0,0.0,1.0,0.0,1.0,0.0,6.0,0.0,0.0,0.0,0.0,0.0,2.0,3.0,11.0,0.0,0.0,0.0,1.0,0.0,1.0,0.0,34.0,0.0,1.0,0.0,1.0,0.0,1.0,1.0,13.0,1.0,0.0,0.0,1.0,0.0,3.0,2.0,7.0,0.0,0.0,1.0,2.0,0.0,0.0,0.0,36.0,0.0,0.0,0.0,2.0,1.0,1.0,1.0,45.0,5.0,0.0,0.0,3.0,0.0,6.0,5.0,7.0,0.0,1.0,0.0,3.0,0.0,0.0,1.0,42.0,0.0,4.0,1.0,4.0,0.0,1.0,2.0,36.0,2.0,1.0,0.0,2.0,0.0,3.0,13.0,9.0,0.0,0.0,0.0,1.0,1.0,2.0,1.0,32.0,0.0,0.0,0.0,0.0,0.0,0.0,1.0,107.0,24.0,11.0,7.0,2.0,3.0,11.0,24.0,32.0,1.0,4.0,0.0,6.0,3.0,4.0,5.0,50.0,2.0,1.0,1.0,2.0,0.0,1.0,3.0,45.0,15.0,2.0,3.0,1.0,0.0,2.0,9.0,14.0,1.0,1.0,1.0,0.0,0.0,3.0,1.0,34.0,2.0,0.0,2.0,2.0,0.0,1.0,1.0,94.0,29.0,6.0,6.0,2.0,1.0,6.0,13.0,15.0,0.0,0.0,1.0,1.0,1.0,0.0,1.0,38.0,0.0,0.0,2.0,2.0,1.0,0.0,1.0,27.0,7.0,0.0,0.0,0.0,0.0,4.0,5.0,7.0,0.0,0.0,2.0,1.0,0.0,0.0,0.0,26.0,0.0,1.0,0.0,1.0,0.0,1.0,0.0,8.0,0.0,0.0,0.0,1.0,0.0,3.0,2.0,8.0,0.0,0.0,1.0,0.0,0.0,0.0,0.0,30.0,0.0,5.0,0.0,0.0,0.0,0.0,0.0,7.0,0.0,0.0,0.0,0.0,0.0,1.0,1.0,5.0,0.0,0.0,0.0,1.0,0.0,0.0,0.0,37.0,0.0,2.0,0.0,1.0,0.0,1.0,1.0,5.0,0.0,0.0,0.0,0.0,0.0,0.0,1.0,4.0,0.0,0.0,1.0,2.0,0.0,0.0,0.0,34.0,0.0,2.0,0.0,2.0,0.0,0.0,3.0,6.0,0.0,0.0,0.0,0.0,0.0,0.0,2.0,3.0,0.0,0.0,0.0,1.0,0.0,0.0,0.0,29.0,0.0,2.0,0.0,1.0,0.0,0.0,1.0,3.0,0.0,0.0,0.0,0.0,0.0,1.0,2.0,4.0,0.0,0.0,1.0,0.0,0.0,0.0,0.0,33.0,1.0,0.0,0.0,1.0,0.0,0.0,0.0,9.0,0.0,0.0,0.0,1.0,0.0,2.0,3.0,8.0,0.0,0.0,0.0,2.0,0.0,0.0,0.0,21.0,0.0,3.0,1.0,2.0,0.0,0.0,3.0,15.0,2.0,0.0,1.0,0.0,0.0,3.0,5.0,8.0,1.0,0.0,1.0,2.0,0.0,0.0,1.0,45.0,0.0,1.0,0.0,4.0,1.0,0.0,3.0,45.0,6.0,1.0,2.0,3.0,1.0,5.0,9.0,8.0,1.0,0.0,0.0,2.0,1.0,0.0,1.0,32.0,1.0,1.0,1.0,0.0,0.0,1.0,1.0,150.0,38.0,16.0,12.0,7.0,6.0,12.0,22.0,32.0,1.0,2.0,1.0,2.0,3.0,1.0,2.0,46.0,0.0,2.0,2.0,3.0,1.0,2.0,3.0,55.0,9.0,7.0,5.0,0.0,2.0,2.0,9.0,11.0,2.0,1.0,0.0,0.0,0.0,4.0,2.0,53.0,1.0,1.0,1.0,1.0,0.0,0.0,1.0,92.0,24.0,13.0,10.0,3.0,2.0,6.0,18.0,11.0,1.0,0.0,0.0,3.0,1.0,0.0,0.0,31.0,2.0,0.0,1.0,0.0,0.0,0.0,2.0,24.0,3.0,1.0,1.0,0.0,3.0,3.0,7.0,13.0,1.0,0.0,1.0,2.0,0.0,0.0,1.0,35.0,0.0,1.0,0.0,2.0,1.0,1.0,1.0,11.0,0.0,0.0,0.0,1.0,0.0,1.0,3.0,7.0,0.0,0.0,1.0,2.0,0.0,0.0,0.0,21.0,0.0,2.0,0.0,2.0,0.0,1.0,1.0,7.0,0.0,0.0,0.0,0.0,0.0,1.0,0.0,6.0,0.0,0.0,2.0,0.0,0.0,0.0,0.0,30.0,0.0,2.0,0.0,0.0,0.0,1.0,1.0,4.0,0.0,0.0,0.0,0.0,0.0,0.0,1.0,6.0,0.0,0.0,0.0,0.0,0.0,0.0,0.0,30.0,0.0,0.0,1.0,1.0,0.0,4.0,2.0,2.0,0.0,0.0,0.0,0.0,0.0,0.0,1.0,4.0,0.0,0.0,0.0,0.0,0.0,0.0,0.0,32.0,0.0,4.0,0.0,3.0,0.0,1.0,1.0,5.0,0.0,0.0,0.0,0.0,1.0,0.0,1.0,8.0,0.0,0.0,0.0,1.0,0.0,1.0,0.0,30.0,0.0,5.0,0.0,1.0,0.0,1.0,0.0,30.0,3.0,0.0,0.0,0.0,0.0,4.0,4.0,13.0,0.0,0.0,1.0,3.0,0.0,0.0,0.0,56.0,0.0,3.0,1.0,3.0,1.0,1.0,2.0,22.0,2.0,0.0,0.0,2.0,0.0,5.0,3.0,7.0,0.0,0.0,0.0,1.0,1.0,1.0,0.0,33.0,0.0,0.0,1.0,1.0,0.0,0.0,1.0,74.0,19.0,6.0,10.0,2.0,2.0,1.0,9.0,8.0,1.0,0.0,0.0,0.0,0.0,2.0,1.0,40.0,0.0,2.0,3.0,1.0,1.0,0.0,1.0,58.0,14.0,3.0,8.0,0.0,1.0,2.0,15.0,13.0,2.0,1.0,0.0,0.0,1.0,2.0,1.0,39.0,1.0,1.0,1.0,2.0,1.0,2.0,0.0,49.0,8.0,2.0,1.0,3.0,2.0,5.0,14.0,17.0,1.0,0.0,0.0,1.0,0.0,3.0,3.0,44.0,0.0,1.0,1.0,3.0,0.0,2.0,0.0,93.0,29.0,4.0,6.0,2.0,1.0,10.0,13.0,29.0,0.0,0.0,2.0,2.0,2.0,0.0,1.0,46.0,0.0,1.0,1.0,3.0,1.0,0.0,2.0,9.0,0.0,0.0,0.0,0.0,0.0,1.0,3.0,9.0,0.0,0.0,0.0,0.0,0.0,0.0,1.0,27.0,0.0,3.0,0.0,1.0,0.0,0.0,3.0,7.0,1.0,0.0,0.0,1.0,0.0,1.0,2.0,8.0,0.0,0.0,0.0,2.0,0.0,0.0,0.0,36.0,0.0,2.0,1.0,2.0,0.0,1.0,2.0,4.0,0.0,0.0,0.0,0.0,0.0,0.0,1.0,3.0,0.0,0.0,0.0,0.0,0.0,0.0,0.0,28.0,0.0,3.0,0.0,1.0,0.0,0.0,1.0,7.0,0.0,0.0,0.0,3.0,0.0,1.0,1.0,6.0,0.0,0.0,1.0,0.0,0.0,0.0,0.0,34.0,0.0,1.0,0.0,2.0,0.0,1.0,1.0,18.0,2.0,0.0,0.0,0.0,0.0,4.0,4.0,13.0,0.0,0.0,1.0,2.0,0.0,0.0,2.0,40.0,0.0,4.0,0.0,5.0,0.0,0.0,1.0,6.0,0.0,0.0,0.0,0.0,0.0,3.0,2.0,8.0,0.0,0.0,0.0,2.0,0.0,0.0,0.0,32.0,0.0,1.0,1.0,2.0,0.0,1.0,2.0,26.0,4.0,2.0,0.0,4.0,0.0,5.0,3.0,11.0,0.0,0.0,0.0,3.0,0.0,1.0,1.0,38.0,0.0,2.0,2.0,2.0,0.0,0.0,0.0,32.0,2.0,1.0,2.0,2.0,1.0,4.0,9.0,5.0,0.0,0.0,0.0,1.0,1.0,1.0,0.0,34.0,1.0,2.0,0.0,0.0,0.0,0.0,3.0,206.0,42.0,19.0,14.0,5.0,6.0,12.0,27.0,37.0,2.0,1.0,1.0,6.0,5.0,2.0,2.0,61.0,0.0,1.0,0.0,4.0,0.0,1.0,2.0,27.0,4.0,1.0,3.0,0.0,0.0,2.0,6.0,9.0,0.0,0.0,1.0,0.0,0.0,2.0,2.0,44.0,0.0,0.0,2.0,1.0,1.0,1.0,3.0,32.0,10.0,0.0,1.0,0.0,1.0,3.0,2.0,15.0,0.0,0.0,2.0,0.0,0.0,0.0,1.0,43.0,0.0,3.0,1.0,3.0,0.0,0.0,1.0,24.0,2.0,0.0,0.0,2.0,0.0,3.0,5.0,16.0,0.0,0.0,2.0,2.0,1.0,0.0,1.0,27.0,0.0,1.0,2.0,2.0,0.0,1.0,5.0,14.0,0.0,0.0,0.0,1.0,0.0,3.0,2.0,10.0,0.0,0.0,0.0,2.0,1.0,0.0,0.0,36.0,0.0,2.0,0.0,1.0,0.0,2.0,4.0,4.0,0.0,0.0,0.0,0.0,0.0,0.0,1.0,5.0,0.0,0.0,2.0,0.0,0.0,0.0,0.0,36.0,0.0,4.0,1.0,1.0,0.0,0.0,0.0,10.0,0.0,0.0,0.0,1.0,0.0,0.0,3.0,4.0,0.0,0.0,1.0,0.0,0.0,0.0,0.0,42.0,0.0,3.0,0.0,1.0,0.0,2.0,1.0,13.0,1.0,0.0,0.0,2.0,0.0,2.0,5.0,16.0,0.0,0.0,2.0,2.0,0.0,0.0,0.0,44.0,0.0,0.0,1.0,3.0,0.0,1.0,0.0,12.0,0.0,0.0,0.0,2.0,0.0,3.0,4.0,15.0,1.0,0.0,2.0,1.0,1.0,1.0,0.0,35.0,0.0,4.0,1.0,1.0,0.0,0.0,4.0,28.0,2.0,1.0,0.0,2.0,0.0,3.0,6.0,12.0,0.0,0.0,0.0,4.0,0.0,0.0,2.0,35.0,0.0,2.0,1.0,3.0,0.0,3.0,2.0,76.0,11.0,3.0,1.0,4.0,1.0,8.0,15.0,28.0,4.0,1.0,0.0,4.0,1.0,1.0,4.0,55.0,0.0,2.0,2.0,2.0,1.0,0.0,2.0,238.0,44.0,18.0,14.0,10.0,8.0,15.0,33.0,48.0,4.0,0.0,2.0,9.0,6.0,0.0,5.0,48.0,0.0,1.0,2.0,6.0,1.0,3.0,4.0,138.0,33.0,15.0,12.0,3.0,3.0,5.0,23.0,18.0,1.0,1.0,0.0,2.0,1.0,1.0,1.0,61.0,0.0,1.0,1.0,1.0,2.0,0.0,3.0,60.0,15.0,5.0,8.0,1.0,3.0,6.0,14.0,12.0,3.0,1.0,0.0,3.0,0.0,1.0,1.0,66.0,0.0,0.0,2.0,3.0,0.0,1.0,2.0,27.0,6.0,0.0,2.0,3.0,1.0,4.0,6.0,15.0,1.0,0.0,1.0,1.0,0.0,1.0,2.0,29.0,0.0,1.0,0.0,3.0,1.0,1.0,1.0,12.0,0.0,0.0,0.0,0.0,0.0,2.0,5.0,13.0,0.0,0.0,0.0,0.0,0.0,0.0,0.0,35.0,0.0,4.0,0.0,4.0,1.0,0.0,2.0,4.0,0.0,0.0,0.0,0.0,0.0,0.0,3.0,4.0,0.0,0.0,0.0,0.0,1.0,0.0,0.0,34.0,0.0,0.0,0.0,0.0,0.0,1.0,3.0,4.0,0.0,0.0,0.0,0.0,0.0,0.0,1.0,2.0,0.0,0.0,2.0,0.0,0.0,0.0,0.0,44.0,0.0,2.0,1.0,1.0,0.0,1.0,1.0,7.0,0.0,0.0,0.0,0.0,0.0,0.0,1.0,4.0,0.0,0.0,0.0,0.0,0.0,0.0,0.0,40.0,0.0,2.0,0.0,2.0,0.0,1.0,0.0,5.0,0.0,0.0,0.0,0.0,0.0,1.0,1.0,6.0,0.0,0.0,1.0,1.0,0.0,0.0,0.0,27.0,0.0,3.0,0.0,3.0,0.0,2.0,1.0),.Dim=c(274,24)))

#initial value for chain 3

list(a=c(-2.692,-3.554,-4.876,-6.496,-2.897,-4.592,-2.585,-2.088,-2.789,-4.323,-5.335,-2.775,-2.494,-3.738,-3.226,-2.97,-2.006,-3.567,-2.876,-2.191,-1.774,-2.658,-2.188,-1.895),

b=structure(.Data=c(0.1064,0.2307,0.3256,0.4836,0.06045,0.2075,0.0519,0.09143,0.02365,0.1655,0.3205,-0.02615,-0.009425,0.04119,0.1477,0.0845,-0.002079,0.1792,-0.06122,0.05257,-0.02951,0.1068,-0.08069,-0.02514,0.01029,0.0156,0.01494,0.0222,0.01141,0.01275,0.009749,0.00912,0.005518,0.01018,0.007061,-0.001202,0.009206,0.01205,0.003197,0.007935,0.001846,-0.003482,0.003147,0.002441,0.004702,0.001347,0.003641,0.006052,-0.004659,-0.01327,-0.01115,-0.01054,-0.005016,-0.009323,-0.001855,-3.519E-4,-6.106E-4,0.00351,-6.191E-5,-0.001886,-0.001643,-0.001306,0.01259,0.004338,-3.464E-4,0.002866,-0.001245,0.001237,-0.002871,-0.008503,0.01022,-0.00366,0.0157,0.01008,0.03077,0.05705,-0.02491,0.01601,0.02556,0.01761,0.01986,0.02899,-0.03312,0.02232,2.805E-4,0.01243,0.01962,-0.007118,0.02664,0.02827,0.008094,0.001542,0.04005,0.01182,0.03593,0.06104),.Dim=c(4,24)),

d=c(0.124,0.3916,0.4124,0.2867,0.1916,0.7226,0.172,0.4858,0.5469,0.223,0.1386,0.4668,0.165,0.04791,0.2253,0.4958,0.5687,0.5744,0.1981,0.1164,0.4274,0.1881,0.09398,0.1635,0.2897,0.2129,0.237,0.5067,0.4667,0.2007,0.3956,0.1562,0.4078,0.4284,0.5545,0.2265,0.3225,0.2551,0.1387,0.1088,0.5648,0.7699,0.8511,0.7296,0.1884,0.6177,0.4865,0.3023,0.236,0.2014,0.1648,0.2967,0.3874,0.5928,0.2131,0.3042,0.2539,0.3913,0.3746,0.2614,0.7238,0.7038,0.4944,0.4498,0.9335,0.8746,0.9951,0.9485,0.5139,0.374,0.205,0.7787,0.9939,0.4693,0.2834,0.9753,0.3937,0.05375,0.343,0.2945,0.3799,0.9843,0.5737,0.4589,0.8523,0.5611,0.3047,0.4098,0.3749,0.4878,0.712,0.7119,0.843,0.6298,0.5402,0.4952,0.1278,0.4554,0.6059,0.5861,0.9816,0.5039,0.4192,0.4675,0.9335,0.6085,0.3302,0.6439,0.6294,0.4238,0.8227,0.3703,0.4618,0.9683,0.4143,0.1556,0.8935,0.9506,0.8118,0.6819,0.5874,0.0122,0.2475,0.9174,0.3742,0.9253,0.5256,0.255,0.9718,0.8155,0.4834,0.3661,0.4966,0.3404,0.5064,0.4948,0.3826,0.636,0.4543,0.6722,0.2964,0.8431,0.1316,0.9921,0.9531,0.7235,0.9822,0.8272,0.6508,0.6691,0.338,0.7266,0.6584,0.5521,0.7358,0.2972,0.3602,0.6846,0.4813,0.5463,0.8803,0.3078,0.2353,0.5152,0.3451,0.5095,0.5804,0.6115,0.6348,0.4004,0.1421,0.4339,0.3805,0.4703,0.506,0.323,0.4798,0.6062,0.7078,0.451,0.3354,0.3028,0.278,0.3137,0.6056,0.4572,0.2597,0.5122,0.4591,0.8758,0.5304,0.3117,0.5206,0.5613,0.4217,0.6021,0.6158,0.5194,0.1528,0.6535,0.4935,0.5662,0.4274,0.439,0.1344,0.3139,0.6536,0.4471,0.7926,0.8502,0.9802,0.9131,0.7963,0.4297,0.4837,0.4443,0.2678,0.4235,0.401,0.2682,0.3095,0.659,0.5812,0.4881,0.5792,0.4981,0.425,0.2326,0.4333,0.3244,0.7525,0.4297,0.2073,0.825,0.8671,0.7396,0.2527,0.3736,0.1162,0.4012,0.1772,0.1952,0.4868,0.6767,0.727,0.3243,0.6547,0.6288,0.7669,0.623,0.4946,0.4646,0.7799,0.6241,0.2583,0.3171,0.6129,0.9637,0.9399,0.5505,0.6169,0.3865,0.4236,0.4056,0.2751,0.1548,0.2856,0.2603,0.9662,0.7342,0.846,0.4877,0.5032,0.3121),

present=structure(.Data=c(19.0,1.0,0.0,0.0,0.0,0.0,2.0,5.0,2.0,0.0,0.0,0.0,1.0,0.0,0.0,0.0,25.0,0.0,1.0,0.0,0.0,1.0,1.0,1.0,23.0,2.0,0.0,0.0,2.0,0.0,1.0,5.0,6.0,0.0,1.0,0.0,2.0,0.0,0.0,0.0,26.0,0.0,0.0,1.0,2.0,0.0,0.0,0.0,15.0,1.0,0.0,0.0,0.0,1.0,0.0,6.0,4.0,0.0,0.0,1.0,0.0,0.0,0.0,1.0,20.0,0.0,0.0,1.0,1.0,0.0,0.0,0.0,158.0,37.0,8.0,13.0,9.0,5.0,15.0,28.0,29.0,4.0,5.0,0.0,6.0,4.0,4.0,4.0,28.0,0.0,2.0,1.0,0.0,0.0,0.0,1.0,72.0,27.0,11.0,7.0,2.0,2.0,5.0,11.0,10.0,1.0,2.0,0.0,2.0,0.0,0.0,1.0,25.0,0.0,0.0,1.0,0.0,2.0,0.0,0.0,24.0,5.0,0.0,0.0,0.0,0.0,3.0,8.0,7.0,0.0,0.0,1.0,1.0,0.0,0.0,1.0,14.0,0.0,0.0,1.0,1.0,0.0,0.0,0.0,7.0,0.0,0.0,0.0,0.0,0.0,1.0,0.0,2.0,0.0,0.0,1.0,1.0,0.0,0.0,0.0,14.0,0.0,0.0,0.0,1.0,0.0,1.0,0.0,9.0,0.0,0.0,0.0,0.0,0.0,0.0,1.0,2.0,0.0,0.0,1.0,0.0,1.0,0.0,0.0,19.0,0.0,1.0,0.0,1.0,0.0,1.0,1.0,3.0,0.0,0.0,0.0,0.0,0.0,1.0,1.0,2.0,0.0,0.0,1.0,0.0,0.0,0.0,0.0,14.0,0.0,2.0,0.0,0.0,1.0,0.0,1.0,4.0,0.0,0.0,0.0,0.0,0.0,0.0,2.0,2.0,0.0,0.0,1.0,1.0,0.0,0.0,0.0,17.0,0.0,4.0,2.0,1.0,0.0,0.0,0.0,4.0,0.0,0.0,0.0,0.0,0.0,0.0,3.0,10.0,0.0,0.0,1.0,1.0,1.0,0.0,1.0,19.0,0.0,1.0,0.0,1.0,0.0,0.0,0.0,10.0,0.0,0.0,0.0,0.0,0.0,1.0,4.0,5.0,0.0,0.0,0.0,1.0,0.0,0.0,0.0,16.0,0.0,1.0,1.0,0.0,0.0,1.0,1.0,25.0,4.0,0.0,0.0,5.0,0.0,3.0,6.0,12.0,1.0,1.0,0.0,2.0,1.0,0.0,0.0,23.0,0.0,2.0,0.0,0.0,0.0,1.0,0.0,19.0,2.0,0.0,0.0,3.0,0.0,2.0,6.0,5.0,0.0,0.0,0.0,0.0,1.0,0.0,1.0,22.0,0.0,0.0,0.0,2.0,0.0,0.0,0.0,132.0,33.0,9.0,11.0,7.0,3.0,10.0,25.0,18.0,2.0,3.0,0.0,3.0,1.0,1.0,0.0,22.0,0.0,1.0,1.0,1.0,1.0,0.0,0.0,40.0,10.0,2.0,3.0,2.0,2.0,3.0,9.0,12.0,1.0,3.0,0.0,1.0,0.0,1.0,1.0,17.0,1.0,0.0,0.0,0.0,0.0,0.0,0.0,43.0,17.0,3.0,2.0,1.0,1.0,3.0,5.0,10.0,0.0,1.0,1.0,0.0,0.0,3.0,2.0,15.0,0.0,0.0,1.0,0.0,0.0,1.0,0.0,24.0,2.0,0.0,0.0,2.0,0.0,1.0,2.0,11.0,1.0,0.0,0.0,0.0,0.0,0.0,0.0,11.0,0.0,0.0,0.0,0.0,0.0,1.0,0.0,6.0,3.0,0.0,0.0,1.0,0.0,1.0,1.0,7.0,0.0,0.0,0.0,0.0,0.0,0.0,1.0,18.0,0.0,1.0,0.0,1.0,0.0,0.0,1.0,5.0,0.0,0.0,0.0,1.0,0.0,0.0,2.0,7.0,0.0,0.0,0.0,2.0,0.0,0.0,0.0,27.0,0.0,2.0,0.0,2.0,0.0,0.0,0.0,1.0,0.0,0.0,0.0,0.0,0.0,0.0,0.0,2.0,0.0,0.0,0.0,0.0,0.0,0.0,0.0,14.0,0.0,1.0,0.0,1.0,0.0,1.0,0.0,4.0,0.0,0.0,0.0,0.0,0.0,0.0,1.0,4.0,0.0,0.0,1.0,1.0,0.0,0.0,0.0,20.0,0.0,1.0,0.0,1.0,0.0,1.0,0.0,3.0,0.0,0.0,0.0,0.0,0.0,0.0,4.0,3.0,0.0,0.0,0.0,0.0,0.0,0.0,0.0,17.0,0.0,1.0,0.0,0.0,0.0,1.0,2.0,3.0,1.0,0.0,0.0,0.0,0.0,0.0,1.0,2.0,0.0,0.0,0.0,0.0,0.0,1.0,0.0,24.0,0.0,0.0,0.0,0.0,0.0,0.0,1.0,12.0,1.0,0.0,0.0,1.0,1.0,1.0,1.0,5.0,0.0,0.0,0.0,1.0,0.0,0.0,1.0,25.0,0.0,3.0,3.0,1.0,0.0,1.0,1.0,15.0,1.0,0.0,0.0,1.0,0.0,1.0,5.0,6.0,0.0,0.0,0.0,1.0,0.0,0.0,2.0,17.0,0.0,0.0,1.0,0.0,0.0,1.0,1.0,137.0,33.0,9.0,6.0,9.0,6.0,8.0,19.0,10.0,0.0,1.0,0.0,3.0,3.0,1.0,3.0,27.0,0.0,1.0,0.0,2.0,0.0,0.0,1.0,74.0,19.0,5.0,4.0,1.0,2.0,3.0,13.0,5.0,0.0,0.0,1.0,2.0,0.0,2.0,2.0,15.0,0.0,1.0,0.0,2.0,0.0,1.0,0.0,44.0,9.0,2.0,0.0,4.0,0.0,0.0,10.0,8.0,0.0,0.0,1.0,0.0,0.0,2.0,1.0,23.0,0.0,0.0,1.0,0.0,0.0,0.0,1.0,32.0,4.0,0.0,0.0,0.0,0.0,1.0,6.0,5.0,0.0,1.0,1.0,0.0,0.0,2.0,0.0,21.0,1.0,2.0,0.0,0.0,0.0,0.0,0.0,4.0,0.0,0.0,0.0,0.0,0.0,0.0,2.0,2.0,0.0,0.0,0.0,1.0,0.0,0.0,0.0,9.0,0.0,0.0,0.0,0.0,0.0,0.0,2.0,6.0,1.0,0.0,0.0,0.0,0.0,1.0,4.0,2.0,0.0,0.0,0.0,0.0,0.0,0.0,0.0,21.0,0.0,4.0,0.0,2.0,0.0,0.0,0.0,1.0,0.0,0.0,0.0,0.0,0.0,0.0,0.0,2.0,0.0,0.0,0.0,0.0,0.0,0.0,0.0,13.0,0.0,0.0,0.0,0.0,0.0,1.0,0.0,5.0,0.0,0.0,0.0,0.0,0.0,0.0,3.0,6.0,0.0,0.0,1.0,1.0,0.0,0.0,0.0,18.0,0.0,1.0,0.0,0.0,0.0,1.0,1.0,6.0,0.0,0.0,0.0,0.0,0.0,0.0,2.0,5.0,0.0,0.0,1.0,2.0,0.0,0.0,0.0,26.0,0.0,1.0,0.0,0.0,0.0,0.0,0.0,18.0,0.0,0.0,0.0,1.0,0.0,1.0,3.0,7.0,0.0,0.0,0.0,3.0,0.0,0.0,0.0,35.0,0.0,2.0,0.0,2.0,1.0,1.0,1.0,14.0,1.0,0.0,0.0,1.0,0.0,3.0,1.0,6.0,0.0,0.0,0.0,1.0,1.0,0.0,0.0,20.0,0.0,1.0,0.0,0.0,2.0,0.0,0.0,80.0,6.0,4.0,2.0,5.0,1.0,9.0,16.0,21.0,1.0,2.0,0.0,3.0,3.0,7.0,2.0,31.0,0.0,1.0,1.0,2.0,1.0,2.0,0.0,194.0,40.0,18.0,14.0,9.0,5.0,12.0,28.0,28.0,1.0,4.0,0.0,6.0,6.0,2.0,2.0,33.0,0.0,0.0,0.0,1.0,0.0,1.0,1.0,50.0,12.0,4.0,2.0,3.0,2.0,0.0,9.0,10.0,0.0,4.0,1.0,1.0,0.0,1.0,0.0,22.0,3.0,1.0,0.0,2.0,0.0,0.0,0.0,52.0,8.0,3.0,2.0,2.0,0.0,0.0,8.0,8.0,1.0,2.0,1.0,2.0,1.0,0.0,2.0,23.0,0.0,0.0,0.0,1.0,0.0,0.0,0.0,44.0,13.0,3.0,0.0,3.0,0.0,2.0,4.0,6.0,0.0,0.0,0.0,2.0,0.0,0.0,1.0,15.0,0.0,0.0,0.0,1.0,0.0,0.0,0.0,7.0,0.0,0.0,0.0,1.0,0.0,1.0,2.0,2.0,0.0,0.0,0.0,0.0,0.0,0.0,0.0,18.0,1.0,0.0,0.0,0.0,0.0,1.0,0.0,3.0,0.0,0.0,0.0,0.0,0.0,1.0,1.0,2.0,0.0,0.0,0.0,0.0,1.0,0.0,0.0,17.0,0.0,0.0,0.0,0.0,0.0,0.0,0.0,3.0,0.0,0.0,0.0,1.0,0.0,1.0,1.0,1.0,0.0,0.0,1.0,0.0,0.0,0.0,0.0,29.0,0.0,3.0,0.0,0.0,0.0,0.0,0.0,3.0,0.0,0.0,0.0,0.0,0.0,0.0,1.0,1.0,0.0,0.0,0.0,0.0,0.0,0.0,0.0,11.0,0.0,0.0,0.0,0.0,0.0,0.0,0.0,2.0,0.0,0.0,0.0,0.0,0.0,1.0,2.0,1.0,0.0,0.0,0.0,0.0,0.0,0.0,0.0,21.0,0.0,1.0,0.0,0.0,0.0,0.0,0.0,7.0,0.0,0.0,0.0,1.0,0.0,1.0,3.0,5.0,0.0,0.0,0.0,0.0,0.0,0.0,1.0,23.0,0.0,0.0,0.0,1.0,0.0,0.0,0.0,30.0,2.0,0.0,0.0,1.0,0.0,3.0,10.0,8.0,0.0,0.0,1.0,0.0,3.0,0.0,1.0,22.0,0.0,0.0,3.0,1.0,0.0,0.0,1.0,166.0,37.0,17.0,11.0,13.0,4.0,12.0,28.0,28.0,2.0,3.0,1.0,7.0,5.0,1.0,2.0,36.0,0.0,2.0,1.0,1.0,1.0,2.0,2.0,104.0,33.0,13.0,12.0,5.0,4.0,6.0,19.0,13.0,1.0,3.0,0.0,4.0,2.0,1.0,1.0,25.0,1.0,0.0,2.0,0.0,1.0,0.0,0.0,18.0,2.0,3.0,0.0,1.0,2.0,2.0,5.0,4.0,0.0,1.0,0.0,0.0,0.0,0.0,0.0,27.0,1.0,0.0,2.0,0.0,0.0,0.0,0.0,24.0,3.0,0.0,0.0,1.0,0.0,1.0,4.0,3.0,0.0,1.0,0.0,0.0,1.0,1.0,0.0,23.0,2.0,0.0,1.0,0.0,1.0,0.0,0.0,6.0,1.0,0.0,0.0,0.0,0.0,1.0,3.0,2.0,0.0,0.0,1.0,0.0,0.0,0.0,1.0,9.0,0.0,0.0,0.0,1.0,0.0,2.0,0.0,2.0,0.0,0.0,0.0,0.0,0.0,0.0,0.0,3.0,0.0,0.0,0.0,0.0,0.0,1.0,0.0,20.0,0.0,1.0,0.0,0.0,0.0,0.0,1.0,6.0,0.0,0.0,0.0,0.0,0.0,0.0,1.0,4.0,0.0,0.0,1.0,2.0,0.0,0.0,0.0,16.0,0.0,0.0,1.0,0.0,0.0,1.0,1.0,5.0,0.0,0.0,0.0,0.0,0.0,0.0,2.0,5.0,0.0,0.0,1.0,3.0,0.0,0.0,0.0,22.0,0.0,2.0,0.0,0.0,0.0,1.0,0.0,3.0,0.0,0.0,0.0,0.0,0.0,0.0,2.0,4.0,0.0,0.0,2.0,0.0,0.0,0.0,1.0,17.0,0.0,1.0,0.0,2.0,0.0,0.0,0.0,7.0,0.0,0.0,0.0,0.0,0.0,0.0,2.0,2.0,0.0,0.0,1.0,2.0,0.0,0.0,0.0,17.0,1.0,2.0,0.0,0.0,0.0,0.0,1.0,28.0,3.0,1.0,0.0,2.0,0.0,2.0,7.0,8.0,0.0,0.0,0.0,1.0,0.0,0.0,1.0,24.0,0.0,1.0,2.0,1.0,1.0,0.0,1.0,35.0,6.0,2.0,1.0,2.0,1.0,2.0,6.0,10.0,0.0,1.0,0.0,2.0,0.0,1.0,2.0,15.0,1.0,0.0,1.0,1.0,0.0,0.0,0.0,52.0,13.0,7.0,4.0,2.0,4.0,2.0,9.0,11.0,1.0,3.0,1.0,1.0,0.0,0.0,1.0,18.0,0.0,1.0,2.0,0.0,2.0,0.0,0.0,66.0,19.0,10.0,8.0,2.0,0.0,7.0,13.0,4.0,2.0,2.0,0.0,2.0,0.0,0.0,0.0,22.0,1.0,0.0,1.0,1.0,0.0,0.0,0.0,26.0,5.0,2.0,1.0,0.0,0.0,0.0,5.0,8.0,0.0,0.0,1.0,0.0,0.0,1.0,1.0,31.0,1.0,0.0,1.0,0.0,1.0,0.0,0.0,18.0,2.0,0.0,0.0,2.0,0.0,2.0,3.0,6.0,0.0,0.0,1.0,0.0,0.0,0.0,0.0,20.0,0.0,2.0,2.0,0.0,1.0,0.0,0.0,16.0,1.0,0.0,0.0,4.0,0.0,1.0,2.0,7.0,0.0,0.0,0.0,1.0,1.0,0.0,0.0,13.0,0.0,1.0,0.0,0.0,0.0,0.0,0.0,8.0,0.0,0.0,0.0,1.0,0.0,2.0,2.0,3.0,0.0,0.0,1.0,1.0,0.0,0.0,0.0,14.0,0.0,1.0,0.0,0.0,1.0,0.0,1.0,4.0,0.0,0.0,0.0,0.0,0.0,0.0,2.0,2.0,0.0,0.0,1.0,0.0,0.0,0.0,0.0,11.0,0.0,1.0,0.0,1.0,0.0,0.0,0.0,10.0,0.0,0.0,0.0,2.0,0.0,0.0,3.0,6.0,0.0,0.0,1.0,1.0,0.0,0.0,0.0,15.0,0.0,1.0,0.0,2.0,0.0,0.0,0.0,16.0,2.0,0.0,0.0,2.0,0.0,1.0,6.0,11.0,1.0,1.0,0.0,2.0,0.0,0.0,1.0,19.0,0.0,0.0,2.0,1.0,0.0,0.0,1.0,100.0,28.0,5.0,3.0,6.0,1.0,3.0,15.0,17.0,2.0,3.0,0.0,5.0,3.0,0.0,3.0,40.0,1.0,0.0,1.0,0.0,2.0,0.0,0.0,19.0,1.0,0.0,0.0,2.0,0.0,1.0,5.0,11.0,0.0,0.0,0.0,2.0,0.0,0.0,2.0,24.0,1.0,1.0,0.0,2.0,0.0,0.0,2.0,15.0,1.0,0.0,0.0,2.0,0.0,1.0,2.0,3.0,0.0,0.0,0.0,1.0,0.0,0.0,0.0,17.0,0.0,0.0,1.0,1.0,0.0,0.0,1.0,66.0,16.0,3.0,0.0,4.0,2.0,5.0,16.0,11.0,0.0,3.0,0.0,2.0,1.0,7.0,3.0,29.0,0.0,0.0,1.0,2.0,0.0,0.0,1.0,110.0,27.0,11.0,9.0,3.0,1.0,4.0,20.0,11.0,0.0,1.0,0.0,1.0,1.0,0.0,1.0,27.0,1.0,0.0,1.0,0.0,1.0,0.0,0.0,44.0,12.0,0.0,1.0,1.0,0.0,5.0,5.0,12.0,0.0,0.0,2.0,0.0,0.0,1.0,0.0,22.0,0.0,1.0,1.0,2.0,1.0,1.0,1.0,12.0,0.0,0.0,0.0,0.0,0.0,1.0,3.0,8.0,0.0,0.0,1.0,1.0,1.0,0.0,0.0,28.0,0.0,1.0,1.0,2.0,0.0,3.0,0.0,32.0,4.0,0.0,0.0,2.0,0.0,1.0,4.0,14.0,0.0,0.0,0.0,1.0,0.0,0.0,0.0,30.0,0.0,1.0,1.0,2.0,0.0,2.0,1.0,15.0,4.0,0.0,0.0,3.0,0.0,1.0,3.0,3.0,0.0,0.0,0.0,4.0,0.0,0.0,0.0,23.0,0.0,2.0,1.0,0.0,1.0,0.0,1.0,106.0,25.0,11.0,8.0,5.0,2.0,6.0,20.0,16.0,2.0,3.0,1.0,1.0,2.0,0.0,2.0,39.0,0.0,1.0,1.0,2.0,0.0,1.0,1.0,94.0,32.0,6.0,6.0,7.0,2.0,11.0,23.0,13.0,1.0,5.0,0.0,1.0,6.0,2.0,2.0,36.0,1.0,0.0,1.0,1.0,0.0,2.0,1.0,19.0,6.0,0.0,0.0,0.0,0.0,0.0,4.0,12.0,1.0,2.0,1.0,2.0,0.0,0.0,1.0,34.0,0.0,1.0,1.0,0.0,1.0,0.0,0.0,47.0,9.0,2.0,0.0,2.0,0.0,6.0,13.0,12.0,0.0,0.0,2.0,2.0,1.0,0.0,4.0,31.0,1.0,1.0,0.0,0.0,0.0,1.0,0.0,8.0,0.0,1.0,0.0,0.0,0.0,0.0,2.0,7.0,0.0,0.0,1.0,3.0,0.0,0.0,0.0,29.0,0.0,0.0,1.0,0.0,0.0,0.0,1.0,5.0,0.0,0.0,0.0,0.0,0.0,0.0,2.0,5.0,0.0,0.0,1.0,1.0,0.0,0.0,0.0,28.0,0.0,2.0,0.0,0.0,0.0,0.0,0.0,4.0,0.0,0.0,0.0,0.0,0.0,0.0,1.0,3.0,0.0,0.0,0.0,1.0,0.0,0.0,0.0,19.0,0.0,0.0,0.0,1.0,0.0,1.0,1.0,7.0,1.0,0.0,0.0,0.0,0.0,1.0,3.0,7.0,0.0,0.0,1.0,1.0,0.0,0.0,0.0,30.0,0.0,3.0,0.0,1.0,0.0,1.0,1.0,27.0,2.0,0.0,0.0,3.0,0.0,2.0,9.0,6.0,0.0,0.0,0.0,3.0,1.0,0.0,1.0,33.0,0.0,0.0,1.0,1.0,1.0,3.0,1.0,24.0,1.0,1.0,2.0,3.0,0.0,0.0,9.0,10.0,0.0,1.0,0.0,2.0,1.0,1.0,2.0,29.0,0.0,0.0,0.0,1.0,0.0,0.0,0.0,80.0,16.0,12.0,5.0,4.0,2.0,2.0,11.0,11.0,2.0,1.0,1.0,2.0,1.0,0.0,0.0,18.0,2.0,0.0,0.0,1.0,2.0,0.0,0.0,7.0,0.0,1.0,0.0,1.0,0.0,0.0,6.0,3.0,0.0,0.0,0.0,0.0,0.0,0.0,0.0,28.0,1.0,1.0,1.0,1.0,1.0,0.0,0.0,8.0,0.0,0.0,0.0,0.0,0.0,1.0,1.0,8.0,0.0,0.0,0.0,1.0,0.0,1.0,0.0,22.0,0.0,1.0,0.0,2.0,2.0,0.0,2.0,1.0,0.0,0.0,0.0,0.0,0.0,0.0,1.0,3.0,0.0,0.0,1.0,1.0,0.0,0.0,0.0,34.0,0.0,1.0,0.0,1.0,0.0,1.0,0.0,5.0,0.0,0.0,0.0,0.0,0.0,1.0,1.0,4.0,0.0,0.0,0.0,1.0,0.0,0.0,0.0,28.0,0.0,2.0,0.0,1.0,0.0,1.0,0.0,12.0,2.0,0.0,0.0,1.0,0.0,2.0,3.0,7.0,0.0,0.0,1.0,1.0,0.0,1.0,0.0,28.0,0.0,0.0,0.0,1.0,1.0,2.0,1.0,62.0,9.0,7.0,3.0,2.0,4.0,5.0,11.0,23.0,2.0,1.0,0.0,3.0,2.0,2.0,3.0,23.0,1.0,0.0,1.0,2.0,0.0,1.0,2.0,258.0,44.0,19.0,14.0,14.0,11.0,20.0,37.0,75.0,6.0,2.0,0.0,12.0,12.0,0.0,7.0,61.0,0.0,2.0,1.0,4.0,0.0,3.0,4.0,13.0,0.0,0.0,0.0,0.0,0.0,0.0,5.0,5.0,0.0,0.0,0.0,2.0,0.0,0.0,0.0,20.0,0.0,2.0,1.0,1.0,0.0,1.0,1.0,2.0,0.0,0.0,0.0,1.0,0.0,1.0,1.0,5.0,0.0,0.0,0.0,1.0,0.0,0.0,0.0,28.0,0.0,1.0,0.0,2.0,0.0,1.0,0.0,1.0,0.0,0.0,0.0,0.0,0.0,0.0,1.0,5.0,0.0,0.0,1.0,1.0,0.0,0.0,0.0,19.0,0.0,0.0,0.0,0.0,0.0,1.0,0.0,23.0,1.0,1.0,0.0,2.0,1.0,2.0,6.0,9.0,0.0,0.0,0.0,2.0,2.0,0.0,1.0,24.0,1.0,2.0,1.0,2.0,0.0,1.0,1.0,46.0,9.0,3.0,4.0,0.0,1.0,2.0,10.0,4.0,0.0,1.0,0.0,0.0,0.0,2.0,0.0,22.0,1.0,0.0,0.0,1.0,2.0,1.0,0.0,16.0,5.0,2.0,2.0,0.0,1.0,0.0,4.0,9.0,1.0,3.0,0.0,1.0,0.0,2.0,0.0,23.0,0.0,0.0,1.0,1.0,2.0,0.0,0.0,22.0,3.0,0.0,1.0,0.0,0.0,1.0,2.0,10.0,1.0,2.0,0.0,1.0,0.0,2.0,1.0,32.0,0.0,1.0,0.0,3.0,2.0,1.0,0.0,34.0,7.0,1.0,1.0,5.0,0.0,5.0,7.0,12.0,1.0,1.0,0.0,0.0,0.0,2.0,1.0,35.0,0.0,1.0,1.0,2.0,1.0,1.0,1.0,3.0,1.0,0.0,0.0,0.0,0.0,1.0,0.0,2.0,0.0,0.0,1.0,0.0,0.0,0.0,0.0,27.0,1.0,1.0,0.0,2.0,1.0,1.0,0.0,1.0,0.0,0.0,0.0,0.0,0.0,0.0,3.0,2.0,0.0,0.0,1.0,2.0,0.0,0.0,0.0,19.0,0.0,0.0,1.0,2.0,1.0,0.0,0.0,2.0,0.0,0.0,0.0,0.0,0.0,0.0,1.0,7.0,0.0,0.0,0.0,1.0,0.0,0.0,0.0,21.0,0.0,0.0,0.0,1.0,1.0,1.0,1.0,10.0,1.0,0.0,0.0,0.0,0.0,2.0,3.0,11.0,0.0,0.0,3.0,1.0,0.0,0.0,0.0,14.0,0.0,1.0,1.0,1.0,0.0,1.0,1.0,24.0,4.0,2.0,2.0,2.0,0.0,0.0,3.0,4.0,2.0,0.0,0.0,1.0,0.0,0.0,0.0,34.0,1.0,0.0,2.0,1.0,0.0,0.0,1.0,9.0,1.0,0.0,0.0,0.0,0.0,2.0,2.0,3.0,0.0,0.0,0.0,0.0,0.0,0.0,0.0,33.0,0.0,1.0,1.0,1.0,1.0,1.0,1.0,6.0,0.0,0.0,0.0,0.0,0.0,2.0,1.0,9.0,0.0,0.0,0.0,2.0,0.0,0.0,0.0,35.0,0.0,0.0,0.0,1.0,0.0,1.0,0.0,8.0,2.0,0.0,0.0,1.0,0.0,2.0,1.0,4.0,0.0,0.0,1.0,1.0,0.0,0.0,0.0,32.0,0.0,0.0,0.0,2.0,0.0,1.0,2.0,33.0,4.0,3.0,0.0,3.0,1.0,3.0,8.0,14.0,1.0,1.0,0.0,0.0,0.0,3.0,1.0,28.0,0.0,1.0,0.0,1.0,0.0,1.0,1.0,81.0,29.0,5.0,6.0,4.0,2.0,4.0,17.0,5.0,0.0,2.0,0.0,2.0,1.0,1.0,0.0,29.0,1.0,0.0,1.0,2.0,1.0,2.0,1.0,46.0,11.0,3.0,5.0,3.0,1.0,2.0,11.0,17.0,1.0,3.0,0.0,2.0,0.0,2.0,0.0,40.0,1.0,0.0,0.0,0.0,1.0,1.0,0.0,68.0,16.0,2.0,2.0,3.0,2.0,5.0,14.0,15.0,0.0,1.0,1.0,2.0,1.0,0.0,0.0,27.0,0.0,1.0,0.0,0.0,0.0,1.0,1.0,31.0,3.0,0.0,0.0,4.0,1.0,3.0,6.0,14.0,1.0,0.0,1.0,1.0,1.0,0.0,1.0,21.0,0.0,2.0,0.0,1.0,0.0,0.0,3.0,9.0,0.0,0.0,0.0,0.0,0.0,1.0,1.0,11.0,0.0,0.0,0.0,1.0,0.0,0.0,0.0,29.0,0.0,0.0,1.0,2.0,0.0,2.0,1.0,20.0,5.0,1.0,1.0,0.0,1.0,1.0,5.0,7.0,0.0,0.0,0.0,0.0,0.0,1.0,1.0,25.0,0.0,0.0,0.0,1.0,1.0,0.0,0.0,4.0,1.0,0.0,0.0,0.0,0.0,0.0,1.0,3.0,0.0,0.0,1.0,0.0,0.0,0.0,0.0,32.0,0.0,1.0,0.0,1.0,0.0,1.0,0.0,3.0,0.0,0.0,0.0,0.0,0.0,1.0,2.0,5.0,0.0,0.0,0.0,0.0,0.0,0.0,0.0,22.0,0.0,1.0,0.0,0.0,0.0,0.0,0.0,9.0,1.0,0.0,0.0,1.0,0.0,1.0,4.0,8.0,0.0,0.0,0.0,2.0,0.0,0.0,0.0,31.0,0.0,1.0,1.0,0.0,0.0,0.0,0.0,23.0,2.0,2.0,0.0,1.0,0.0,2.0,10.0,7.0,1.0,0.0,0.0,1.0,1.0,1.0,0.0,25.0,0.0,0.0,1.0,1.0,0.0,1.0,1.0,35.0,10.0,0.0,0.0,1.0,1.0,5.0,5.0,15.0,1.0,0.0,0.0,3.0,0.0,0.0,0.0,35.0,0.0,1.0,2.0,2.0,0.0,0.0,1.0,8.0,0.0,0.0,0.0,0.0,0.0,2.0,1.0,2.0,0.0,0.0,0.0,0.0,1.0,1.0,0.0,25.0,0.0,1.0,1.0,1.0,0.0,0.0,0.0,8.0,0.0,0.0,0.0,0.0,0.0,2.0,2.0,9.0,0.0,0.0,0.0,2.0,0.0,0.0,0.0,33.0,0.0,1.0,0.0,2.0,1.0,2.0,0.0,9.0,0.0,0.0,0.0,0.0,0.0,1.0,4.0,10.0,0.0,0.0,0.0,0.0,0.0,0.0,0.0,33.0,0.0,3.0,0.0,1.0,0.0,0.0,2.0,5.0,0.0,0.0,0.0,0.0,0.0,0.0,1.0,3.0,0.0,0.0,1.0,0.0,0.0,0.0,0.0,16.0,0.0,1.0,0.0,1.0,0.0,1.0,0.0,6.0,0.0,0.0,0.0,1.0,0.0,1.0,2.0,6.0,0.0,0.0,0.0,1.0,0.0,0.0,1.0,23.0,0.0,0.0,0.0,2.0,0.0,4.0,0.0,24.0,3.0,0.0,0.0,1.0,0.0,5.0,6.0,8.0,0.0,0.0,0.0,2.0,0.0,0.0,0.0,38.0,1.0,1.0,1.0,2.0,1.0,1.0,2.0,57.0,9.0,0.0,1.0,2.0,0.0,3.0,15.0,16.0,0.0,0.0,1.0,3.0,0.0,1.0,1.0,40.0,0.0,0.0,2.0,1.0,1.0,2.0,1.0,69.0,8.0,1.0,2.0,5.0,0.0,8.0,16.0,15.0,0.0,1.0,1.0,3.0,2.0,3.0,1.0,30.0,0.0,0.0,0.0,2.0,0.0,1.0,2.0,107.0,28.0,15.0,12.0,4.0,3.0,6.0,15.0,20.0,0.0,1.0,1.0,3.0,3.0,1.0,1.0,42.0,0.0,1.0,0.0,1.0,0.0,1.0,2.0,45.0,9.0,7.0,2.0,1.0,1.0,2.0,7.0,8.0,0.0,0.0,0.0,0.0,0.0,3.0,0.0,40.0,1.0,0.0,2.0,1.0,0.0,0.0,0.0,30.0,6.0,0.0,2.0,0.0,0.0,2.0,5.0,6.0,1.0,0.0,0.0,1.0,0.0,0.0,1.0,20.0,2.0,0.0,0.0,1.0,1.0,0.0,0.0,111.0,24.0,11.0,6.0,3.0,1.0,9.0,18.0,21.0,1.0,2.0,1.0,2.0,2.0,1.0,0.0,27.0,0.0,1.0,1.0,3.0,2.0,0.0,3.0,4.0,0.0,0.0,0.0,1.0,0.0,0.0,1.0,6.0,0.0,0.0,0.0,0.0,0.0,0.0,0.0,28.0,0.0,0.0,0.0,3.0,0.0,0.0,1.0,6.0,0.0,0.0,0.0,0.0,0.0,0.0,1.0,5.0,0.0,0.0,0.0,0.0,0.0,0.0,0.0,33.0,0.0,1.0,0.0,2.0,0.0,2.0,1.0,1.0,0.0,0.0,0.0,0.0,0.0,0.0,1.0,2.0,0.0,0.0,0.0,1.0,0.0,0.0,0.0,25.0,0.0,2.0,0.0,3.0,0.0,1.0,1.0,19.0,0.0,0.0,0.0,1.0,0.0,1.0,4.0,14.0,0.0,0.0,0.0,2.0,1.0,0.0,0.0,27.0,0.0,0.0,0.0,4.0,0.0,1.0,0.0,6.0,0.0,0.0,0.0,0.0,0.0,1.0,2.0,7.0,0.0,0.0,2.0,1.0,1.0,0.0,0.0,30.0,0.0,2.0,1.0,1.0,0.0,1.0,2.0,238.0,43.0,20.0,14.0,13.0,10.0,19.0,34.0,63.0,6.0,4.0,0.0,8.0,12.0,0.0,4.0,72.0,1.0,0.0,3.0,4.0,2.0,0.0,1.0,63.0,14.0,7.0,3.0,1.0,1.0,2.0,13.0,6.0,1.0,1.0,0.0,1.0,0.0,0.0,0.0,21.0,0.0,0.0,0.0,0.0,0.0,0.0,0.0,73.0,6.0,5.0,6.0,2.0,4.0,5.0,14.0,20.0,1.0,1.0,0.0,2.0,1.0,1.0,2.0,22.0,1.0,0.0,0.0,0.0,1.0,1.0,0.0,21.0,3.0,0.0,0.0,1.0,1.0,4.0,3.0,10.0,0.0,0.0,1.0,0.0,0.0,0.0,0.0,35.0,0.0,2.0,0.0,1.0,1.0,0.0,2.0,16.0,1.0,0.0,0.0,1.0,0.0,3.0,4.0,12.0,0.0,0.0,1.0,1.0,2.0,0.0,0.0,37.0,0.0,2.0,1.0,2.0,0.0,1.0,3.0,3.0,0.0,0.0,0.0,0.0,0.0,0.0,2.0,6.0,0.0,0.0,1.0,1.0,1.0,0.0,0.0,30.0,0.0,0.0,0.0,1.0,0.0,1.0,2.0,7.0,0.0,0.0,0.0,0.0,0.0,0.0,1.0,5.0,0.0,0.0,0.0,1.0,0.0,0.0,0.0,26.0,0.0,2.0,0.0,1.0,0.0,0.0,1.0,5.0,0.0,0.0,0.0,0.0,0.0,1.0,1.0,3.0,0.0,0.0,3.0,0.0,0.0,0.0,0.0,19.0,0.0,1.0,0.0,2.0,0.0,2.0,0.0,6.0,0.0,0.0,0.0,0.0,0.0,0.0,0.0,6.0,0.0,0.0,0.0,1.0,1.0,0.0,0.0,30.0,0.0,1.0,0.0,2.0,0.0,1.0,2.0,5.0,0.0,0.0,0.0,0.0,0.0,0.0,2.0,4.0,0.0,0.0,1.0,2.0,0.0,0.0,0.0,35.0,0.0,3.0,1.0,1.0,0.0,1.0,0.0,12.0,1.0,0.0,0.0,1.0,0.0,3.0,7.0,8.0,1.0,0.0,0.0,3.0,0.0,1.0,0.0,36.0,1.0,1.0,1.0,0.0,0.0,0.0,1.0,31.0,6.0,2.0,0.0,2.0,0.0,3.0,8.0,9.0,1.0,0.0,0.0,2.0,3.0,3.0,1.0,29.0,0.0,1.0,1.0,3.0,1.0,2.0,1.0,29.0,8.0,0.0,1.0,0.0,1.0,1.0,10.0,3.0,1.0,0.0,0.0,0.0,1.0,1.0,0.0,33.0,2.0,0.0,0.0,0.0,0.0,0.0,0.0,44.0,16.0,5.0,4.0,0.0,0.0,2.0,11.0,9.0,0.0,0.0,0.0,2.0,1.0,1.0,0.0,28.0,0.0,0.0,0.0,0.0,1.0,0.0,0.0,73.0,10.0,4.0,9.0,3.0,1.0,9.0,18.0,28.0,3.0,4.0,0.0,2.0,1.0,6.0,5.0,41.0,2.0,3.0,1.0,1.0,0.0,4.0,1.0,37.0,4.0,1.0,0.0,2.0,0.0,4.0,3.0,14.0,0.0,0.0,0.0,1.0,1.0,1.0,0.0,31.0,0.0,2.0,2.0,2.0,0.0,1.0,4.0,34.0,1.0,0.0,1.0,1.0,0.0,2.0,7.0,13.0,0.0,0.0,0.0,2.0,1.0,0.0,0.0,33.0,0.0,0.0,0.0,4.0,1.0,0.0,1.0,9.0,0.0,0.0,0.0,0.0,0.0,1.0,2.0,3.0,0.0,0.0,0.0,1.0,0.0,0.0,1.0,25.0,0.0,0.0,0.0,2.0,0.0,0.0,1.0,3.0,0.0,0.0,0.0,0.0,0.0,1.0,1.0,4.0,0.0,0.0,0.0,0.0,0.0,0.0,0.0,32.0,0.0,1.0,0.0,1.0,0.0,2.0,0.0,9.0,1.0,0.0,0.0,0.0,0.0,1.0,3.0,8.0,0.0,0.0,0.0,2.0,0.0,0.0,0.0,32.0,0.0,1.0,0.0,1.0,1.0,1.0,2.0,5.0,0.0,0.0,0.0,0.0,0.0,1.0,2.0,1.0,0.0,0.0,1.0,0.0,0.0,0.0,0.0,25.0,0.0,0.0,1.0,0.0,0.0,0.0,1.0,12.0,2.0,0.0,0.0,1.0,0.0,2.0,5.0,6.0,0.0,0.0,0.0,2.0,0.0,2.0,0.0,33.0,1.0,1.0,1.0,2.0,0.0,0.0,1.0,58.0,7.0,2.0,1.0,1.0,0.0,4.0,11.0,18.0,1.0,0.0,0.0,4.0,2.0,1.0,1.0,26.0,0.0,0.0,0.0,0.0,0.0,0.0,0.0,44.0,14.0,6.0,3.0,0.0,2.0,3.0,10.0,5.0,0.0,1.0,1.0,1.0,0.0,0.0,0.0,32.0,2.0,0.0,1.0,1.0,1.0,0.0,0.0,29.0,2.0,3.0,2.0,0.0,0.0,1.0,12.0,6.0,0.0,0.0,0.0,0.0,0.0,1.0,0.0,36.0,2.0,0.0,2.0,1.0,1.0,0.0,1.0,95.0,17.0,12.0,8.0,1.0,2.0,4.0,19.0,20.0,2.0,1.0,1.0,1.0,4.0,0.0,3.0,40.0,0.0,1.0,1.0,1.0,1.0,0.0,2.0,32.0,2.0,0.0,1.0,3.0,0.0,4.0,7.0,10.0,0.0,1.0,2.0,2.0,0.0,0.0,0.0,34.0,1.0,2.0,1.0,2.0,0.0,0.0,4.0,2.0,0.0,0.0,0.0,0.0,0.0,0.0,3.0,2.0,0.0,0.0,0.0,2.0,0.0,0.0,0.0,32.0,0.0,1.0,0.0,2.0,0.0,2.0,0.0,4.0,0.0,0.0,0.0,1.0,0.0,0.0,3.0,7.0,0.0,0.0,2.0,1.0,0.0,0.0,0.0,30.0,0.0,1.0,1.0,2.0,0.0,0.0,2.0,11.0,1.0,0.0,0.0,0.0,0.0,2.0,2.0,7.0,0.0,0.0,1.0,2.0,0.0,1.0,0.0,28.0,0.0,2.0,1.0,1.0,0.0,1.0,2.0,15.0,1.0,1.0,0.0,3.0,0.0,2.0,3.0,9.0,0.0,0.0,0.0,2.0,0.0,0.0,0.0,28.0,0.0,1.0,0.0,1.0,0.0,2.0,1.0,22.0,4.0,1.0,1.0,0.0,0.0,4.0,5.0,7.0,0.0,0.0,0.0,1.0,0.0,2.0,0.0,29.0,1.0,1.0,1.0,2.0,0.0,1.0,1.0,99.0,32.0,9.0,6.0,2.0,2.0,5.0,16.0,13.0,0.0,0.0,0.0,1.0,1.0,0.0,1.0,23.0,0.0,0.0,0.0,1.0,3.0,0.0,0.0,27.0,5.0,6.0,3.0,1.0,0.0,1.0,7.0,7.0,1.0,0.0,0.0,0.0,0.0,1.0,1.0,37.0,1.0,0.0,0.0,1.0,1.0,0.0,1.0,35.0,9.0,2.0,2.0,0.0,2.0,1.0,4.0,10.0,0.0,1.0,1.0,1.0,0.0,2.0,1.0,37.0,2.0,0.0,2.0,2.0,1.0,1.0,0.0,30.0,5.0,1.0,1.0,0.0,0.0,4.0,6.0,10.0,1.0,1.0,1.0,0.0,0.0,1.0,1.0,24.0,0.0,2.0,0.0,2.0,0.0,1.0,1.0,3.0,0.0,0.0,0.0,0.0,0.0,0.0,0.0,2.0,0.0,0.0,1.0,0.0,0.0,0.0,0.0,25.0,0.0,0.0,0.0,0.0,1.0,0.0,0.0,4.0,0.0,0.0,0.0,0.0,0.0,0.0,2.0,4.0,0.0,0.0,0.0,0.0,0.0,1.0,0.0,34.0,0.0,1.0,0.0,2.0,1.0,0.0,2.0,4.0,0.0,0.0,0.0,0.0,0.0,0.0,3.0,1.0,0.0,0.0,1.0,1.0,0.0,0.0,0.0,25.0,0.0,2.0,0.0,0.0,0.0,0.0,0.0,9.0,0.0,0.0,0.0,0.0,0.0,2.0,1.0,4.0,0.0,0.0,0.0,0.0,0.0,0.0,0.0,40.0,0.0,2.0,0.0,2.0,1.0,0.0,0.0,3.0,0.0,0.0,0.0,1.0,0.0,1.0,3.0,10.0,0.0,0.0,2.0,2.0,0.0,0.0,0.0,35.0,0.0,1.0,0.0,1.0,0.0,2.0,0.0,18.0,1.0,0.0,0.0,0.0,0.0,1.0,7.0,10.0,0.0,0.0,1.0,2.0,0.0,1.0,0.0,35.0,0.0,1.0,1.0,2.0,0.0,1.0,1.0,30.0,4.0,1.0,0.0,3.0,0.0,3.0,4.0,8.0,0.0,0.0,1.0,1.0,0.0,0.0,2.0,22.0,1.0,1.0,2.0,1.0,1.0,0.0,2.0,86.0,11.0,6.0,9.0,3.0,1.0,9.0,16.0,27.0,2.0,3.0,0.0,2.0,3.0,4.0,3.0,32.0,0.0,0.0,1.0,3.0,1.0,1.0,3.0,175.0,35.0,14.0,12.0,7.0,7.0,13.0,31.0,33.0,2.0,3.0,0.0,8.0,3.0,3.0,6.0,43.0,0.0,2.0,1.0,1.0,0.0,0.0,5.0,32.0,4.0,0.0,1.0,0.0,2.0,3.0,13.0,12.0,0.0,0.0,0.0,0.0,0.0,1.0,0.0,38.0,1.0,2.0,0.0,1.0,2.0,1.0,0.0,16.0,2.0,0.0,0.0,2.0,0.0,2.0,1.0,11.0,0.0,0.0,0.0,0.0,1.0,0.0,1.0,31.0,0.0,2.0,0.0,2.0,0.0,0.0,2.0,17.0,1.0,0.0,0.0,0.0,0.0,1.0,4.0,8.0,0.0,0.0,1.0,1.0,1.0,0.0,0.0,26.0,0.0,1.0,0.0,1.0,0.0,0.0,2.0,19.0,0.0,0.0,0.0,0.0,1.0,2.0,5.0,7.0,0.0,0.0,0.0,2.0,1.0,0.0,0.0,27.0,0.0,2.0,0.0,2.0,0.0,0.0,1.0,6.0,0.0,0.0,0.0,0.0,0.0,1.0,0.0,2.0,0.0,0.0,1.0,1.0,0.0,0.0,0.0,26.0,0.0,1.0,0.0,2.0,0.0,3.0,1.0,7.0,0.0,0.0,0.0,0.0,0.0,0.0,2.0,4.0,0.0,0.0,0.0,0.0,0.0,0.0,0.0,33.0,0.0,2.0,0.0,0.0,0.0,1.0,0.0,5.0,0.0,0.0,0.0,1.0,0.0,0.0,1.0,8.0,0.0,0.0,3.0,1.0,0.0,0.0,0.0,37.0,0.0,0.0,0.0,2.0,1.0,1.0,1.0,8.0,0.0,0.0,0.0,0.0,0.0,1.0,2.0,6.0,0.0,0.0,0.0,2.0,0.0,1.0,0.0,25.0,0.0,0.0,0.0,1.0,0.0,0.0,1.0,47.0,9.0,1.0,1.0,1.0,2.0,4.0,13.0,11.0,2.0,2.0,1.0,0.0,0.0,1.0,2.0,30.0,1.0,0.0,1.0,2.0,0.0,0.0,0.0,81.0,18.0,11.0,9.0,1.0,3.0,4.0,13.0,8.0,1.0,1.0,1.0,1.0,1.0,3.0,0.0,18.0,1.0,0.0,1.0,1.0,0.0,1.0,1.0,67.0,11.0,3.0,3.0,4.0,1.0,4.0,10.0,9.0,0.0,2.0,0.0,0.0,0.0,1.0,2.0,42.0,1.0,1.0,0.0,0.0,2.0,1.0,1.0,109.0,17.0,3.0,2.0,6.0,0.0,9.0,27.0,32.0,4.0,0.0,0.0,3.0,1.0,3.0,2.0,50.0,0.0,3.0,1.0,1.0,0.0,0.0,2.0,9.0,0.0,0.0,0.0,0.0,0.0,3.0,4.0,9.0,0.0,0.0,1.0,0.0,0.0,0.0,0.0,29.0,0.0,0.0,0.0,2.0,1.0,1.0,3.0,7.0,0.0,0.0,0.0,0.0,0.0,0.0,2.0,4.0,0.0,0.0,3.0,1.0,0.0,0.0,0.0,35.0,0.0,3.0,0.0,1.0,0.0,0.0,2.0,4.0,0.0,0.0,0.0,0.0,0.0,0.0,1.0,6.0,0.0,0.0,1.0,1.0,0.0,0.0,0.0,27.0,0.0,2.0,0.0,1.0,0.0,0.0,1.0,7.0,0.0,0.0,0.0,1.0,0.0,0.0,2.0,4.0,0.0,0.0,0.0,0.0,0.0,0.0,0.0,40.0,0.0,1.0,1.0,0.0,0.0,0.0,0.0,4.0,0.0,0.0,0.0,0.0,0.0,0.0,2.0,6.0,0.0,0.0,0.0,3.0,0.0,0.0,1.0,28.0,0.0,2.0,0.0,1.0,0.0,1.0,0.0,8.0,0.0,0.0,0.0,0.0,0.0,0.0,3.0,7.0,0.0,0.0,0.0,1.0,0.0,0.0,0.0,40.0,0.0,0.0,0.0,1.0,0.0,1.0,0.0,30.0,2.0,0.0,0.0,1.0,0.0,3.0,5.0,9.0,0.0,0.0,1.0,3.0,0.0,0.0,0.0,33.0,1.0,1.0,0.0,1.0,2.0,0.0,0.0,12.0,0.0,1.0,0.0,1.0,0.0,1.0,2.0,4.0,0.0,0.0,0.0,1.0,0.0,1.0,0.0,27.0,1.0,0.0,1.0,0.0,2.0,0.0,1.0,73.0,10.0,5.0,5.0,1.0,0.0,8.0,23.0,10.0,1.0,0.0,0.0,1.0,0.0,4.0,0.0,38.0,0.0,0.0,1.0,1.0,0.0,1.0,1.0,52.0,12.0,7.0,7.0,0.0,0.0,1.0,11.0,5.0,0.0,0.0,0.0,1.0,0.0,0.0,0.0,27.0,1.0,0.0,1.0,1.0,1.0,0.0,0.0,52.0,11.0,3.0,3.0,1.0,3.0,6.0,11.0,17.0,2.0,2.0,0.0,1.0,1.0,3.0,3.0,24.0,1.0,0.0,1.0,1.0,0.0,1.0,1.0,26.0,4.0,0.0,1.0,2.0,0.0,5.0,3.0,12.0,0.0,0.0,1.0,2.0,0.0,1.0,1.0,30.0,0.0,1.0,0.0,0.0,1.0,1.0,1.0,16.0,0.0,0.0,0.0,1.0,0.0,2.0,7.0,8.0,0.0,0.0,2.0,1.0,1.0,0.0,0.0,28.0,0.0,2.0,0.0,0.0,0.0,1.0,2.0,6.0,0.0,0.0,0.0,1.0,0.0,1.0,2.0,4.0,0.0,0.0,1.0,1.0,0.0,0.0,0.0,35.0,0.0,0.0,0.0,1.0,0.0,0.0,2.0,2.0,0.0,0.0,0.0,0.0,0.0,1.0,0.0,4.0,0.0,0.0,0.0,0.0,0.0,0.0,0.0,31.0,0.0,1.0,1.0,1.0,0.0,2.0,1.0,3.0,0.0,0.0,0.0,0.0,0.0,1.0,1.0,2.0,0.0,0.0,0.0,2.0,0.0,0.0,1.0,32.0,0.0,1.0,0.0,3.0,0.0,1.0,1.0,8.0,0.0,0.0,0.0,1.0,0.0,1.0,2.0,15.0,0.0,0.0,1.0,2.0,0.0,0.0,0.0,32.0,0.0,1.0,0.0,4.0,0.0,4.0,0.0,16.0,0.0,0.0,0.0,0.0,0.0,1.0,4.0,12.0,0.0,0.0,0.0,1.0,0.0,0.0,0.0,34.0,0.0,0.0,1.0,1.0,0.0,1.0,2.0,30.0,6.0,0.0,0.0,1.0,0.0,4.0,9.0,11.0,0.0,0.0,0.0,3.0,0.0,1.0,0.0,31.0,0.0,1.0,1.0,1.0,0.0,1.0,4.0,35.0,4.0,2.0,0.0,2.0,0.0,4.0,13.0,15.0,0.0,0.0,0.0,2.0,0.0,3.0,2.0,33.0,0.0,0.0,0.0,1.0,2.0,1.0,1.0,137.0,28.0,7.0,11.0,5.0,3.0,15.0,28.0,25.0,3.0,3.0,0.0,5.0,6.0,6.0,4.0,51.0,0.0,1.0,1.0,1.0,0.0,3.0,4.0,30.0,6.0,3.0,3.0,1.0,1.0,1.0,9.0,4.0,2.0,1.0,0.0,0.0,0.0,2.0,0.0,22.0,1.0,1.0,2.0,1.0,2.0,0.0,0.0,80.0,23.0,4.0,6.0,3.0,0.0,2.0,14.0,12.0,0.0,0.0,1.0,0.0,0.0,0.0,1.0,37.0,0.0,0.0,1.0,2.0,2.0,0.0,0.0,33.0,6.0,0.0,1.0,0.0,0.0,3.0,4.0,11.0,0.0,0.0,2.0,1.0,0.0,0.0,0.0,30.0,0.0,2.0,0.0,2.0,0.0,0.0,1.0,7.0,0.0,0.0,0.0,0.0,0.0,1.0,1.0,9.0,0.0,0.0,1.0,0.0,0.0,2.0,0.0,27.0,0.0,0.0,0.0,1.0,0.0,0.0,0.0,9.0,0.0,0.0,0.0,0.0,0.0,1.0,2.0,7.0,0.0,0.0,0.0,1.0,0.0,0.0,0.0,31.0,0.0,2.0,0.0,2.0,0.0,3.0,2.0,3.0,0.0,0.0,0.0,0.0,0.0,1.0,2.0,4.0,0.0,0.0,0.0,2.0,0.0,0.0,0.0,35.0,0.0,1.0,1.0,3.0,0.0,0.0,0.0,5.0,0.0,0.0,0.0,0.0,0.0,0.0,3.0,4.0,0.0,0.0,2.0,0.0,0.0,0.0,0.0,25.0,0.0,1.0,0.0,1.0,0.0,1.0,0.0,7.0,0.0,0.0,0.0,0.0,0.0,1.0,3.0,7.0,0.0,0.0,0.0,0.0,0.0,0.0,0.0,38.0,1.0,2.0,0.0,1.0,0.0,1.0,1.0,8.0,1.0,0.0,0.0,0.0,0.0,1.0,0.0,7.0,0.0,0.0,0.0,1.0,0.0,0.0,0.0,30.0,0.0,0.0,2.0,0.0,0.0,0.0,2.0,17.0,3.0,0.0,0.0,1.0,1.0,4.0,4.0,9.0,0.0,0.0,0.0,2.0,0.0,0.0,0.0,39.0,0.0,0.0,0.0,2.0,0.0,0.0,2.0,34.0,4.0,1.0,1.0,3.0,1.0,3.0,7.0,5.0,1.0,0.0,0.0,1.0,0.0,0.0,2.0,29.0,0.0,1.0,1.0,0.0,1.0,1.0,1.0,164.0,40.0,15.0,14.0,6.0,7.0,11.0,31.0,34.0,1.0,1.0,0.0,5.0,6.0,3.0,2.0,44.0,0.0,0.0,0.0,1.0,1.0,1.0,6.0,35.0,6.0,2.0,3.0,0.0,0.0,3.0,8.0,6.0,2.0,0.0,0.0,1.0,0.0,2.0,0.0,43.0,2.0,0.0,2.0,1.0,1.0,0.0,0.0,85.0,22.0,12.0,9.0,1.0,2.0,2.0,14.0,9.0,0.0,0.0,0.0,2.0,1.0,0.0,0.0,24.0,1.0,0.0,0.0,0.0,0.0,0.0,1.0,24.0,4.0,1.0,1.0,0.0,2.0,3.0,3.0,11.0,1.0,0.0,1.0,2.0,0.0,0.0,1.0,31.0,0.0,1.0,0.0,2.0,1.0,1.0,1.0,12.0,0.0,0.0,0.0,1.0,0.0,2.0,3.0,7.0,0.0,0.0,1.0,2.0,0.0,0.0,0.0,25.0,0.0,2.0,0.0,2.0,0.0,1.0,1.0,5.0,0.0,0.0,0.0,1.0,0.0,1.0,0.0,6.0,0.0,0.0,2.0,0.0,0.0,0.0,0.0,33.0,0.0,1.0,0.0,0.0,0.0,0.0,2.0,5.0,0.0,0.0,0.0,0.0,1.0,1.0,1.0,6.0,0.0,0.0,0.0,1.0,0.0,0.0,0.0,39.0,0.0,0.0,0.0,1.0,0.0,1.0,2.0,2.0,0.0,0.0,0.0,1.0,0.0,0.0,3.0,6.0,0.0,0.0,3.0,2.0,0.0,0.0,0.0,38.0,0.0,0.0,0.0,3.0,0.0,3.0,1.0,4.0,0.0,0.0,0.0,0.0,0.0,0.0,1.0,9.0,0.0,0.0,0.0,2.0,0.0,1.0,1.0,33.0,0.0,1.0,0.0,2.0,1.0,0.0,1.0,38.0,1.0,1.0,0.0,4.0,0.0,3.0,8.0,12.0,0.0,0.0,0.0,2.0,1.0,0.0,0.0,65.0,0.0,4.0,2.0,2.0,0.0,0.0,4.0,16.0,1.0,0.0,0.0,0.0,0.0,1.0,6.0,4.0,0.0,0.0,0.0,1.0,0.0,0.0,0.0,39.0,0.0,1.0,1.0,0.0,0.0,1.0,3.0,50.0,14.0,8.0,6.0,0.0,0.0,0.0,8.0,7.0,0.0,1.0,0.0,0.0,0.0,0.0,2.0,35.0,1.0,0.0,0.0,1.0,0.0,1.0,1.0,39.0,8.0,3.0,3.0,0.0,0.0,2.0,10.0,13.0,1.0,1.0,0.0,1.0,0.0,0.0,1.0,35.0,1.0,0.0,1.0,2.0,3.0,1.0,0.0,58.0,7.0,2.0,1.0,3.0,0.0,4.0,12.0,14.0,0.0,0.0,1.0,1.0,0.0,3.0,3.0,44.0,0.0,1.0,1.0,2.0,0.0,1.0,0.0,108.0,18.0,9.0,3.0,2.0,2.0,8.0,20.0,19.0,1.0,1.0,1.0,1.0,2.0,1.0,2.0,47.0,0.0,0.0,1.0,0.0,2.0,0.0,3.0,13.0,0.0,0.0,0.0,0.0,0.0,1.0,4.0,8.0,0.0,0.0,0.0,0.0,0.0,0.0,1.0,32.0,0.0,2.0,0.0,1.0,1.0,1.0,3.0,7.0,1.0,0.0,0.0,0.0,0.0,1.0,1.0,5.0,0.0,0.0,1.0,0.0,0.0,0.0,0.0,27.0,1.0,1.0,0.0,2.0,0.0,0.0,1.0,4.0,0.0,0.0,0.0,0.0,0.0,0.0,1.0,5.0,0.0,0.0,1.0,0.0,0.0,0.0,0.0,33.0,0.0,3.0,0.0,1.0,0.0,1.0,1.0,12.0,0.0,0.0,0.0,0.0,0.0,1.0,2.0,8.0,0.0,0.0,1.0,1.0,0.0,0.0,0.0,32.0,0.0,1.0,0.0,1.0,0.0,3.0,1.0,15.0,1.0,0.0,0.0,0.0,0.0,2.0,3.0,10.0,0.0,0.0,1.0,2.0,0.0,0.0,0.0,46.0,0.0,2.0,1.0,2.0,0.0,0.0,1.0,4.0,0.0,0.0,0.0,0.0,0.0,1.0,3.0,4.0,0.0,0.0,1.0,2.0,0.0,0.0,0.0,34.0,0.0,0.0,1.0,2.0,0.0,1.0,1.0,23.0,3.0,2.0,0.0,3.0,0.0,3.0,4.0,5.0,0.0,0.0,0.0,1.0,0.0,1.0,1.0,28.0,0.0,0.0,1.0,1.0,1.0,0.0,0.0,26.0,1.0,0.0,1.0,1.0,1.0,2.0,7.0,4.0,0.0,0.0,0.0,0.0,0.0,1.0,0.0,38.0,1.0,1.0,0.0,0.0,0.0,0.0,0.0,216.0,42.0,18.0,14.0,12.0,11.0,11.0,32.0,41.0,5.0,3.0,0.0,6.0,5.0,4.0,4.0,60.0,0.0,0.0,2.0,2.0,0.0,2.0,1.0,18.0,4.0,0.0,0.0,0.0,1.0,0.0,8.0,4.0,0.0,1.0,0.0,0.0,0.0,0.0,0.0,40.0,2.0,0.0,0.0,0.0,1.0,0.0,0.0,39.0,8.0,0.0,0.0,0.0,1.0,4.0,2.0,20.0,0.0,0.0,2.0,2.0,0.0,0.0,1.0,39.0,0.0,2.0,1.0,3.0,0.0,1.0,1.0,19.0,2.0,0.0,0.0,1.0,0.0,3.0,6.0,13.0,0.0,0.0,2.0,2.0,1.0,0.0,1.0,25.0,0.0,1.0,0.0,2.0,0.0,1.0,4.0,14.0,0.0,0.0,0.0,1.0,0.0,3.0,2.0,10.0,0.0,0.0,0.0,2.0,1.0,0.0,0.0,38.0,0.0,2.0,0.0,3.0,0.0,2.0,4.0,5.0,0.0,0.0,0.0,0.0,0.0,0.0,0.0,4.0,0.0,0.0,2.0,0.0,0.0,0.0,0.0,34.0,0.0,3.0,0.0,2.0,0.0,0.0,2.0,6.0,0.0,0.0,0.0,0.0,0.0,2.0,2.0,8.0,0.0,0.0,1.0,1.0,0.0,0.0,0.0,40.0,0.0,1.0,0.0,2.0,0.0,0.0,0.0,21.0,1.0,0.0,0.0,0.0,0.0,3.0,3.0,18.0,0.0,0.0,1.0,2.0,0.0,0.0,1.0,45.0,0.0,0.0,1.0,3.0,0.0,3.0,2.0,13.0,1.0,0.0,0.0,1.0,1.0,1.0,6.0,13.0,1.0,0.0,1.0,1.0,0.0,0.0,0.0,34.0,0.0,1.0,1.0,1.0,0.0,0.0,4.0,20.0,1.0,0.0,0.0,2.0,0.0,6.0,5.0,12.0,0.0,0.0,0.0,2.0,0.0,0.0,1.0,43.0,1.0,1.0,1.0,2.0,0.0,0.0,2.0,76.0,13.0,5.0,3.0,3.0,1.0,7.0,15.0,26.0,3.0,1.0,0.0,5.0,1.0,3.0,3.0,42.0,1.0,1.0,2.0,2.0,1.0,1.0,2.0,239.0,44.0,20.0,14.0,11.0,11.0,19.0,36.0,55.0,3.0,3.0,0.0,10.0,10.0,2.0,5.0,56.0,0.0,2.0,1.0,5.0,2.0,1.0,4.0,110.0,32.0,16.0,12.0,3.0,4.0,8.0,12.0,22.0,1.0,4.0,1.0,0.0,2.0,1.0,1.0,41.0,1.0,0.0,3.0,1.0,1.0,2.0,0.0,49.0,7.0,7.0,9.0,0.0,2.0,5.0,15.0,12.0,1.0,1.0,1.0,1.0,0.0,1.0,1.0,35.0,1.0,0.0,1.0,1.0,2.0,1.0,0.0,27.0,6.0,0.0,1.0,3.0,0.0,4.0,6.0,15.0,1.0,0.0,1.0,1.0,0.0,1.0,2.0,24.0,0.0,1.0,0.0,2.0,1.0,1.0,1.0,8.0,0.0,0.0,0.0,0.0,0.0,0.0,3.0,11.0,0.0,0.0,0.0,0.0,0.0,0.0,0.0,32.0,0.0,1.0,0.0,2.0,1.0,0.0,3.0,5.0,0.0,0.0,0.0,0.0,0.0,1.0,0.0,5.0,0.0,0.0,0.0,0.0,1.0,0.0,0.0,35.0,0.0,1.0,0.0,0.0,0.0,1.0,3.0,3.0,0.0,0.0,0.0,0.0,0.0,0.0,0.0,0.0,0.0,0.0,1.0,0.0,0.0,0.0,0.0,37.0,0.0,1.0,0.0,1.0,0.0,0.0,0.0,6.0,1.0,0.0,0.0,0.0,0.0,1.0,2.0,4.0,0.0,0.0,1.0,0.0,0.0,0.0,0.0,41.0,0.0,0.0,1.0,3.0,0.0,0.0,0.0,7.0,0.0,0.0,0.0,1.0,0.0,0.0,1.0,14.0,0.0,0.0,0.0,1.0,0.0,0.0,0.0,41.0,0.0,0.0,0.0,4.0,0.0,1.0,1.0),.Dim=c(274,24)))
